# 1,7‐Digallatetracarborane: Between Nucleophilic *closo*‐Cluster and “Inverse Sandwich” Digallylene

**DOI:** 10.1002/anie.6667904

**Published:** 2026-06-11

**Authors:** Julijan Sarcevic, Tobias Heitkemper, Niklas Mehner, Maximilian Kubis, Christian P. Sindlinger

**Affiliations:** ^1^ Institut für Anorganische Chemie Universität Stuttgart Stuttgart Germany; ^2^ Institut für Anorganische Chemie RWTH Aachen University Aachen Germany

**Keywords:** borole, carborane clusters, gallium, main group chemistry, sandwich compounds

## Abstract

This manuscript describes the synthesis and chemistry of an unprecedented carborane cluster of low‐valent main‐group elements with a unique structural motif. On the basis of a dianionic borole π‐ligand platform (L = [R_5_(C_4_B)]^2−^) an “inverse sandwich”, Janus‐type digallylene complex L(Ga^+I^)_2_ featuring two nucleophilic Ga(I) ions was accessed. This compound can also be described as a *closo*‐type 1,7‐digallatetracarborane cluster. Preliminary reactivity studies on this new compound class have been conducted. Assessment of Lewis‐basicity in coordination chemistry with transition metals and main group Lewis‐acids indicate electronic communication between the Ga‐atoms. Oxidation with free borole (L) gives rise to bent digallocene L(Ga^+II^)–(Ga^+II^)L, the first group 13 element‐based dimetallocene‐type compound. Examples demonstrate that L(Ga^+I^)_2_ can behave as both a discrete digallacarborane cluster and a bis‐gallylene π‐complex capable of releasing Ga^+^.

## Graphical abstract

A dianionic π‐ligand is used to make a Janus‐type twofold complex with low‐valent gallium Ga(+I). The resulting compound can be seen as and reveals the reactivity of both: a Wade‐type *closo*‐carborane cluster with nucleophilic Ga‐sites, as well as π‐ligand stabilized Ga(I) atoms in an inverse sandwich compound.

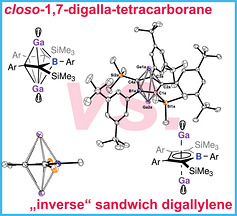

## 1 Introduction

The cyclopentadienyl ligand (Cp^−^) has been instrumental in the early developments of low‐valent main group organometallic species. Cp^−^ and its respective substituted derivatives (Cp^R^)^−^ allowed breakthrough discoveries and isolations of a number of fundamental low‐valent main group species [1, 2, 3, 4, 5, 6, 7, 8, 9, 10, 11]. This is due to the ligand's robustness caused by the effective delocalization of anionic charge, its tunable steric profile occupying a fair share of a half‐sphere around a respective bound atom, and its propensity to serve both as a σ‐ as well as a π−donor. Respective main group element (E) derivatives [(Cp^R^)_n_E] reveal a rich chemistry with regard to reactivity toward small molecules or applicability as metal‐centered donor ligands in coordination chemistry [12, 13, 14]. Over the years, by isoelectronic replacement of one or more carbon atoms with main group elements, a plethora of metallolyl derivatives more or less mimicking the electronic structure of Cp^−^ have been synthetically accessed and often successfully applied in coordination chemistry. For example, a rich chemistry of tetreloles ([(RC)_4_ER‘]^−^ or [(RC)_4_E]^2−^ E = Si‐Pb) has been developed [15, 16, 17, 18, 19, 20].

Respective five‐membered (multi‐)anionic π‐ligands containing boron have been extensively studied over the years. Ligand platforms of this type span a wide chemical range that progressively transitions with increasing boron content from discrete heterocycles such as boroles (C_4_B), diboroles (C_3_B_2_) [21, 22, 23] and triboroles (C_2_B_3_) [24, 25, 26] into the regime of well‐known anionic *nido*‐type carborane clusters (e.g., “dicarbollides”) [27, 28, 29].

**SCHEME 1:**
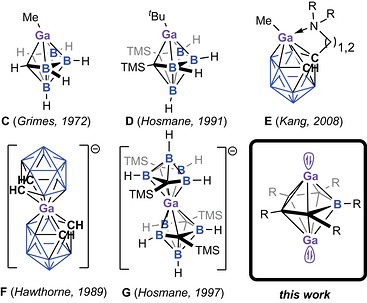
Selected previously structurally authenticated gallacarborane clusters. Blue edges in cluster polyhedra represent BH sites.

Our group aims to apply dianionic boroles as π‐ligands to support electron‐rich, low‐valent main group atoms. In the past, boroles had already been successfully used in transition metal [30, 31, 32, 33, 34, 35, 36, 37, 38, 39] and s‐block element coordination [32, 40, 41, 42, 43]. Compared to ionic alkali metal salts and highly efficient covalent transition metal bonding, the p‐block elements form expectably weaker covalent interactions with the borole π‐system. One key obstacle for the successful extension of the dianionic borole ligand into p‐block chemistry is its propensity to react as a reducing agent toward p‐block precursors rather than a metathetic source of the ligand [44, 45, 46]. Only recently successful strategies were developed to allow for isolation of divalent group 14 element E(+II) (E = Si, Ge, Sn) compounds of boroles [46, 47, 48, 49]. We would now like to report on the successful synthesis and chemistry of two borolediide ligands (**A** and **B**) supporting two mono‐valent Ga(I) centers in a pentagonal‐bipyramidal “inverse sandwich” compound, or 1,7‐Digallatetra‐carborane. Previously known gallacarborane‐clusters are limited to Ga(+III) compounds (selected examples **C**–**G**, Scheme 1) [50, 51, 52, 53, 54]. 1,7‐digallatetracarborane thus represents a significant extension of this chemical space and reveals broad and unique reactivity.

## 2 Results and Discussion

**SCHEME 2:**
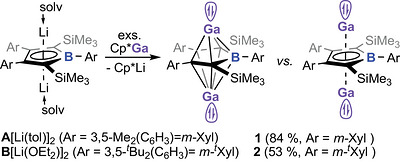
Synthesis of Ga(I) “inverse” borole sandwich compounds **1** and **2** and its alternative depiction as *closo*‐digallacarborane clusters.

When a solution of the toluene solvate of the dilithio derivative of borole **A**, **A**[(Li(tol)]_2_ in toluene was treated with an excess of Cp*Ga, clean formation of a new compound **1** was observed over the course of three days (Scheme 2). Compound **1** could be isolated in good yields of 84% as a colorless solid and identified to be the *closo*‐1,7‐digallatetracarborane cluster or the “inverse” sandwich compound of a borole dianion and two Ga(I) atoms capping both sides of the central five‐membered heterocycle. The proposed constitution of **1** is corroborated by mass spectrometry, NMR‐spectroscopy, elemental analysis, and (most convincingly) by subsequent reactivity studies (*vide infra*), but could not be unambiguously proven by X‐ray crystallography as we remained unable to obtain suitable single crystals.

**FIGURE 1:**
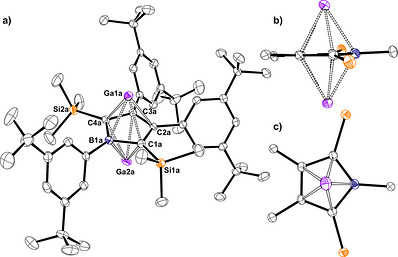
Perspectives on the molecular structure of *closo*‐digallacarborane **2**. Atomic displacement parameters shown at 50% probability. A second molecule in the asymmetric unit, hydrogen atoms, as well as minor disorder of organic moieties, are not shown for the sake of clarity. Selected bond lengths [Å] and angles [°] are: B1a–C1a 1.559(3), C1a–C2a 1.455(3), C2a–C3a 1.439(3), C3a–C4a 1.459(3); B1a–C4a 1.551(3), B1a–Ga1a 2.399(3), C1a–Ga1a 2.412(3), C2a–Ga1a 2.438(2), C3a–Ga1a 2.422(2), C4a–Ga1a 2.413(3), B1a–Ga2a 2.410(3), C1a–Ga2a 2.437(3), C2a–Ga2a 2.440(2), C3a–Ga2a 2.460(3), C4a–Ga2a 2.435(3). Ctr_BC4_–Ga1a 2.056, Ctr_BC4_–Ga2a 2.079, Ga1a–Ctr_BC4_–Ga2a 175.97°.

However, we also performed respective lithium–gallium metathesis reactions with the dilithio compound of a related borole substitution derivative **B**[Li(Et_2_O)]_2_ and successfully obtained the analogous digallium compound **2** in combined yields of 53%. Single crystals suitable for X‐ray diffraction of **2** were obtained and unambiguously reveal the proposed structure of the pentagonal bipyramid (Figure 1). We have no reason to assume a deviating structural motif for **1**. Monitoring the formation of **1** and **2** via NMR spectroscopy suggests a reasonable stepwise exchange of Li^+^ against Ga^+^ via a heterobimetallic mixed Li^+^/Ga^+^ intermediate. At the beginning, ^7^Li NMR spectroscopy reveals the rather highfield‐shifted resonance of **A**(Li)_2_ with *δ*(^7^Li) = −8.9 ppm (**A**[Li(tol)]_2_/−6.0 (**A**[Li(Et_2_O)]_2_). In the course of the reaction, an intermediate featuring a further ^7^Li resonance (shifted to higher frequencies around *δ*(^7^Li) = −3 ppm) along with a further set of ^1^H resonances characteristic for a borole containing species can be observed. We propose this intermediate to be the putative mixed Li^+^/Ga^+^ intermediate (see Figure SI4) [55]. We have tried to independently access and isolate this intermediate from reactions of **A**/**B**(Li_2_) with one equiv. of Cp*Ga and can confirm predominant formation of the respective Li^+^/Ga^+^ intermediate, but failed sofar to obtain pure material that would allow unambiguous characterization.

It is important to note that the solvation of the dilithio boroldiide starting material is crucial for the successful full conversion and itself depends on the substitution pattern of the borole. While the ether adduct **B**[Li(Et_2_O)]_2_ can be applied to slowly but reliably produce **2**, the presence of any amount of residual ether will suppress the successful full conversion and isolation of **1** from **A**(Li)_2_. For this reason, we specifically targeted to access the toluene solvate **A**[Li(tol)]_2_.

In our hands, the amount of ether donor present in the starting material, ranging from one down to approximately 0.2 ether molecules per lithium atom, simply depending on how excessively **A**[Li(Et_2_O)_n_]_2_ was dried in vacuo, significantly influenced the conversion times to **2**. The clear relationship is that the lower the ether content, the faster the conversion. We reason that increasing amounts of ether liberated during the course of the reaction stabilize the lithium–boroldiide interaction and progressively hamper efficient substitution of Li^+^ against Ga^+^.

The molecular structure of **2** features two Ga(I) atoms occupying the apical positions of a pentagonal bipyramid, with both Ga atoms sitting centrally over the [BC_4_]‐cycle as indicated by the Ga‐Ctr_BC4_‐Ga angle of approximately 176°. Ga1a sits slightly closer to the central borole moiety (Ctr_BC4_–Ga1a 2.056 Å) than Ga2a (2.079 Å). The bond lengths of the central [C_4_B]‐moiety (B–C_α_ 1.55 Å, C_α_–C_β_ 1.46 Å, and C_β_–C_β_ 1.44 Å; where C_α_ = C_1,4_ and C_β_ = C_2,3_ of the borole C_4_B) are very similar to those in the parent dilithio borolediide **A**[Li(tol)]_2_ (B–C_α_ 1.54 Å, C_α_–C_β_ 1.46 Å, and C_β_–C_β_ 1.43 Å; Figure SI1) indicating preservation of the borolediide moiety upon coordination of Ga(I).

NMR spectroscopic characteristics of **1** (**2**) reveal resonances at δ(^11^B) = 31.5 (32.4) ppm, roughly in the range of basal B‐atoms in known *closo*‐carboranes [52]. ^13^C_α_ and ^13^C_β_ resonate at 132.5 (132.7) and 145.5 (143.6) ppm, respectively. ^11^B‐ and ^13^C‐NMR shifts can be compared to previously reported low‐valent main‐group borole compounds [48, 49]. ^13^C‐resonances (particularly C_α_) reveal substantial low‐field shifts (e.g., compared to the parent dilithio compound **A**(Li)_2_ [C_α_ 104.6; C_β_ 128.5 ppm]). Attempts to detect ^71^Ga‐NMR resonances failed for **1** and **2,** likely due to unsuitable correlation times leading to signal broadening beyond recognizability, even at elevated temperatures [56]. This is fully in line with previous ^71^Ga‐NMR studies conducted on bulky Cp^R^Ga derivatives by Schnöckel and coworkers [57]. A number of neutral *closo* (**C**, **D**, **E**) and anionic *commo‐*galla(di)carborane (**F**, **G**) clusters (Scheme 1) have been previously prepared and structurally characterized; however, all of these are richer in boron and exclusively feature Ga(III) atoms [50, 51, 52, 53, 54]. In *ortho*‐(di)carbollide‐type ligand systems **C**–**F**, the Ga atom sits slightly dislocated from the center of the five‐membered coordination face, maximizing short contacts to more polarizable boron atoms [27].

This acentric location of the Ga atom in Ga(III) triboroldiide compounds **C**–**F** hints at a more evenly distributed charge density in borolediide compounds discussed here. As to be expected for larger ionic radii, the Ga(I) atoms in **2** reveal longer contacts to the borolediide (Ctr_BC4_–Ga 2.05–2.08) ligand compared to all cases of the Ga(III) atoms in **C**–**F** which sit much closer to the dianionic [C_2_B_3_]‐ligand (Ga–Ctr_B3C2_ approximately 1.78–1.93 Å).

Compound **1** revealed thermal stability in solution up to 80°C for several days without any sign of decomposition.

Within the small series of known, loosely related compounds, *closo*‐1,7‐digallatetracarborane clusters **1** and **2** uniquely feature nucleophilic Ga(I) atoms with lone‐pairs of electrons that can engage in further chemistry of the cluster. Recently, digallylenes featuring two flexibly bridged Ga(I) ions in spatial proximity have been shown to reveal cooperative reactivity [58, 59, 60, 61, 62].

The cation [GaCp*Ga]^+^, previously reported by Fischer and coworkers and later by Schorpp and Krossing, is iso‐electronically related to **1** and **2** [63, 64]. The Ga–Ctr_Cp*_ (2.228 and 2.237 Å) in this cation is considerably longer than in **2**, in line with increased electrostatic interactions of Ga^+^ with the dianionic ligand. Accordingly, the nature of [GaCp*Ga]^+^ was described to feature a rather weak and readily dissociating arene–Ga^+^ interaction between the neutral GaCp* molecule and a Ga(I) ion. Thus, [GaCp*Ga]^+^ reacts as a source of Ga^+^ and GaCp* individually, rather than as an authentic stable *closo‐*cluster or an “inverse sandwich” compound [63, 65]. EDA analysis for the bonding interactions between [Ga(H_4_C_4_BH)]^−^ and GaCp* with a Ga^+^ ion allows direct comparison between **H** and [GaCp*Ga]^+^.

**FIGURE 2:**
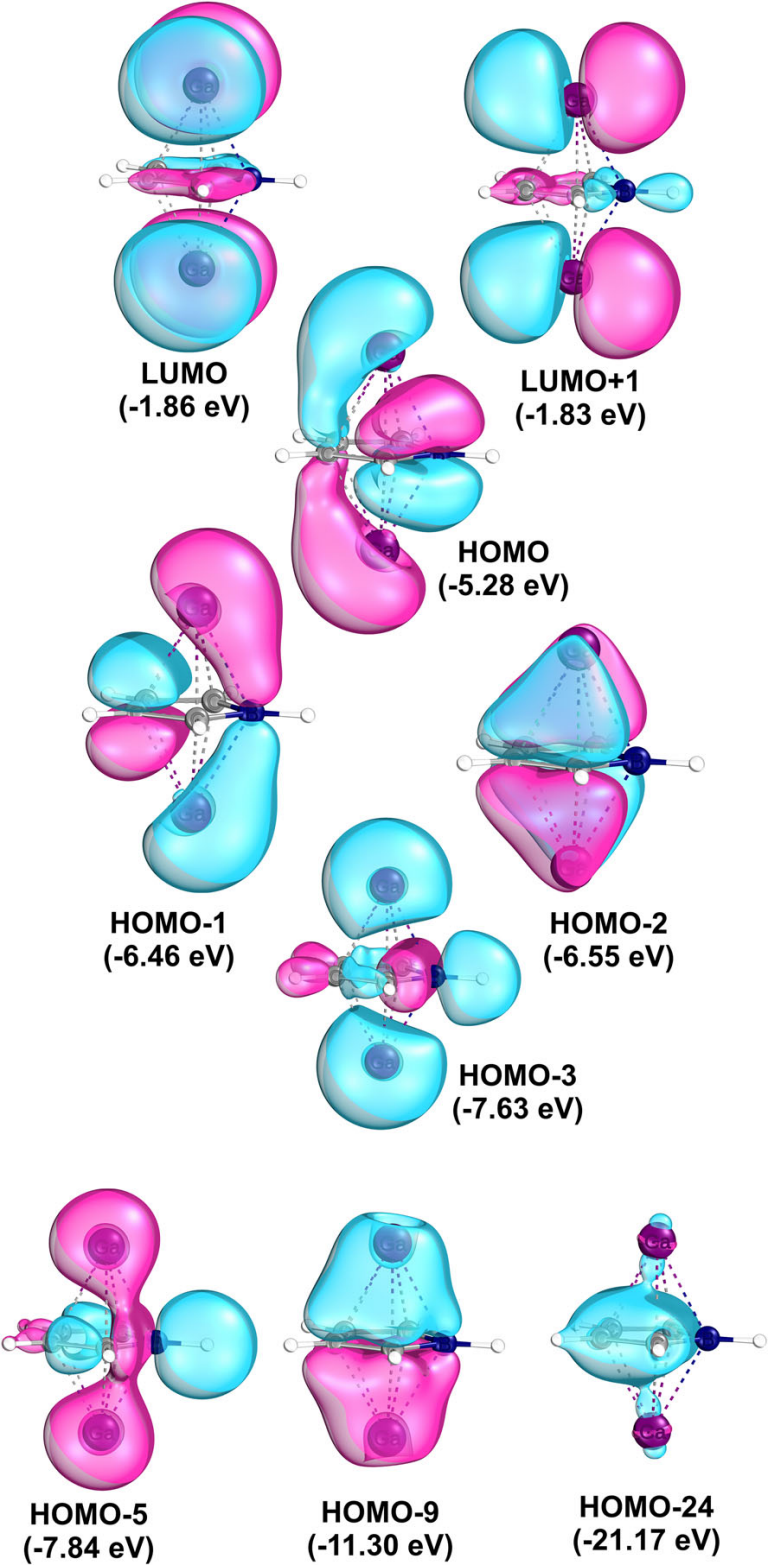
Frontier MO depictions and energies of the parent Ga(H_4_C_4_BH)Ga (H) (BP86‐D3BJ/def2‐TZVP). *Iso*‐density surfaces are drawn at the 80% level.

Next to obviously higher electrostatic interactions in **H**, the ΔE_orb_ value describing covalent interaction is significantly higher in **H** (−97.5 kcal mol^−1^) than in [GaCp*Ga]^+^ (−75.5 kcal mol^−1^) (see Table SI3). To gain further insight into the bonding in such *closo*‐1,7‐digallatetracarborane clusters, DFT (BP86‐D3BJ/def2‐TZVP) calculations were performed on **1**/**2** as well as on the parent model compound Ga(H_4_C_4_BH)Ga **H** and a summary on the frontier molecular orbitals of **H** is depicted in Figure 2 [66, 67, 68, 69, 70, 71, 72]. The MO pattern observed in **H** is representative for **1** and **2**, for which corresponding motifs were identified in respective energetic order (Figures SI23 and SI24).

The HOMO (−5.28 eV) for (H) resides between the two Ga‐atom and the C_α_BC_α_‐moiety of the borole and reveals strong contributions from both Ga atoms with significant lobes pointing outside the cluster. The HOMO‐1 (−6.46 eV) is similar in nature with σ‐type interactions of the Ga p‐orbitals and the C_β_C_β_‐moiety; however, it is energetically significantly lower and close in energy to HOMO‐2 (−6.55 eV). HOMO‐2 represents classic π‐bonding interactions of each Ga‐atom with the π‐system of the free borolediide ligand. However, the contribution of Ga‐atom orbitals to HOMO‐2 of (H) seems to be small. LUMO and LUMO+1 are essentially linear combinations of empty p‐orbitals at Ga‐atoms with little to no contribution from the C_4_B‐moiety. It is noteworthy that the energetic order of the HOMO levels of comparable interactions in isoelectronic [GaCp*Ga]^+^ and related Cp*Ga deviates significantly with the latter revealing the Ga–Cp* π‐bonding interactions being the HOMO/HOMO‐1 and the *σ*‐type interactions being more stable (HOMO‐2, Figures SI21 and 22). As expected for η^5^‐π‐complexes, intrinsic bonding orbital analysis reveals delocalized bonding involving two 5c2e‐bonds (Ga_2_BC_α_C_β_) and one 4c2e‐bond (Ga_2_C_β_C_β_) as well as two low‐lying Ga 4s‐orbital based lone pairs (Figure SI27). The HOMO of **1**/**2** is found at −4.87 eV. This points at a potential nucleophilic behavior of these Ga‐atoms as the respective HOMO in a comparable Si(II)‐borole compound had been calculated at −5.23 eV [48].

**SCHEME 3:**
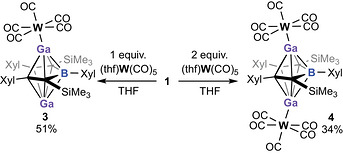
Mono‐ and bis‐adduct formation of **1** with [(thf)W(CO)_5_].

**FIGURE 3:**
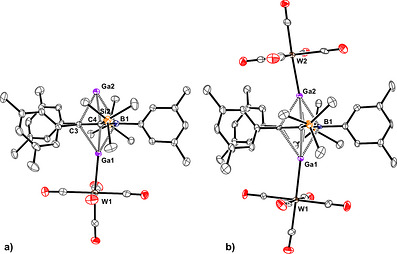
ORTEP depictions of the molecular structures of compounds **3** (a) and **4** (b). Atomic displacement parameters shown at 50% probability. Selected bond lengths [Å] and angles [°] are given. For **3**: B1–Ga2 2.403(2), C1–Ga2 2.444(2), C4–Ga2 2.435(2), C2–Ga2 2.473(2), C3–Ga2 2.446(2), B1–Ga1 2.247(2), C1–Ga1 2.261(2), C4–Ga1 2.291(2), C2–Ga1 2.371(2), C3–Ga1 2.358(2), Ga1–W1 2.5533(5), W1–C_ax_ 1.994(2), W1–C_eq_ 2.038(2)–2.048(2), Ctr_BC4_–Ga1 1.919, Ctr_BC4_–Ga2 2.078, Ga1‐Ctr_BC4_‐Ga2 172.4°, W1–Ga1–Ga2 173.06(1)°; for **4**: B1–Ga1 2.260(9), B1–Ga2 2.241(9), Ga1–W1 2.547(1), Ga2–W2 2.534(1), W1–C_ax_ 2.003(9), W1–C_eq_ 2.015(9)‐2.04(1), W2–C_ax_ 2.002(9), W2–C_eq_ 2.03(1)‐2.05(1), Ctr_BC4_–Ga1 1.934, Ctr_BC4_–Ga2 1.919, Ga1–Ctr_BC4_–Ga2 169.6°, W1–Ga1–Ga2 172.47(5)°, W2–Ga2–Ga1 170.18(4)°.

To probe the chemical accessibility of the lone pairs of electrons at the Ga(I) atoms, **1** was added to THF solutions of freshly generated tungsten pentacarbonyl complex (thf)W(CO)_5_. Depending on the applied equivalents of **1** to (thf)W(CO)_5_ we were able to isolate either the mono‐adduct **3** (**1**[W(CO)_5_]) or the bis‐adduct **4** (Scheme 3) as colorless solids in mediocre yields. The molecular structure of both compounds was confirmed by X‐ray diffraction (Figure 3), highlighting the potential of **1** to act as a Janus‐type ligand in coordination chemistry. Spectroscopically, the first adduct formation barely affects the NMR chemical shifts of potentially indicative nuclei of the starting material, and only the second adduct reveals a minor shift in the ^11^B resonance.

The ^11^B NMR signals are found at δ(^11^B) = 31.5 (**1**), 31.2 (**3**), and 27.2 (**4**) ppm. The characteristic CO‐stretching frequencies of the [W(CO)_5_] fragment are observed by ATR‐IR spectroscopy of solid samples at 2060/1977/1913 cm^−1^ (**3**) and 2065/1999/1916 cm^−1^ (**4**) indicating a very subtle blue‐shift upon addition of the second fragment in line with a decreasing σ‐donor strength. Albeit a weak effect, this points to the electronic dependency of the two Ga(I) atoms. Overall, the observed CO‐stretching frequencies are in the same range as previously observed for Cp*Ga–W(CO)_5_ (2065/1986/1910 cm^−1^) Cp^iPr5^Ga–W(CO)_5_ (2062/1913 cm^−1^) [73, 74].

*Closo*‐digallacarborane (**1**) is thus a slightly more potent σ‐donor than Cp*Ga when coordinating to one [W(CO)_5_] fragment but reveals almost identical stretching frequencies in its bis‐adduct. Computational assessment (BP86‐D3BJ/def2‐TZVPP/J CPCM = benzene) of this stepwise adduct formation affords a Δ*H*/Δ*G* of −21.2/−17.7 kcal mol^−1^ for the first adduct formation (**1** to **3**) and −16.9/−11.7 kcal mol^−1^ for the second adduct formation (**3** to **4**). Thus, both reactions are almost equally exothermic. As the enthalpy of adduct formation is barely affected, the slightly reduced free energy of the second addition is likely associated with strongly reduced degrees of freedom in **4**. The computationally approximated thermodynamics (BP86‐D3BJ/def2‐TZVPP in benzene) for the consecutive formation of **H‐W(CO)_5_** and **H‐[W(CO)_5_]_2_** from **H** and (thf)W(CO)_5_ are Δ*H*/Δ*G* −12.1/−11.9 kcal mol^−1^ for **H‐W(CO)_5_** and −5.3/−4.0 kcal mol^−1^ for **H‐[W(CO)_5_]_2_**. The second addition is thus significantly less exothermal than the first addition, in line with a reduced nucleophilicity. The respective MO with a suitable Ga‐contribution in **H‐W(CO)_5_** is HOMO‐3 found at −6.49 eV in line with significantly reduced Lewis‐basicity of the Ga(I) atoms upon adduct formation (see Figure 2).

In **3**, the HOMO is rather delocalized with only a reduced character of a Ga(I) lone‐pair of electrons at −5.44 eV, and lower in energy compared to the HOMO of free **1** (−4.87 eV).

The molecular structures of **3** and **4** are depicted in Figure 3. In mono‐adduct **3**, the coordinating Ga‐atom sits much closer to the ring‐plane (Ctr_BC4_–Ga1 1.92 vs. Ctr_BC4_–Ga2 2.08 Å) and Ga1 is slightly dislocated toward the C_α_‐B‐C_α_‐side. The W–C_ax_ distance (1.994(2) Å) is notably shorter than the bonds to the equatorially bound carbonyls indicating a notable *trans‐*influence of the gallium(I) donor.

For comparison, the W–CO distances in Cp*GaW(CO)_5_ are reported at 2.008(5) (*C*_ax_) and 2.036(7)–2.059(7) Å for C_eq_, and the Ga‐Ctr_C5_ is found at 1.915 Å [73]. In **4**, both [Ga–W(CO)_5_] fragments reveal virtually identical bonding features. The Ga–Ga–W angles deviate from linearity for both compounds **3** (173.1°) and **4** (172.5°/170.2°). This structural feature was previously rationalized in Cp*Ga–M(CO)_5_ (M = Cr, W) by packing effects [73, 75]. For **3**/**4** this deviation from linearity with a bending of the [W(CO)_5_] fragments away from the B‐atom is reasonably in line with the spatial orientation of the Kohn–Sham HOMO (see Figure 2 for representative HOMO of **H**) and thus likely not due to packing. Computationally probed adducts to parent cluster **H‐W(CO)_5_** and **H‐[W(CO)_5_]_2_** reveal even more pronounced bending (Ga–Ga–W angles of 153° and 156°, respectively).

**SCHEME 4:**
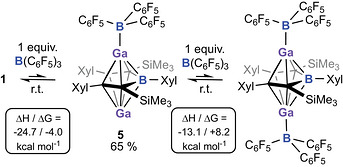
Formation of mono‐borane adduct **5**. Thermodynamics at BP86‐D3BJ/def2‐TZVPP (CPCM = benzene) level of theory at 298 K.

**FIGURE 4:**
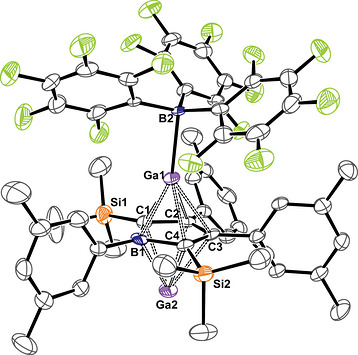
ORTEP depiction of the molecular structure of compound **5**. Hydrogen atoms and lattice pentane molecules are omitted for clarity. Atomic displacement parameters shown at 50% probability. Selected bond lengths [Å] and angles [°] are given. B1–C1 1.568(8), C1–C2 1.488(8), C2–C3 1.437(8), C3–C4 1.470(6), C4–B1 1.581(9), B1–Ga2 2.396(5), C1–Ga2 2.421(5), C2–Ga2 2.432(5), C3–Ga2 2.459(5), C4–Ga2 2.474(5), B1–Ga1 2.245(5), C1–Ga1 2.243(5), C2–Ga1 2.342(5), C3–Ga1 2.359(5), C4–Ga1 2.281(5), Ga1–B2 2.251(5), Ctr_BC4_–Ga1 1.902, Ctr_BC4_–Ga2 2.071, Ga1–Ctr_BC4–_Ga2 172.7°, Ctr_BC4_–Ga1–B2 170.6°.

The ability of Ga(I) atoms in **1** to act as a Lewis‐base was also probed against B(C_6_F_5_)_3_ as a well‐established main‐group Lewis‐acid (Scheme 4) [76, 77, 78, 79, 80]. The addition of one equiv. of B(C_6_F_5_)_3_ to digalla carborane **1** results in a clear high‐field‐shift of the ^19^F‐NMR resonance of the *para*‐F atom in C_6_F_5_ from δ(^19^F) = −141.9 ppm to −147.0 ppm, indicative of the formation of a Lewis acid‐base adduct **5** [**1·**(B(C_6_F_5_)_3_]. Variable temperature ^11^B NMR spectroscopy suggests a labile adduct. At ambient temperatures, two overlapping ^11^B signals are observed at δ(^11^B) = 36.5 and 31.8 ppm. Upon cooling, the signal assigned to the bound B(C_6_F_5_)_3_ shifts to lower frequencies (at −50°C, δ(^11^B) = −10.2 ppm) into a range expected for such adducts. Borane‐adduct **5** can be isolated in good yields of 65% and its structure was corroborated by X‐ray diffraction (Figure 4). As observed in tungsten complexes **3**/**4**, the Ga‐atom involved in adduct formation (Ga1) sits much closer to the central (C_4_B) moiety (Ctr_C4B_–Ga1 1.902 Å; Ctr_C4B_–Ga2 2.071 Å). Ga2 resides centrally over the (C_4_B)‐ring with small deviations between the individual Ga–(C_4_B) contacts, similar to those observed in cluster **1**. Ga1 sits slightly dislocated closer to the C_α_‐B‐C_α_‐side atop the C_4_B plane, indicated by comparatively long Ga1–C_β_ contacts.

A number of LGa^+I^ → B(C_6_F_5_)_3_ adducts are documented in the literature, and the Ga–B distances in these are found between 2.108(2) and 2.161(2) Å consistently shorter than in **5** (Ga1–B2 2.251(5) Å) [81, 82, 83, 84, 85]. The Ctr_C5_–Ga contacts in (CpGa) → B(C_6_F_5_)_3_ and (Cp*Ga)→B(C_6_F_5_)_3_ are, however, nearly identical to Ctr_BC4_–Ga1 [81, 84, 86].

Unlike mono‐[W(CO)_5_] adduct **3**, B(C_6_F_5_)_3_‐adduct **5** does not engage in further adduct formation, but instead free B(C_6_F_5_)_3_ is in dynamic exchange with bound borane in mono‐adduct **5**. This is corroborated by spectroscopic insight as upon stepwise addition of a further equivalent of borane B(C_6_F_5_)_3_ to solutions of **5**, ^19^F‐ NMR‐monitoring reveals only a single set of signals for a (C_6_F_5_)‐moiety.

With increasing borane content, the *ortho*‐, *meta*‐, and *para*‐^19^F‐resonances observed in **5** are increasingly shifted back toward free B(C_6_F_5_)_3_. In mixtures formally containing **1** and two equivalents of B(C_6_F_5_)_3_ the ^19^F resonances are observed close to the averages between pure **5** and B(C_6_F_5_)_3_.

Computational insight (BP86‐D3BJ/def2‐TZVPP/J CPCM = benzene) into the thermodynamics (at 298 K) of this reaction is shown in Scheme 4. Both adduct formations are predicted to be exothermal, but the second adduct reveals a notably reduced Δ*H*. The second addition is accordingly predicted endergonic (Δ*G* = +8.2 kcal mol^−1^) whereas the first addition is still exergonic (Δ*G* = −4.0 kcal mol^−1^) fully in line with the experimental observations. The HOMO in **5** strongly resembles the HOMO in **1** and should thus be considered to be involved in further Ga‐bonding.

**SCHEME 5:**
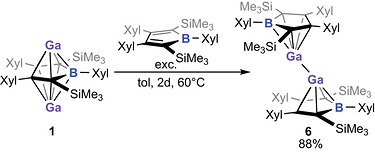
Formation of digallane‐type digallocene **6** by two‐electron oxidation of *closo‐*digallacarborane **1** with free borole **A**.

It is energetically predicted at −5.69 eV and thus more stabilized than the HOMO in mono‐[W(CO)_5_]‐adduct **3** (−5.44 eV, *vide supra*). The behavior of *closo*‐1,7‐digalla‐tetracarborane **1** toward B(C_6_F_5_)_3_ thus clearly indicates a dependency of the basicity of the two Ga(I) atoms and thus electronic communication between the Ga‐atoms within the cluster framework. We further anticipated studying electronic communication by the Ga(I) atoms and conducted cyclovoltammetric (CV) measurements, expecting to observe two distinct consecutive oxidative events for each individual Ga atom. However, in our hands, we failed to obtain meaningful CV data as reproducibly detrimental electrode‐fouling occurred with the formation of a metallic layer on the glassy carbon working electrode after a single cycle at both oxidative and reductive potentials. Yet, we succeeded in chemically oxidizing *closo*‐1,7‐digallatetra‐carborane **1** selectively by adding an excessive amount of free borole **A** (1,3,4‐Xyl_3_‐2,5‐(SiMe_3_)‐borole) to solutions of **1** with full conversion of **1** after two days at 60°C (Scheme 5).

**FIGURE 5:**
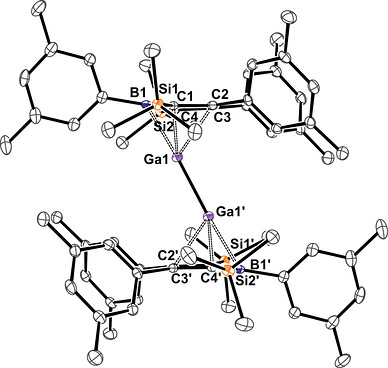
ORTEP depiction of the molecular structure of compound **6**. Hydrogen atoms and a lattice toluene molecule are omitted for clarity. Atomic displacement parameters shown at 50% probability. Selected bond lengths [Å] and angles [°] are given. Ga1–Ga1’ 2.3701(4), B1–C1 1.581(2), C1–C2 1.462(1), C2–C3 1.429(2), C3–C4 1.459(1), C4–B1 1.575(2), B1–Ga1 2.162(1), C1–Ga1 2.149(1), C2–Ga1 2.294(1), C3–Ga1 2.292(1), C4–Ga1 2.147(1), Ctr_BC4_–Ga1 1.804, Ctr_BC4_–Ga1–Ga1’ 145.2°, B1–Ga1–Ga1’ 176.8°.

Borole **A** serves as a two‐electron oxidant toward **1**, cleanly yielding the unprecedented digallocene **6** in excellent yields of 88% as an orange solid after the removal of excessive borole. An excess of free borole and elevated temperatures were applied, as consumption of **1** and formation of **6** under stoichiometric conditions at room temperature are drastically slowing down in the course of the reaction, and we did not observe complete conversion even after several weeks. Crystals suitable for X‐ray analysis were obtained from saturated solutions in toluene and reveal an unusual dimetallocene‐type structure (Figure 5). Both Ga(I) atoms in **1** are formally oxidized to Ga(II) and a Ga–Ga bond forms between two **A**(Ga)‐fragments. Only very few dimetallocene derivatives Cp^R^M‐MCp^R^ (M = Be^+I^ [87, 88], Zn^+I^ [89, 90], and Li^+^/(Al or Ga)^+I^ [74, 91]), are known and still represent synthetically very demanding and intriguing motifs. The latter “dimetallocenes”, however, do not constitute covalent M–M bonds but rather feature weak dative M → M’ interactions.

The digallane/digallocene compound **6** represents the first dimetallocene‐type sandwich compound with an unusual [M_2_]^4+^ core of low‐valent group 13 metals coordinated by the borolediide, electronically mimicking the typically applied cyclopentadienyls Cp^R^. Moreover, to the best of our knowledge, digallane **6** is the first structurally characterized dimetallocene of any group 13 metal. Known examples of classic Cp‐based dimetallocenes with a dicationic [M_2_]^2+^ moiety reveal essentially perpendicular orientation of the M–M bond with regards to the Cp plane (Ctr_CpR_–M–M ≈ 180°).

However, **6** reveals pronounced deviation of linearity/perpendicularity (Ctr_C4B_–Ga1–Ga1' = 145.2°). This deviation from the linear arrangement of Ctr_BC4_‐Ga‐Ga is reproduced by computational DFT optimization of the structure. This deviation likely stems from asymmetric charge distribution and shape of the relevant frontier molecular orbitals on the dianionic, *C*_2v_‐symmetric borole‐fragment compared to cyclopentadienide of *D*_5h_ symmetry. To allow for improved orbital interaction between Ga‐atom and the ligand, this results in a preferential tilted orientation of the Ga–Ga vector of the [Ga–Ga]^4+^ fragment toward the [C_α_BC_α_] moiety in the borolediide **A** ^2−^ rather than a perpendicular orientation toward the centroid of the heterocycle. It is worthy to note that the preference of a Ga atom to interact stronger with the [C_α_BC_α_] moiety increases with the formal oxidation number of Ga. Only naked Ga^+I^ atoms (as in **2**) reveal little spatial preference in their coordination behavior toward [C_4_B].

The structure of **6** resembles the only comparable digallane reported by Hosmane, Cowley and coworkers. They describe a twofold *nido*‐carborate [(BH)_4_(CSiMe_3_)_2_]^2−^ capped [Ga–Ga]^4+^ fragment as a low‐yielding side‐product obtained from the reaction of Na_2_[(BH)_4_(CSiMe_3_)_2_] with *^t^* BuGaCl_2_ (Ctr_C2B3_–Ga–Ga = 159.6°/164.4°) [92]. The Ga–Ga distance of **6** (2.3722(4) Å) is slightly longer than reported for Hosmane/Cowley's system (2.340(2) Å) but well in the common range (2.33–2.39 Å) of other digallanes for which diverse coordination environments are known [93, 94, 95, 96, 97, 98, 99]. We found neither experimental nor computational indication that could advocate for a putative dimeric hydride [(borole)Ga^+III^H]_2_ instead of the digallocene **6** [100].

**SCHEME 6:**
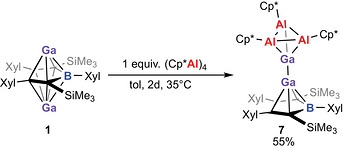
Formation of *conjuncto*‐cluster **7** by reaction of *closo‐*digallacarborane **1** with (Cp*Al)_4_.

Given the unique structure of **1**, we also anticipated accessing the putative homologous “inverse sandwich” 1,7‐dialuminatetra‐carborane for which we have so far not found viable synthetic routes. In an attempt to metathetically exchange Ga^+I^ versus Al^+I^, digallium cluster **1** was treated with (Cp*Al)_4_ [7, 9, 101]. Instead of Al/Ga exchange, major rearrangements occur, and the formation of a new, intensely red *conjuncto‐*cluster **7** is observed (Scheme 6).

In the presence of excessive AlCp*, the conversion of **1** to **7**, as monitored by NMR‐spectroscopy, progresses rather cleanly over the course of two days. The resulting brick‐red compound **7** can be isolated in moderate yields of 55%. However, solutions of isolated crystals of **7** reveal partial decomposition to the starting materials until mixtures of stable composition (approximately 0.8 equiv. of **7** vs. 0.2 equiv. of **1**) are obtained after approximately four days, strongly suggesting an equilibrium. This is corroborated by a rather weak exergonicity of the reaction, as predicted by DFT calculations [102].

**FIGURE 6:**
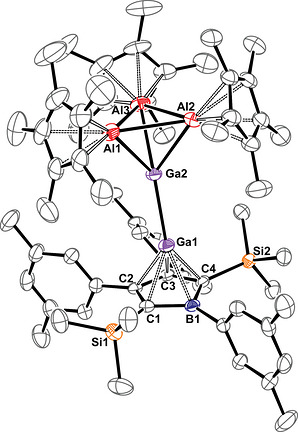
ORTEP depiction of the molecular structure of compound **7**. Hydrogen atoms and a lattice solvent molecule are omitted for clarity. Atomic displacement parameters shown at 50% probability. Selected bond lengths [Å] and angles [°] are given. Ga1–Ga2 2.5482(8), Ga2–Al1 2.554(1), Ga2–Al2 2.545(1), Ga2–Al3 2.577(1), Al1–Al2 2.760(1), Al2–Al3 2.749(1), Al3–Al1 2.764(1), B1–C1 1.553(4), C1–C2 1.451(4), C2–C3 1.429(3), C3–C4 1.455(4), C4–B1 1.549(4), B1–Ga1 2.240(3), C1–Ga1 2.192(2), C2–Ga1 2.254(3), C3–Ga1 2.280(3), C4–Ga1 2.214(3), Ctr_BC4_–Ga1 1.844, Ctr_BC4_–Ga1–Ga2 163.3°, B1–Ga1–Ga2 160.5(1)°.

The molecular structure of **7** was identified by X‐ray crystallography (Figure 6). One Ga atom is separated from the digallium cluster and incorporated in a tetrahedral [GaAl_3_] cluster fragment, revealing a Ga–Ga interaction with the remaining borole‐bound Ga‐atom. The Ga1–Ga2 separation (2.5482(8) Å) is significantly longer than in dimetallocene‐type digallane **6** (2.3701(4) Å).

**SCHEME 7:**
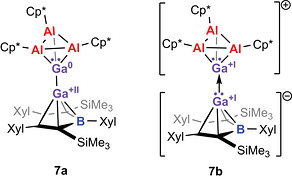
Alternative electronic and bonding descriptions of **A**GaGa(AlCp*)_3_ **7**.

An adequate bonding description of **7** is likely best addressed on the grounds of structural features. Two fundamental fragmentations of **7** seem plausible (Scheme 7). It can be seen as a borole‐derived gallyl gallylene **A**Ga^+II^–Ga^0^, which replaces one Cp*Al: fragment in the iconic parent tetrameric (Cp*Al)_4_ (**7a**, Scheme 7). Alternatively, **7** may be described as a formally monoanionic, Ga‐nucleophilic *nido*‐cluster [**A**Ga^+I^]^−^ forming an electrostatically dominated Ga–Ga bond to a Ga‐electrophilic [GaAl_3_Cp*_3_]^+^ fragment (**7b**, Scheme 7).

The borole‐bound Ga1 atom is located rather centered over the borole ligand, similar to what is otherwise only observed for the coordination mode of Ga^+I^ atoms as in **1** and thus advocates for the polar description **7b**. The much shorter Ga1–Ctr_C4B_ distance in **7** (1.844 Å) compared to **1** (2.056, 2.079 Å) is explained by the much stronger electrostatic interaction of a borole dianion to a single Ga^+^ atom. The electrostatic nature of the Ga–Ga bond in **7b** is further corroborated computationally. NBO analysis finds a polar Ga–Ga bond with 62% contribution of Ga1 (83% s‐orbital character, 17% p‐orbital character) and 38% from Ga2 (23% s‐orbital character, 77% p‐orbital character) [103, 104]. Furthermore, an EDA‐NOCV analysis of the fragments derived from homolytic versus heterolytic Ga–Ga bond splitting revealed smaller orbital interaction energy values (*E*_orb_) for the dative interaction of singlet fragments in **7b**, thus strongly advocating for **7b** as the appropriate bonding description [105, 106, 107, 108]. The results of the energy decomposition analysis are summarized in Table 1.

**TABLE 1:** Results of an EDA‐NOCV calculation to assess the nature of the Ga–Ga bond in **7** (BP86‐D3BJ/TZP)^a^.

Energy^b^	Homolytic bond scission AGa∙ ∙Ga(AlCp*)_3_ 7	Heterolytic bond scission [AGa]^−^ [Ga(AlCp*)_3_]^+^
Δ*E*_int_	−148.2	−102.5
Δ*E*_Pauli_	145.0	126.2
Δ*E*_disp_	−28.2	−28.2
Δ*E*_elstat_	−91.8	−125.0
Δ*E*_orb_	−173.2	−75.5

^a^ Performed on the structure obtained from single crystal diffractometry after optimization of H‐atoms.

^b^ All energies given in kcal mol^−1.^

**FIGURE 7:**
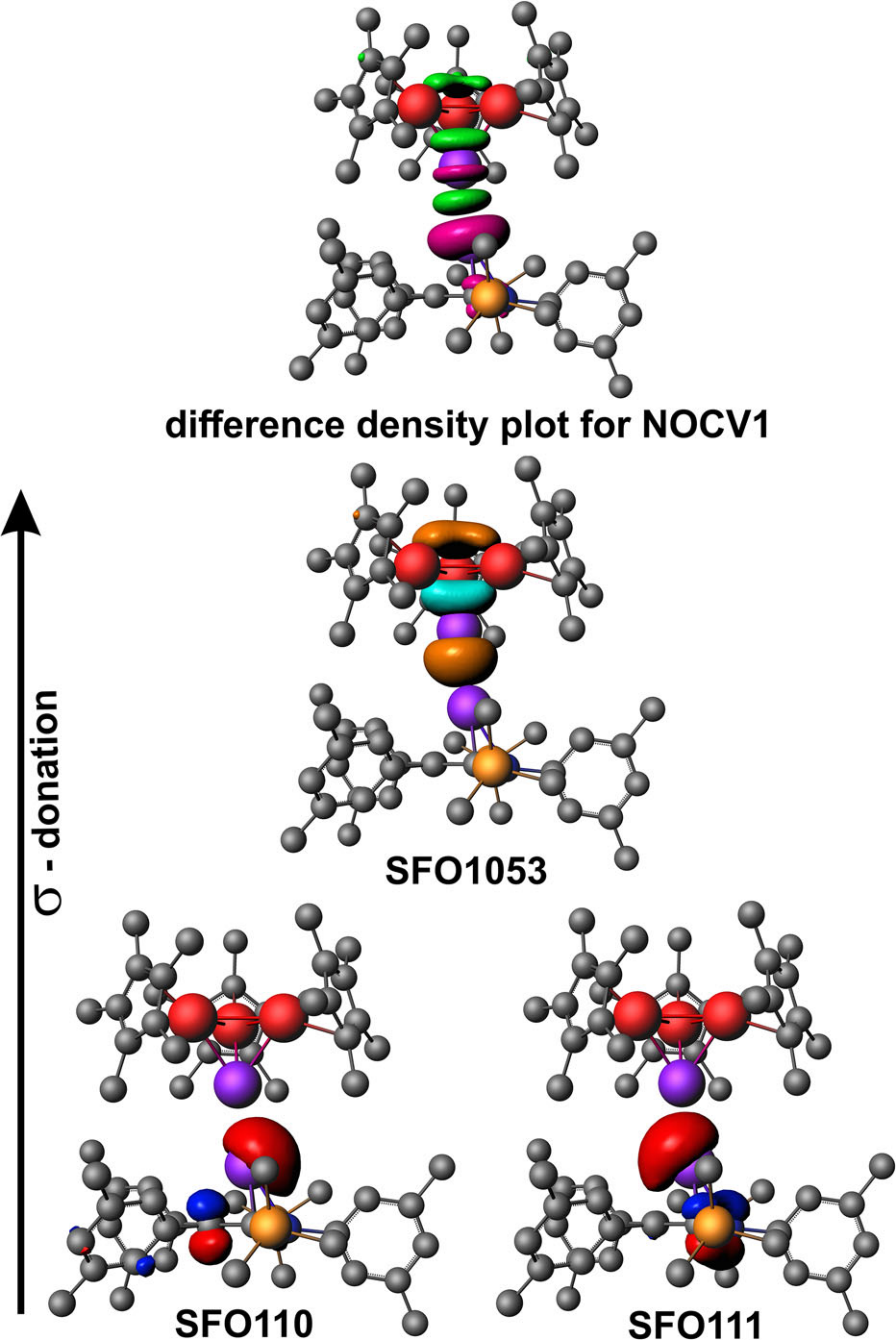
SFO interactions and difference density plot for the NOCV describing the dative Ga–Ga interaction in **7b**. SFO are drawn at an iso‐level of 0.05 a.u.; the difference density plot is drawn at 0.003 a.u. blue/red: occupied SFO, orange/turquoise unoccupied SFO, magenta: electron depletion, green: electron accumulation.

The only major contribution to bonding in **7b** according to EDA‐NOCV stems from the donation of the *nido*‐cluster [**A**Ga^+I^]^−^ fragments highest and second highest SFO into the lowest unoccupied SFO of the [Ga(AlCp*)_3_]^+^ amounting to 54 kcal mol^−1^ (Figure 7). The difference density plot of this NOCV clearly reveals charge donation into the Ga–Ga bond as well as into the [GaAl_3_] cluster, also enhancing the bonding between the tetrahedrally arranged cluster atoms.

The isolated cluster cation [Ga(AlCp*)_3_]^+^ was only recently described by Dabringhaus and Krossing and obtained from the reaction of arene complexes of Ga^+^ ([Ga(PhF)_2‐3_]^+^ [Al(OC(CF_3_)_3_)_4_]^−^) with (Cp*Al)_4_ [109, 110]. They found rather weak Ga–Ga interacting dimers of [Ga(AlCp*)_3_]^+^ in the solid state and in cold solutions and observed very long Al–Al distances (3.09(4) Å) in these [Ga(AlCp*)_3_]^+^ units, suggesting a pronounced character of three individual Al‐donors supporting a Ga^+^ atom rather than a multicenter‐bonded [GaAl_3_]^+^ cluster. The Al–Al distances in **7** are much shorter (average 2.755(6) Å) virtually identical to those reported by Dabringhaus and Krossing for a chelating Me_3_TACN (1,4,7‐trimethyl‐1,4,7‐triazacyclononane) donor adduct to [(Me_3_TACN)Ga(AlCp*)_3_]^+^ [109].

Apparently, the electron density provided by a single donor‐site ligand like Ga‐nucleophilic [**A**Ga]^−^ into the cluster LUMO of [Ga(AlCp*)_3_]^+^ (see Figure 7) is sufficient to enable multi‐center metal‐bonding within the [GaAl_3_]‐cluster. The ^27^Al‐NMR resonances in **7** are found at δ(^27^Al) = −43.4 ppm virtually identical to those reported for isolated [Ga(AlCp*)_3_]^+^ (−43.3 ppm) [109]. We observe no spectroscopic indication for dissociation of the ion pair in **7** in solutions at room temperature and expect an intact Ga–Ga bond. ^1^H‐NOESY NMR spectroscopy confirms spatial proximity of the Cp* moieties and the borole.

According to TD‐DFT, the red color of **7** observed at *λ* = 486 nm is in line with HOMO → LUMO+1 excitations and can be attributed to excitations from the Ga–Ga bond into the degenerate set of perpendicular empty p‐orbitals at the Al‐bound Ga‐atom.

**SCHEME 8:**
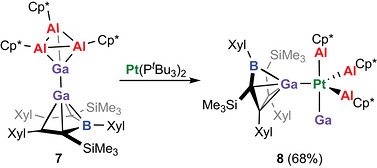
Reaction of **A**GaGa(AlCp*)_3_ **7** with [Pt(P*^t^* Bu_3_)_2_] to give complex **8**.

**FIGURE 8:**
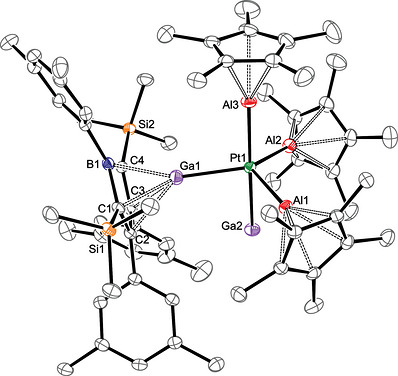
ORTEP depiction of the molecular structure of compound **8**. A second molecule in the asymmetric unit, hydrogen atoms, and co‐crystallized toluene molecules are omitted for clarity. Atomic displacement parameters shown at 50% probability. Selected bond lengths [Å] and angles [°] are given. Ga1–Pt1 2.3990(7), Ga2–Pt1 2.4680(6), Al1–Pt1 2.3083(8), Al2–Pt1 2.316(1), Al3–Pt1 2.361(1), B1–C1 1.568(4), C1–C2 1.467(4), C2–C3 1.421(3), C3–C4 1.458(4), C4–B1 1.553(4), B1–Ga1 2.234(3), C1–Ga1 2.231(3), C2–Ga1 2.378(3), C3–Ga1 2.395(3), C4–Ga1 2.225(3), Ctr_BC4_–Ga1 1.911; Ga1–Pt1–Ga2 83.36(2)°, Ga2–Pt1–Al3 176.13(3)°, Ga1–Pt1–Al1 116.00(3)°, Al1–Pt1–Al2 115.14(4)°, Al2–Pt1–Ga1 124.04°, Ctr_BC4_–Ga1–Pt1 167.1°.

We reason that the formation of **7** occurs by digallium compound **1** reacting as a source of Ga^+^ ions toward (Cp*Al)_4_ and thus reveals reactivity rather assignable to an inverse sandwich complex of two individual Ga^+^ ions than a *closo*‐1,7‐digallatetra‐carborane cluster. This is further corroborated by the reactivity of **7** toward [Pt(P*^t^* Bu_3_)_2_] (Scheme 8). Portion‐wise addition of **7** to solutions of [Pt(P*^t^* Bu_3_)_2_] in toluene over a period of approximately 1 h allows for isolation of platinum complex **8** as a pale yellow‐brown powder in yields of 68%. Crystals of **8** suitable for X‐ray diffraction were grown from saturated solutions in toluene at −40°C. The molecular structure is shown in Figure 8.

The platinum atom is inserted into the entire [Ga(AlCp*)_3_]^+^ cluster with all parts serving as independent ligands, forming a rather unusual penta‐coordinate Pt^0^‐complex with distorted trigonal‐bipyramidal coordination geometry. Two (AlCp*) and an isoelec‐tronic, yet formally anionic [**A**Ga]^−^ *nido*‐cluster occupy the equatorial plane while the axial positions are occupied by a further (AlCp*) and a naked Ga^+^ ion. The ligands of the equatorial plane are slightly vaulting over the Ga^+^ ligand site with Ga2‐Pt‐L_eq_ angles between 80° and 85° likely due to repulsion by the sterically more demanding Cp* moiety. The bond lengths within the [**A**Ga]^−^ fragment are similar to those observed in **7** with the only notable difference being the elongated Ga–C_β_ distances which are approximately 0.1 Å longer.

Cp*E (E = Al, Ga, In) ligands coordinating to zero‐valent Ni, Pd, or Pt metal atoms were previously reported by the groups of Jutzi and Fischer [75, 111, 112, 113, 114, 115, 116, 117]. Most suitable for comparison, Fischer and coworkers reported the formation of a cationic penta‐coordinate [(Ga)Pt(GaCp*)_4_]^+^ from the reaction of [Pt(GaCp*)_4_] with [GaCp*Ga][BAr^F^], the latter essentially serving as a source of the Ga^+^ ligand [65].

Complex **8** thus represents a further, yet neutral, addition to the small number of compounds featuring a naked Ga^+^‐ligand coordinating to a transition metal [65, 116, 118, 119].

The Pt–Ga_terminal_ distance to the axially located Ga‐atom (Pt1–Ga2: 2.4680(6) Å) in **8** is comparable to the one observed by Fischer (2.4593(9) Å), but the equatorially located **A**Ga1–Pt1 distance (2.3990(7) Å) is notably longer than the respective equatorial Pt–(GaCp*) distances (between 2.3474(7) and 2.3577(8) Å) [65]. This can, again, be rationalized by a much more sterically demanding profile of the borole‐derived [**A**Ga]^−^ compared to a neutral (Cp*Ga) ligand. We anticipate the bonding situation in **8** to be identical to Fischer's essentially isoelectronic all‐Gallium [(Ga)Pt(GaCp*)_4_]^+^, for which computational assessment suggested the terminal Ga^+^ ligand to act as a σ‐ and π‐acceptor ligand to the [L_4_Pt]^0^ fragment [65].

**SCHEME 9:**
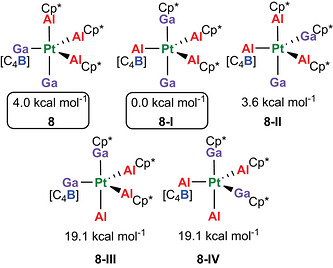
Relative stabilities (Δ*G* ^298^) of (putative) isomers of complex **8** at r^2^SCAN‐3c (CPCM = benzene) level of theory.

^1^H‐NMR spectroscopy at room‐temperature reveals sharp signals for the [**A**Ga]^−^ fragment along with a single, notably broadened signal for the Cp*Al‐moiety. This could either indicate structural exchange between equatorially and axially bound AlCp* ligands in solution or even include dynamic Cp* migration from Pt‐bound Ga^+^ and Al^+^ atoms. No signals in ^71^Ga‐ and ^195^Pt‐NMR could be detected. No signal is observed in ^27^Al‐NMR spectroscopy (despite precedence for observable signals in chemically related systems) [115]. This further hints at a broadening beyond recognizability by dynamic exchange between AlCp* ligands, Cp* and could potentially even involve exchange of the borole–bound group 13 element. Further experimental hints for such behavior stem from X‐ray diffraction. Refinement of the data reveals a slight lack of electron density in the F_o_‐F_c_ map at the **A**Ga1 position, suggesting a potential occupation of this site by an Al atom, whereas the axial AlCp* moiety may be partially occupied by GaCp* (Figure SI20). The extent of this potential positional disorder varied slightly between a number of (mediocre) data sets obtained for this compound in the course of our investigations. Scheme 9 summarizes relative stabilities (r^2^SCAN‐3c level of theory) of putative isomers of **8** with regard to Al versus Ga exchange.

Next to the obviously dominating product **8**, isomer **8‐I** represents the isomer reasonably associated with the observed residual/lacking electron density in X‐ray refinements of **8**. Isomer **8‐I** has both axial sites occupied by Ga‐atoms and is the most stable isomer within this series of putative compounds (**8** to **8‐IV**) according to DFT. While Al‐atoms seem to be preferred in equatorial positions and slightly preferred on the borole π‐platform, the Al^+^‐atom as a terminal ligand is energetically clearly disfavored.

It is worth mentioning that such isomers of **8** with regard to the individual occupation of sites between Al and Ga may either result from dynamic exchange in **8**, or result from minor contributions of isomers of the starting material **7**, as Dabringhaus and Krossing already reported on isomerization (i.e., Cp* migration) in [GaAl_3_Cp*_3_]^+^ [109]. Throughout our studies, we have not gathered any further spectroscopic evidence of any of the putative isomers **8‐I** to **8‐IV**.

## 3 Conclusion

We have successfully prepared the first complex of a borole dianion coordinating to two low‐valent Ga^+^ atoms. This unprecedented *closo*‐1,7‐digalla‐tetracarborane cluster was shown to reveal a rich multifacetted chemistry between a molecular Wade‐type cluster and an “inverse” sandwich complex of individually chemically active Ga^+^ atoms. Depending on the reagents and reaction conditions, both Ga(I) ions of the digallylene cluster can individually but not independently serve as nucleophilic sites toward tungsten carbonyl fragments rendering **1** a Janus‐type metal atom‐based ligand. It was shown that the individual basicity of the second Ga‐atom is affected upon involvement of the first Ga‐atom in Lewis‐acid base reactions, thus proving a certain degree of electronic communication between the Ga‐sites. The *closo*‐1,7‐digalla‐tetracarborane can be selectively oxidized with free borole to give an unprecedented dimetallocene of the group 13 elements. Toward (Cp*Al)_4_, *closo*‐1,7‐digalla‐tetracarborane serves as a Ga^+^ donating agent, resulting in an ion pair of an anionic *nido*‐1‐galla‐tetracarborate cluster, which is isoelectronic to iconic neutral Cp*E, and the recently described [GaAl_3_Cp_3_*]^+^ cation. This latter compound was then found to form an intriguing penta‐coordinate complex with Pt^0^ with a rare terminal Ga^+^ ligand further stabilized by Cp*Al and *nido*‐1‐galla‐tetracarborate ligands.

## Author Contributions

**Julijan Sarcevic**: investigation, validation, writing – review and editing, formal analysis, data curation. **Tobias Heitkemper**: investigation, validation, writing – review and editing, formal analysis, data curation. **Niklas Mehner**: investigation, validation, formal analysis, data curation. **Maximilian Kubis**: investigation. **Christian P. Sindlinger**: conceptualization, investigation, validation, data curation, supervision, project administration, funding acquisition, writing – original draft, writing – review and editing, resources.

## Conflicts of Interest

The authors declare no conflicts of interest.

## Supporting information



Further supporting information, including a detailed methodological summary, synthetic procedures, spectroscopic and crystallographic evidence on the compounds, as well as computational details, is provided in the ESI file. CIF files of the structures reported have been deposited with the CCDC database (deposition numbers 2519914–2519921) [120].
**Supporting File 1**: anie72722‐sup‐0001‐Data.zip.

**Supporting File 2**: anie72722‐sup‐0002‐SuppMat.pdf.

## Data Availability

Data are provided with this manuscript as Supporting Information. Crystallographic information is provided in the CSD database. Further data can be provided by the authors upon request.
