# Supplementary material for: 1,7‐Digallatetracarborane: Between Nucleophilic closo‐Cluster and “Inverse Sandwich” Digallylene
Source: Angew Chem Int Ed Engl. 2026 Jun 11;65(33):e6667904. doi: 10.1002/anie.6667904 (PMC13452514; doi:10.1002/anie.6667904)
Supplement: Supplementary file 1 [file ANIE-65-e6667904-s002.pdf]

Supporting Information

1,7-Digallatetracarborane: between nucleophilic *closo*-Cluster and "inverse sandwich" digallylene.

Julijan Sarcevic[a], Tobias Heitkemper[a], Niklas Mehner[a], Maximilian Kubis[b] and Christian P. Sindlinger[*,a]

[a]Institut für Anorganische Chemie, Universität Stuttgart, Pfaffenwaldring 55, 70569 Stuttgart.

[b]Institut für Anorganische Chemie, RWTH Aachen University, Landoltweg 1a, 52074 Aachen.

Table of Contents

Experimental Details 3
General Information 3
IR spectroscopy 3
UV/Vis spectroscopy 3
Mass spectrometry 3
NMR spectroscopy 3
Starting materials and reagents 4
Synthesis and Analytical Data 5
Compound ALi_2×(tol) 5
Analytical Data for Compound ALi_2×(tol) 5
Molecular structure of Compound ALi_2×(tol) 6
Spectra Plots for Compound ALi_2×(tol) 7
Compound 1 10
Analytical Data for Compound 1 10
Spectra Plots for Compound 1 11
Compound 2 14
Analytical Data for Compound 2 14
Molecular structure of Compound 2 15
Spectra Plots for Compound 2 16
NMR-monitoring of reaction progress from $BLi_2(OEt)_2$ to 2 18
Compound 3 19
Analytical Data for Compound 3 19
Molecular structure of Compound 3 20
Spectra Plots for Compound 3 21
Compound 4 25
Analytical Data for Compound 4 25
Molecular structure of Compound 4 26
Spectra Plots for Compound 4 27
Compound 5 31
Analytical Data for Compound 5 31
Molecular structure of Compound 5 32
Spectra Plots for Compound 5 33

Compound 6 37
Analytical Data for Compound 6 37
Molecular structure of Compound 6 38
Spectra Plots for Compound 6 39
Compound 7 42
Analytical Data for Compound 7 42
Molecular structure of Compound 7 43
Spectra Plots for Compound 7 44
Compound 8 47
Analytical Data for Compound 8 47
Molecular structure of Compound 8 48
Spectra Plots for Compound 8 49
Crystallographic Details 52
General Data Acquisition and Processing 52
Crystallographic and Refinement Details A(Li(tol))$_2$ 52
Crystallographic and Refinement Details 2 52
Crystallographic and Refinement Details 3 53
Crystallographic and Refinement Details 4 53
Crystallographic and Refinement Details 5 53
Crystallographic and Refinement Details 6 54
Crystallographic and Refinement Details 7 54
Crystallographic and Refinement Details 8 54
Tabulated Crystallographic Details 56
Computational Details 57
Structure Optimisation, Frequency Calculation and Thermochemical Approximations 57
NBO, NLMO and NRT Analyses 58
ETS-NOCV 58
Frontier molecular orbitals of Cp*Ga, [GaCp*Ga]$^+$, H and 1 58
Summary and comparison of HOMO of 1, 3 and 5 62
Frontier molecular orbitals of 6 62
IBO-Analysis of H 63
ETS-NOCV on Ga(H$_4$C$_4$BH)Ga H and [GaCp*Ga]$^+$ for comparison 64
Experimental *vs*. DFT-optimised (gas phase) structures with and without dispersion of Compound 7 65
XYZ-coordinates of optimised structures 66
Literature 80

Experimental Details

General Information

All manipulations requiring handling under inert conditions were carried out under argon atmosphere using standard Schlenk techniques or an MBraun Glovebox with an Ar atmosphere. If not otherwise indicated, solvents for Schlenk-type syntheses were obtained from either a GS or an InnovativeTechnologies solvent purification system (SPS). Solvents used in a glovebox were routinely purified, degassed three times using freeze-pump-thaw cycles (fpt) and stored according to the following table:

Table SI1 Solvent purification procedures.

Solvent	Distilled from	Storage
Pentane, per synthesis	Na/K alloy	in a glovebox
Hexane , p.a. grade	K	in a glovebox
Et_2O, non-stabilized	predried from SPS; $LiAlH_4$	in a glovebox
THF, 99.5% grade	predried from mol. sieves (4 Å).; K	in a glovebox
Toluene, per synthesis	Na	in a glovebox
Benzene, p.a. grade	predried from mol. sieves (4 Å).; K	in a glovebox
Fluorobenzene	P_2O_5	in a glovebox
CH_2Cl_2, per synthesis	CaH_2	in a glovebox

Dichloromethane-d_2 was distilled from CaH_2, THF-d_8 was dried over $LiAlD_4$ and vacuum transferred, benzene-d_6 was distilled from potassium, and solvents were degassed and stored in a glove box.
Elemental analysis was performed by the Institut für Anorganische Chemie, Universität Stuttgart.

IR spectroscopy

IR spectra were obtained on a Bruker ALPHA FT-IR spectrometer with Platinum ATR module.

UV/Vis spectroscopy

UV/Vis spectra were obtained using a PerkinElmer Lambda25 UVVis spectrometer and quartz cuvettes fitted with an air-tight Young-type teflon valve or inside an MBraun glovebox with an Avantes Avalight-DH source coupled with an Avantes StarLine AvaSpec-2048 detector. Normalized plots of the s UV/Vis spectra are given below.

Mass spectrometry

Mass spectra were recorded by the analytical facilities run by the Institute of Organic Chemistry at the University of Stuttgart applying either a APCI ionization method on a microTOF-Q instrument by Bruker Daltonics or a Liquid Injection Field Desorption Ionisation-technique on a Exactive Plus Orbitrap MS by Thermo Fisher Scientific instrument with an inert-sample application setup under argon atmosphere. The injection capillary was washed several times with dry, distilled and inertly injected toluene before the samples were injected. Samples usually had a concentration of 1 – 2 mmol/L in toluene with a minimum amount of fluorobenzene added to guarantee solubility and were prepared in a glovebox.

NMR spectroscopy

NMR spectra were recorded with either

in Göttingen: a Bruker Avance III 400 NMR spectrometer equipped with a 5 mm BBFO ATM probe head and operating at 400.13 (^{1}H), 100.61 (^{13}C), 128.38 (^{11}B) and 376.45 MHz (^{19}F) along with a variable temperature set-up or a Bruker Avance Neo 400 NMR spectrometer with a CryoProbeProdigy BB ATM probe head operating at 400.25 MHz (^{1}H) and 100.65 MHz (^{13}C) or a Bruker AVIII HD 500 NMR spectrometer with a CryoProbeProdigy ATM probe head and operating at 500.25 (^{1}H), 125.80 (^{13}C), 160.50 MHz (^{11}B) and 99.37 MHz (^{29}Si) or a Bruker Avance III HD spectrometer operating at 116.64 MHz (^{7}Li).

in Stuttgart: a Bruker Avance 400 NMR spectrometer equipped with a 5 mm BBO ATM probe head and operating at 400.13 (^{1}H), 100.61 (^{13}C), 128.38 (^{11}B), 155.51 (^{7}Li), 104.27 (^{27}Al), 121.97 (^{71}Ga) and 79.49 MHz (^{29}Si) along with a variable temperature set-up or a Bruker Avance-II 250 NMR spectrometer equipped with a 5 mm BBO ATM probe head and operating at 250.13 (^{1}H), 62.90 (^{13}C), 80.25 (^{11}B), 65.18 (^{27}Al), and 49.69 MHz (^{29}Si) or a Bruker Avance III HD 700 NMR spectrometer equipped with a 5 mm QCI cryo-probehead operating at 700.36 (^{1}H) and 176.12 MHz (^{13}C).

Chemical shifts are reported in δ values in ppm relative to external Me_4Si and, if not otherwise stated, referenced using the chemical shift of the solvent ^{2}H lock resonance frequency and Ξ = 19.867187% for ^{29}Si, Ξ = 38.863797 % for ^{7}Li, Ξ = 32.083974% for ^{11}B, and Ξ = 94.094011 % for ^{19}F.[1] ^{1}H and ^{13}C spectra have been referenced on specific values for the respective solvent signal. The proton and carbon signals were assigned where possible via a detailed analysis of ^{1}H, ^{13}C, ^{1}H-^{1}H COSY, ^{1}H-^{1}H NOESY, ^{1}H-^{13}C HSQC, ^{1}H-^{13}C HMBC NMR spectra.

Independent attempts to obtain ^{71}Ga-NMR signals for Gallium clusters (e.g. **2**) were also kindly made by Dr. Klaus Eichele, Institute of Inorganic Chemistry, University of Tübingen, corroborating our own unsuccessful attempts to detect respective resonances.

Young-type teflon-valve borosilicate NMR tubes have been used throughout the study.

Starting materials and reagents

$(Cp^*Al)_4$[2] and Cp*Ga[3] were prepared along procedures as reported in the literature.

1,3,4-(3',5'-*t*$Bu_2(C_6H_3)$-2,5-($SiMe_3$)-borole **B** was prepared as reported.[4] Dilithio salt of borole **B**, $[Li(OEt_2)]_2$[**B**] prepared as recently reported.[5]

Synthesis of Borole A

1,3,4-(3',5'-$Me_2(C_6H_3)$-2,4-($SiMe_3$)-borole **A** was previously reported by our group.[6] For this study, the synthesis was updated and optimized as follows:

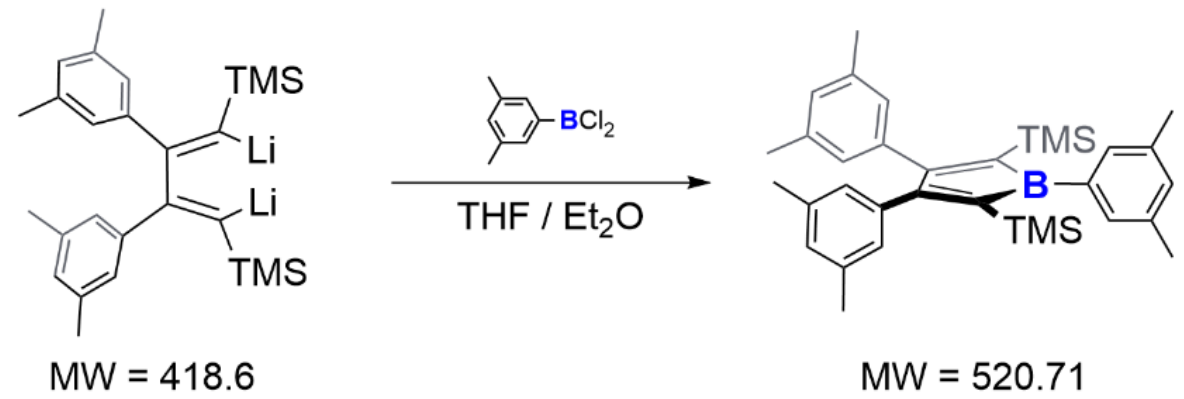

In a glovebox, a solution of *m*-xyleneborondichloride ($3,5\text{-}Me_2(C_6H_3)BCl_2$)[4, 7] (321.0 mg, 1.901 mmol, 1 eq.) in diethylether (9 mL) and THF (2 mL) was added to a stirred solution of dilithiobutadiene (1,4-dilithio-1,4-bis(trimethylsilyl)-2,3-(3',5'-dimethylphyenyl)-buta-1,3-diene)[7] (795.5 mg, 1.901 mmol, 1 eq.) in diethylether (9 mL) and THF (2 mL). The initially red suspension was stirred at ambient temperature for 30 min. The now off-white suspension was filtered using a syringe filter equipped with a glass fiber pad (Whatman GF/B) to give a yellowish-orange filtrate which was subsequently concentrated in vacuum until a viscous oil was obtained. 2-Methyl-tetrahydrofurane (2 mL) was added and subsequently removed *in vacuo* to co-evaporate THF. Addition and and removal of 2-methyl-tetrahydrofurane was repeated to ensure complete sufficient removal of THF yielding a partly crystalline solid which was then dissolved in hexane (5 mL) and filtered through a syringe filter equipped with a glass fiber pad (Whatman GF/B) to give a red filtrate. The hexane was removed under reduced pressure, hexane (5 mL) was added again and subsequently removed to give a dark red, crystalline solid at the walls of the vial. The solid was carefully washed with cold hexane (2 mL, –40°C) after which the crystalline solid turned bright red. The solid was ground into a powder using a spatula and then thoroughly dried *in vacuo* to give the product **A** in the form of a red powder (717.2 mg, 1.377 mmol, 72 %).

Note: 2-Methyl-tetrahydrofurane is used to remove residual THF from the product by co-evaporation. THF itself binds strongly to the Lewis-acidic borole and cannot be reliably removed *via* co-evaporation with hexane. Excess 2-Methyl-tetrahydrofurane partially replaces residual THF but itself forms less stable adducts to the borole and can thus be subsequently removed through co-evaporation with hexane.The applied mixture of diethylether and THF is crucial to suppress the formation of by-products which will form if either of the solvents were used solely.

Analytical data of **A** were in full agreement with the data previously published from otherwise obtained material.[6]

Synthesis and Analytical Data

Compound ALi_2×(tol)

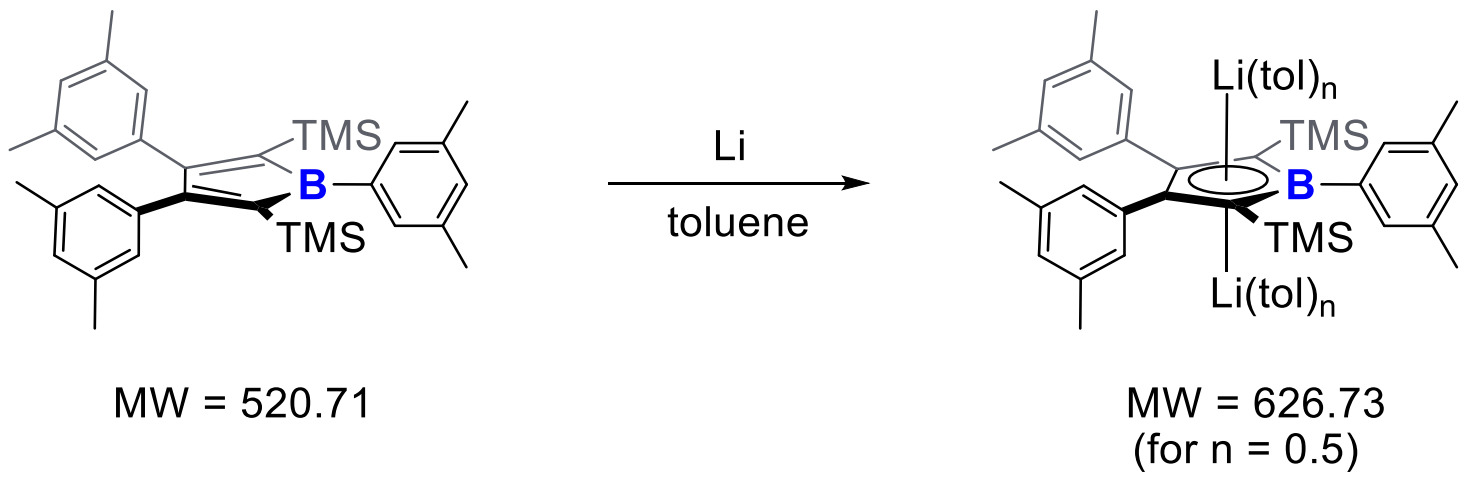

In a glovebox, clips of lithium wire (19.1 mg, 2.768 mmol, 2 eq.) were spreaded in a vial with a spatula to give finely distributed lithium flakes. A solution of the borole **A** (704.9 mg, 1.354 mmol, 1 eq.) in toluene (5 mL) was added and the mixture was left to stir over night. The reddish brown mixture with leftover lithium flakes was filtered using a syringe filter equipped with a glass fiber pad (Whatman GF/B) and the resulting filtrate was concentrated under reduced pressure until a brown oil was obtained. Hexane (2 × 2 mL) was added and subsequently removed under reduced pressure to give a yellow powder. The powder was transfered into a syringe filter equipped with a glass fiber pad (Whatman GF/B) and washed with pentane (2 x 2 mL) to give a colorless powder and a brown filtrate. The filtrate was stored in a freezer and the powder was washed out into a new vial using toluene (2 mL) to give a yellow solution. The toluene was removed under reduced pressure to give yellow crystals. Hexane was repeatedly added (3 × 2 mL) and subsequently removed. The obtained powder was then suspended in pentane (3 mL) and left in a freezer (–40°C) for 30 min. The supernatant was decanted and put aside while the solid was thoroughly dried to give the first crop of the product as a colorless powder (338.2 mg).

Further crops can be obtained by repeated fractioned crystallization from the pentane filtrates and supernatants in a freezer (–40 °C) The crystals are again isolated by decanting and washing with cool pentane (2-3 mL, –40°C). The pentane supernatants can be collected and used for further crystal harvests. After thoroughly drying of the crystals under reduced pressure further combined crops of the product **ALi_2×(tol)** were received (176.5 mg) to give an overall combined yield of **ALi_2×(tol)** as a colorless powder (514.7 mg, 0.8212 mmol, 61 %.). The amount of toluene per **ALi_2** varies depending on drying effort.

Note: Traces of toluene greatly increase the solubility of the product. Multiple steps of suspending the product in hexane and subsequently removing it are carried out to remove toluene through co-evaporation.

Analytical Data for Compound ALi_2×(tol)

NMR:

^{1}H (400.10 MHz, 296 K, , C_6D_6, , C_6D_5H at 7.15 ppm): 7.12-6.99 (m, toluene, Ar-*H*), 6.97 (s, 1H, B-Xy: *p-H*), 6.70 (s, 2H, *p-H*), 6.50 (s, 4H, *o-H*), 2.53 (s, 6H, B-Xy: Ar-CH_3), 2.21 (s, 12H, Ar-CH_3), 2.10 (s, toluene-CH_3), 0.06 (s, 18H, TMS). B-Xy: *o-H*: superimposed by solvent signal.

^{13}C{^{1}H} (100.62 MHz, 298 K, , C_6D_6, solvent signal at 128.0 ppm): 143.8 or 134.5 (*ipso*-C_{ar}), 135.8 (*m*-C_{ar}), 133.6 (B-Xy: *m*-C_{ar}), 129.5 (*o*-C_{ar}), 128.8 (B-Xy: *ipso*-C_{ar}), 128.5 (C_β), ca. 128.0 (B-Xy: *o*-C_{ar}, superimposed by solvent signal), 125.8 (B-Xy: *p*-C_{ar}), 125.7 (*p*-C_{ar}), 104.6 (C_α), 22.1 (B-Xy: *m*-CH_3), 21.5 (*m*-CH_3), 4.7 (TMS).

^{7}Li (155.49 MHz, 296 K, C_6D_6): –8.9.

^{11}B (80.25 MHz, 298 K, C_6D_6): 35.4 ($\omega_{1/2}$ = 423 Hz).

^{29}Si-DEPT (49.69 MHz, 298 K, C_6D_6): –13.1

Elemental Analysis: ($C_{34}H_{45}BLi_2Si_2$ x 0.43 C_7H_8 by NMR) calcd C 78.09, H 8.52, B 1.79, Li 2.3, Si 9.3, observed: C 78.27, H 8.81.

Molecular structure of Compound ALi_2×(tol)

For further details on the diffraction measurement please see the respective section.

ALi_2×(2 tol) crystallized from solutions in hexane in a freezer (–40°C).

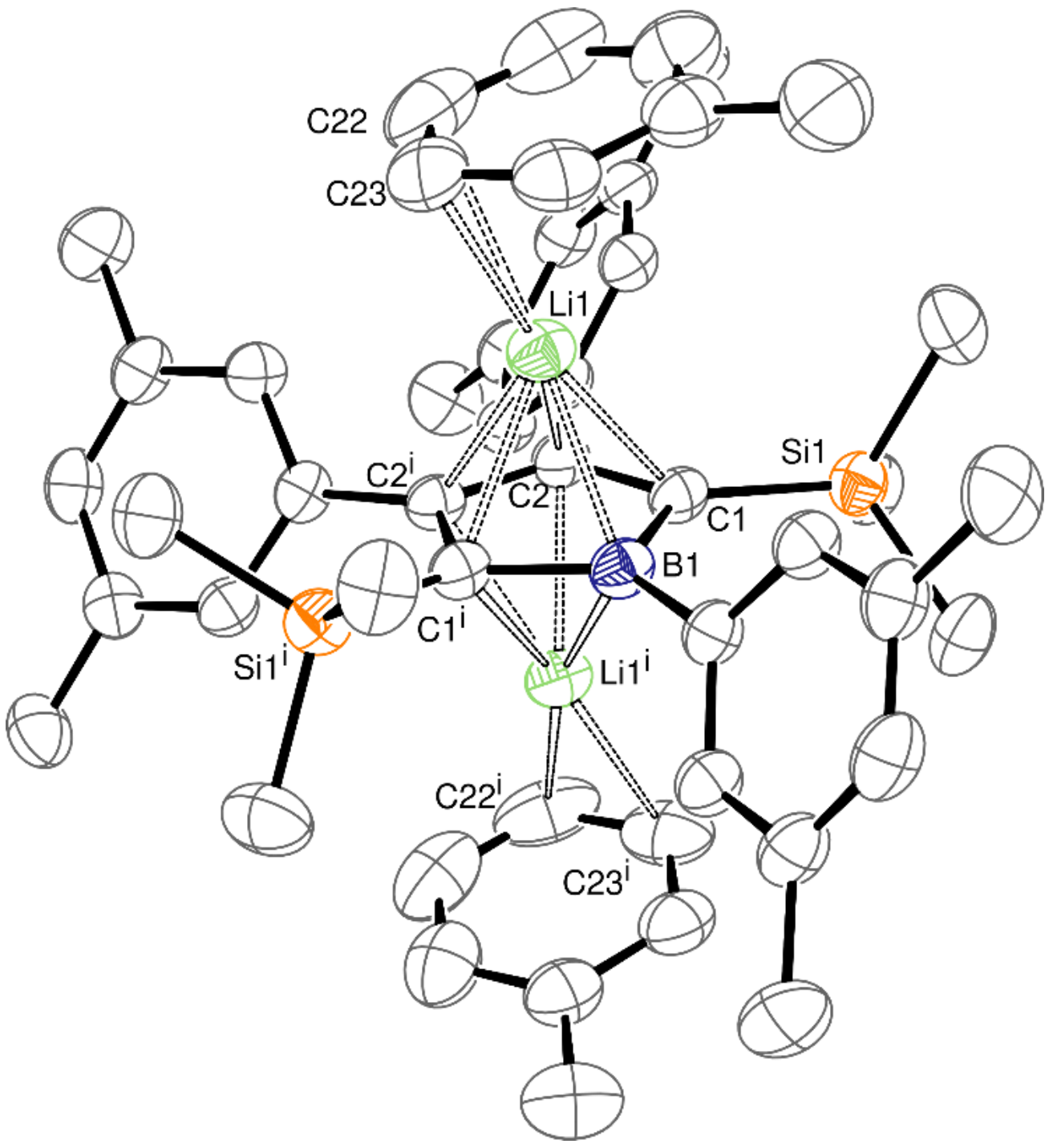

Figure SI1: ORTEP plot of the molecular structure of **ALi_2×(tol)**. Atomic displacement parameters are drawn at 50% probability level. A disordered lattice toluene molecule and hydrogen atoms are omitted for the sake of clarity. Selected bond length in Å: B1-C1 1.554(5), C1-C2 1.462(4), C2-C2^i 1.434(4), B1-Li1 2.212(4), C1-Li1 2.169(5), C2-Li1 2.136(6), Li1-C22 2.508(6), Li1-C23 2.411(6), other Li1–C_{tol} distances: 2.703(6), 2.863(7), 3.073(6) and 3.109(6). The structure was deposited with the CCSD.

Spectra Plots for Compound ALi₂×(tol)

^{1}H-NMR-spectrum of compound **ALi$_2$ x (tol)** in C_6D_6

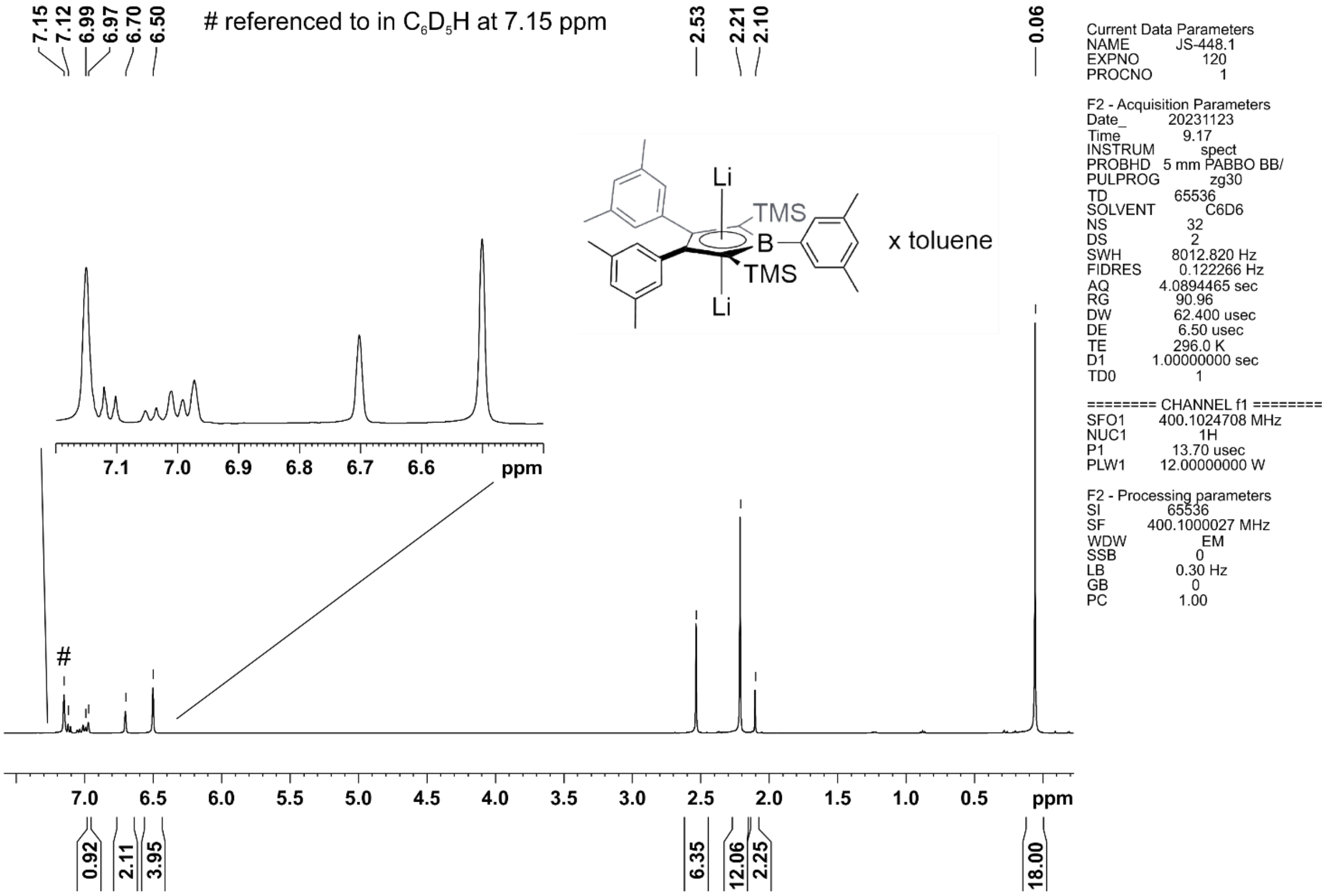

^{13}C{^{1}H}-NMR-spectrum of compound **ALi$_2$ x (tol)** in C_6D_6

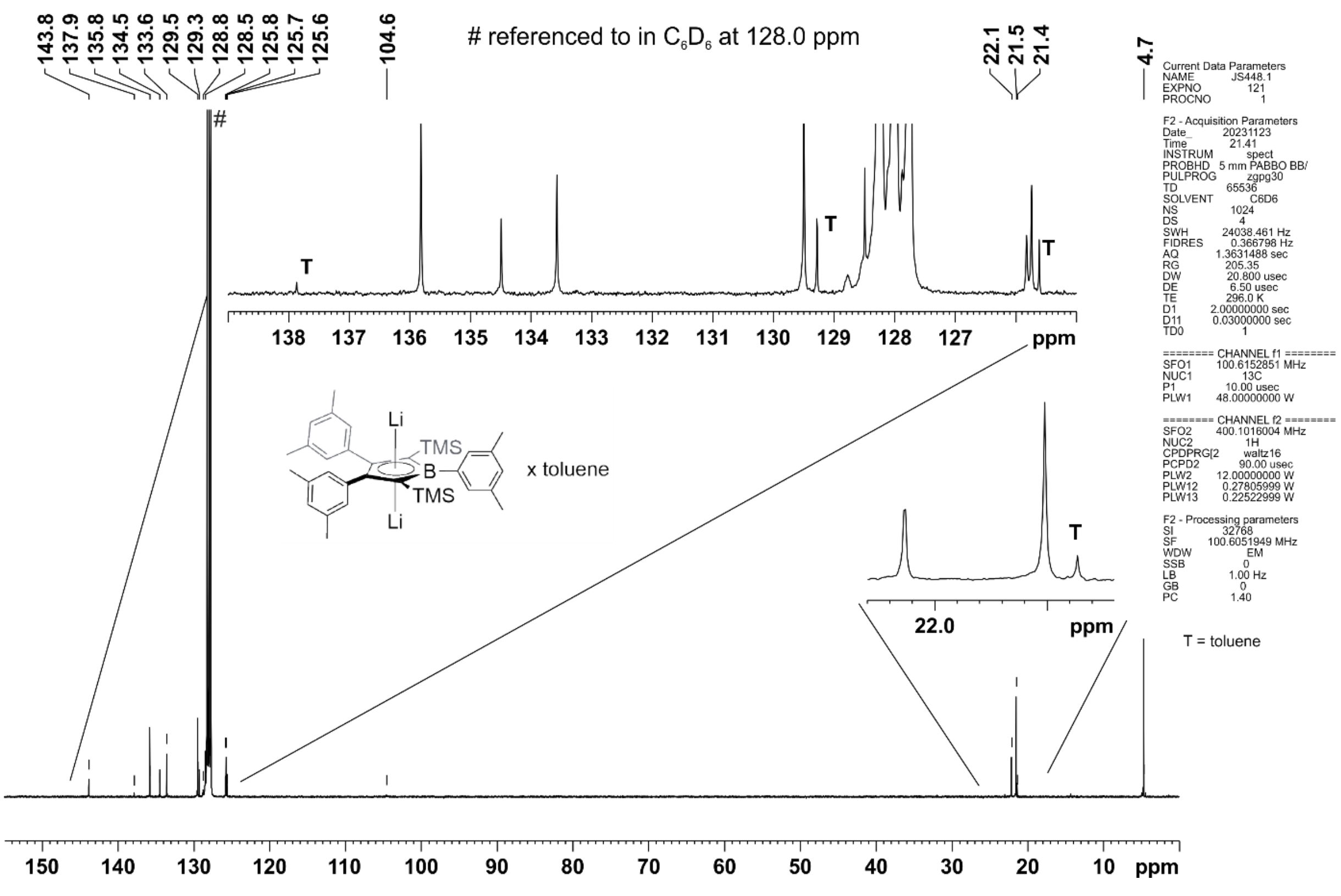

^{7}Li-NMR-spectrum of compound **ALi$_2$ x (tol)** in C_6D_6

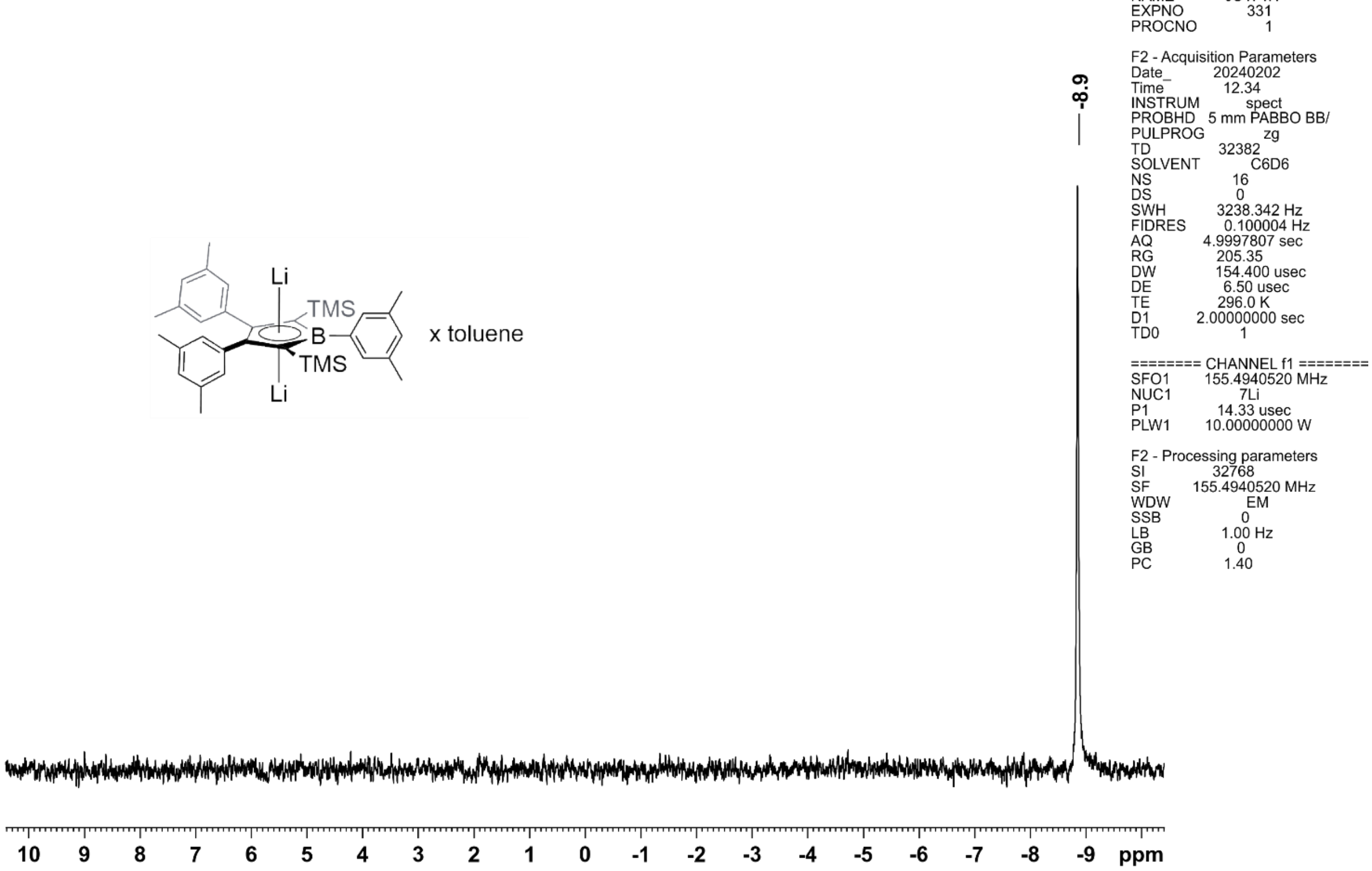

^{11}B-NMR-(background surpressed)-spectrum of compound **ALi$_2$ x (tol)** in C_6D_6

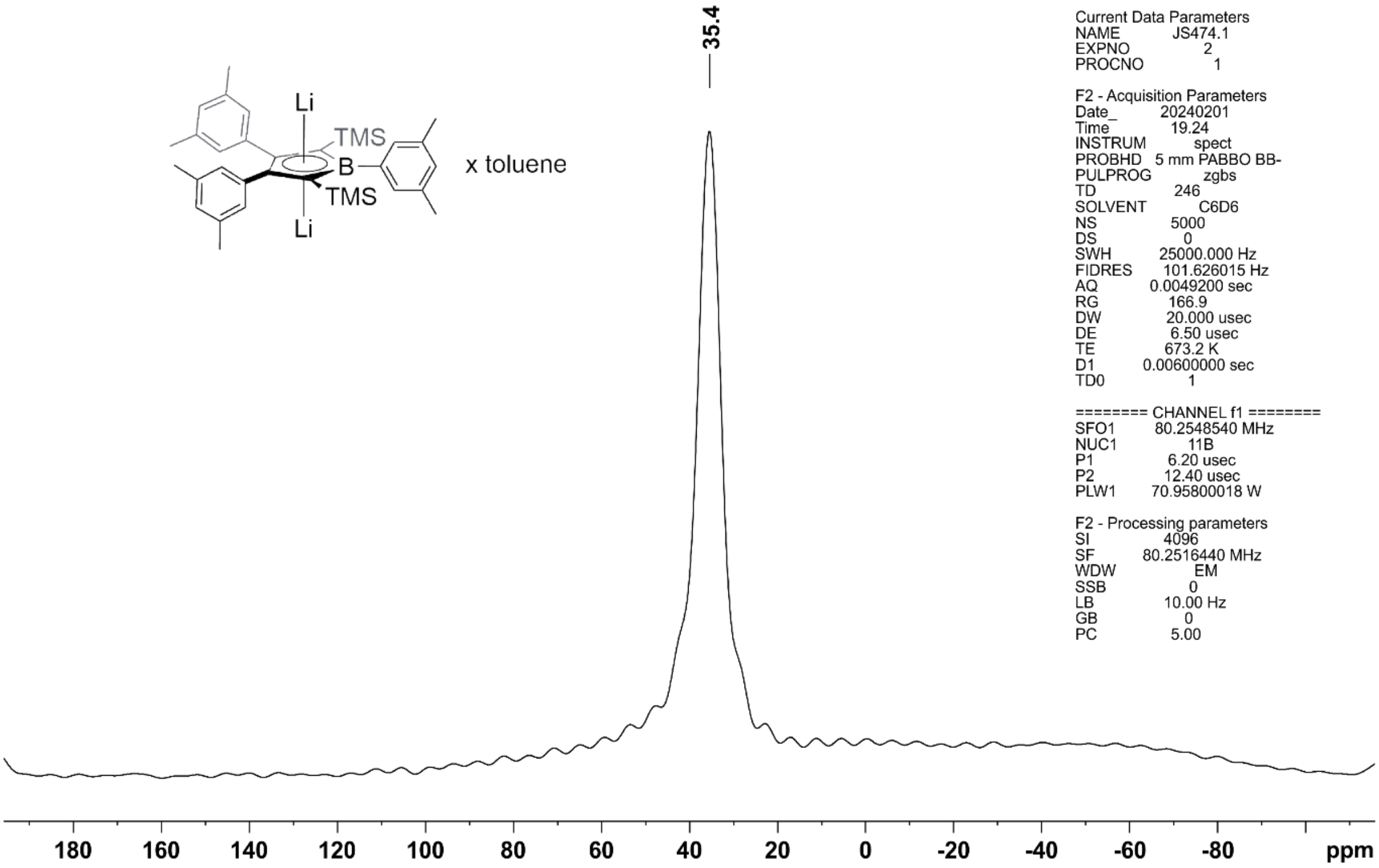

^{29}Si-DEPT-NMR-spectrum of compound **ALi$_2$ x (tol)** in C_6D_6

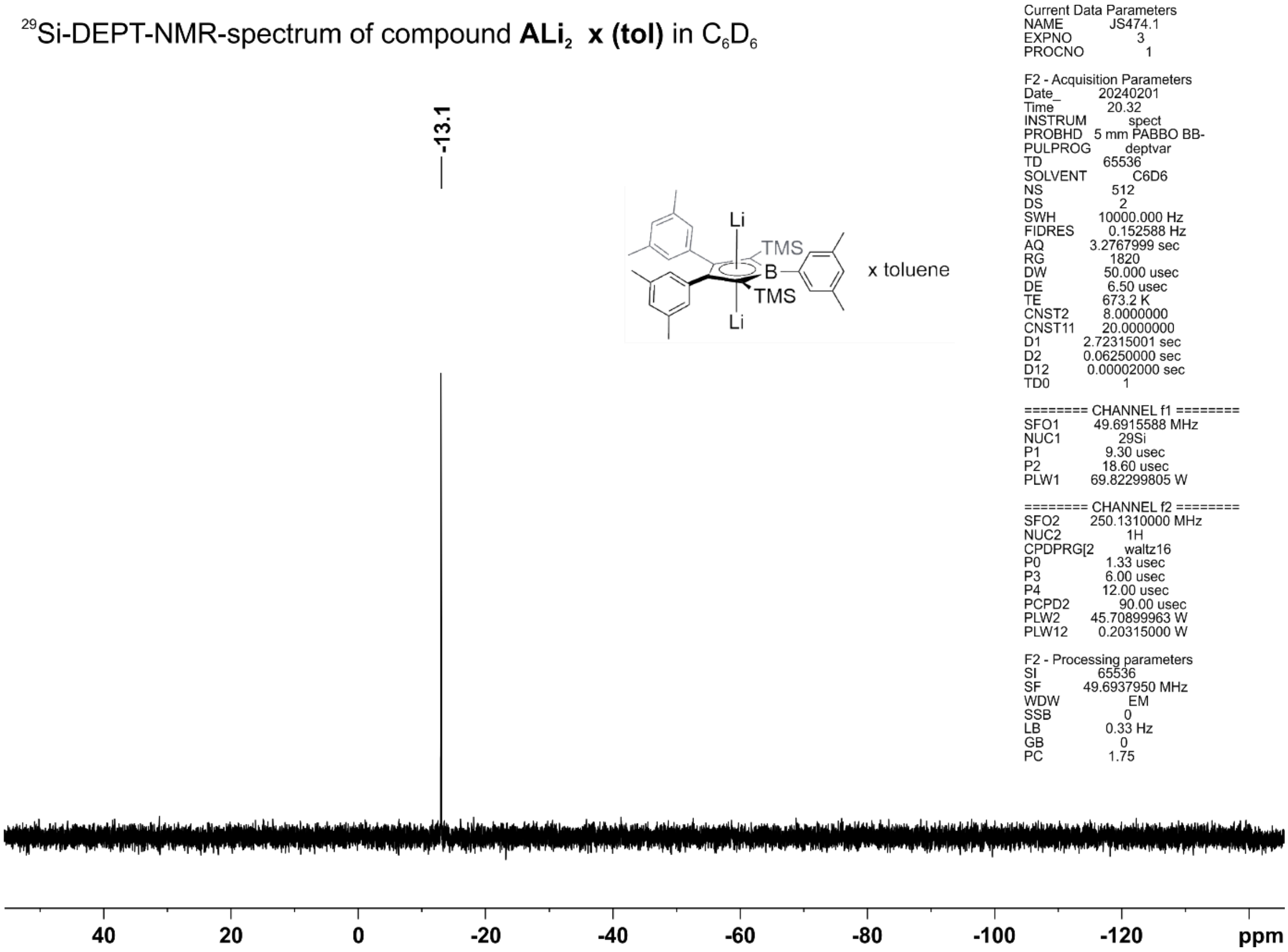

Compound 1

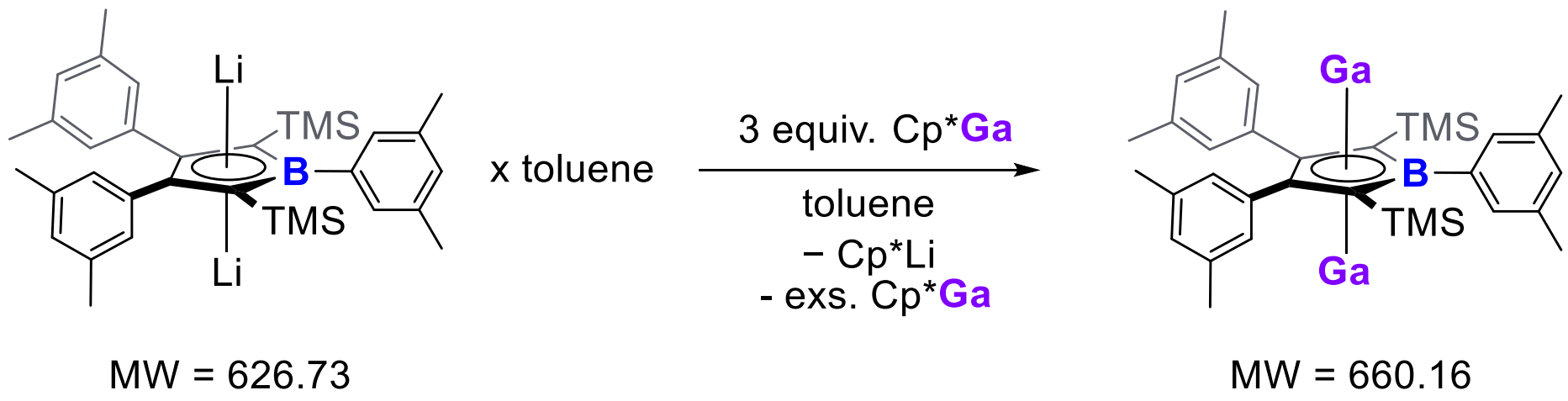

In a glovebox, a solution of pentamethylcyclopentadienyl gallium Cp*Ga (315.5 mg, 1.539 mmol, 3 eq.) in toluene (2 mL) was added to a solution of compound **ALi_2×(tol)$_1$**(321.8 mg, 0.5135 mmol, 1 eq.) in toluene (3 mL). Additional toluene (6 mL) was added and the slowly resulting suspension was stirred for three days at ambient temperature. The colorless suspension was filtered using a syringe filter equipped with a glass fiber pad (Whatman GF/B) to give a nearly colorless filtrate and a colorless residue. The solvent of the filtrate was removed under reduced pressure to give a colorless powder. The residue in the filter was again suspended in toluene (5 mL), filtered and the filtrate was combined with the first crop of the previously obtained powder. The solvent was again removed under reduced pressure and the solid residue was thoroughly dried *in vacuo* to give *closo*-1,7-digalla-tetracarborane **1** as a colorless powder (283.1 mg, 0.4288 mmol, 84 %).

Note: The vast amounts of toluene used in this procedure as well as the repeated evaporation step have been deliberately chosen to ensure the removal of excessive Cp*Ga from the reaction mixture. Application of excessive Cp*Ga is necessary for a complete conversion to the digallium cluster.

Despite numerous attempts, we have not been able to obtain single crystalline samples of **1.**

Analytical Data for Compound 1

NMR:

^{1}H (250.13 MHz, 298 K, , C_6D_6, ppm , C_6D_5H at 7.15 ppm): 7.67 (s, 2H, B-Xy: *o-H*), 7.39 (s, 4H, *o-H*), 6.95 (s, 1H, B-Xy: *p-H*), 6.58 (s, 2H, *p-H*), 2.36 (s, 6H, B-Xy: Ar-CH_3), 2.01 (s, 12H, Ar-CH_3), 0.01 (s, 18H, TMS).

^{13}C{^{1}H} (100.62 MHz, 298 K, ppm, C_6D_6, solvent signal at 128.0 ppm): 145.5 (C_β), 138.4 (*ipso*-C_{ar}), 137.0 (*m*-C_{ar}), 136.1 (B-Xy: *m*-C_{ar}), 132.9 (B-Xy: *o*-C_{ar}), 132.5 (C_α, only visible in 2D spectra), 130.4 (*o*-C_{ar}), 128.8 (*p*-C_{ar}), 21.7 (B-Xy: *m*-CH_3), 21.1 (*m*-CH_3), 2.5 (TMS). B-Xy: *ipso*-C_{ar} not found. Superimposed by solvent signal: B-Xy: *p*-C_{ar}.

^{11}B (80.25 MHz, ppm, 298 K, C_6D_6): 31.5 ($\omega_{1/2}$ = 423 Hz).

^{29}Si-DEPT (49.69 MHz, ppm, 298 K, C_6D_6): –8.4.

^{71}Ga: (91.51 MHz, 298 K – 323 K, ppm, C_6D_6): no signal detected (screened from +300 to – 700 ppm)

Elemental Analysis: ($C_{34}H_{45}BGa_2Si_2$) calcd C 61.86, H 6.87, B 1.64, Ga 21.12, Si 8.51, observed C 61.70, H 7.07.

LIFDI-MS: calcd exact mass: 660.1660 m/z, observed m/z: 660.1645 $[M]^+$.

Spectra Plots for Compound 1

^{1}H-NMR-spectrum of compound **1** in C_6D_6

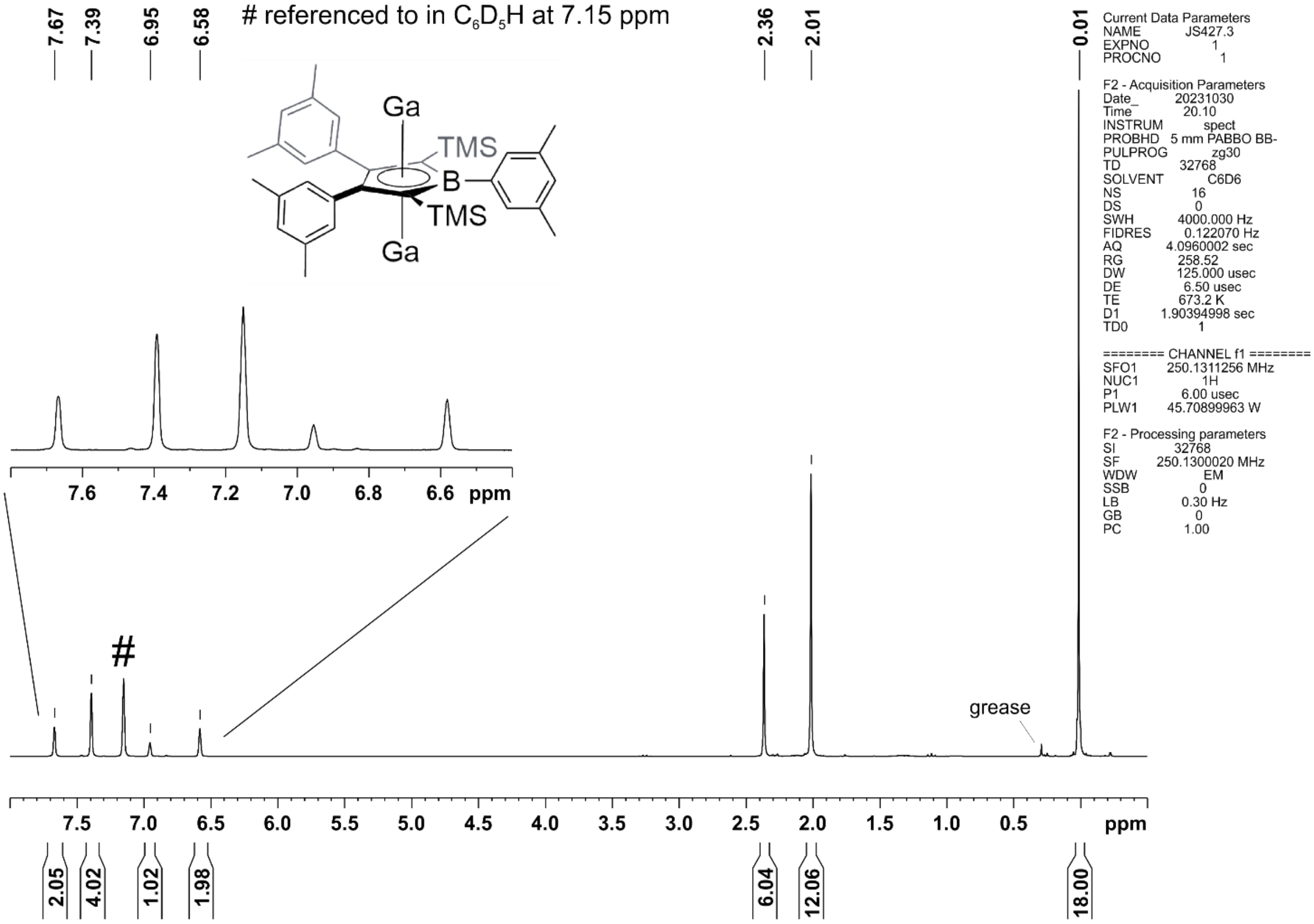

^{13}C{^{1}H}-NMR-spectrum of compound **1** in C_6D_6

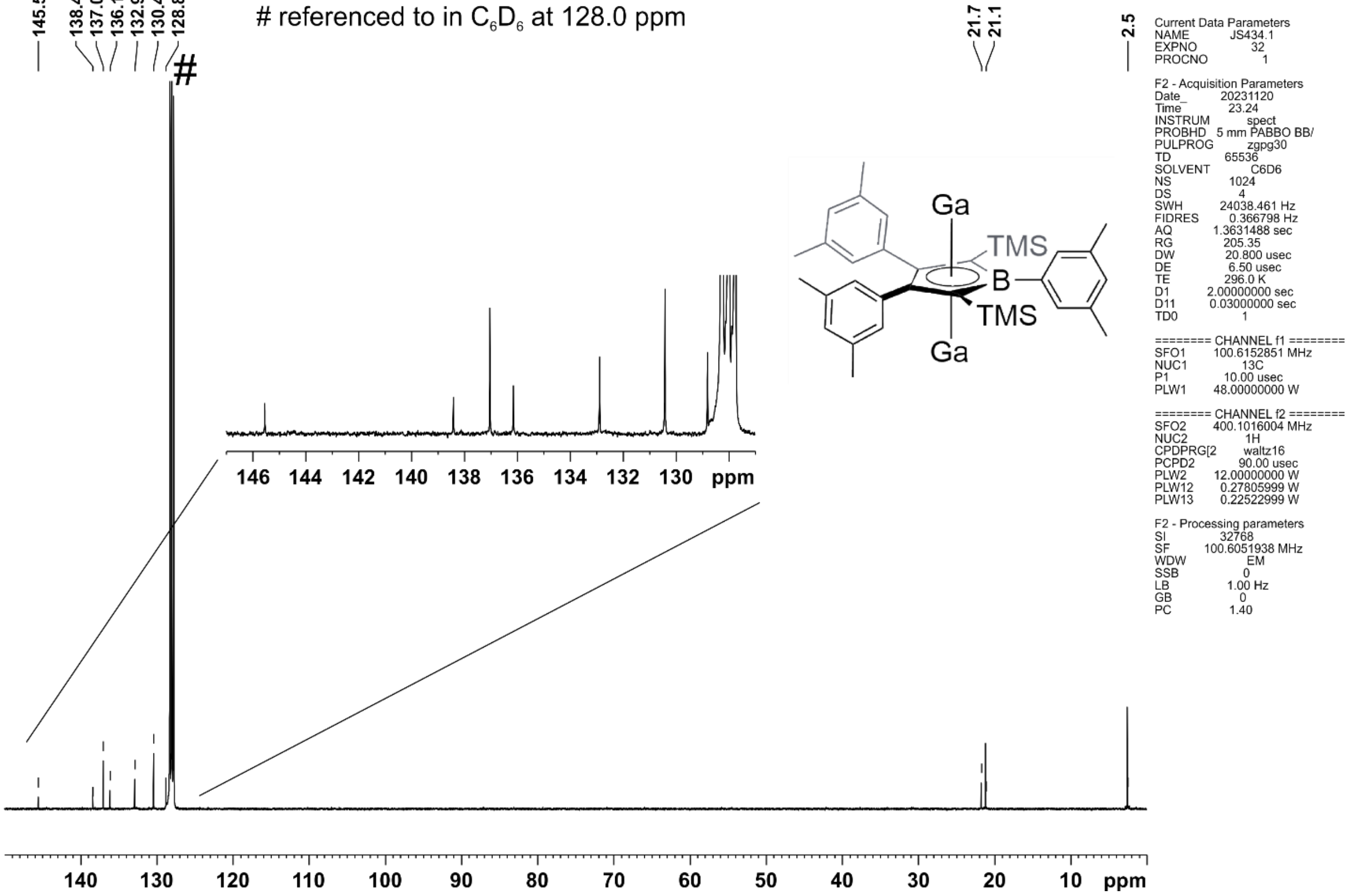

^{11}B-NMR-(background surpressed)-spectrum of compound **1** in C_6D_6

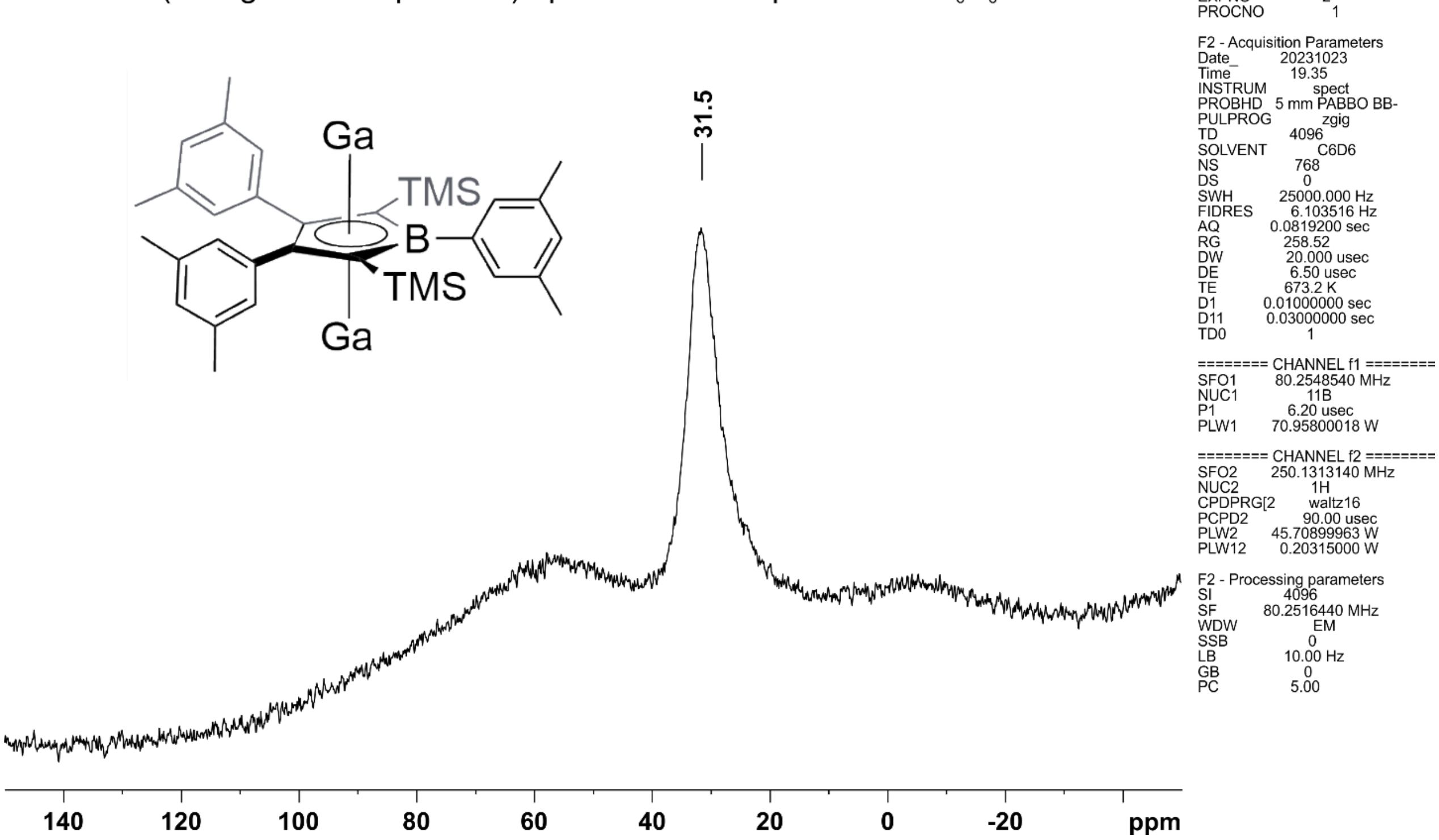

^{29}Si-DEPT-NMR-spectrum of compound **1** in C_6D_6

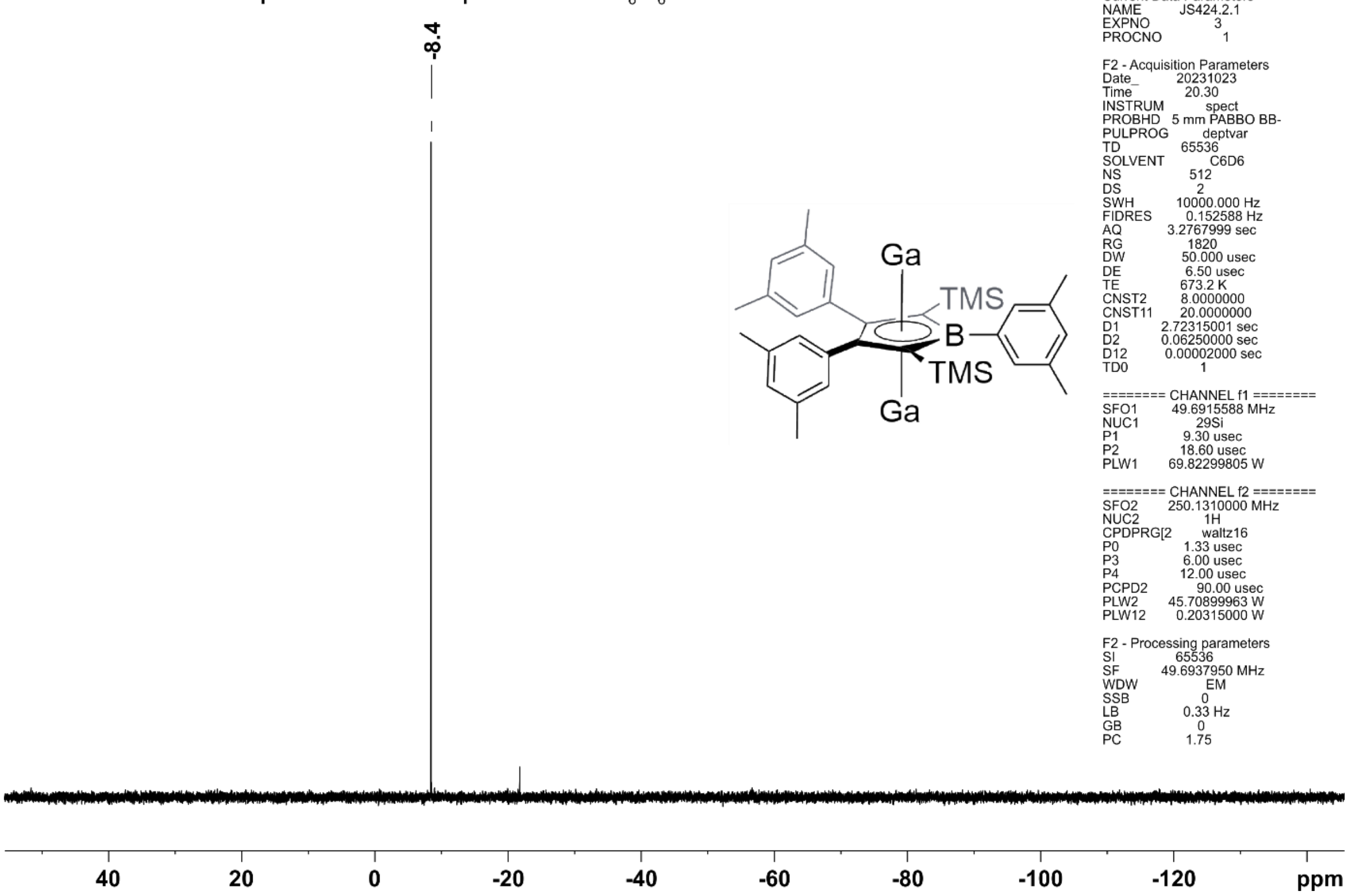

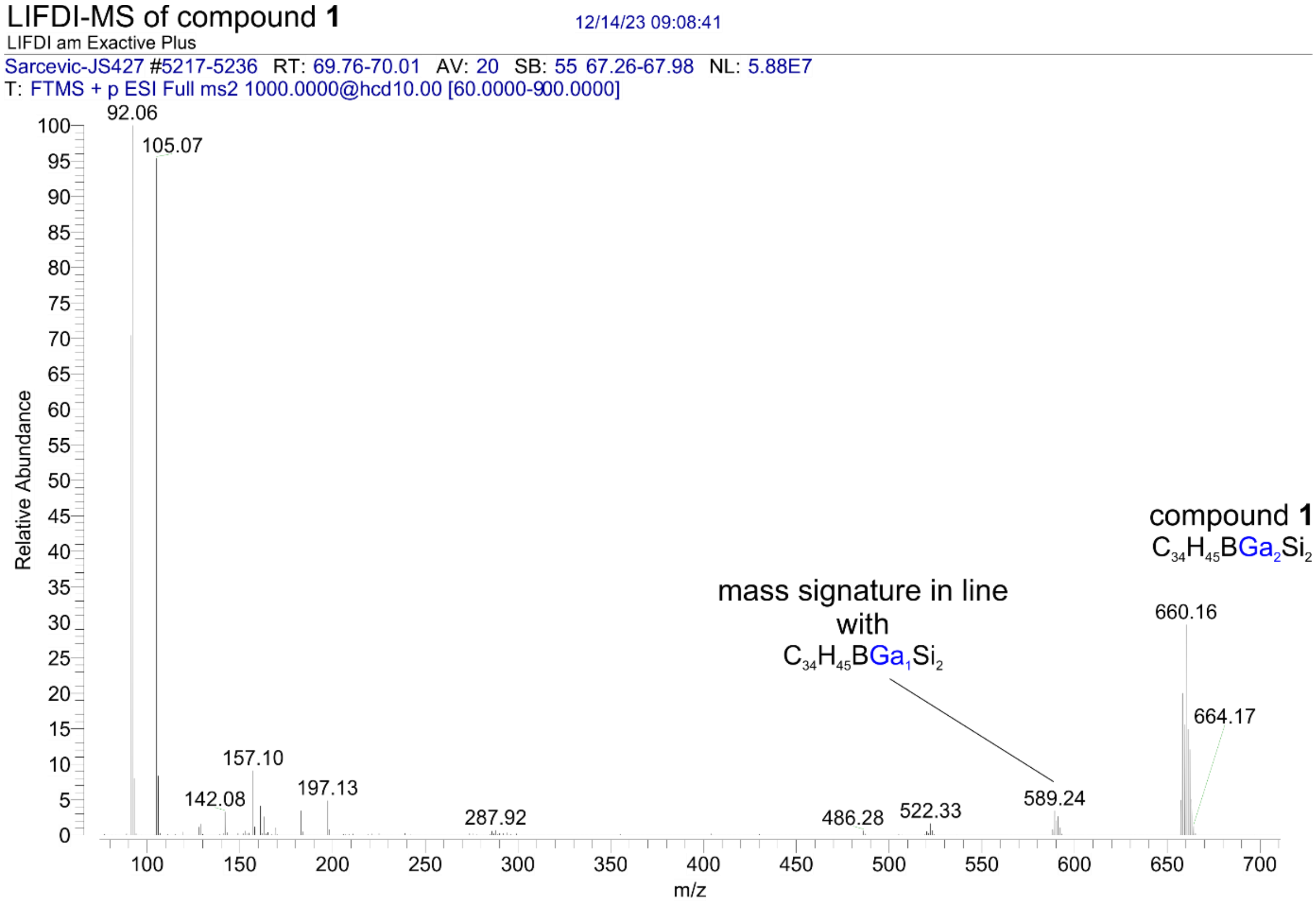
LIFDI-MS of compound 1
12/14/23 09:08:41
LIFDI am Exactive Plus
Sarcevic-JS427 #5217-5236 RT: 69.76-70.01 AV: 20 SB: 55 67.26-67.98 NL: 5.88E7
T: FTMS + p ESI Full ms2 1000.0000@hcd10.00 [60.0000-900.0000]
Relative Abundance
m/z
92.06
105.07
142.08
157.10
197.13
287.92
486.28
522.33
589.24
660.16
664.17
mass signature in line with
C34H45BGa1Si2
compound 1
C34H45BGa2Si2

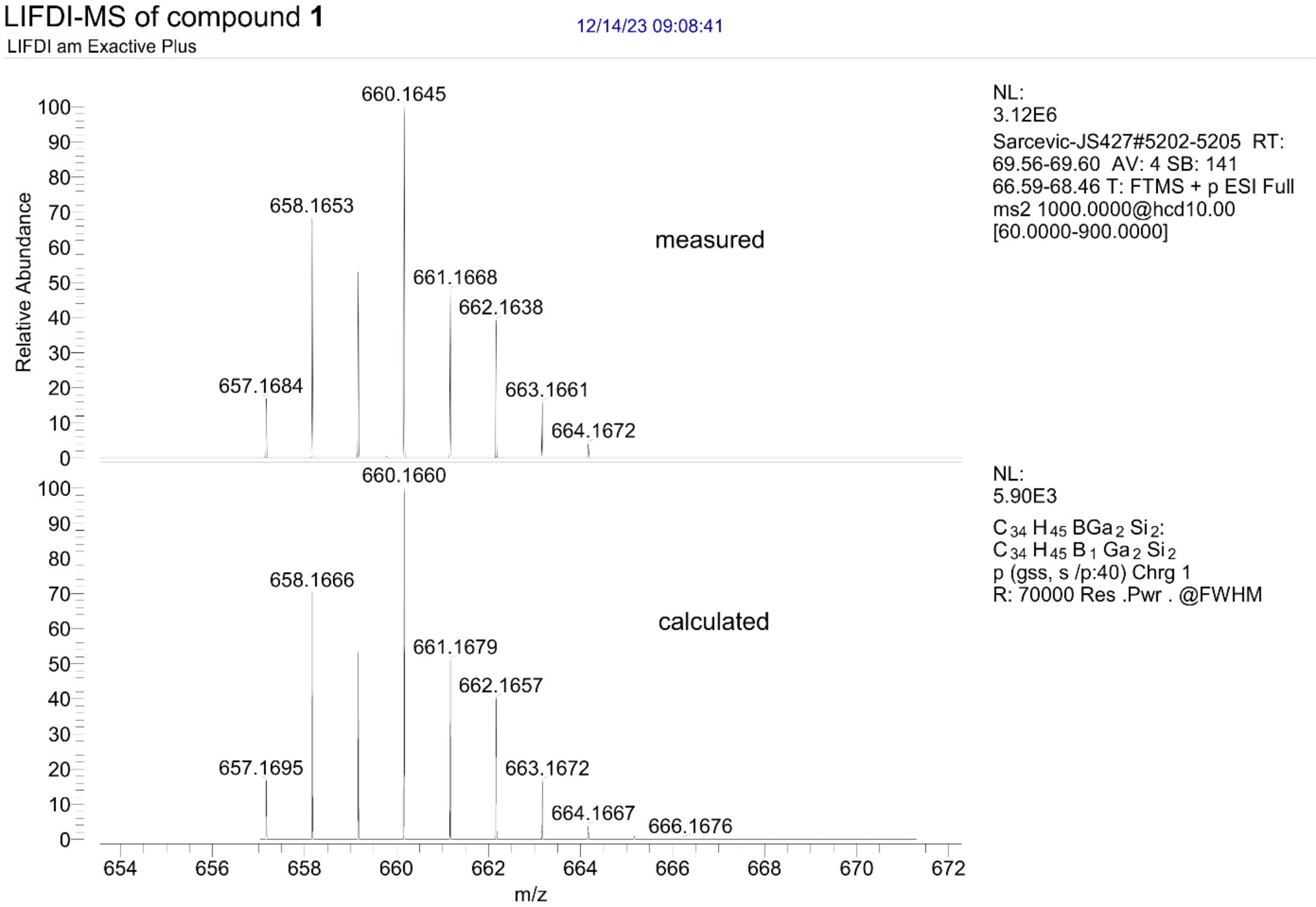
LIFDI-MS of compound 1
12/14/23 09:08:41
LIFDI am Exactive Plus
Relative Abundance
m/z
measured
657.1684
658.1653
660.1645
661.1668
662.1638
663.1661
664.1672
NL: 3.12E6
Sarcevic-JS427#5202-5205 RT: 69.56-69.60 AV: 4 SB: 141 66.59-68.46 T: FTMS + p ESI Full ms2 1000.0000@hcd10.00 [60.0000-900.0000]
calculated
657.1695
658.1666
660.1660
661.1679
662.1657
663.1672
664.1667
666.1676
NL: 5.90E3
C 34 H 45 BGa 2 Si 2:
C 34 H 45 B 1 Ga 2 Si 2
p (gss, s /p:40) Chrg 1
R: 70000 Res .Pwr . @FWHM

Compound 2

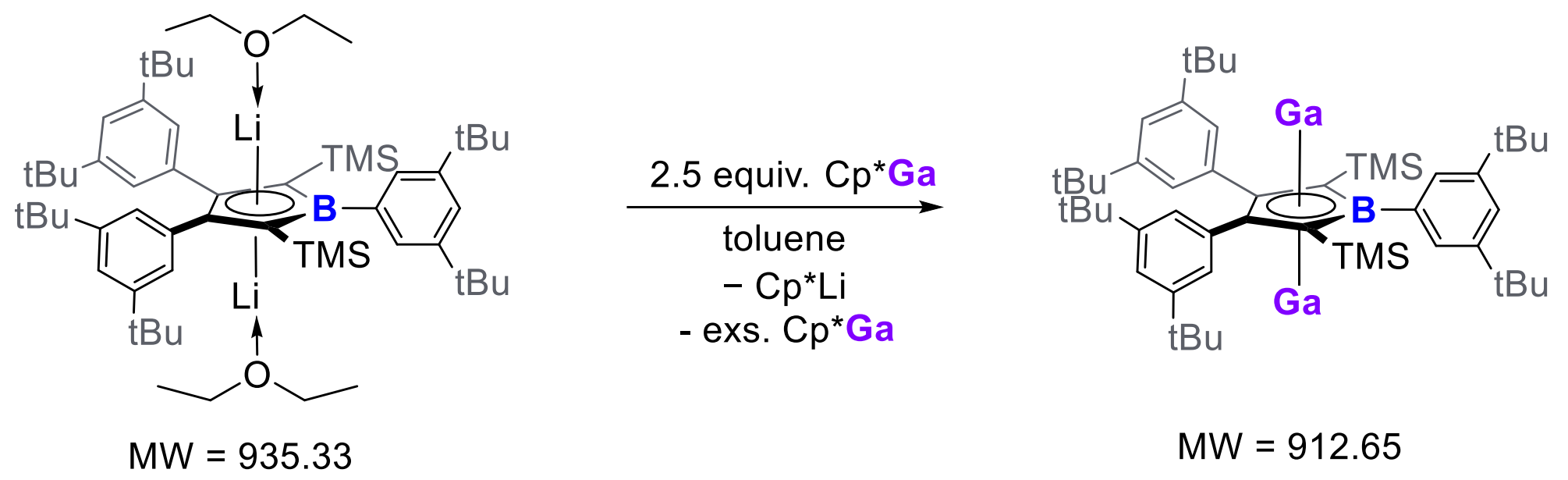

In a glovebox, borole diide **$BLi_2(OEt_2)_2$** (240.5 mg, 0.257 mmol, 1 eq) was dissolved in toluene (8 mL) and a solution of Cp*Ga (105.4 mg, 0.514 mmol, 2 eq) in toluene (2 mL) was added. The yellow solution was transferred out of the glovebox and heated to 60 °C in an oil bath. After eleven days, a pale-yellow turbid solution was obtained. The solvent of the reaction mixture was removed under reduced pressure and transferred back into the glovebox. The obtained solid was dissolved in pentane (3 mL), filtered through a syringe equipped with a pad of glass fiber (Whatman GF/B) and the residue in the filter was washed with pentane (3 x 1 mL). The solvent of the combined filtrates was removed under reduced pressure and the obtained pale-yellow solid was analyzed by NMR-Spectroscopy. The NMR analysis showed full consumption of borole diide **$BLi_2(OEt_2)_2$** and the formation of the desired digallium compound **2** along with and an unknown compound (presumably the mixed Li-Ga-compound) in ca. 87 % and 13 %, respectively. Therefore, the crude product was redissolved in toluene (10 mL) and Cp*Ga (26.3 mg, 0.128 mmol, 0.5 eq) was added. The reaction mixture was heated to 60 °C for seven days and the solvent of the reaction mixture was once again removed under reduced pressure. The remaining solid was extracted with *n*-hexane (4 × 1 mL), filtered through a syringe equipped with a pad of glass fiber (Whatman GF/B) and the solvent of the filtrates was removed under reduced pressure. The obtained solid was cooled to –40 °C and carefully washed with cold *n*-hexane (–40 °C, 2 × 0.3 ml, 2 × 0.2 mL) and the wash phases were decanted with a syringe. After drying the remaining solid under reduced pressure, compound *closo*-1,7-digalla-tetracarborane **2** (73.5 mg, 0.081 mmol, 31 %) was obtained as a white solid. A second crop of product was obtained by removing the solvent of the combined wash phases, redissolving the solid in toluene (0.9 mL) and storing the solution at –40 °C. This way, colorless crystals of compound *closo*-1,7-digalla-tetracarborane **2** (0.052 mg, 0.058 mml, 22 %) were grown.

Analytical Data for Compound 2

NMR:

^{1}H (400.30 MHz, 299 K, C_6D_6, CD_5H at 7.15 ppm): 7.92 (d, $^4J_{HH}$ = 1.9 Hz, 2H, *o-$H_{Ar\text{-}B}$*), 7.57 (t, $^4J_{HH}$ = 1.9 Hz, 1H, *p-$H_{Ar\text{-}B}$*), 7.52 (d, $^4J_{HH}$ = 1.8 Hz, 4H, *o-$H_{Ar\text{-}3,4}$*), 7.29 (t, $^4J_{HH}$ = 1.8 Hz, 2H, *p-$H_{Ar\text{-}3,4}$*), 1.51 (s, 18H, Ar_B-C(*Me*)$_3$), 1.24 (s, 36H, $Ar_{3,4}$-C(*Me*)$_3$), 0.05 (s, 18H, Si(*Me*)$_3$).

^{13}C{^{1}H} (100.67 MHz, 299 K, C_6D_6, solvent signal at 128.0 ppm): 150.1 (*m-$C_{Ar\text{-}3,4}$*), 149. 1(*m-$C_{Ar\text{-}B}$*), 146.3 (*$C_{3,4}$*), 143.6 (br, *ipso-$C_{Ar\text{-}B}$*, detected via HMBC), 137.9 (*ipso-$C_{Ar\text{-}3,4}$*), 132.7 (*$C_{2,5}$*), 129.2 (*o-$C_{Ar\text{-}B}$*), 126.9 (*o-$C_{Ar\text{-}3,4}$*), 120.1 (*p-$C_{Ar\text{-}3,4}$*), 119.8 (*p-$C_{Ar\text{-}B}$*), 35.0 (Ar_B-*C*(CH_3)$_3$), 34.8 ($Ar_{3,4}$-*C*(CH_3)$_3$), 32.0 (Ar_B-C(*C*H_3)$_3$), 31.6 ($Ar_{3,4}$-C(*C*H_3)$_3$), 2.5(Si(*C*H_3)$_3$).

^{11}B (128.43 MHz, 299 K, C_6D_6): 32.4 ($\omega_{1/2} \approx$ 750 Hz).

^{29}Si-DEPT-20 (79.52 MHz, 299 K, C_6D_6): –8.5.

^{71}Ga: (91.51 MHz, 298 K – 323 K, ppm, C_6D_6): no signal detected (screened from +300 to – 700 ppm)

Elemental Analysis: $C_{52}H_{81}BGa_2Si_2$ calcd C 68.44, H 8.95; observed C 68.69, H 9.33.

Molecular structure of Compound 2

For further details on the diffraction measurement please see the respective section.

Crystals suitable for X-ray analysis were grown from saturated *n*-hexane solutions at –40 °C.

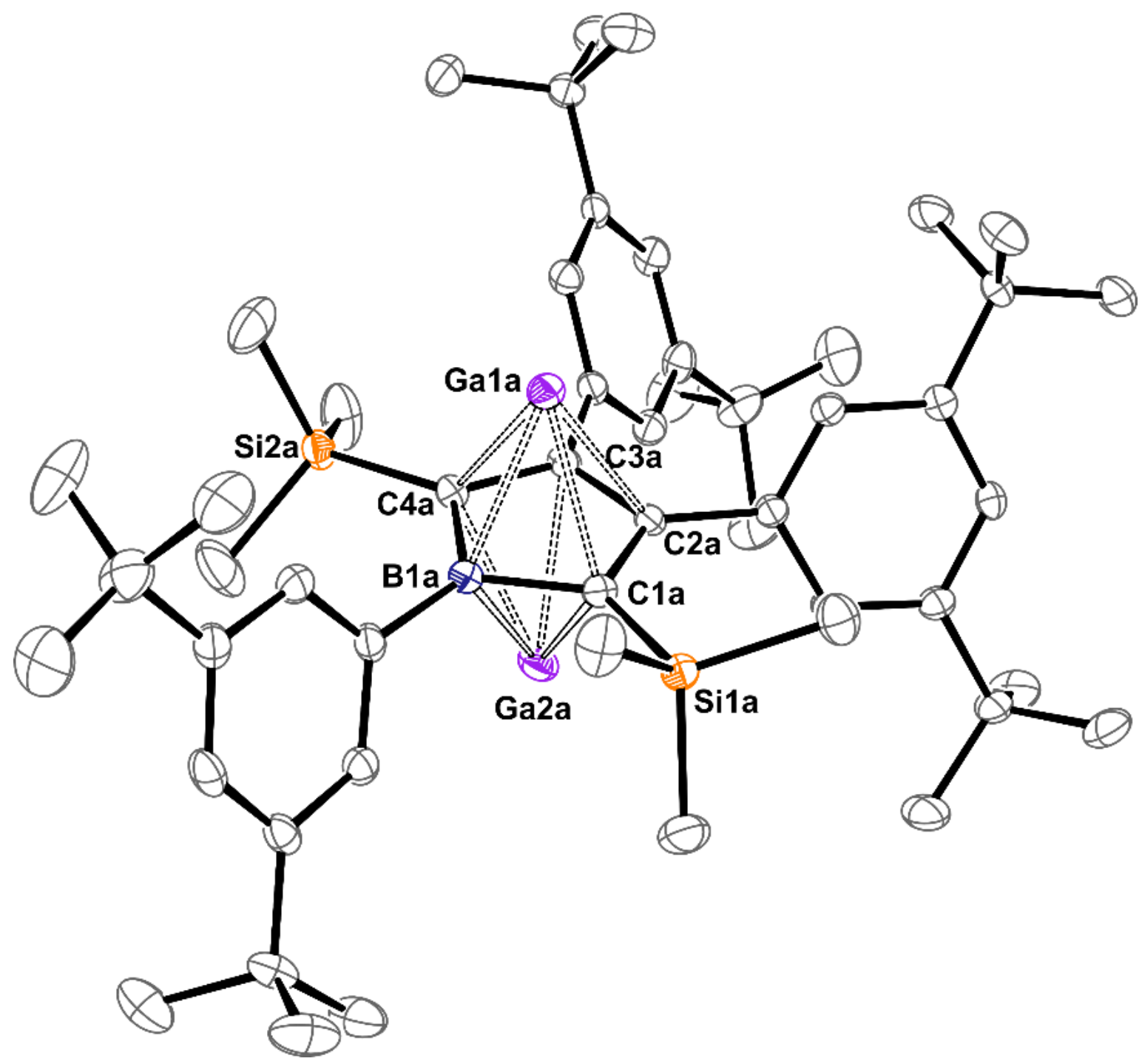

Figure SI2: ORTEP plot of the molecular structure of **2**. Atomic displacement parameters are drawn at 50% probability level. A second molecule in the asymmetric unit, hydrogen atoms as well as minor disorder of organic moieties are not shown for the sake of clarity. Selected bond lengths [Å] and angles [°] are given: B1a–C1a 1.559(3), C1a–C2a 1.455(3), C2a–C3a 1.439(3), C3a–C4a 1.459(3); B1a–C4a 1.551(3), B1a–Ga1a 2.399(3), C1a-Ga1a 2.412(3), C2a–Ga1a 2.438(2), C3a–Ga1a 2.422(2), C4a–Ga1a 2.413(3), B1a–Ga2a 2.410(3), C1a-Ga2a 2.437(3), C2a–Ga2a 2.440(2), C3a–Ga2a 2.460(3), C4a–Ga2a 2.435(3). Ctr_{BC4}–Ga1a 2.056, Ctr_{BC4}–Ga2a 2.079, Ga1a–Ctr_{BC4}–Ga2a 175.97. The structure was deposited with the CCSD.

Spectra Plots for Compound 2

^{1}H-NMR-spectrum of compound **2** in C_6D_6

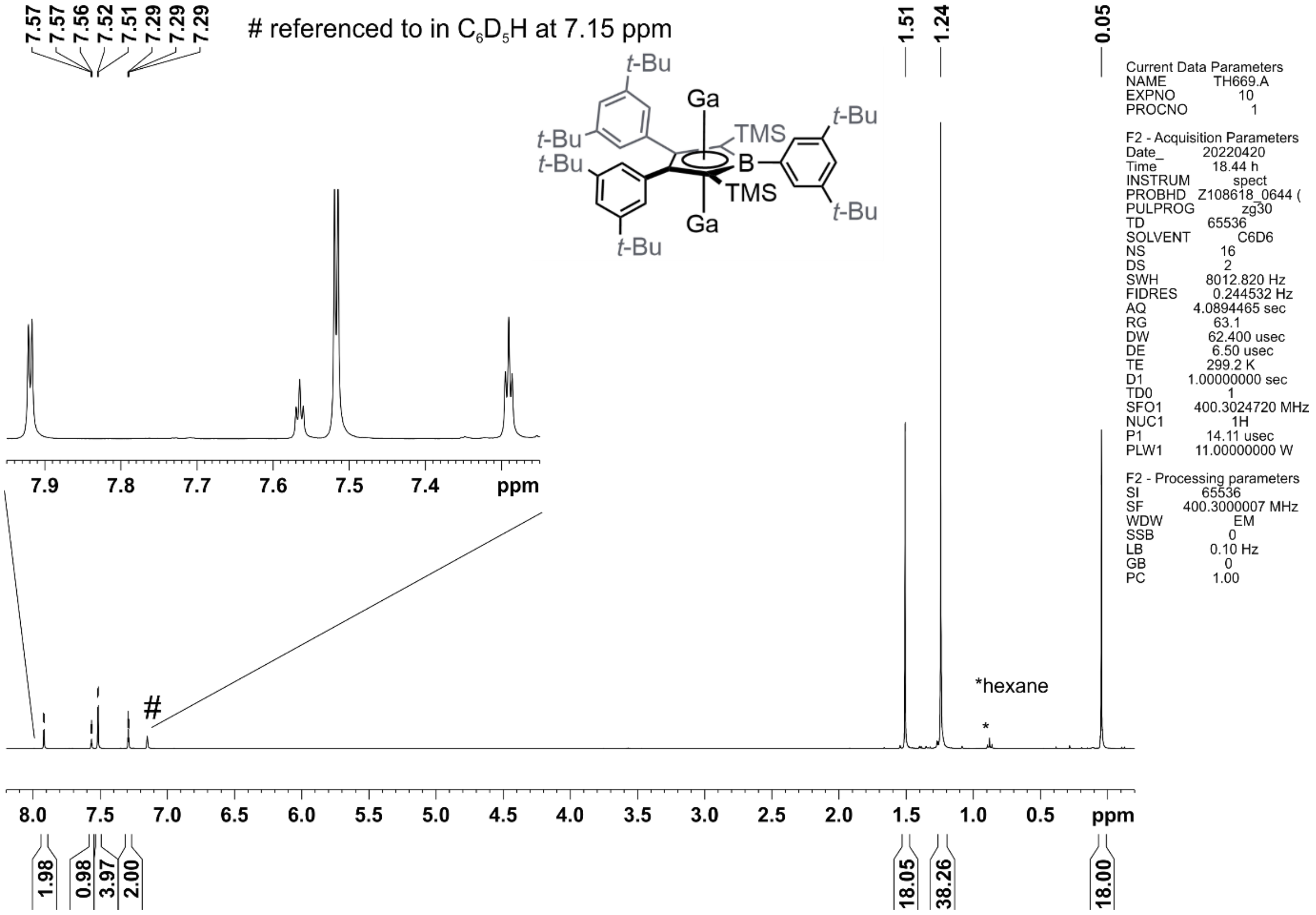

^{13}C{^{1}H}-NMR-spectrum of compound **2** in C_6D_6

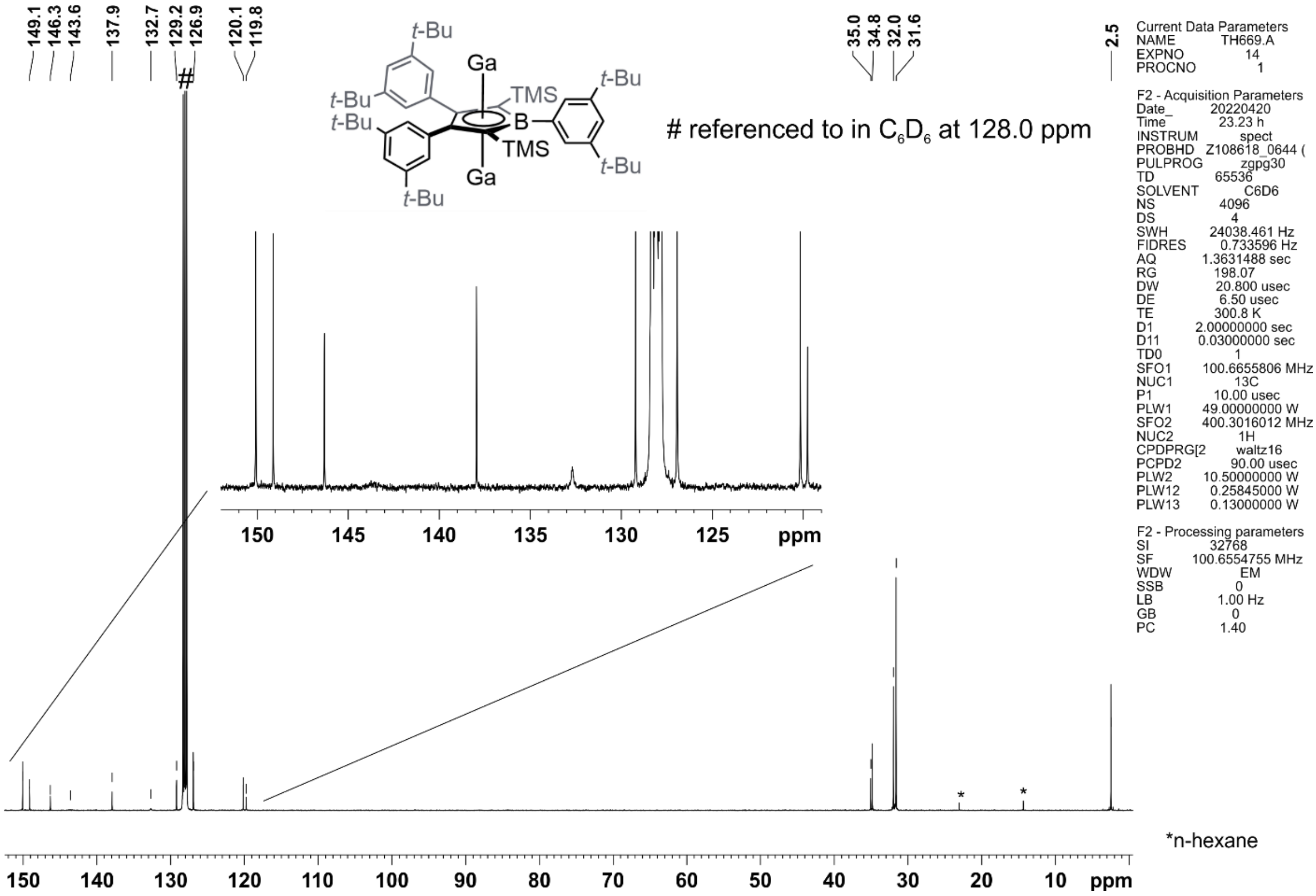

^{11}B-NMR-(background surpressed)-spectrum of compound **2** in C_6D_6

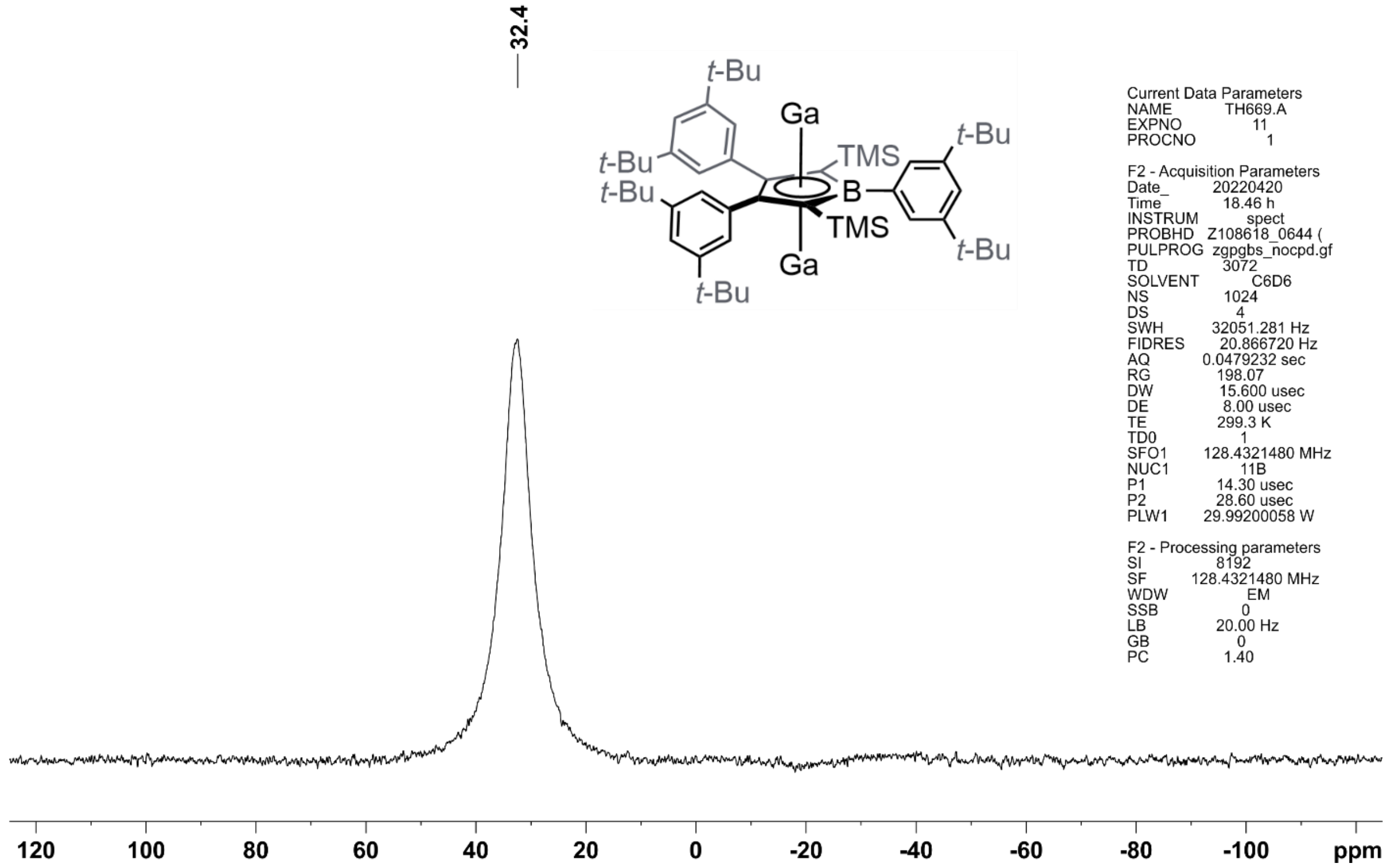

^{29}Si-DEPT-NMR-spectrum of compound **2** in C_6D_6

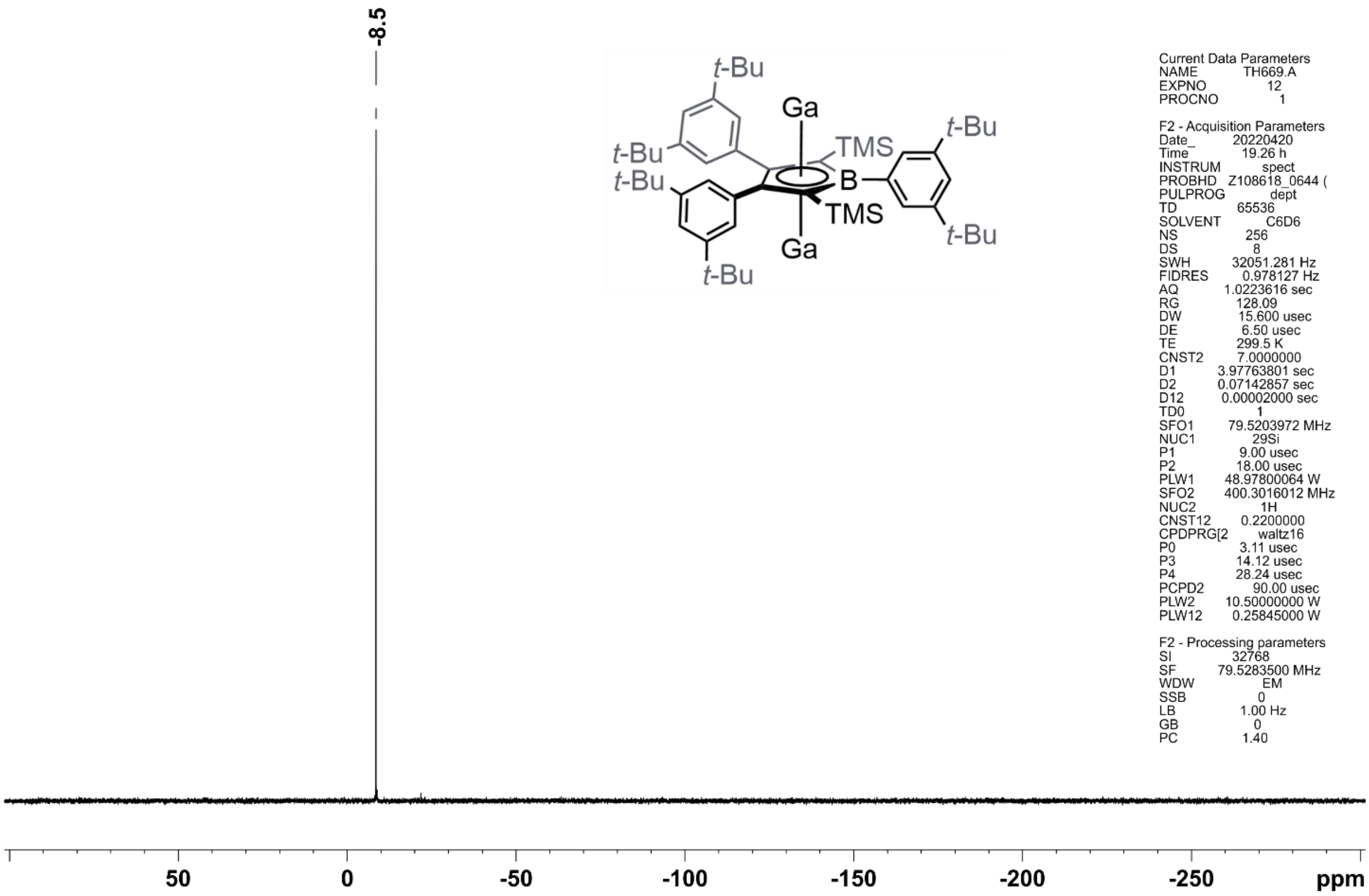

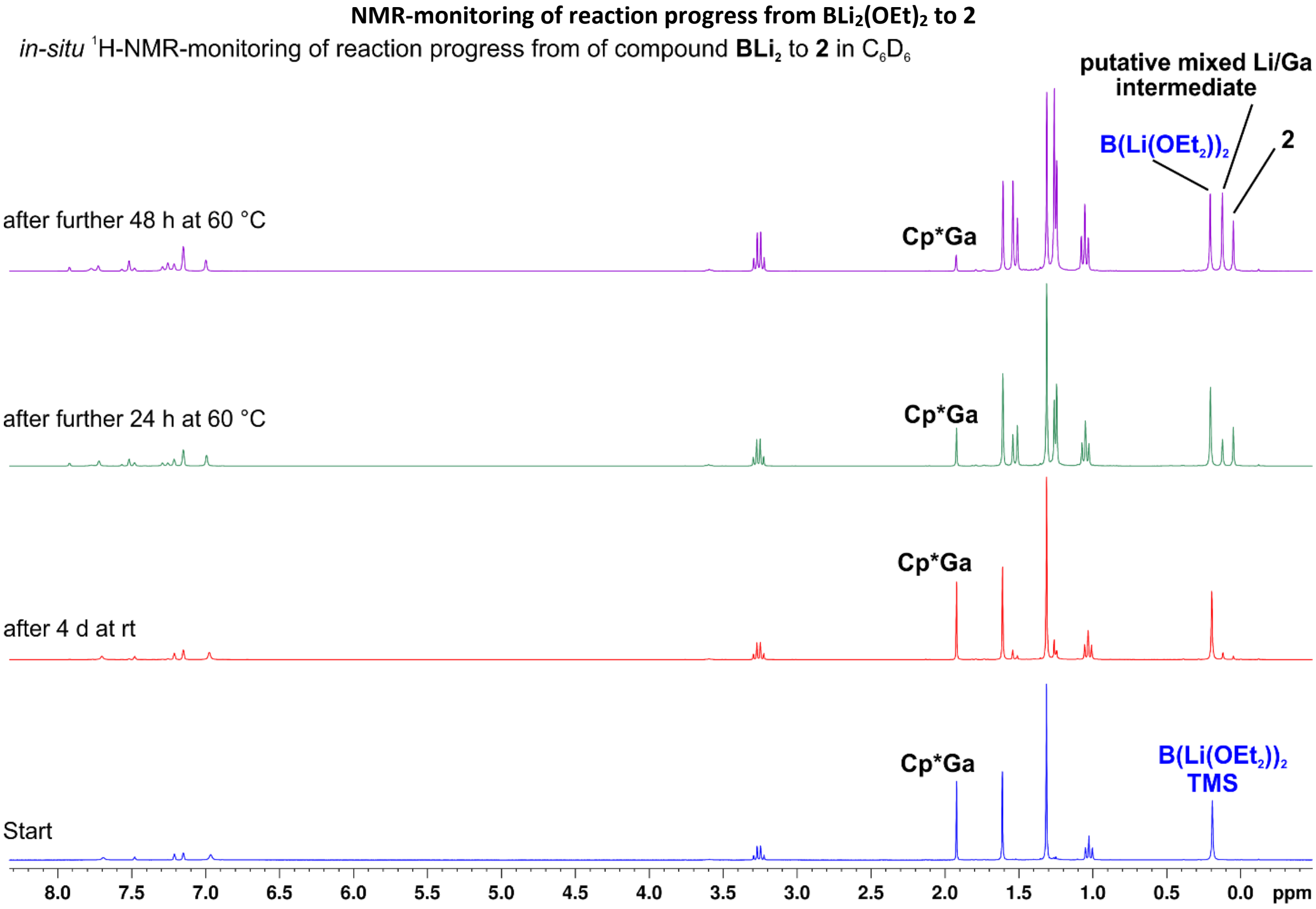

Figure SI3: ^{1}H-NMR-monitoring of the conversion of **$BLi_2(OEt)_2$** to **2**

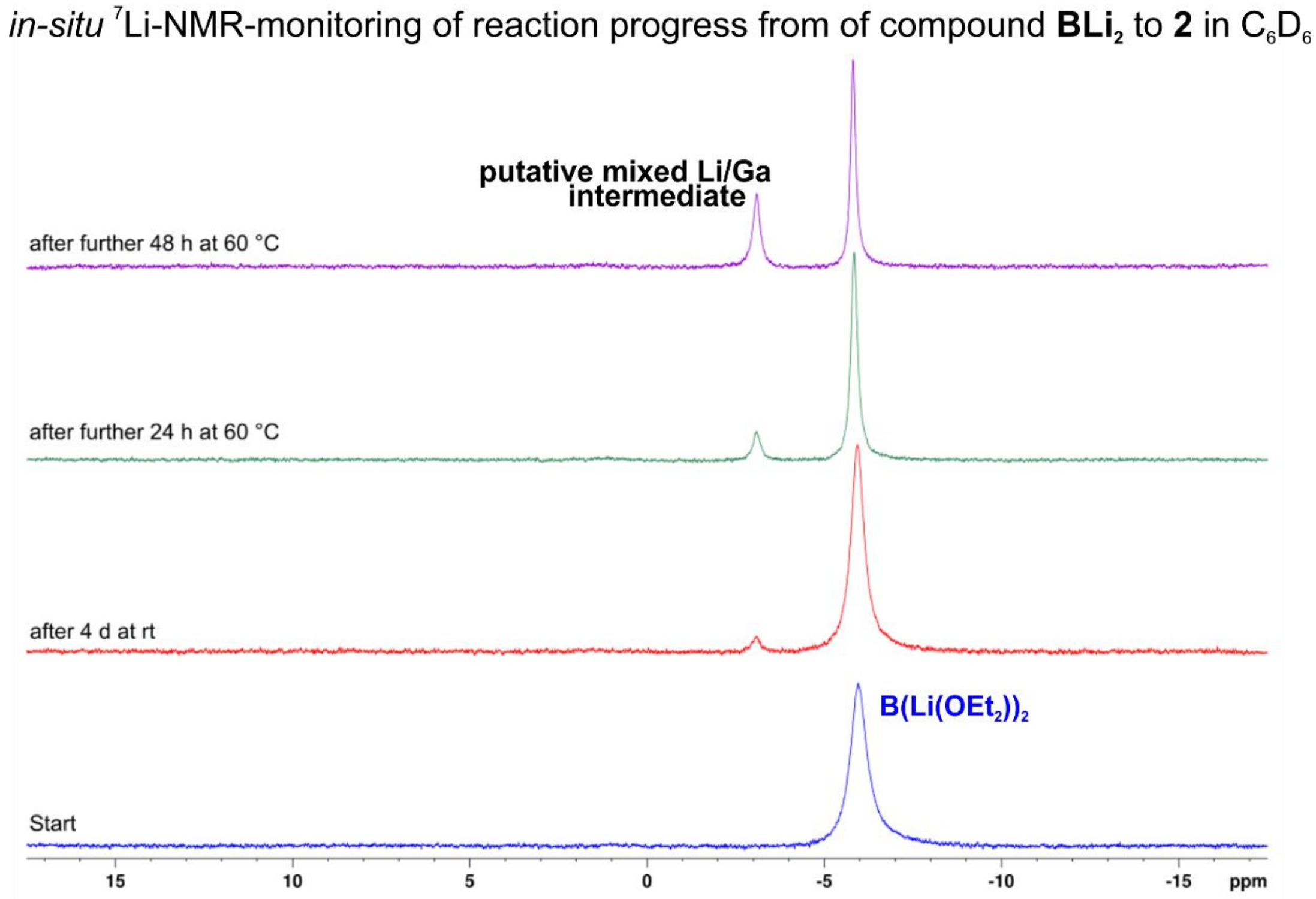

Figure SI4: ^{7}Li-NMR-monitoring of the conversion of **$BLi_2(OEt)_2$** to **2**

Compound 3

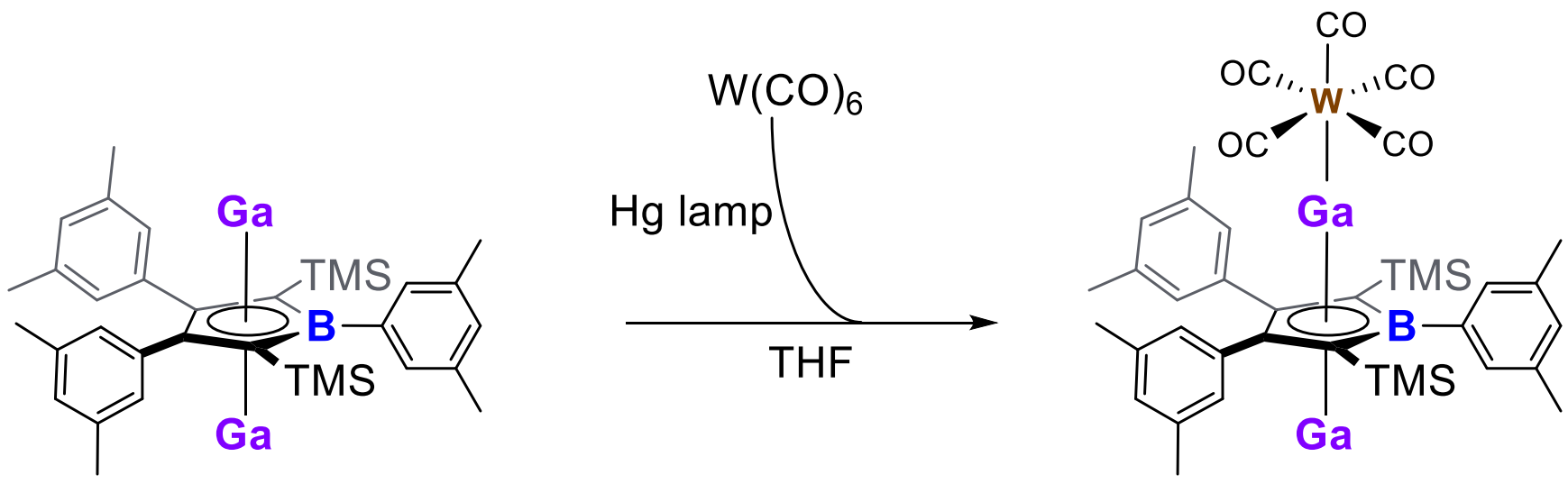

MW = 660.16   MW = 984.05

An ampoule, equipped with a Young-type valve, was charged with a solution of tungsten hexacarbonyl (49.1 mg, 0.139 mmol, 0.9 eq.) in THF (5 mL). The colorless solution was irradiated over the course of 3 h with a mercury-vapor lamp. The now yellow solution was transferred into a glove box and a solution of digallium cluster **1** (103.0 mg, 0.1560 mmol, 1 eq.) in THF (4 mL) was added to the ampoule. The solution was left to sit at ambient temperature for 24 h. The resulting almost colorless solution was transferred into a vial and the THF was removed under reduced pressure. The resulting beige powder was suspended in hexane (2 mL) which was subsequently removed under reduced pressure to remove residual THF traces. The now colorless powder was dissolved in hexane (5 mL) and stored at –40 °C over night to give colorless crystals. The crystals were isolated, suspended in hexane (1 mL) and again stored at –40 °C over night. While still cool, the hexane wash phase was removed by decanting with a syringe and the crystalline solid residue was dried under reduced pressure to give mono-adduct **3** (70.7 mg, 0.0718 mmol, 51 %) in the form of colorless crystals.

Note: Long-term storage of solutions of the product lead to the slow formation of small amounts (ca. 5%) of bis-[$W(CO)_5$]-adduct (**4**) over the course of several days.

Analytical Data for Compound 3

NMR:

^{1}H (400.10 MHz, 296 K, CD_2Cl_2, CD_1Cl_2H at 5.32 ppm): 7.29 (s, 2H, B-Xy: *o-H*), 7.01 (broad s, 4H, *o-H*), 6.99 (superimposed s, 1H, B-Xy: *p-H*), 6.89 (s, 2H, *p-H*), 2.35 (s, 6H, B-Xy: Ar-CH_3), 2.22 (s, 12H, Ar-CH_3), –0.23 (s, 18H, TMS).

^{13}C{^{1}H} (176.12 MHz, 296 K, CD_2Cl_2, solvent signal at 53.84 ppm): 199.0 (O=C_{ax}), 197.5 (O=C_{eq}), 145.4 (C_β), 140.7 (B-Xy: *ipso-*C_{ar}, broad and flat), 137.9 (*m-*C_{ar}), 137.0 (B-Xy: *m-*C_{ar}), 135.7 (*ipso-*C_{ar}), 132.9 (B-Xy: *o-*C_{ar}), 132.3 (C_α), 129.9 (*o-*C_{ar}), 129.5 (*p-*C_{ar}), 128.7 (B-Xy: *p-*C_{ar}), 21.5 (B-Xy: *m-*CH_3), 21.1 (*m-*CH_3), 1.8 (TMS).

^{11}B (80.25 MHz, 298 K, CD_2Cl_2): 31.2 ($\omega_{1/2}$ = 250 Hz).

^{29}Si-DEPT (49.69 MHz, 298 K, CD_2Cl_2): –5.1.

^{71}Ga: no signal detected (screened from +300 to – 700 ppm)

Elemental Analysis: ($C_{39}H_{45}BGa_2Si_2WO_5$) calcd: C 47.60, H 4.61; observed: C 47.36, H 4.62.

LIFDI-MS: calcd exact mass for **3** ($C_{39}H_{45}BGa_2Si_2WO_5$): 984.0924 m/z, observed m/z: 984.0916 [M]$^+$; further minor fragments observed: 1308.02 [**4**]$^+$, 914.7 [M-1Ga+1H]$^+$, 1053.02 [M+H_2Ga]$^+$, 660.16 [**1**], 589.24, 521.32.

IR-ATR: 1882 cm^{-1} (s), 1900 cm^{-1} (vs), 1913 cm^{-1} (vs, O=C_{eq} stretch), 1927 cm^{-1} (s), 1977 cm^{-1} (vw, O=C_{ax} stretch), 2060 cm^{-1} (s, all-symm. stretch).

Molecular structure of Compound 3

For further details on the diffraction measurement please see the respective section.

Crystals suitable for X-ray analysis were grown from saturated *n*-hexane solutions at –40 °C.

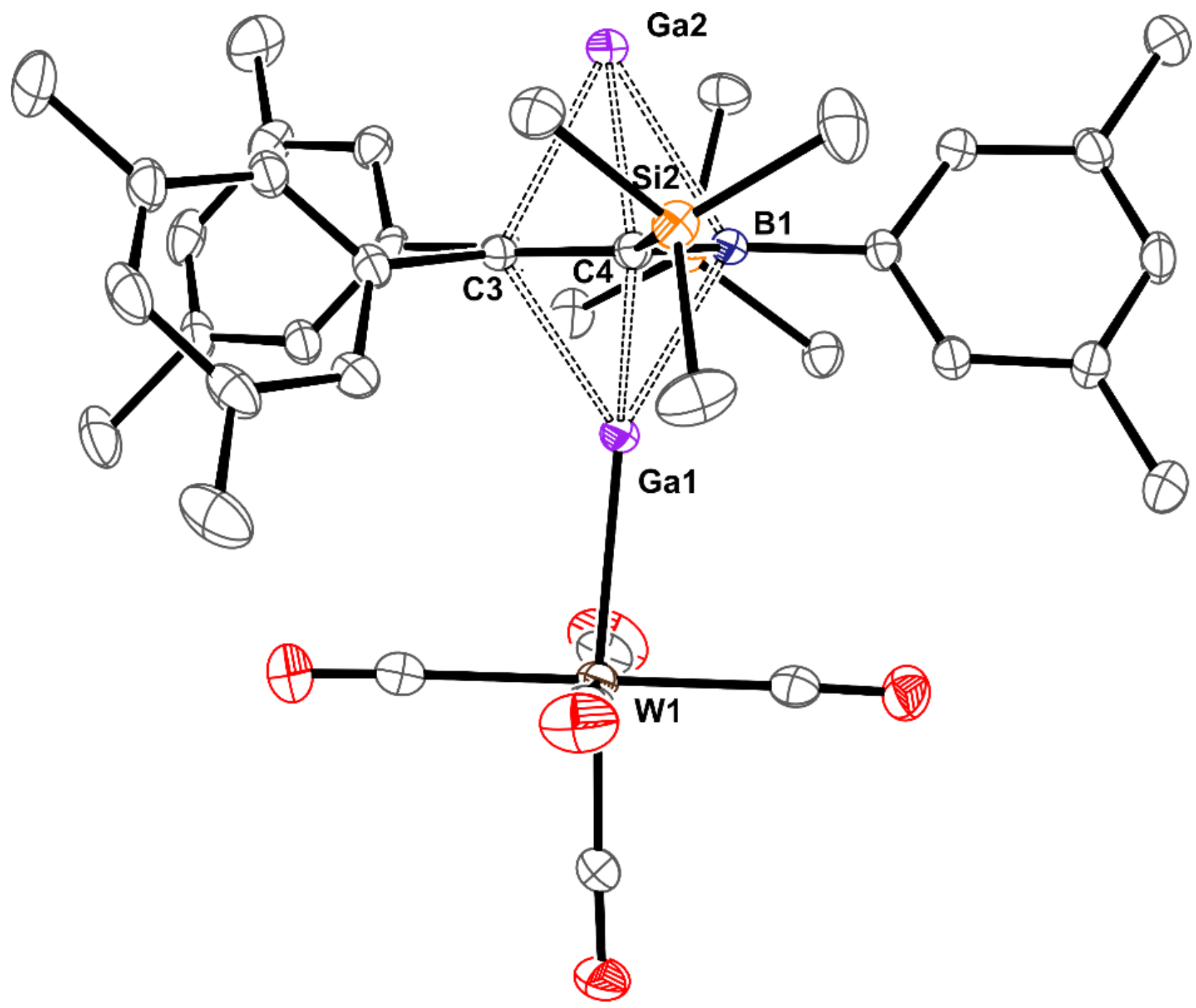

Figure SI5: ORTEP plot of the molecular structure of **3**. Atomic displacement parameters are drawn at 50% probability level. For the sake of clarity, hydrogen atoms are not depicted. Selected bond lengths [Å] and angles [°] are given: B1–Ga2 2.403(2), C1–Ga2 2.444(2), C4–Ga2 2.435(2), C2–Ga2 2.473(2), C3–Ga2 2.446(2), B1–Ga1 2.247(2), C1–Ga1 2.261(2), C4–Ga1 2.291(2), C2–Ga1 2.371(2), C3–Ga1 2.358(2), Ga1–W1 2.5533(5), W1–C_{ax} 1.994(2), W1–C_{eq} 2.038(2)-2.048(2), Ctr_{BC4}–Ga1 1.919, Ctr_{BC4}–Ga2 2.078, Ga1-Ctr_{BC4}-Ga2 172.4°, W1-Ga1-Ga2 173.06(1)°. The structure was deposited with the CCSD.

Spectra Plots for Compound 3

^{1}H-NMR-spectrum of compound **3** in CD_2Cl_2

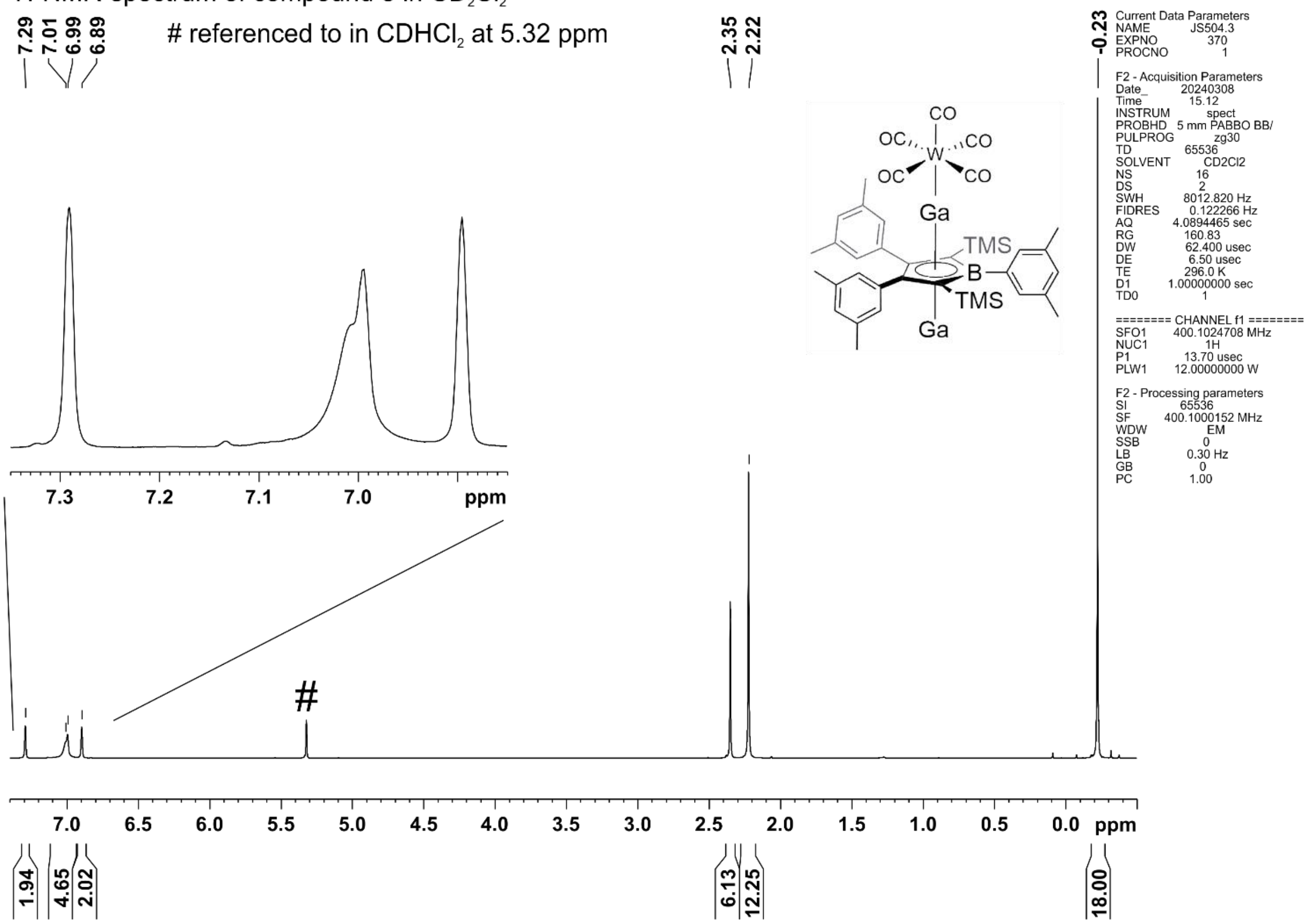

^{13}C{^{1}H}-NMR-spectrum of compound **3** in CD_2Cl_2

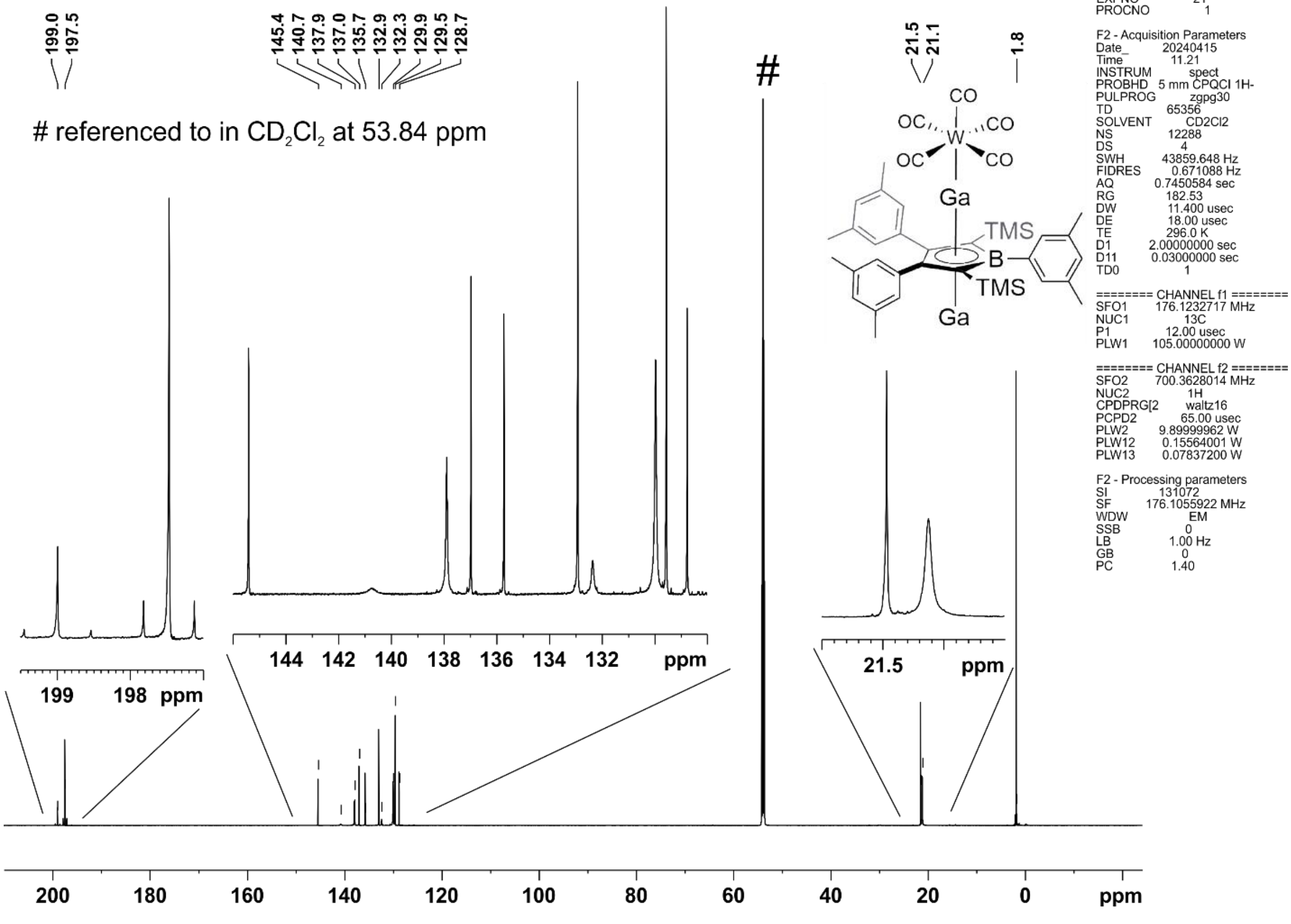

^{11}B-NMR-(background surpressed)-spectrum of compound **3** in CD_2Cl_2

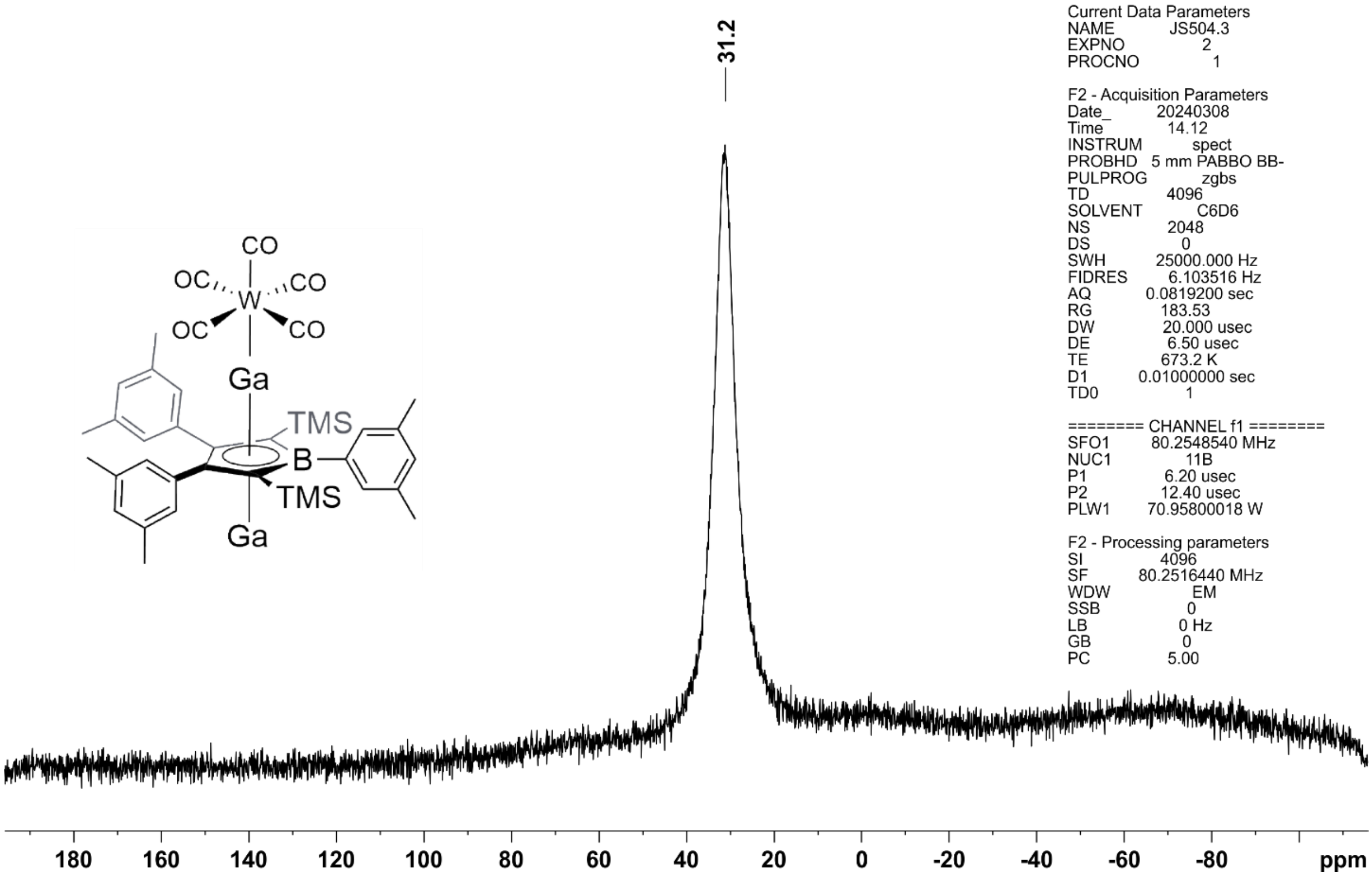

^{29}Si-DEPT-NMR-spectrum of compound **3** in CD_2Cl_2

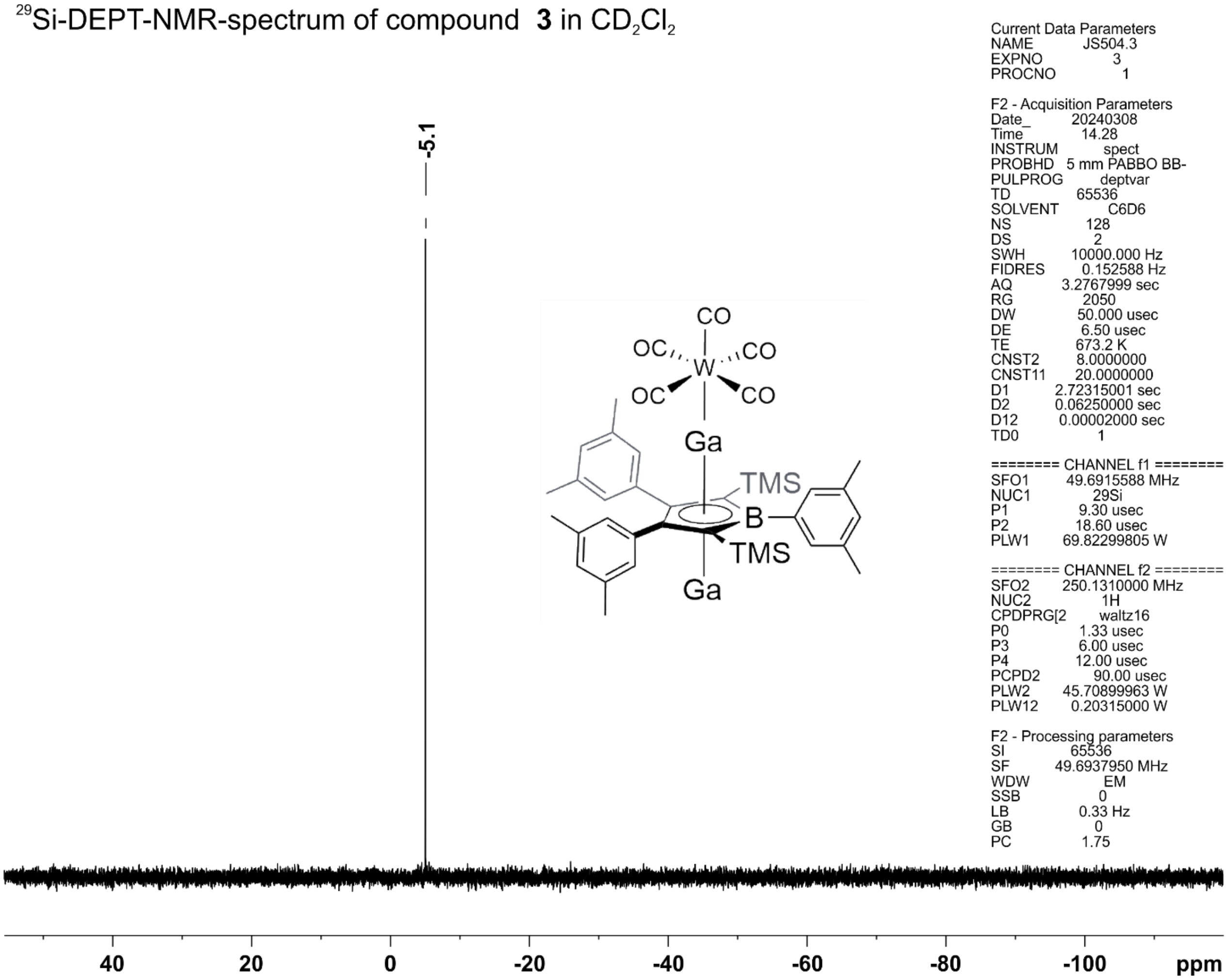

LIFDI-MS of compound **3**

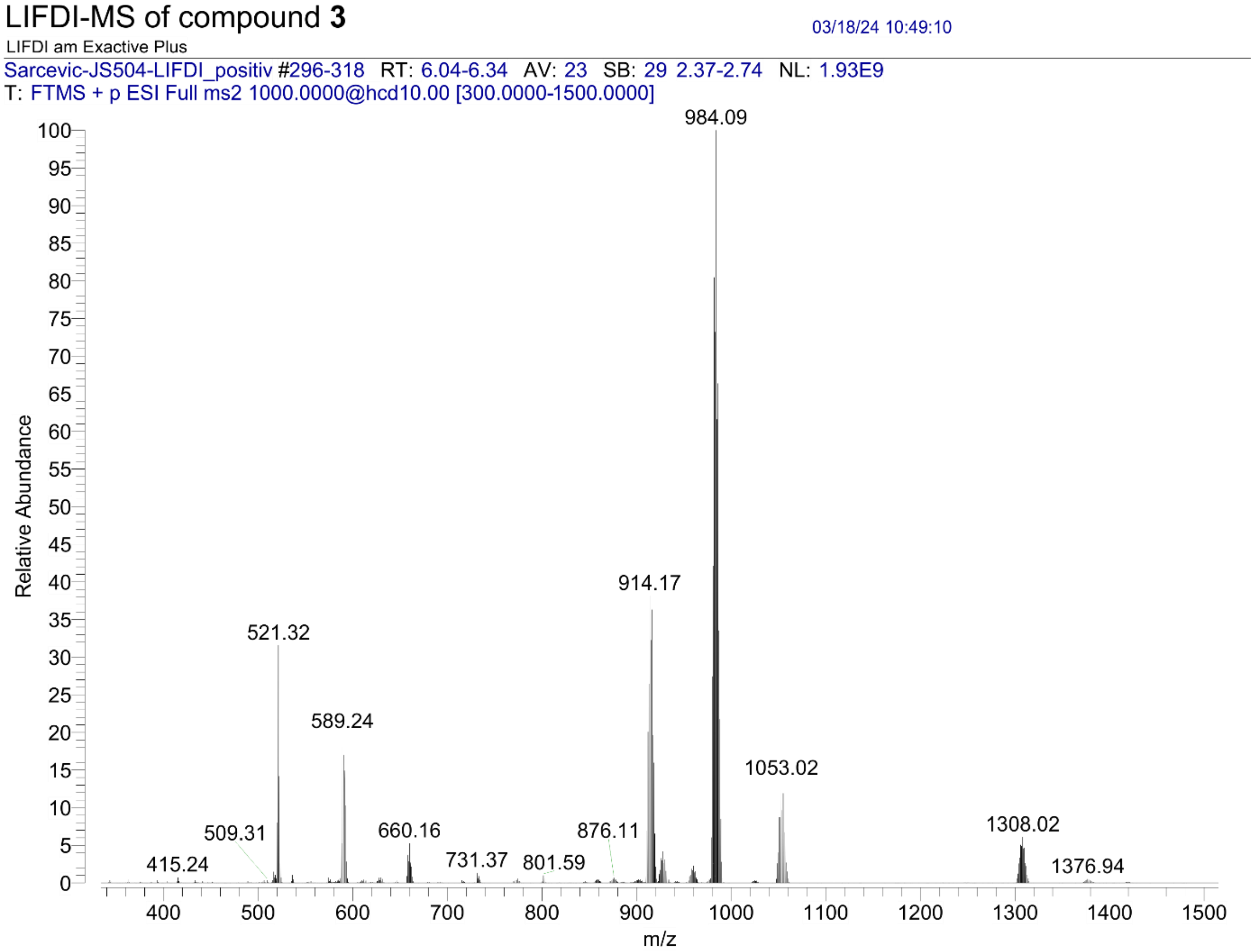

LIFDI-MS of compound **3**

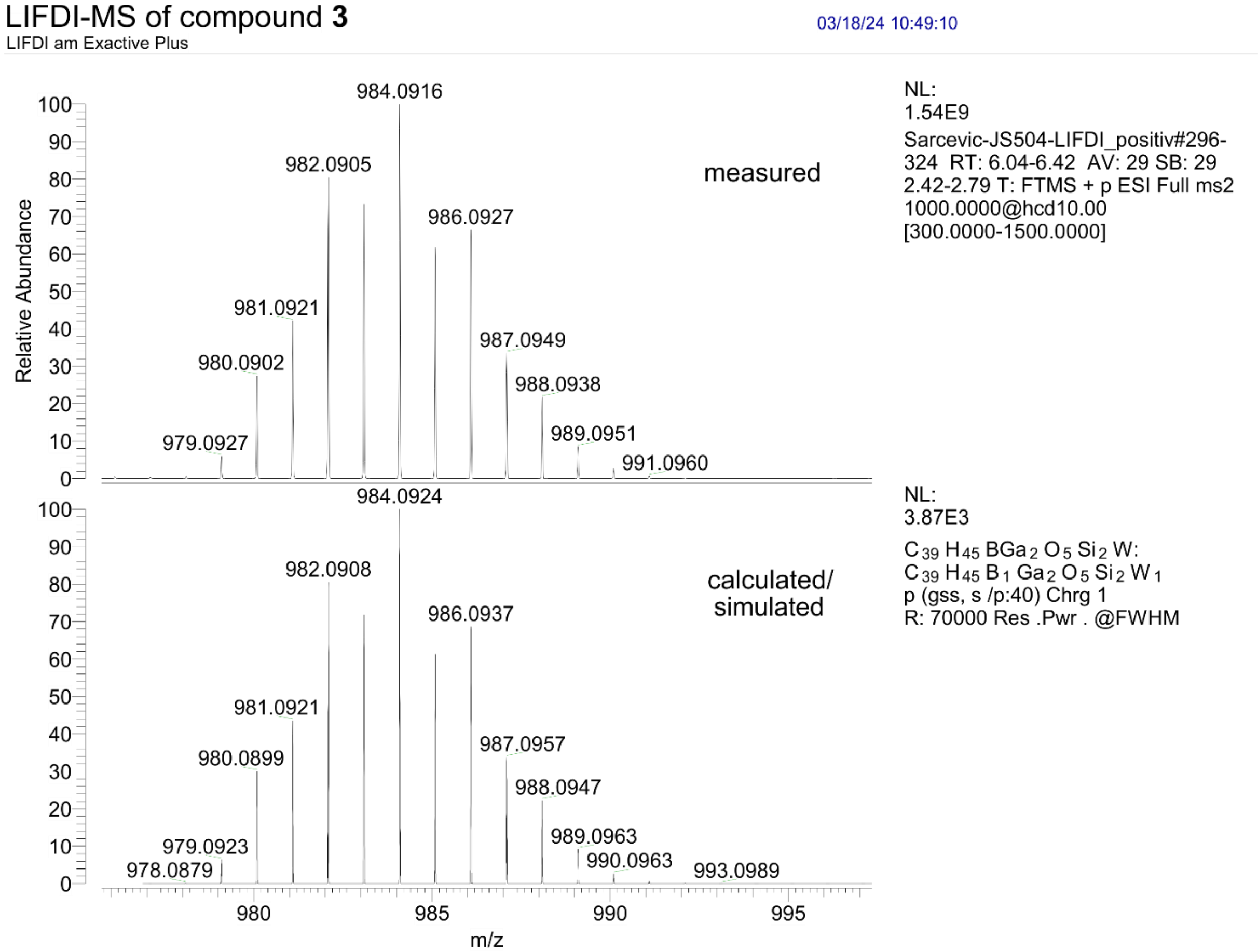

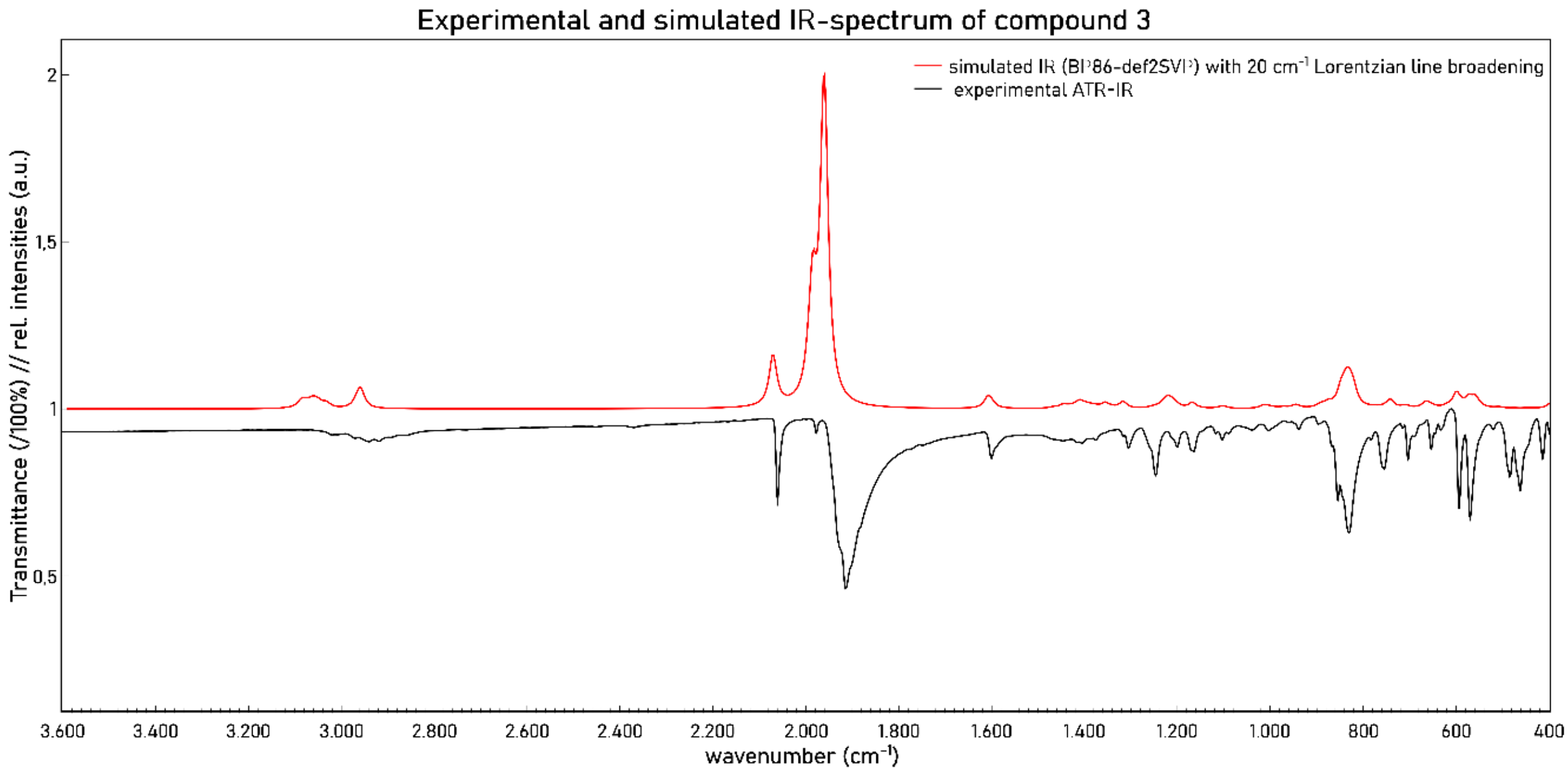

Computational (BP86/def2-SVP) IR intensities of the carbonyl range

Wavenumber (cm^{-1})	Rel. IR intensity (a.u.)		description
1959,42	1396,71	vs	asymm. C=O stretch (mainly eq)
1961,41	1440,44	vs	asymm. C=O stretch (mainly eq)
1985,04	913,97	s	asymm. C=O stretch (mainly ax)
1994,34	97,14	w	asymm. C=O stretch (mainly eq)
2070,65	438,17	m	symm. C=O stretch (ax+eq)

Compound 4

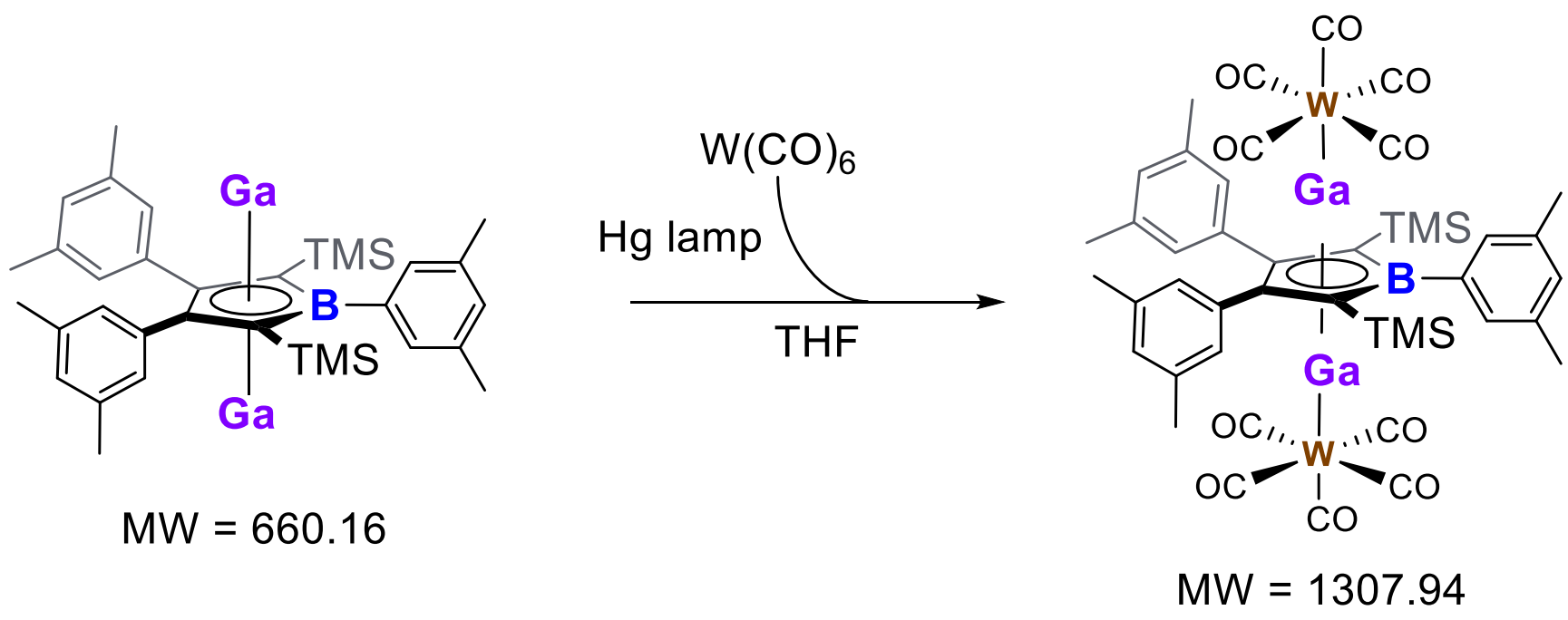

An ampoule, equipped with a Young-type valve, was charged with a solution of tungsten hexacarbonyl (108.5 mg, 0.3083 mmol, 2 eq.) in THF (3 mL). The colorless solution was irradiated over the course of 3 h with a mercury-vapor lamp. The now yellow solution was transferred into a glove box and a solution of digallium cluster **1** (101.6 mg, 0.1539 mmol, 1 eq.) in THF (4 mL) was added. The solution was left to sit at ambient temperature over night. The solvent was removed from the resulting almost colorless solution under reduced pressure. The resulting colorless powder was suspended in hexane (2 mL) which was subsequently removed under reduced pressure to ensure complete removal THF traces by co-evaporation. The colorless powder was washed with hexane (2 × 3 mL) and dried *in vacuo*. The powder was transferred into a syringe filter equipped with a glass fiber pad (Whatman GF/B) and washed with a mixture of toluene (1 mL) and hexane (1 mL). The remaining residue was extracted with dichloromethane (2 mL), which was then removed from the filtrate to give the product in the form of a pale yellow, crystalline solid of di-adduct **4** (68.3 mg, 0.0522 mmol, 34 %). Crystals of **4** suitable for X-ray diffraction were obtained by storing the toluene/hexane washing solution at –40 °C.

Note: The (toluene/hexane) washing step in the syringe filter was necessary to remove traces of the mono tungsten adduct. The solubility of **4** in aliphatic/aromatic hydrocarbon solvents is very poor.

Analytical Data for Compound 4

NMR:

^{1}H (400.10 MHz, 296 K, CD_2Cl_2, CD_1Cl_2H at 5.32 ppm): 7.29 (s, 2H, B-Xy: *o-H*), 7.08 (s, 1H, B-Xy: *p-H*), 6.97 (s, 2H, *p-H*), 6.96 (s, 4H, *o-H*), 2.36 (s, 6H, B-Xy: Ar-C*H$_3$*), 2.21 (s, 12H, Ar-C*H$_3$*), –0.11 (s, 18H, TMS).

^{13}C{^{1}H} (176.12 MHz, 296 K, CD_2Cl_2, solvent signal at 53.84 ppm): 197.1 (O=C_{ax}), 196.1 (O=C_{eq}), 191.8 ($W(CO)_6$), 145.6 (C_β), 138.6 (*m*-C_{ar}), 138.0 (B-Xy: *m*-C_{ar}), 137.6 (B-Xy: *ipso*-C_{ar}, very broad and flat), 133.8 (C_α), 133.5 (*ipso*-C_{ar}), 132.7 (B-Xy: *o*-C_{ar}), 130.7 (*p*-C_{ar}), 130.0 (B-Xy: *p*-C_{ar}), 129.3 (*o*-C_{ar}), 21.4 (B-Xy: *m*-CH_3), 21.0 (*m*-CH_3), 1.5 (TMS).

^{11}B (80.25 MHz, 298 K, CD_2Cl_2): 27.2 ($\omega_{1/2}$ = 603 Hz).

^{29}Si-DEPT (49.69 MHz, 298 K, CD_2Cl_2): –5.3.

^{71}Ga: no signal detected (screened from +300 to – 700 ppm)

Elemental Analysis: ($C_{44}H_{45}BGa_2Si_2W_2O_{10}$) calcd: C 40.41, H 3.47, observed: C 40.31, H 3.44.

LIFDI-MS: calcd exact mass: 1308.0184 m/z, observed m/z: 1308.0184[M]$^+$

IR-ATR: 1916 cm^{-1} (O=C_{eq} stretch), 1999 cm^{-1} (O=C_{ax} stretch), 2065 cm^{-1} (all-symm. stretch).

Molecular structure of Compound 4

For further details on the diffraction measurement please see the respective section.

Crystals suitable for X-ray analysis were grown from *n*-hexane/toluene solutions at –40 °C.

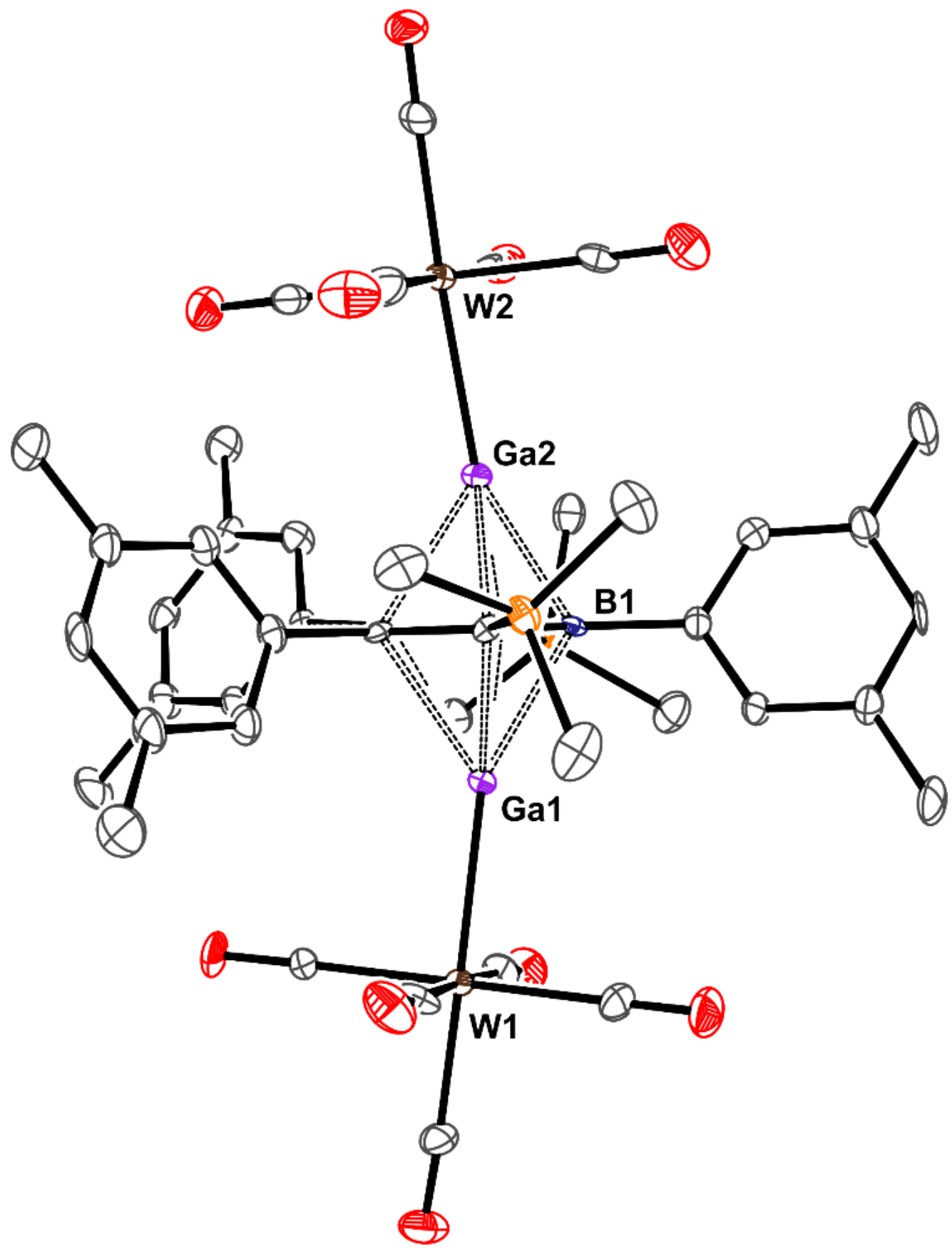

Figure SI6: ORTEP plot of the molecular structure of **4**. Atomic displacement parameters are drawn at 50% probability level. For the sake of clarity, hydrogen atoms are not depicted. Selected bond lengths [Å] and angles [°] are given: B1–Ga1 2.260(9), C1–Ga1 2.31(1), C4–Ga1 2.29(1), C2–Ga1 2.41(1), C3–Ga1 2.361(9), B1–Ga2 2.241(9), C1–Ga2 2.30(1), C4–Ga2 2.29(1), C2–Ga2 2.35(1), C3–Ga2 2.387(9), Ga1–W1 2.547(1), Ga2–W2 2.534(1), W1–C_{ax} 2.003(9), W1–C_{eq} 2.015(9)-2.04(1), W2–C_{ax} 2.002(9), W2–C_{eq} 2.03(1)-2.05(1), Ctr_{BC4}–Ga1 1.934, Ctr_{BC4}–Ga2 1.919, Ga1-Ctr_{BC4}-Ga2 169.6°, W1-Ga1-Ga2 172.47(5)°, W2-Ga2-Ga1 170.18(4)°.The structure was deposited with the CCSD.

Spectra Plots for Compound 4

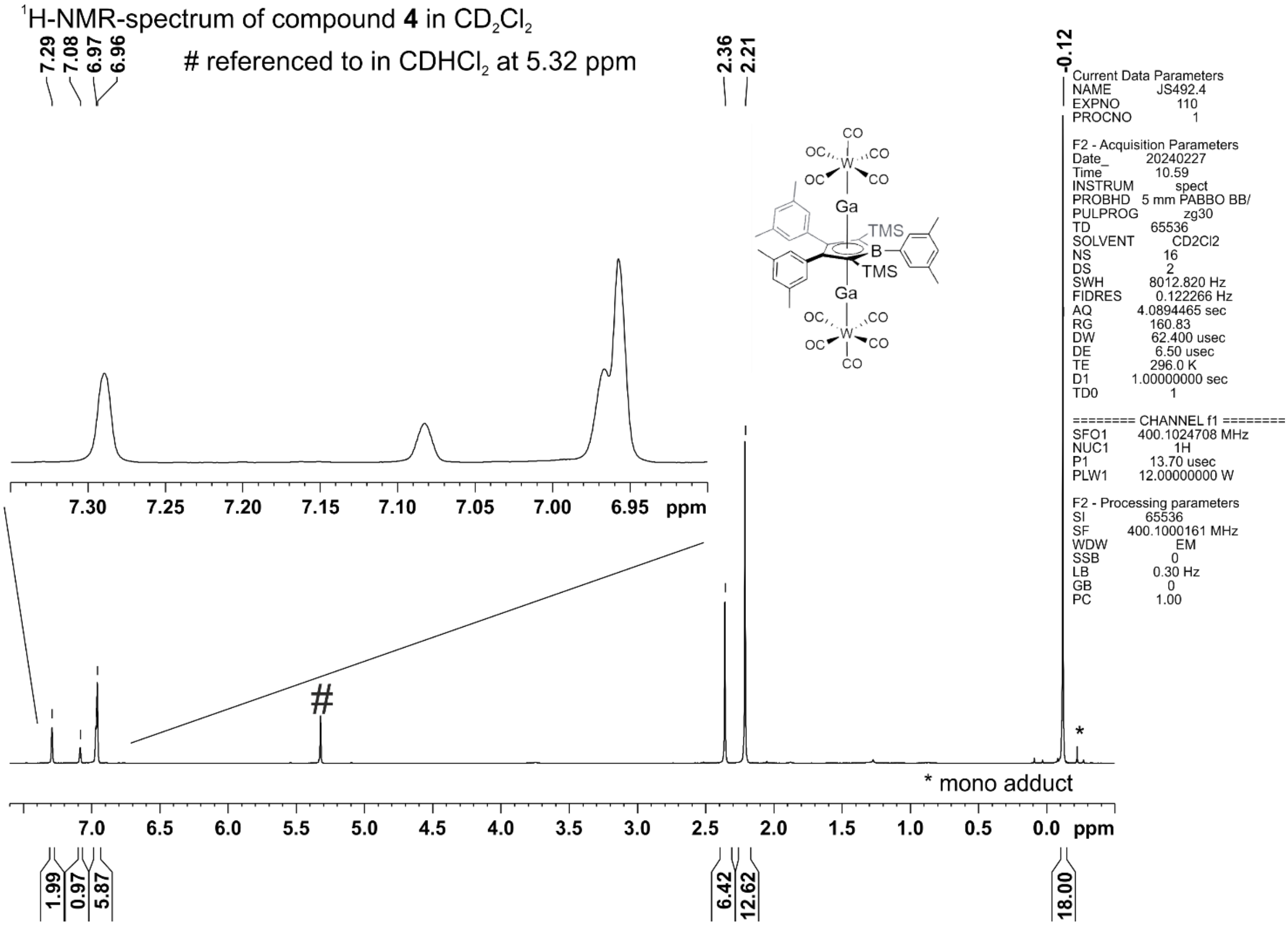

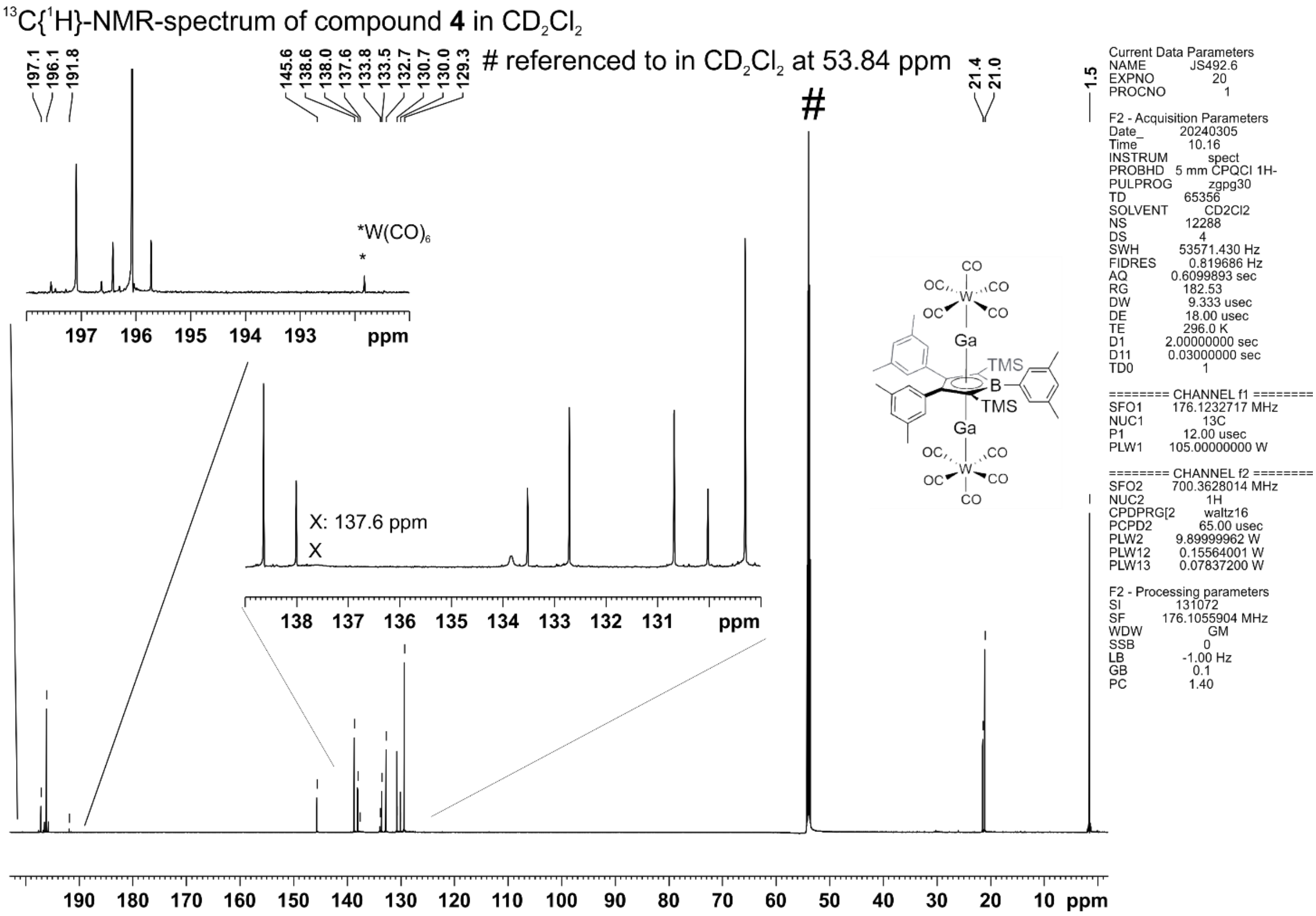

^{11}B-NMR-(background supressed)-spectrum of compound **4** in CD_2Cl_2

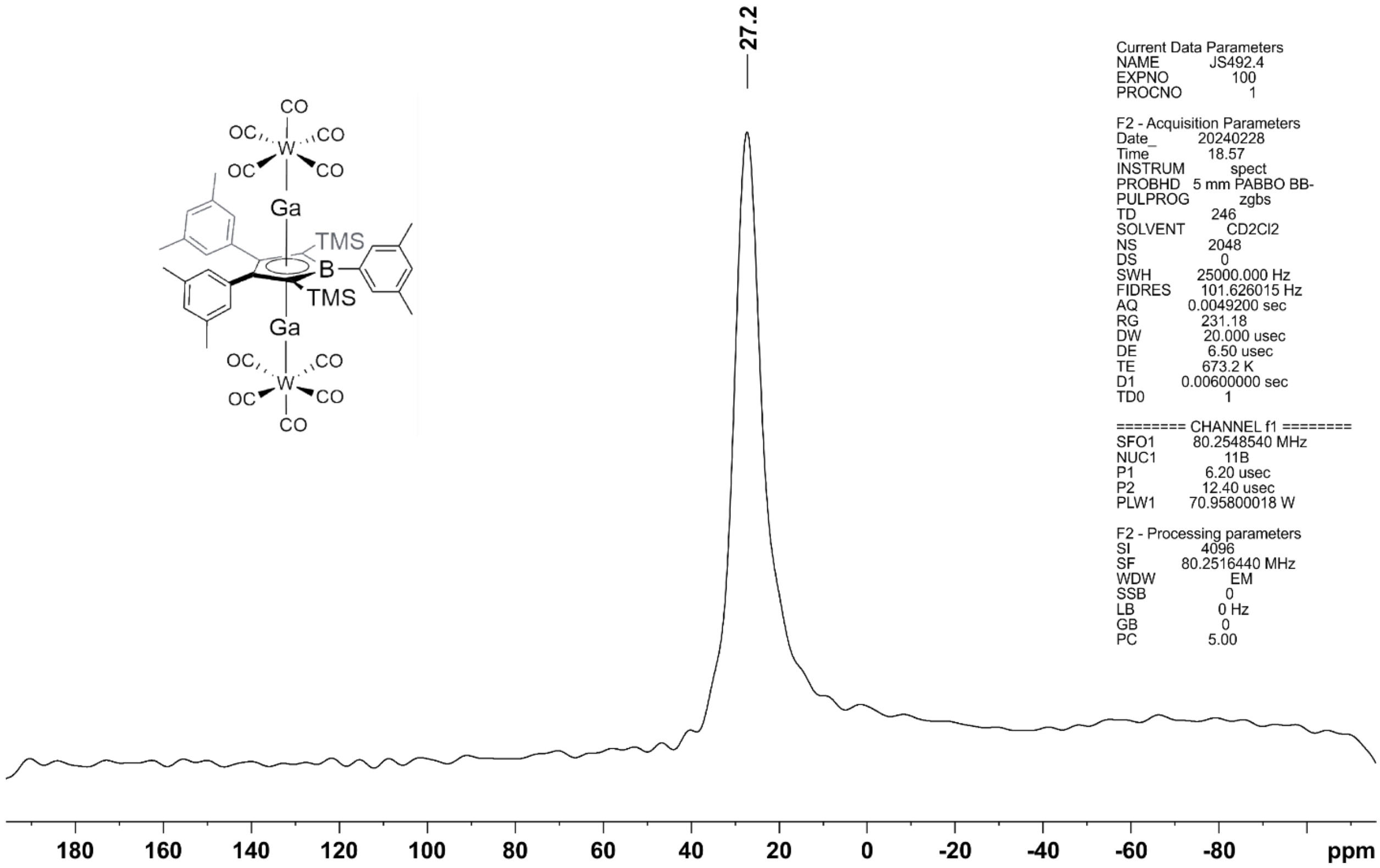

^{29}Si-DEPT-NMR-spectrum of compound **4** in CD_2Cl_2

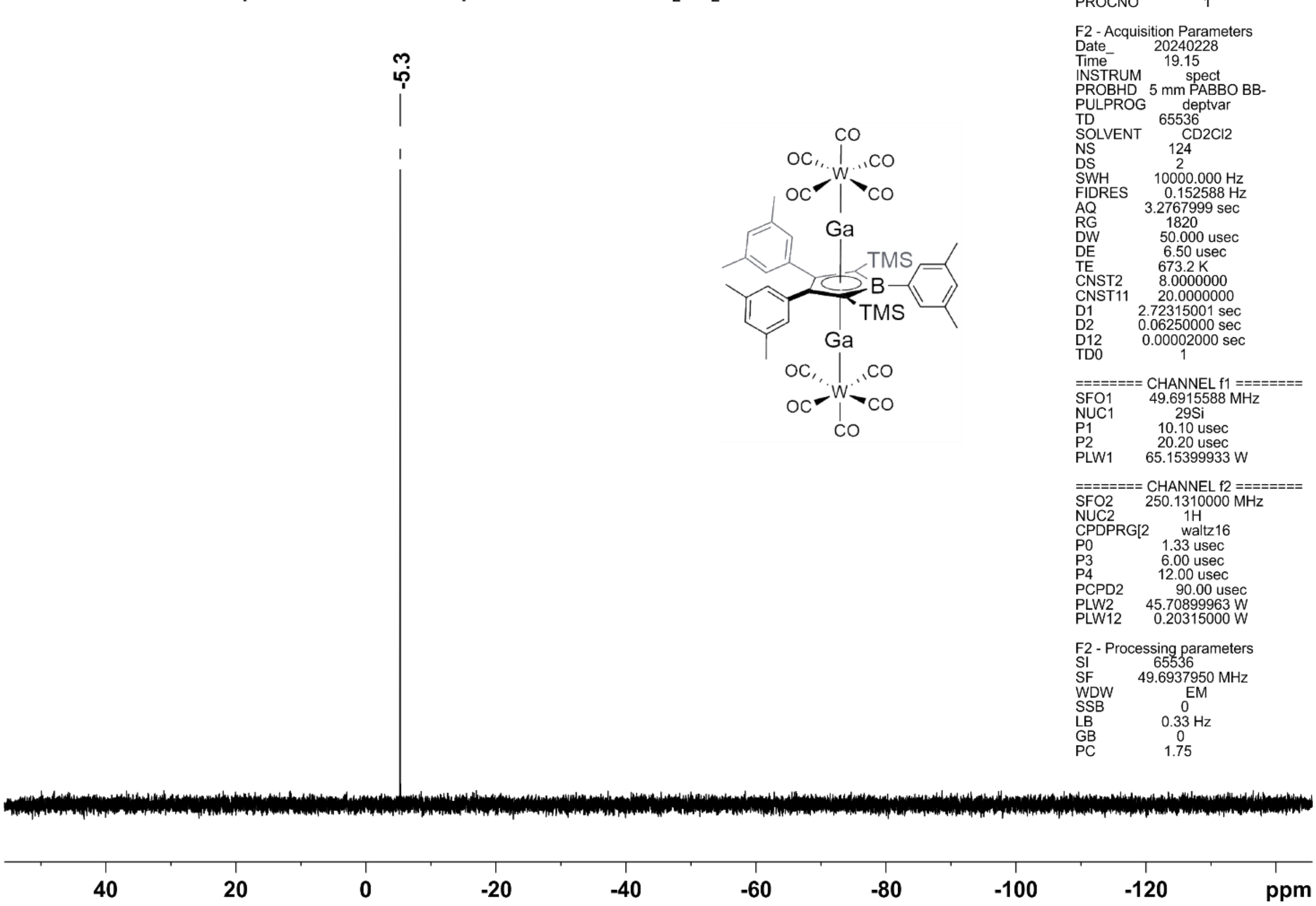

LIFDI-MS of compound **4**

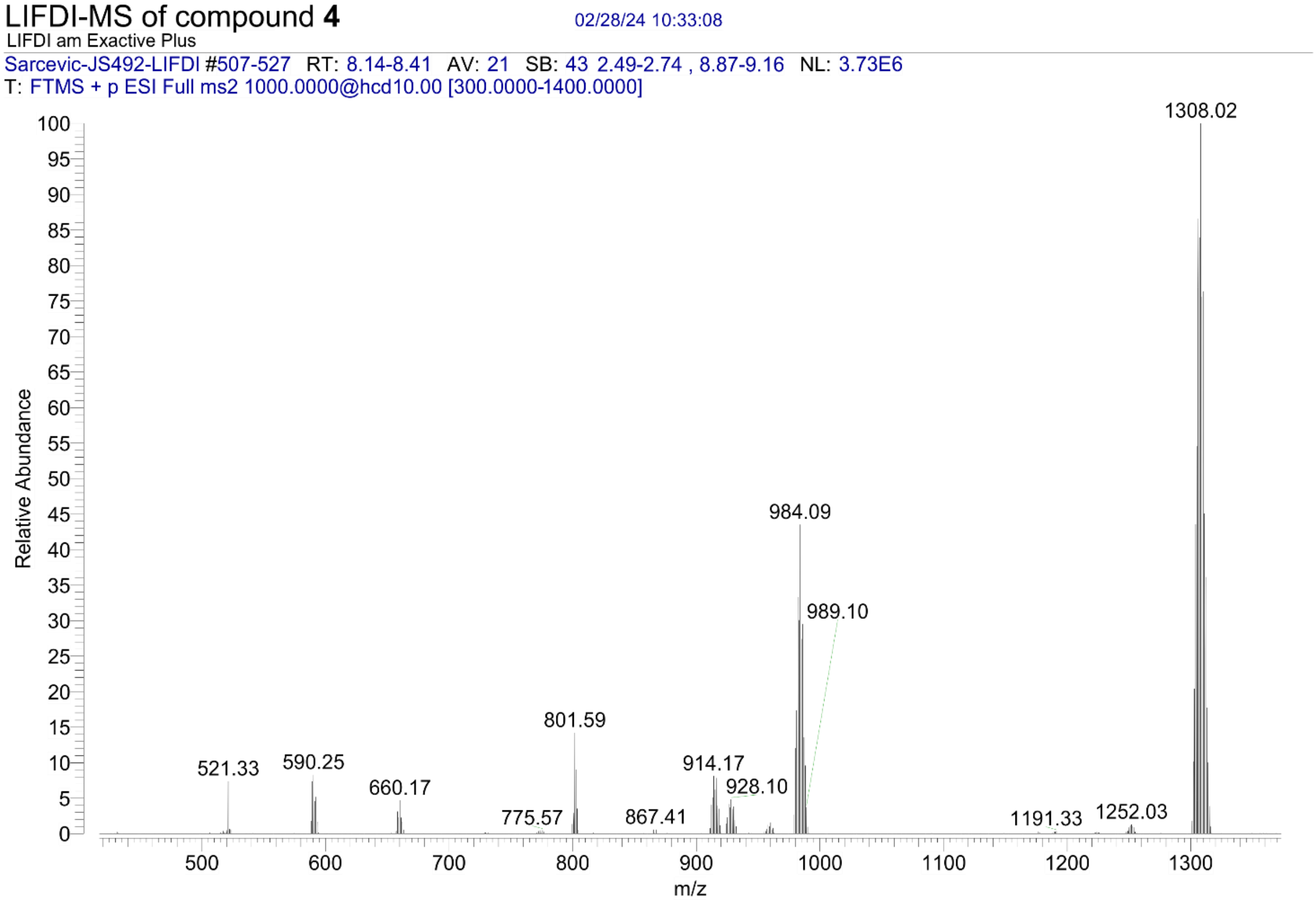

LIFDI-MS of compound **4**

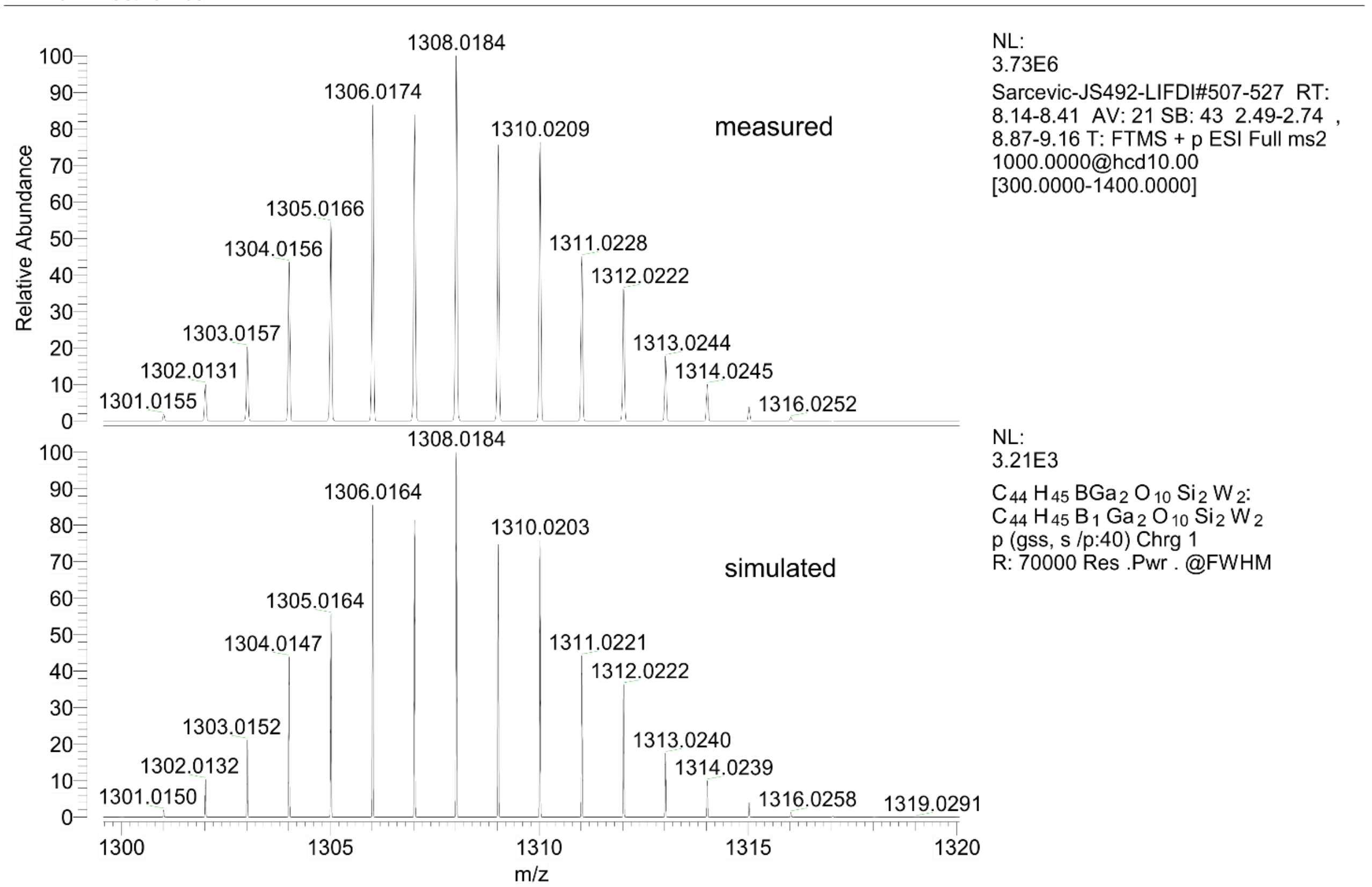

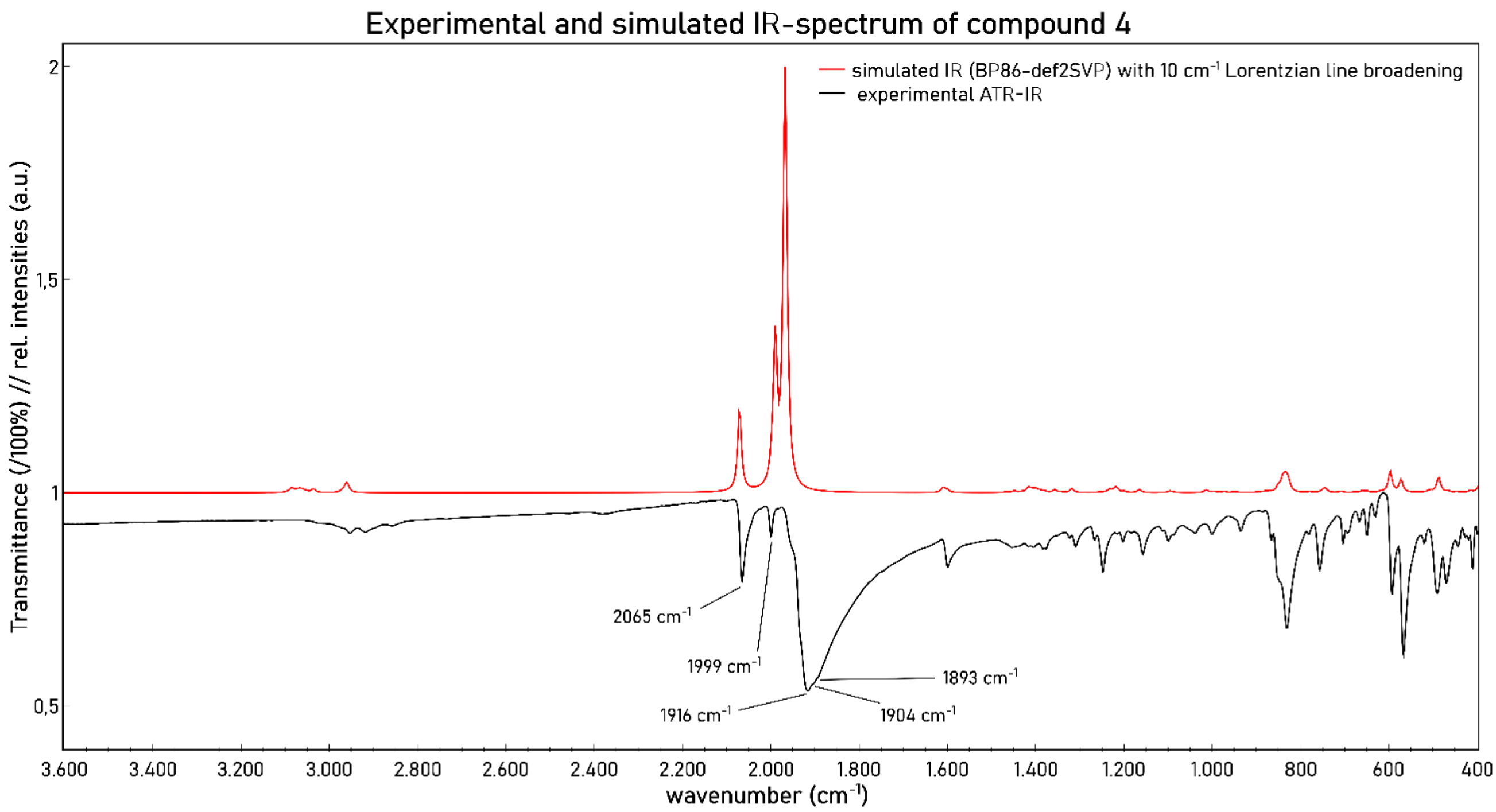

Computational (BP86/def2-SVP) IR intensities of the carbonyl range

Wavenumber (cm^{-1})	Rel. IR intensity (a.u.)		description
1961,2	221,86	w	asymm. C=O stretch (mainly eq)
1963,3	164,66	w	asymm. C=O stretch (mainly eq)
1967,0	2445,78	vs	asymm. C=O stretch (mainly eq)
1968,0	2567,01	vs	asymm. C=O stretch (mainly eq)
1989,4	1620,79	s	asymm. C=O stretch (mainly ax)
1991,0	174,20	w	asymm. C=O stretch (mainly ax)
1996,4	65,53	w	asymm. C=O stretch (mainly eq)
1997,2	114,88	w	asymm. C=O stretch (mainly eq)
2070,5	1008,37	s	symm. C=O stretch (ax+eq)
2077,0	0,62	vw	symm. C=O stretch (ax+eq)

Compound 5

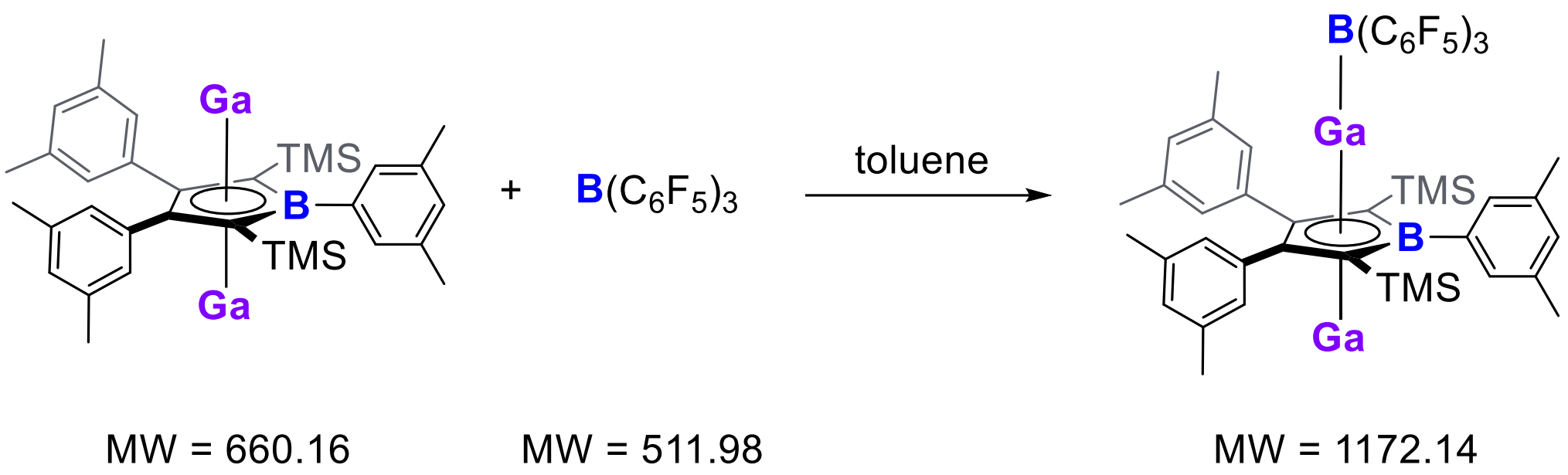

In a glovebox, digallium cluster **1** (26.1 mg, 0.0395 mmol, 1 eq.) and tris(pentafluorophenyl)borane (20.2 mg, 0.0395 mmol, 1 eq.) were solved in toluene (2 mL) and stirred for 1 h at ambient temperature. The solvent was removed under reduced pressure and the solid residue was washed with cold (–40°C) pentane (1 mL) and dried *in vacuo* to give the borane-adduct **5** (29.9 mg, 0.0255 mmol, 65 %) as an off-white powder.

Note: Solutions of **5** repeatedly turned yellow upon storage. ^{19}F-NMR-spectra reveal further signal sets pointing at decomposition of **5**, while ^{1}H-NMR-spectra remained clean.

Analytical Data for Compound 5

NMR:

^{1}H (250.13 MHz, 298 K, C_6D_6, C_6D_5H at 7.15 ppm) 7.51 (s, 2H, B-Xy: *o-H*), 7.13 (s, 4H, *o-H*), 6.91 (s, 1H, B-Xy: *p-H*), 6.55 (s, 2H, *p-H*), 2.33 (s, 6H, B-Xy: Ar-CH_3), 1.99 (s, 12H, Ar-CH_3), –0.04 (s, 18H, TMS).

^{13}C{^{1}H} (100.62 MHz, 298 K, C_6D_6, solvent signal at 128.0 ppm): 145.4 (C_β), 142.7 (in 2D spectra, B-Xy: *ipso*-C_{ar}), 137.2 (*m*-C_{ar}), 136.3 (B-Xy: *m*-C_{ar}), 133.2 (B-Xy: *o*-C_{ar}), 132.6 (C_α, only visible in 2D spectra), 130.3 (*o*-C_{ar}), 129.3 (*p*-C_{ar}), 128.7 (B-Xy: *p*-C_{ar}), 21.6 (B-Xy: *m*-CH_3), 21.0 (*m*-CH_3), 2.6 (TMS). Not found: *ipso*-C_{ar}, BCF

^{11}B (80.25 MHz, 298 K, C_6D_6): 36.5 (br, C_4B), 31.8 (br, Ga-$B(C_6F_5)_3$)

^{11}B (128.38 MHz, 223 K, C_6D_6 /tol-*d8*): 31 (very broad, C_4B), –10 (br, Ga-$B(C_6F_5)_3$)

^{29}Si-DEPT (49.69 MHz, 298 K, C_6D_6): –7.6.

^{71}Ga: no signal detected (screened from +300 to – 700 ppm)

^{19}F (376.43 MHz, 298 K, C_6D_6): –127.9 (m, *o-F*), –146.9 (m, *p-F*), –160.8 (m, *m-F*).

Elemental Analysis: ($C_{52}H_{45}B_2F_{15}Ga_2Si_2$) calcd: C 53.28, H 3.87, observed: C 54.13, H 4.23.

Molecular structure of Compound 5

For further details on the diffraction measurement please see the respective section.

Crystals suitable for X-ray analysis were grown from saturated solutions in pentane at –40 °C.

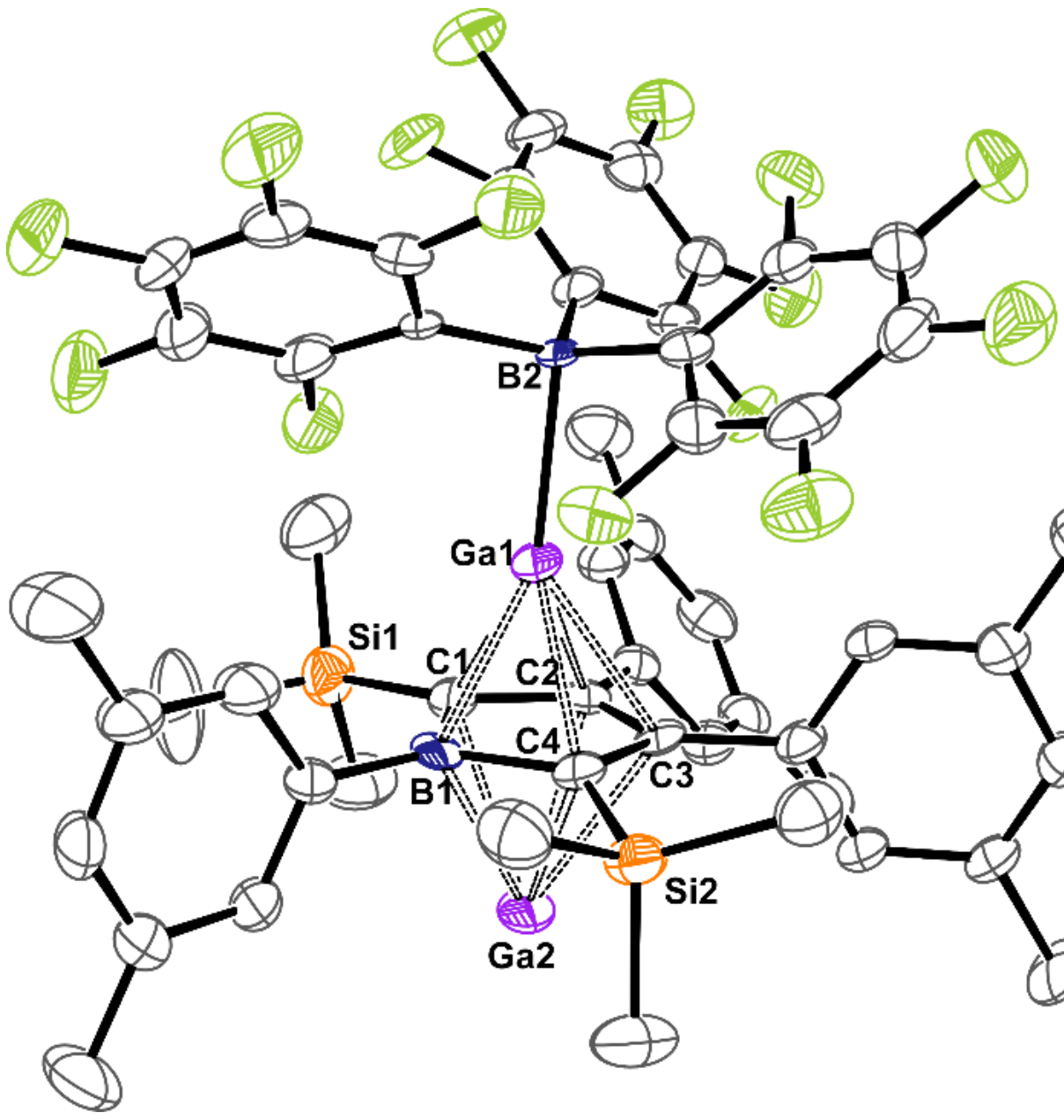

Figure SI7: ORTEP plot of the molecular structure of **5**. Atomic displacement parameters are drawn at 50% probability level. For the sake of clarity, hydrogen atoms and a lattice pentane molecule are not depicted. Selected bond lengths [Å] and angles [°] are given: B1–C1 1.568(8), C1–C2 1.488(8), C2–C3 1.437(8), C3–C4 1.470(6), C4–B1 1.581(9), B1–Ga2 2.396(5), C1–Ga2 2.421(5), C2–Ga2 2.432(5), C3–Ga2 2.459(5), C4–Ga2 2.474(5), B1–Ga1 2.245(5), C1–Ga1 2.243(5), C2–Ga1 2.342(5), C3–Ga1 2.359(5), C4–Ga1 2.281(5), Ga1–B2 2.251(5), Ctr_{BC4}–Ga1 1.902, Ctr_{BC4}–Ga2 2.071, Ga1-Ctr_{BC4}-Ga2 172.7°, Ctr_{BC4}-Ga1-B2 170.6°.

Spectra Plots for Compound 5

^{1}H-NMR-spectrum of compound **5** in C_6D_6

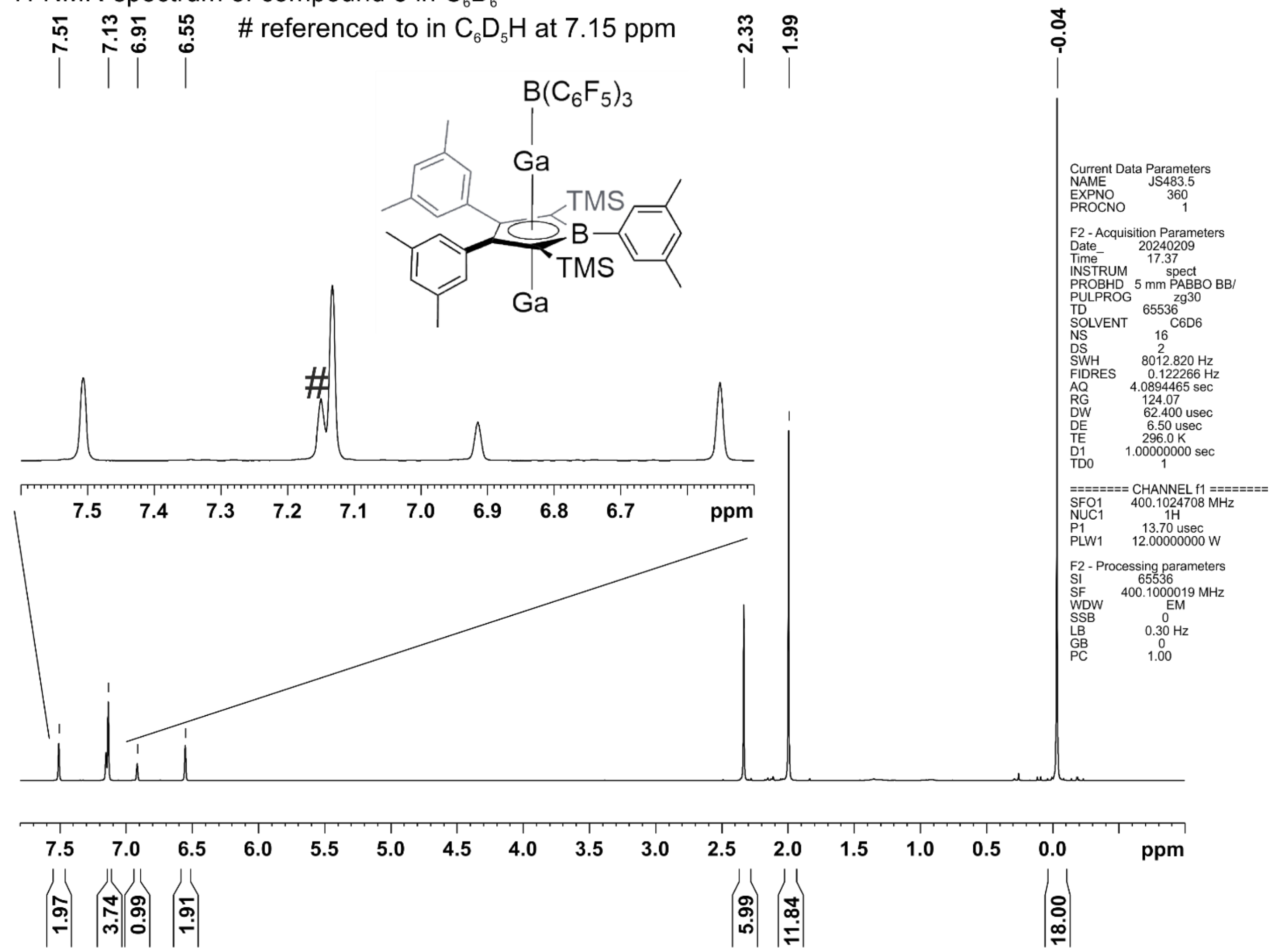

^{13}C{^{1}H}-NMR-spectrum of compound **5** in C_6D_6

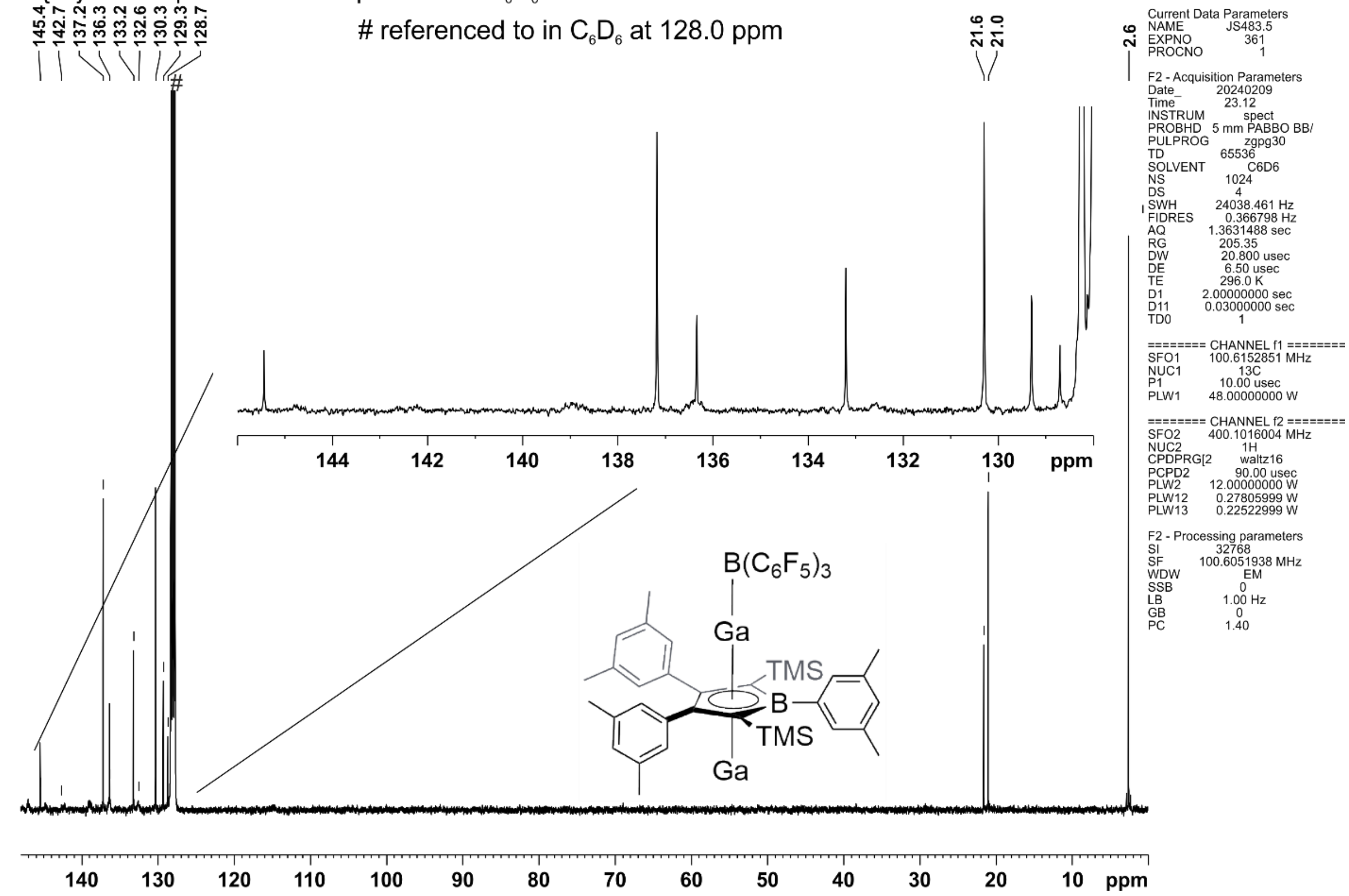

^{11}B-NMR-spectrum (background suppressed) of compound **5** in C_6D_6

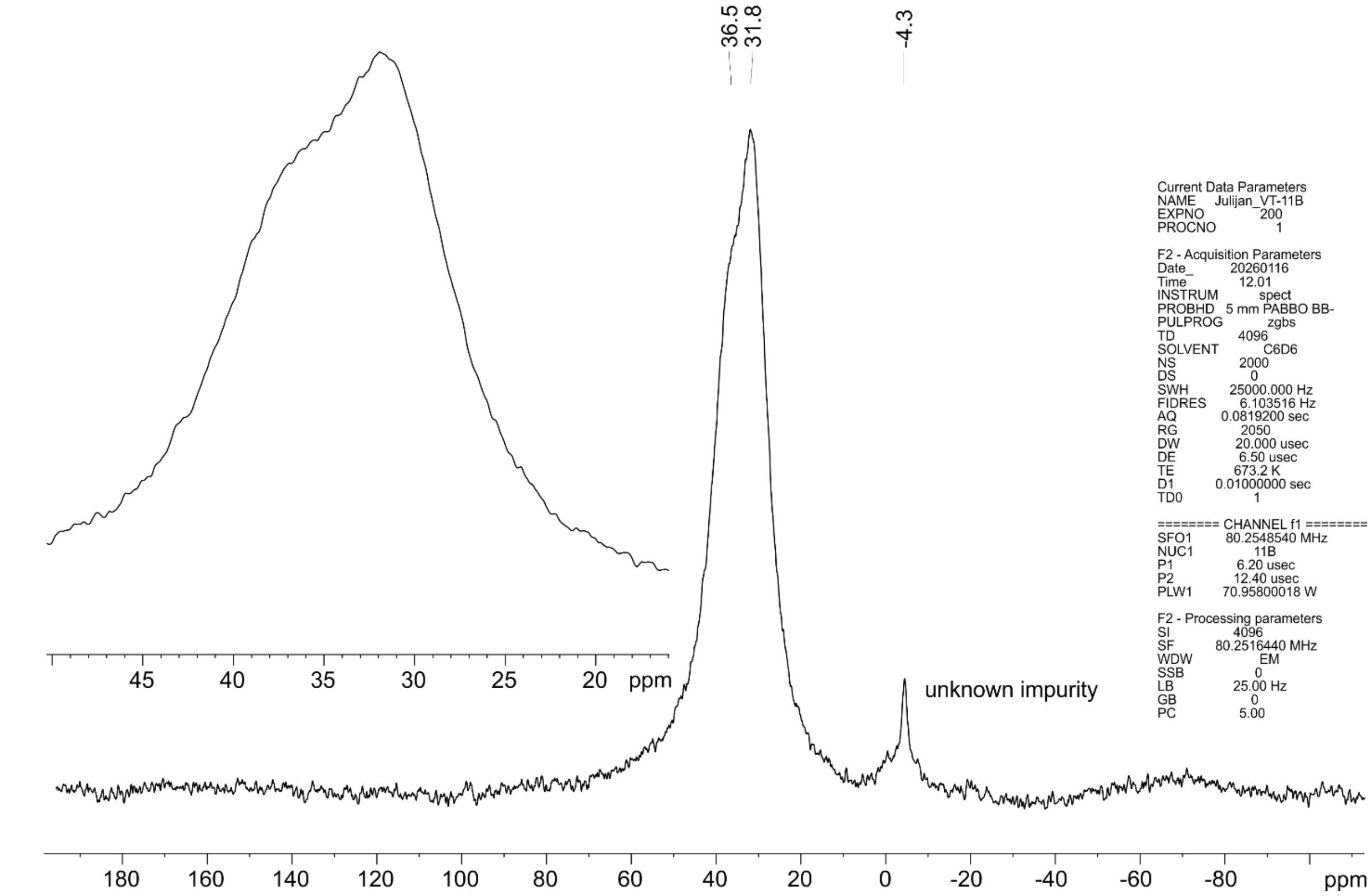

^{19}F-NMR-spectrum of compound **5** in C_6D_6

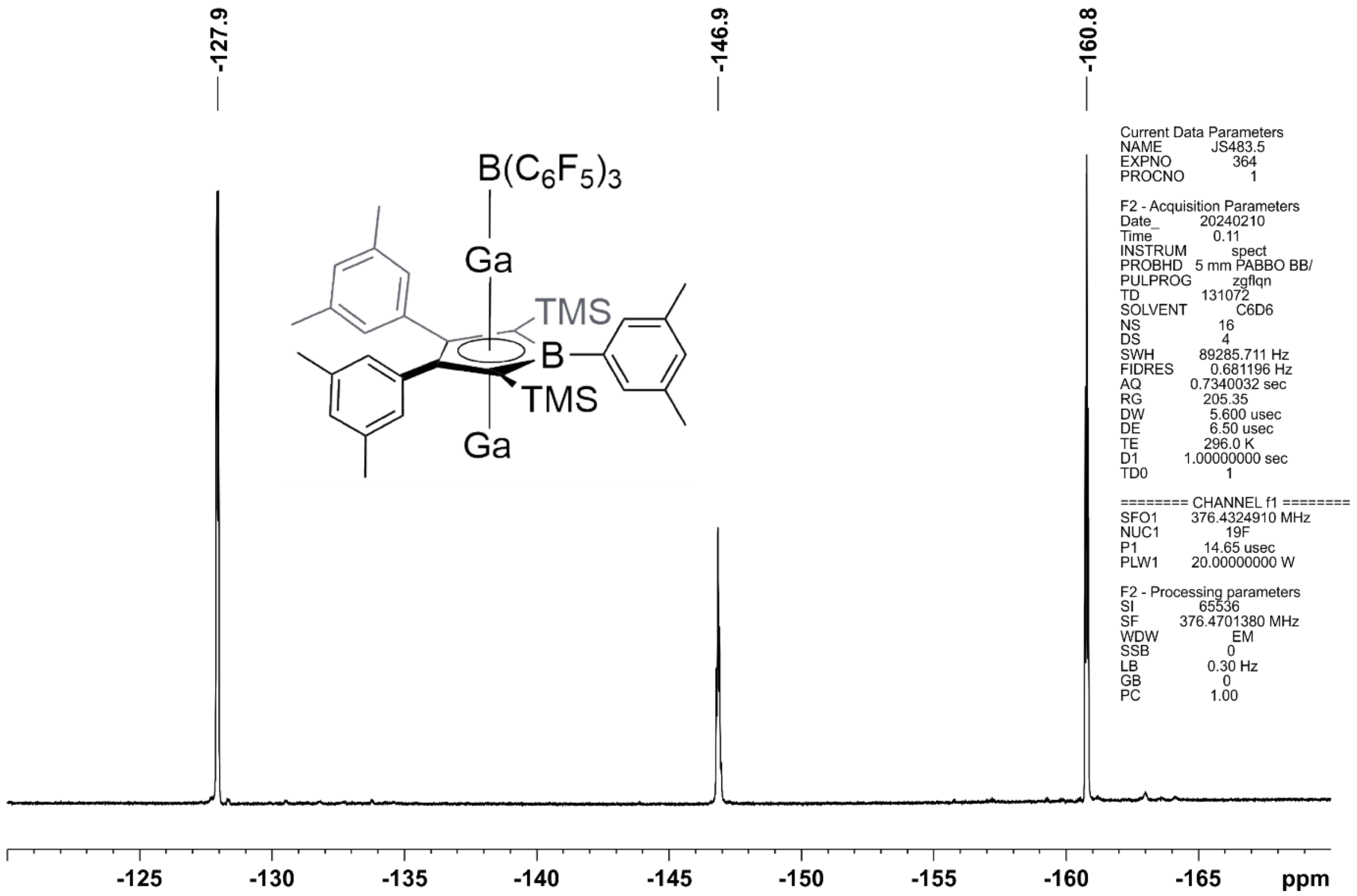

^{29}Si-DEPT-NMR-spectrum of compound **5** in C_6D_6

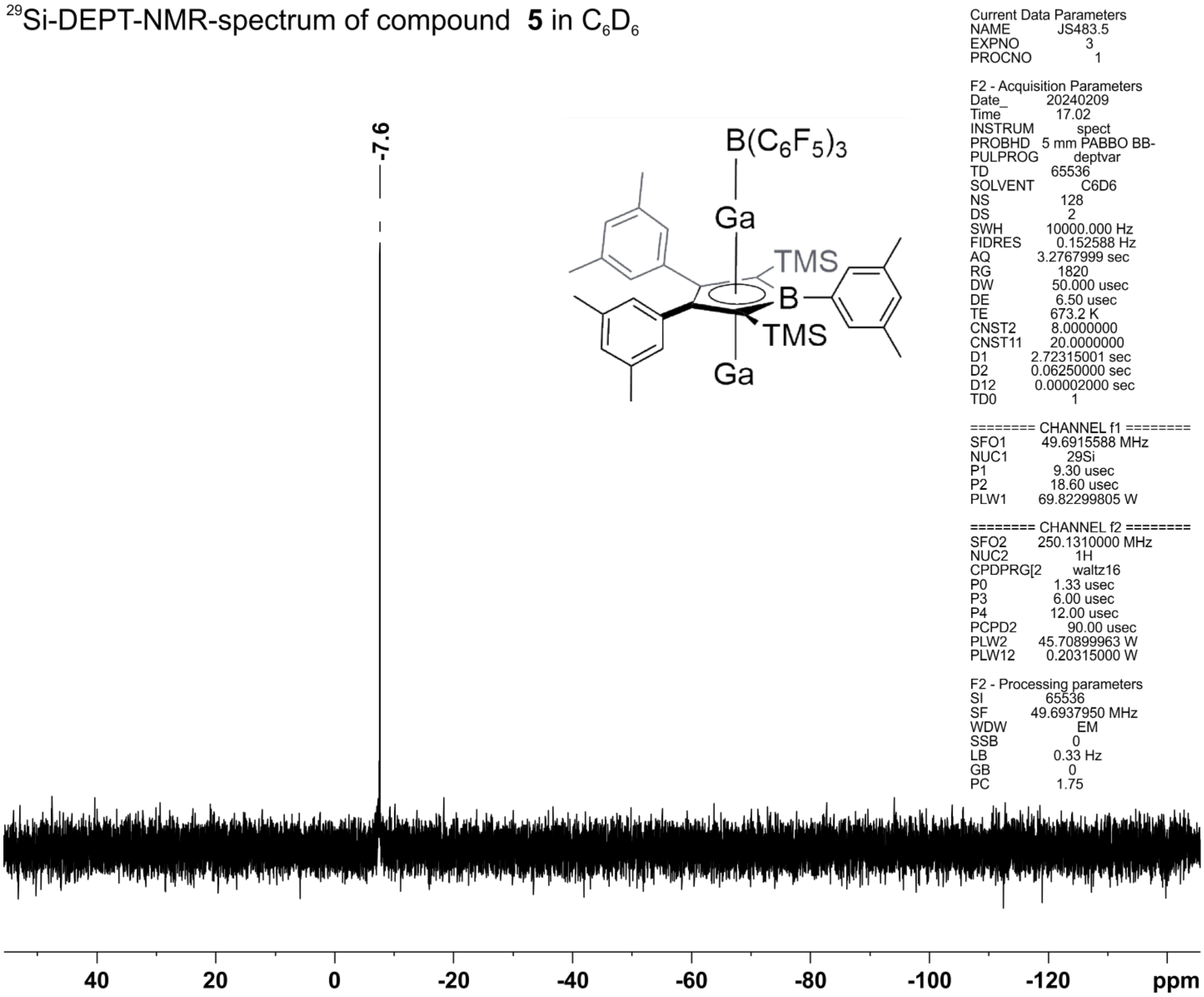

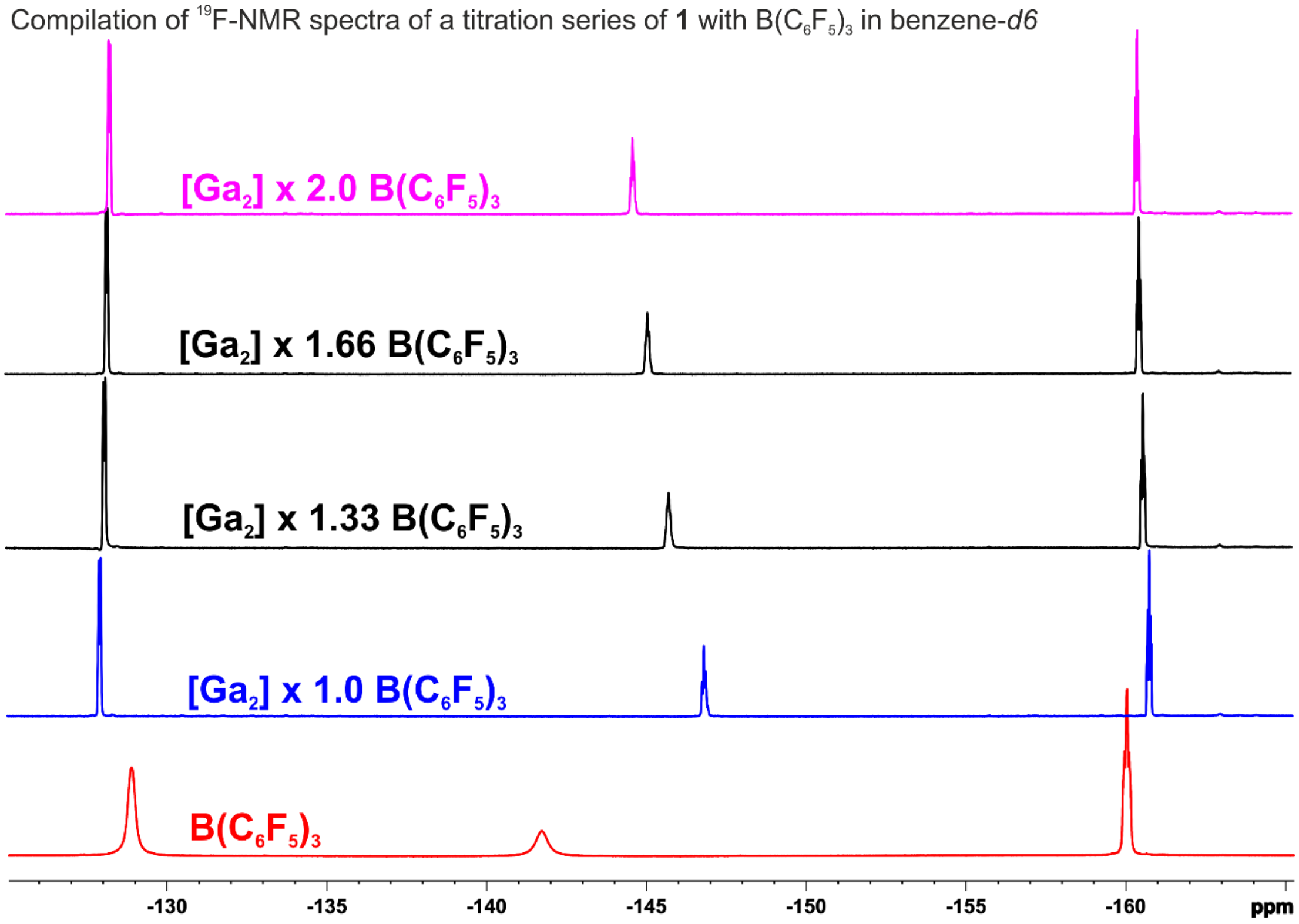

Figure SI8: ^{19}F-NMR-monitoring of the conversion of titration of **1** with $B(C_6F_5)_3$

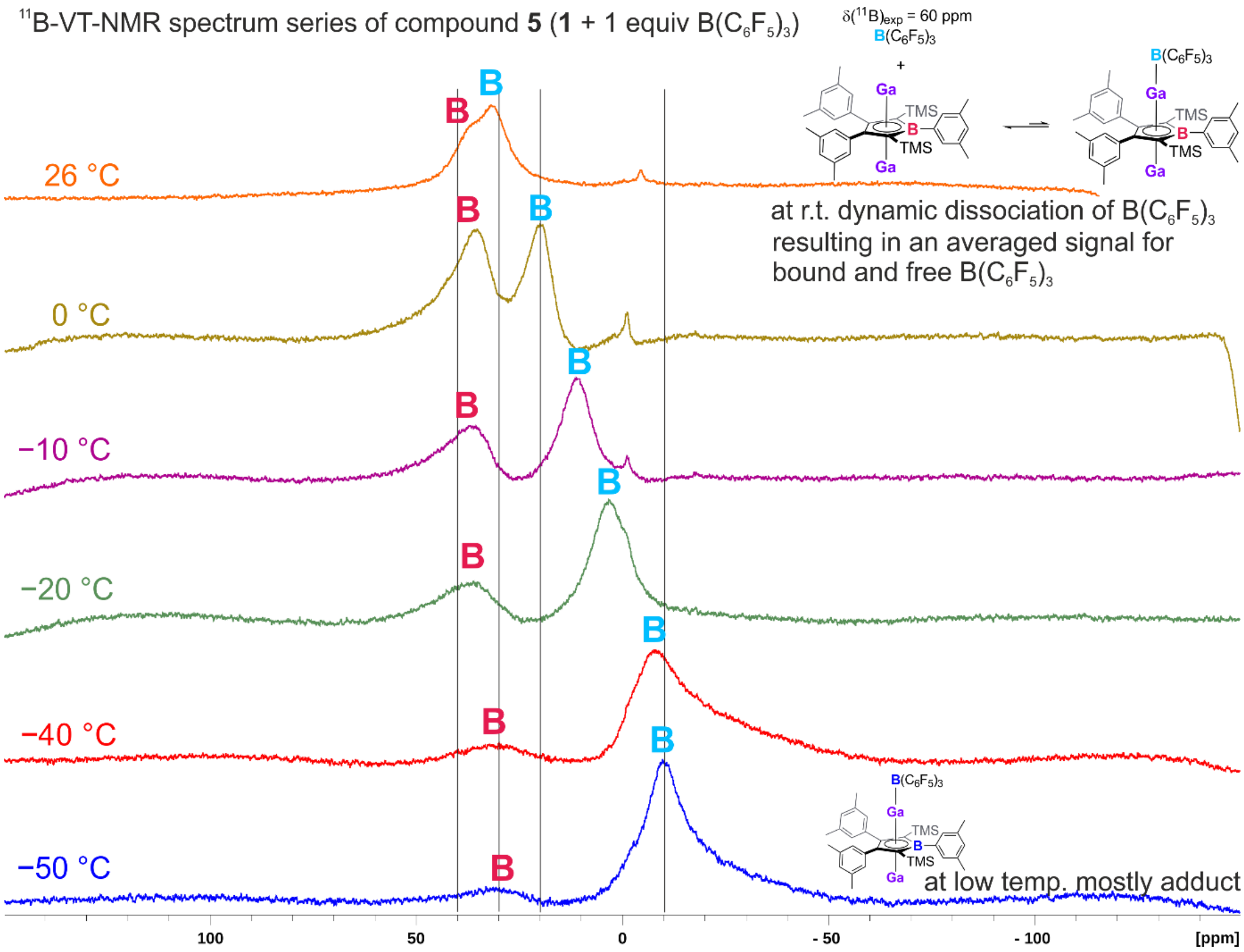

Figure SI9: ^{11}B-variable temperature-NMR spectra of **5** (**1** with one equiv. of $B(C_6F_5)_3$

Compound 6

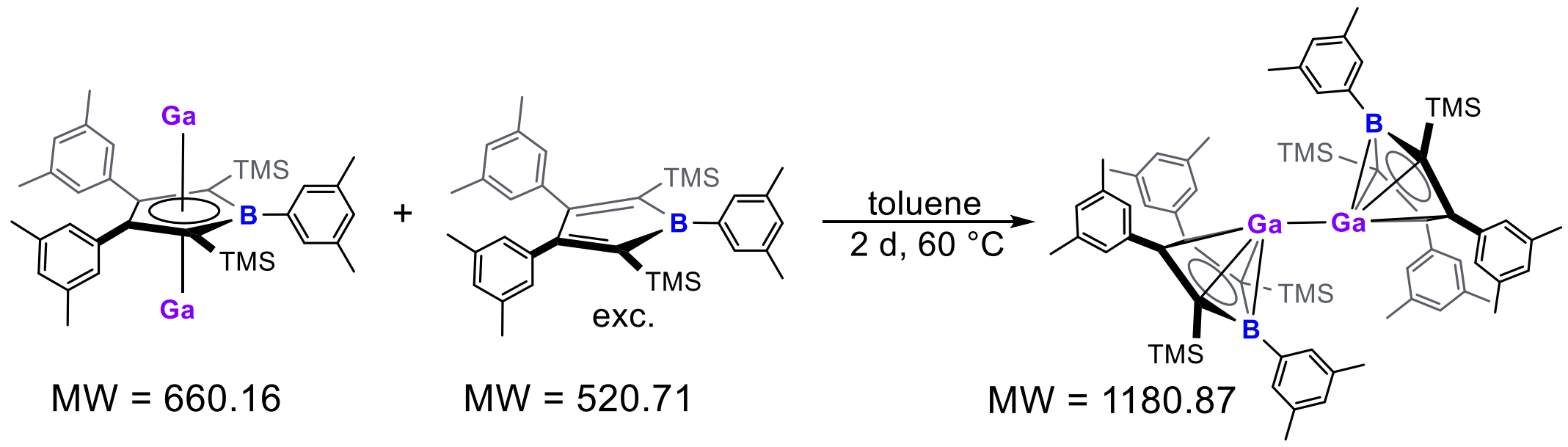

In a glovebox, an ampoule equipped with a Young-type valve, was charged consecutively with a solution of gallium cluster **1** (98.4 mg, 0.149 mmol, 1 eq.) in toluene (2 mL) and red solution of borole **A** (388.9 mg, 0.7468 mmol, 5 eq.) in toluene (3 mL). The ampoule was transferred out of the glovebox and the solution was kept at 60 °C for two days after which the solvent was removed under reduced pressure to give a red crystalline solid. The ampule was reintroduced to the glovebox and the crystalline, red solid was repeatedly washed with pentane (3 × 3 mL, 2 × 2 mL). After drying *in vacuo*, the digallane **6** (154.3 mg, 0.1307 mmol, 88 %) was isolated as a bright orange powder.

Note: The conversion of **1** and free borole proceeds selectively, but very slow. The excess of borole is applied to drive the reaction to completion, while heating to 60 °C allows for a reasonable conversion time. Crystals suitable for X-ray diffraction were obtained from saturated solutions in toluene.

Analytical Data for Compound 6

NMR:

^{1}H (500.14 MHz, 296 K, C_6D_6, C_6D_5H at 7.15 ppm): 7.54 (s, 4H, B-Xy: *o-H*), 7.19 (s, 8H, *o-H*), 6.92 (s, 2H, B-Xy: *p-H*), 6.62 (s, 4H, *p-H*), 2.36 (s, 12H, B-Xy: Ar-C*H₃*), 2.16 (s, 24H, Ar-C*H₃*), 0.09 (s, 36H, TMS).

^{13}C{^{1}H} (125.77 MHz, 296 K, C_6D_6, solvent signal at 128.0 ppm): 142.7 (C_β), 137.9 (*ipso*-C_{ar}), 137.1 (*m*-C_{ar}), 136.1 (B-Xy: *m*-C_{ar}), 133.5 (B-Xy: *o*-C_{ar}), 130.6 (*o*-C_{ar}), 129.4 (*p*-C_{ar}), 128.6 (B-Xy: *p*-C_{ar}), 127.3 (B-Xy: *ipso*-C_{ar}, broad), 114.6 (C_α), 21.7 (B-Xy: *m*-CH_3), 21.3 (*m*-CH_3), 3.1 (TMS).

^{11}B (80.25 MHz, 298 K, C_6D_6): 32.1 ($\omega_{1/2}$ = 1850 Hz).

^{29}Si-DEPT (49.69 MHz, 298 K, , C_6D_6): –7.3.

^{71}Ga: no signal detected (screened from +300 to – 700 ppm)

Elemental Analysis: ($C_{68}H_{90}B_2Ga_2Si_4$) calcd C 69.16, H 7.68; observed C 69.35, H 8.32.

UV-Vis: λ_{max} 426 nm (toluene).

Molecular structure of Compound 6

For further details on the diffraction measurement please see the respective section. Crystals suitable for X-ray analysis were grown from saturated solutions in toluene at –40 °C.

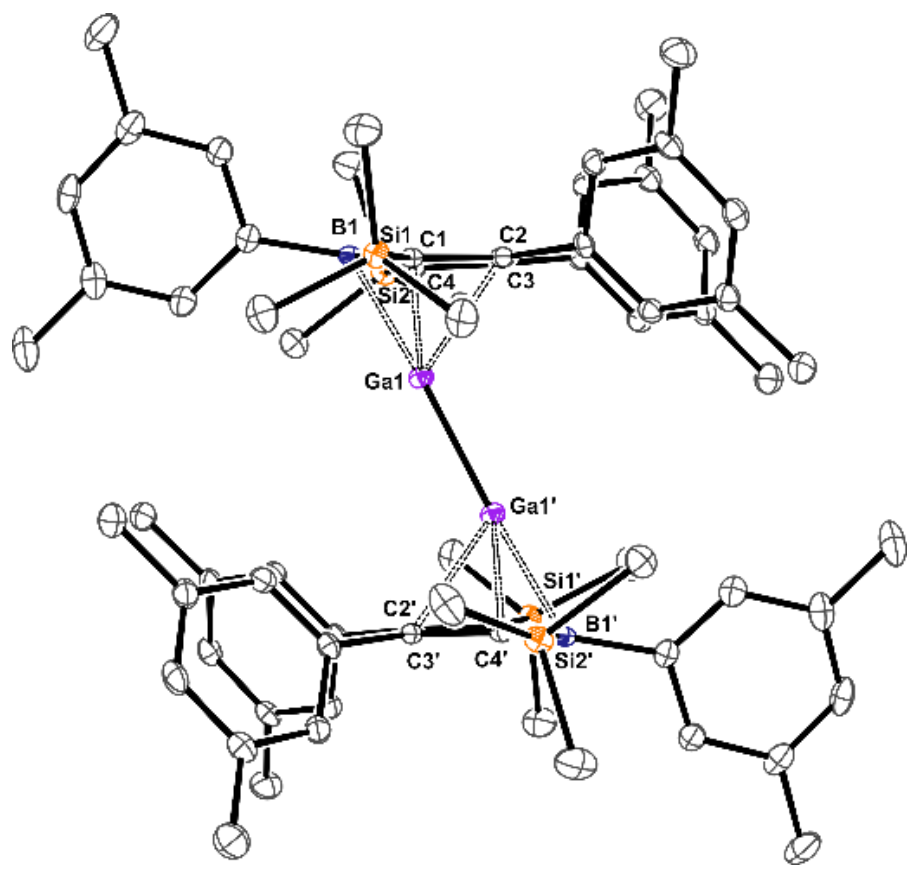

Figure SI10: ORTEP plot of the molecular structure of **6**. Atomic displacement parameters are drawn at 50% probability level. Hydrogen atoms and a lattice toluene molecule are omitted for clarity. Selected bond lengths [Å] and angles [°] are given: Ga1–Ga1' 2.3701(4), B1–C1 1.581(2), C1–C2 1.462(1), C2–C3 1.429(2), C3–C4 1.459(1), C4–B1 1.575(2), B1–Ga1 2.162(1), C1–Ga1 2.149(1), C2–Ga1 2.294(1), C3–Ga1 2.292(1), C4–Ga1 2.147(1), Ctr_{BC4}–Ga1 1.804, Ctr_{BC4}-Ga1-Ga1' 145.2°, B1-Ga1-Ga1' 176.8°. The structure was deposited with the CCSD.

To rule out a putative hydride-bridged dimer, a high quality/high-resolution X-ray diffraction data set with resolution down to 0.45 Å has been obtained. Refinement of the data applying the independent atom model and inspection of the difference density map (F_o-F_c) at a level of 0.13 e/Å allows for clear identification of (a deliberately unmodelled) rotationally disordered methyl group , while (apart from spherical residual electron density around the Ga-atoms) no residual electron density that could be reasonable associated with bridging hydrides between the Ga-atoms is observed.

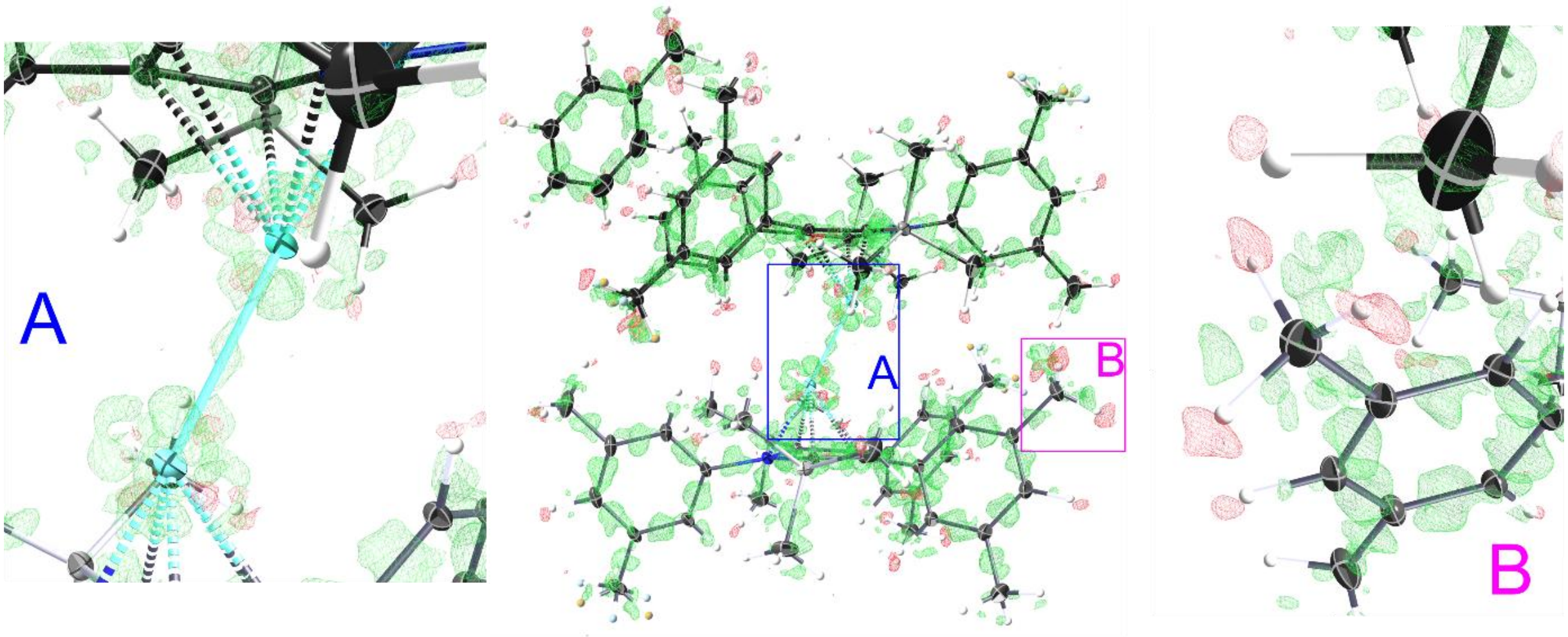

Figure SI11: Depictions of the residual electron density (F_o-F_c) map of an IAM-refined high-resolution (0.45 Å) data set of digallane **6**. Full molecule (central) and excerpts (A, right and B, left) are shown. **A** details the Ga-Ga bonding, while **B** details a deliberately unmodelled rotationally disordered methyl group. Resolution is sufficient to clearly identify residual electron density of fractions of H-atoms in the methyl group, while no reasonable electron density associable with bridging hydrides is observed.

Spectra Plots for Compound 6

^{1}H-NMR-spectrum of compound **6** in C_6D_6

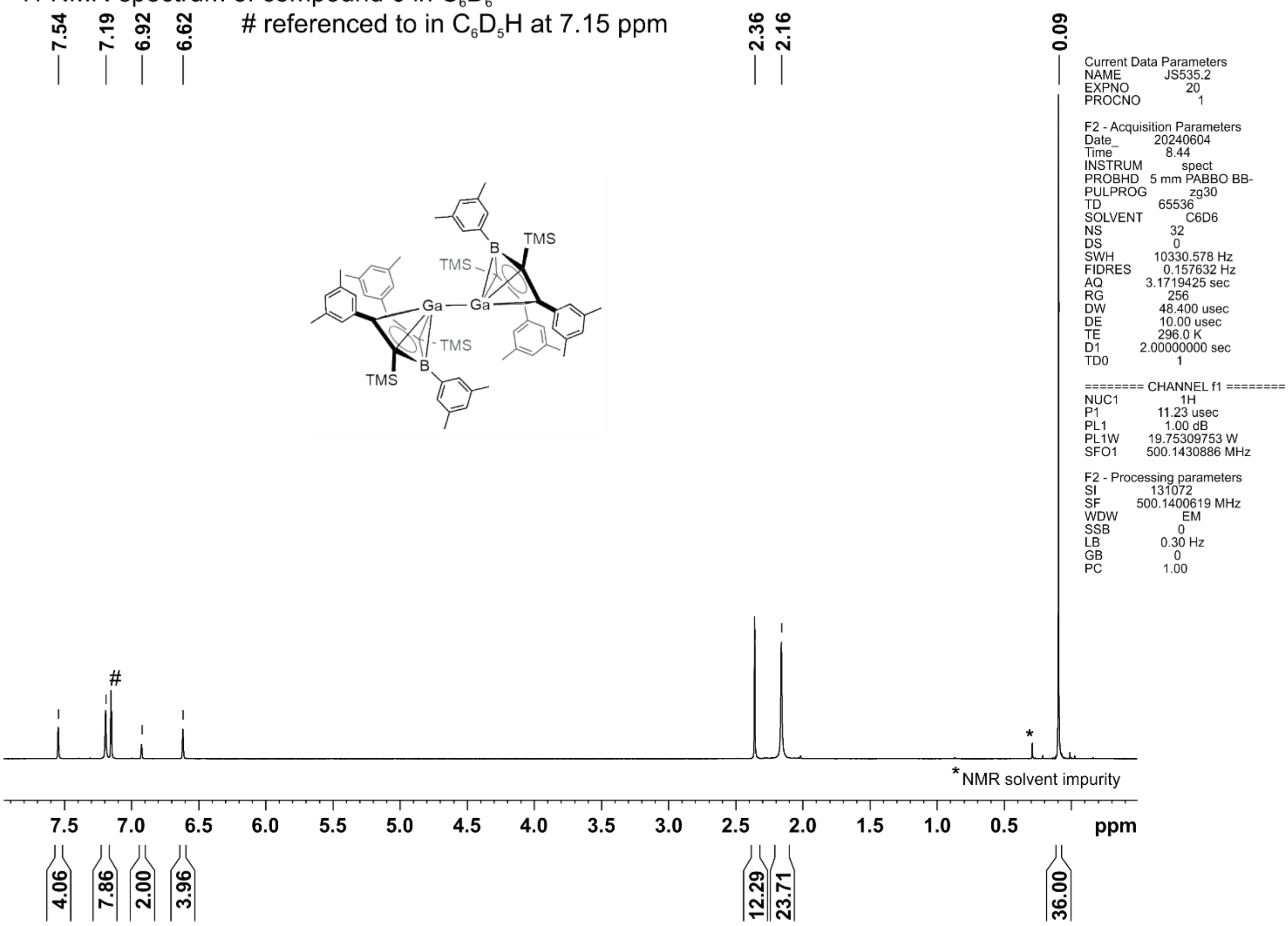

^{13}C{^{1}H}-NMR-spectrum of compound **6** in C_6D_6

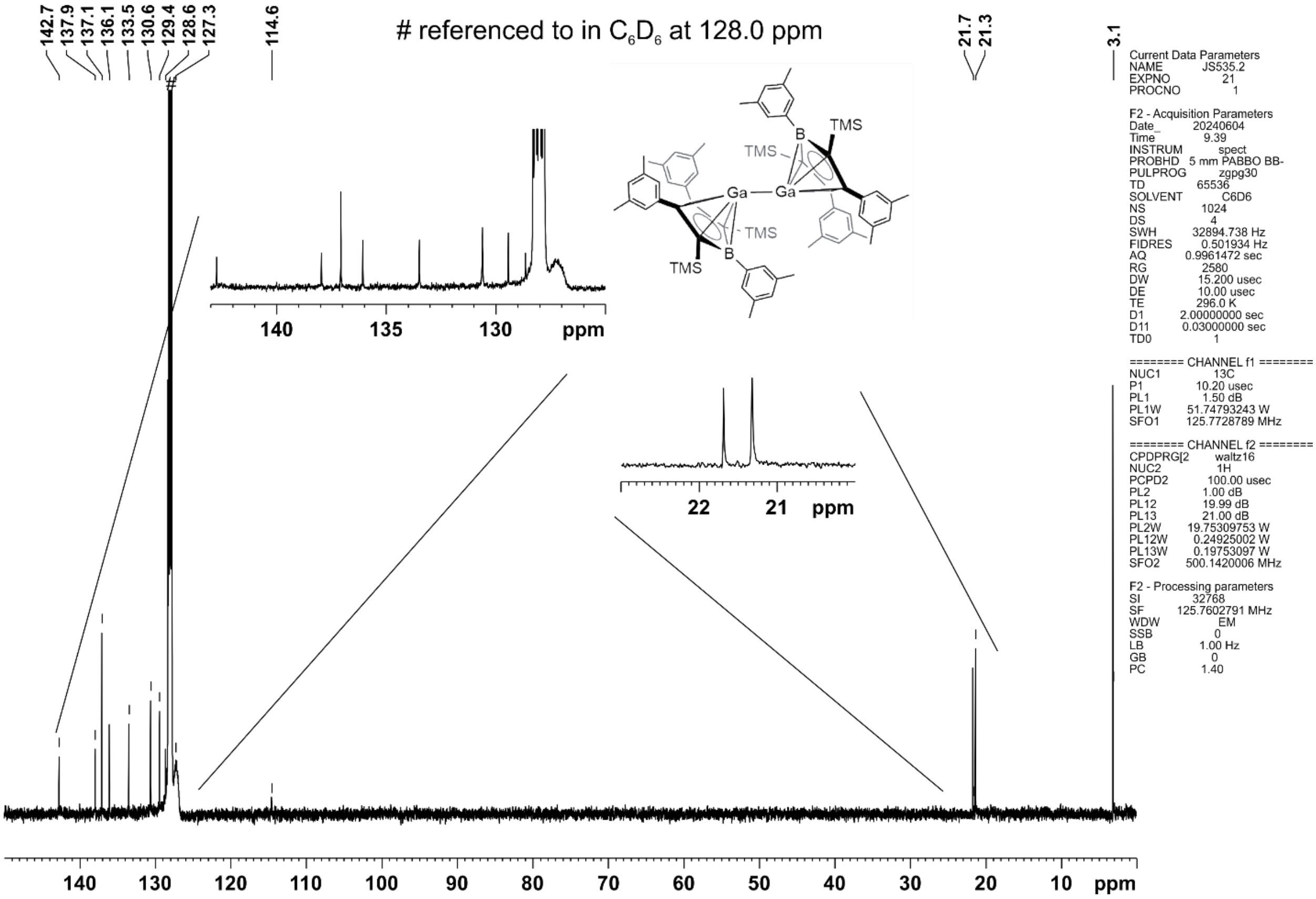

^{11}B-NMR-spectrum (background suppressed) of compound **6** in C_6D_6

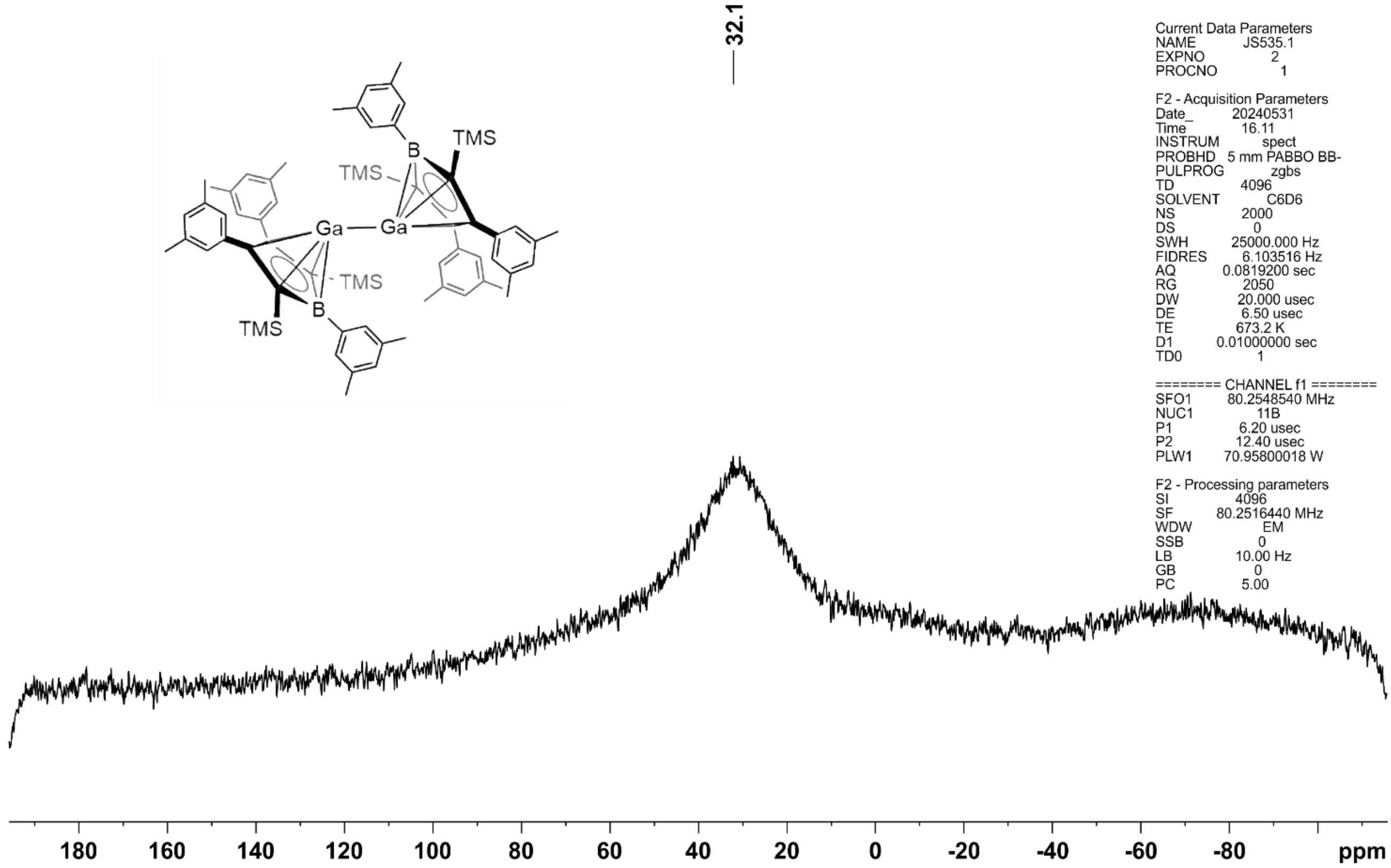

^{29}Si-DEPT-NMR-spectrum of compound **6** in C_6D_6

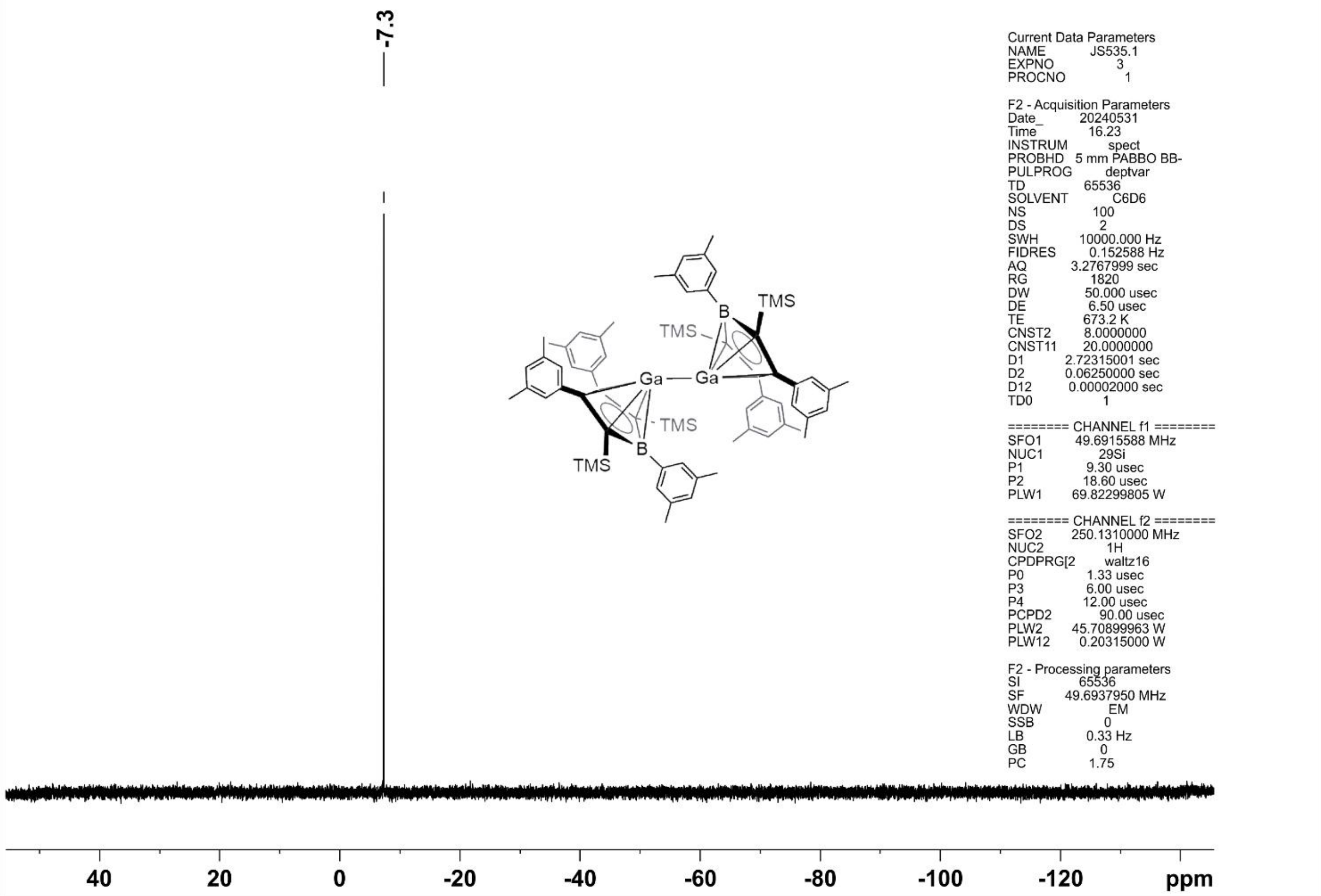

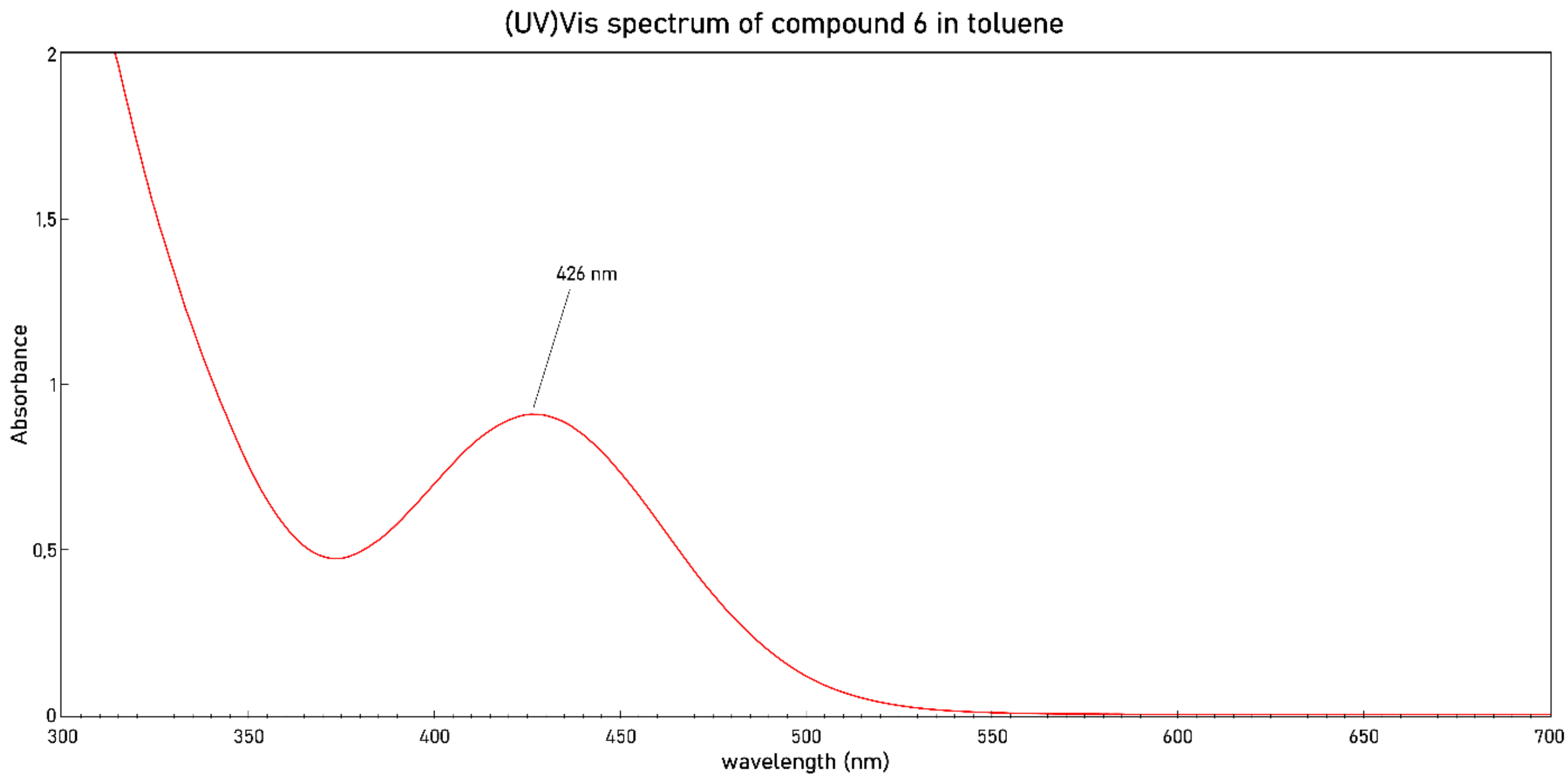
(UV)Vis spectrum of compound 6 in toluene
426 nm
Absorbance
wavelength (nm)
2
1,5
1
0,5
0
300
350
400
450
500
550
600
650
700

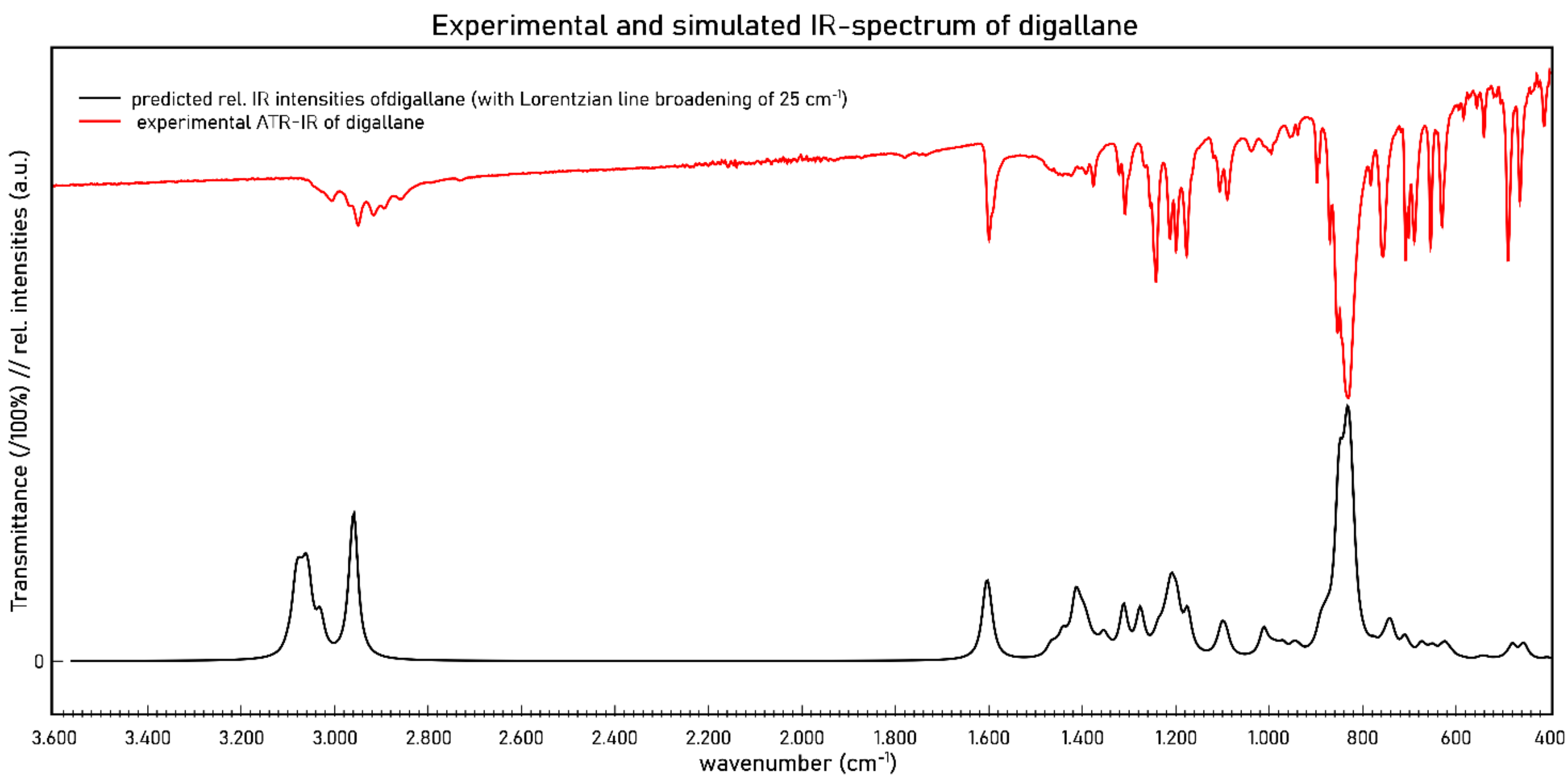
Experimental and simulated IR-spectrum of digallane
predicted rel. IR intensities ofdigallane (with Lorentzian line broadening of 25 cm⁻¹)
experimental ATR-IR of digallane
Transmittance (/100%) // rel. intensities (a.u.)
wavenumber (cm⁻¹)
0
3.600
3.400
3.200
3.000
2.800
2.600
2.400
2.200
2.000
1.800
1.600
1.400
1.200
1.000
800
600
400

Compound 7

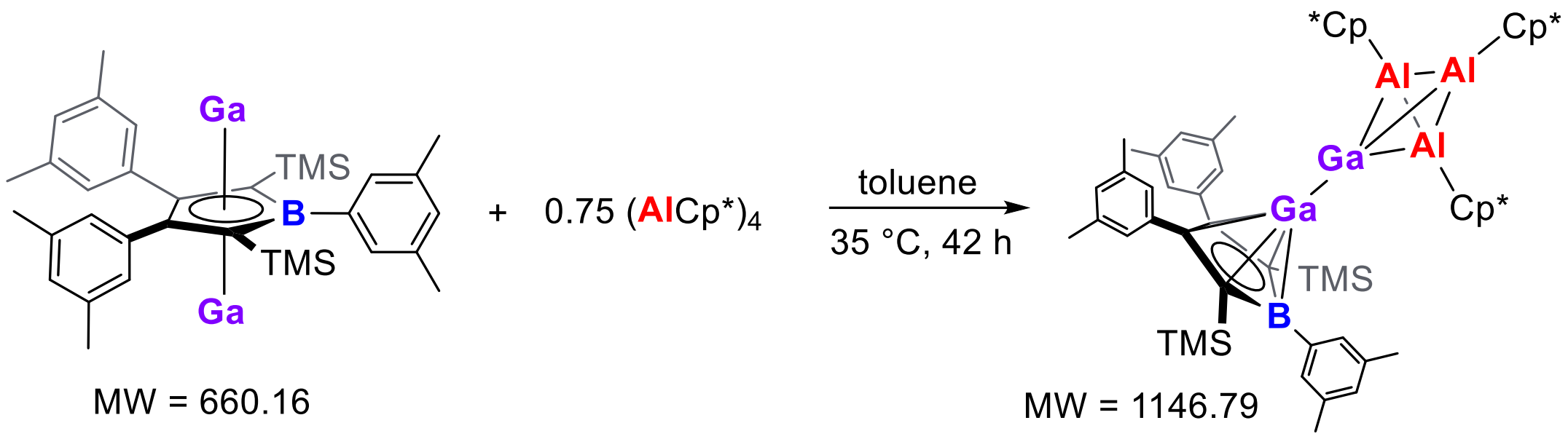

In a glovebox, a vial was charged with digallium cluster **1** (202.1 mg, 0.3061 mmol, 1 eq.) and $(AlCp^*)_4$ (199.0 mg, 0.3057 mmol, 1 eq.). The two solids were suspended in toluene (11 mL) and the mixture was left to stir for 42 h at 35 °C. The red suspension was filtered through a syringe filter equipped with a glass fibre pad (Whatman GF/B) to give a dark red filtrate and a slightly yellowish residue. The solvent was removed under reduced pressure from the filtrate to give a mainly red crystalline solid. The solid was carefully washed with diethyl ether (3 × 2 mL) and pentane (2 × 3 mL) effectively by suspending the solid in the solvent, followed by centrifugation in a mini-centrifuge and decanting the supernatant washing phases . After drying *in vacuo*, the *conjuncto*-cluster compound **7** (191.5 mg, 1.687 mmol, 55 %) was obtained as a red powder (191.5 mg, 1.687 mmol, 55 %). Orange crystals suitable for X-ray diffraction were obtained from solutions in diethylether.

Note: The compound **7** is only sparingly soluble in diethyl ether which allows the removal of residual starting materials by washing, in particular $(AlCp^*)_4$. The compound **7** is stable as a solid but in solutions partial decomposition into the starting materials was observed within the first hours until a stable equilibrium mixture of approximately 80:20 (product : starting material) is reached after four days. The digallium cluster can be optically observed as a colorless impurity before the washing step with diethylether.

Analytical Data for Compound 7

NMR:

^{1}H (700.36 MHz, 298 K, C_6D_6, C_6D_5H at 7.15 ppm): 7.64 (s, 2H, B-Xy: *o-H*), 7.21 (s, 4H, *o-H*), 6.97 (s, 1H, B-Xy: *p-H*), 6.66 (s, 2H, *p-H*), 2.48 (s, 6H, B-Xy: Ar-CH_3), 2.22 (s, 12H, Ar-CH_3), 1.90 (s, 45H CH_3-Cp), 0.24 (s, 18H, TMS).

^{13}C{^{1}H} (100.62 MHz, 298 K, C_6D_6, solvent signal at 128.0 ppm): 147.7 (B-Xy: *ipso-C$_{ar}$*) 136.5 (C_β), 135.6 (*m-C$_{ar}$*), 134.7 (B-Xy: *m-C$_{ar}$*), 134.2 (B-Xy: *o-C$_{ar}$*), 131.1 (*o-C$_{ar}$*), 129.3 (*ipso-C$_{ar}$*), 127.7 (*p-C$_{ar}$*), 127.0 (B-Xy: *p-C$_{ar}$*), 114.9 (Cp*-ring), 108.5 (C_α), 22.0 (B-Xy: *m*-CH_3), 21.5 (*m*-CH_3), 11.1 (Cp- CH_3) 4.1 (TMS).

^{11}B (80.25 MHz, 298 K, C_6D_6): 32.8 ($\omega_{1/2}$ = 628 Hz).

^{29}Si-DEPT (49.69 MHz, 298 K, C_6D_6): –9.3.

^{27}Al (65.17 MHz, 298 K, C_6D_6): –43.4 ($\omega_{1/2}$ = ca. 810 Hz)

^{71}Ga: no signal detected (screened from +300 to – 700 ppm)

Elemental Analysis: ($C_{64}H_{90}Al_3BGa_2Si_2$) calcd C 67.03, H 7.91; observed C 66.96, H 8.12.

UV-Vis: λ_{max} 485 nm (toluene).

Molecular structure of Compound 7

For further details on the diffraction measurement please see the respective section. Crystals suitable for X-ray analysis were grown from solutions in diethyl ether.

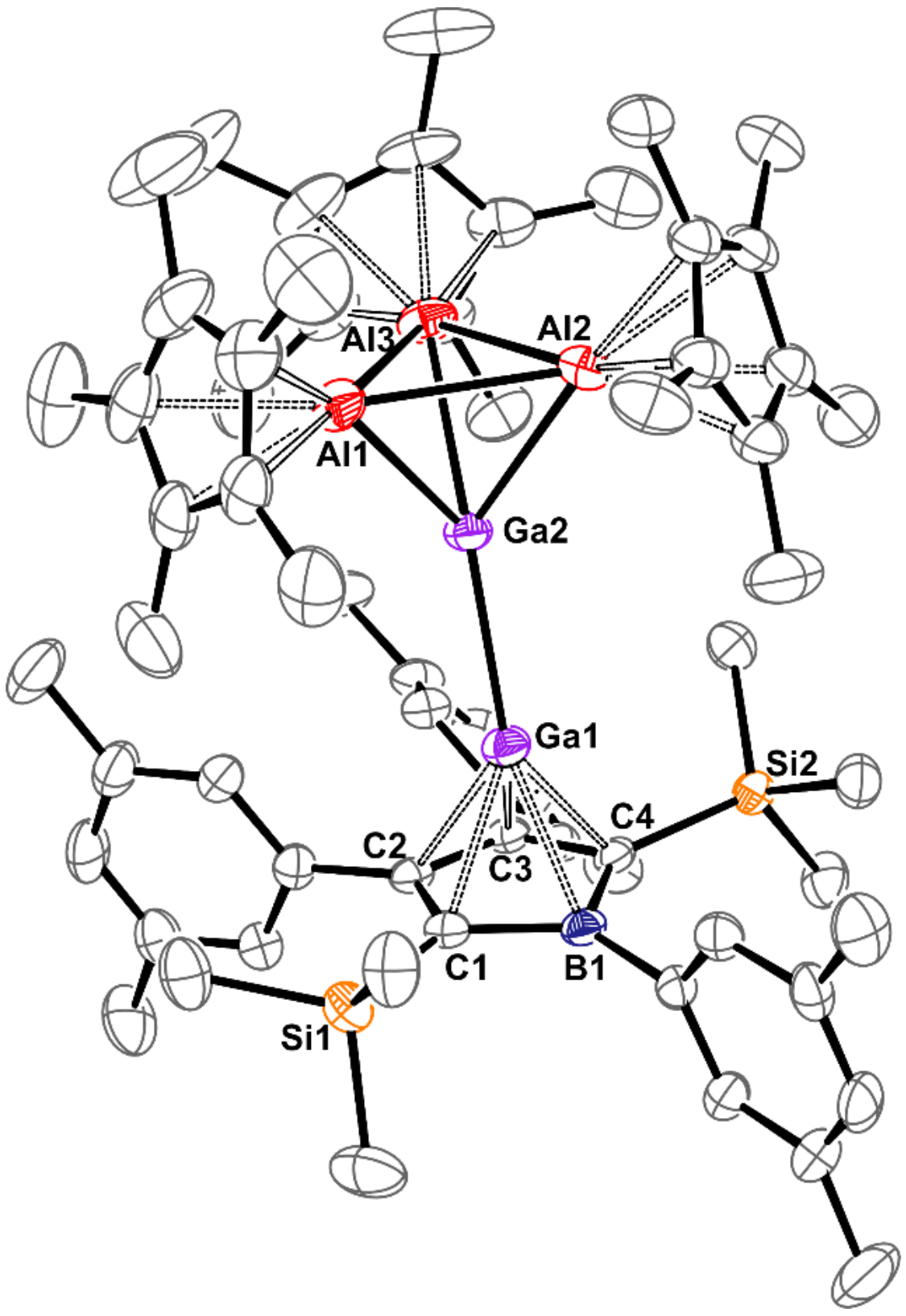

Figure SI12: ORTEP plot of the molecular structure of **7**. Atomic displacement parameters are drawn at 50% probability level. Hydrogen atoms and a lattice ether molecule are omitted for clarity. Selected bond lengths [Å] and angles [°] are given: Ga1–Ga2 2.5482(8), Ga2–Al1 2.554(1), Ga2–Al2 2.545(1), Ga2–Al3 2.577(1), Al1–Al2 2.760(1), Al2–Al3 2.749(1), Al3–Al1 2.764(1), B1–C1 1.553(4), C1–C2 1.451(4), C2–C3 1.429(3), C3–C4 1.455(4), C4–B1 1.549(4), B1–Ga1 2.240(3), C1–Ga1 2.192(2), C2–Ga1 2.254(3), C3–Ga1 2.280(3), C4–Ga1 2.214(3), Ctr_{BC4}–Ga1 1.844, Ctr_{BC4}-Ga1-Ga2 163.3°, B1-Ga1-Ga2 160.5(1)°. The structure was deposited with the CCSD.

Spectra Plots for Compound 7

^{1}H-NMR-spectrum of compound **7** in C_6D_6

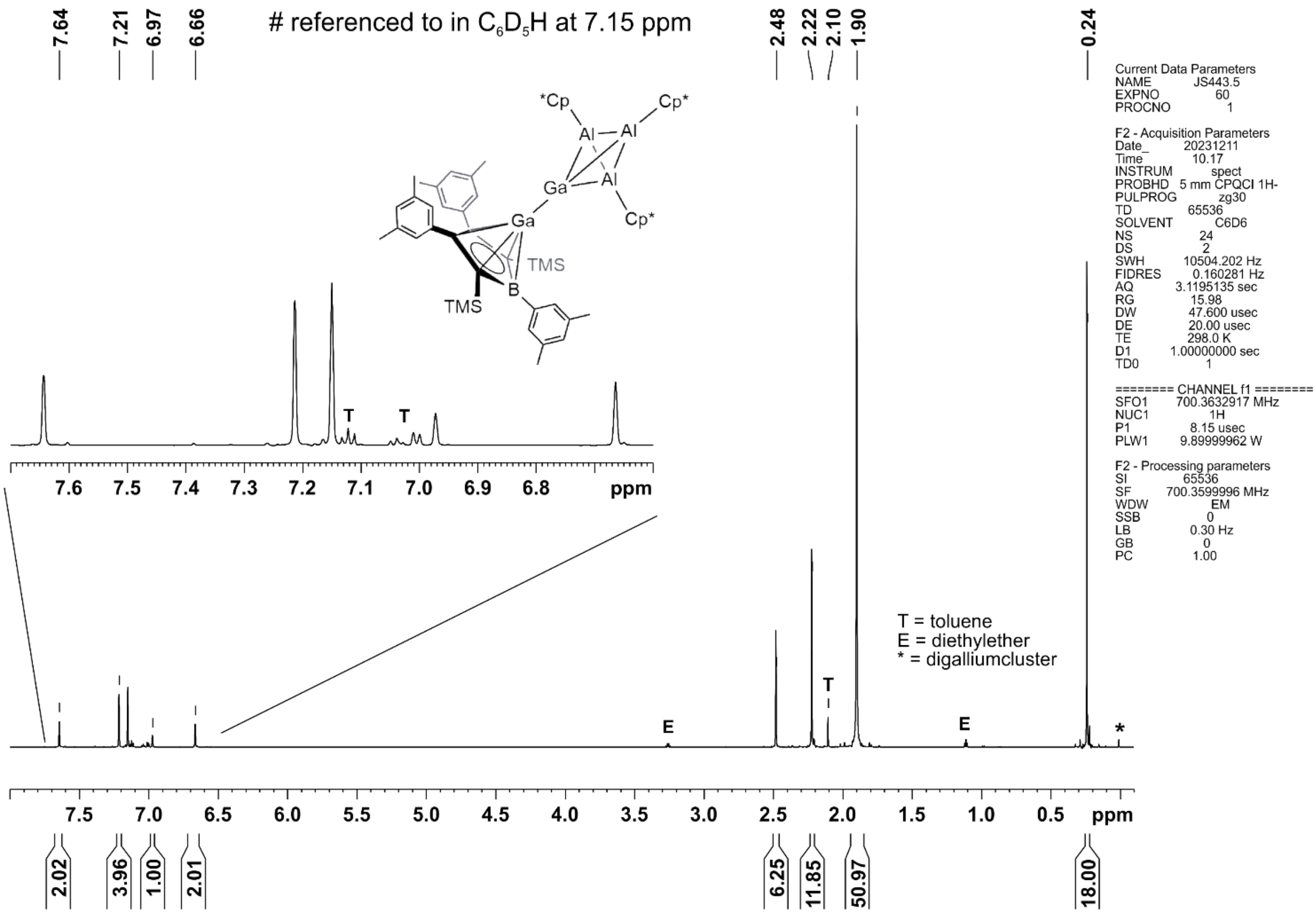

^{13}C{^{1}H}-NMR-spectrum of compound **7** in C_6D_6

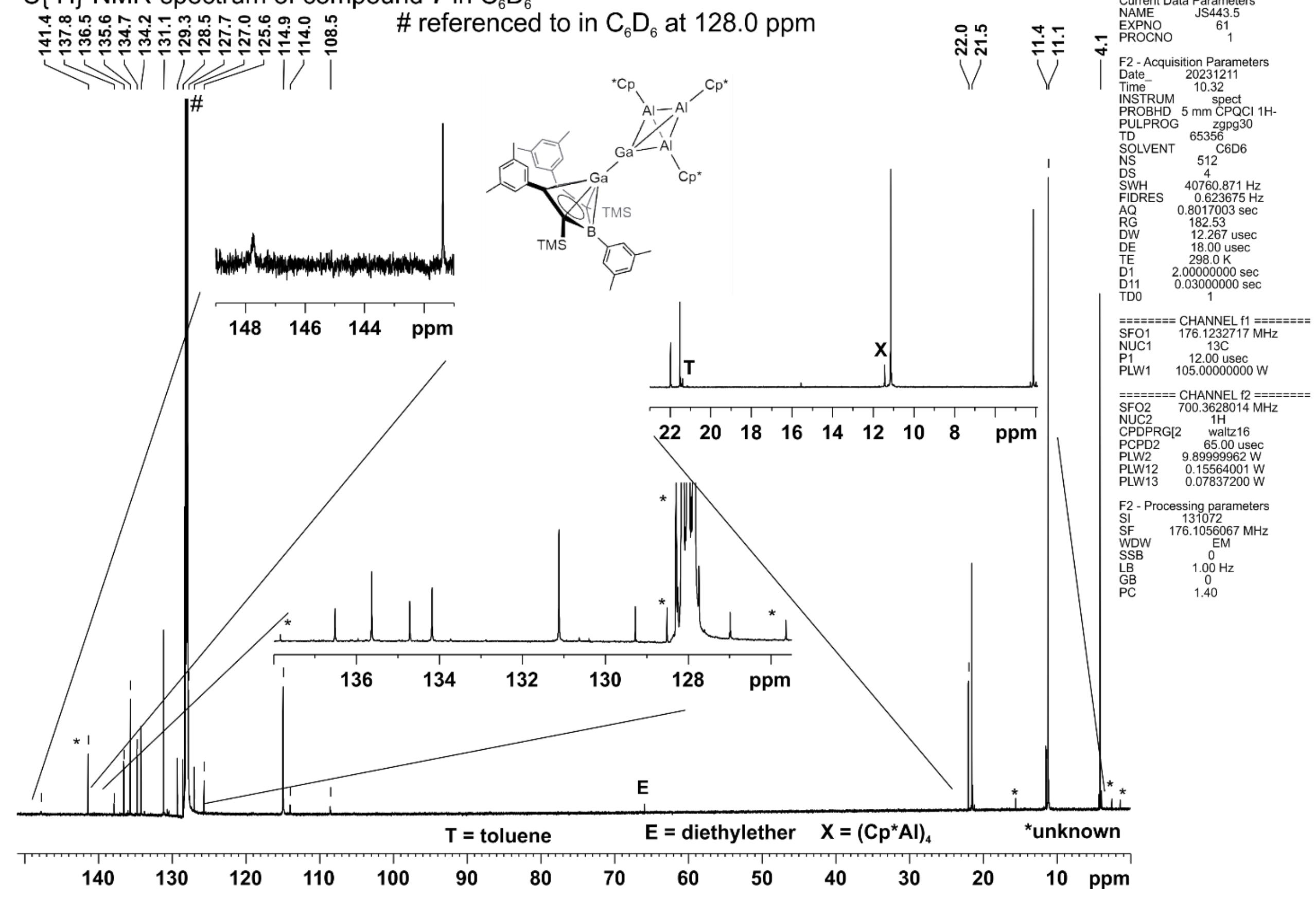

^{11}B-NMR-spectrum (background suppressed) of compound **7** in C_6D_6

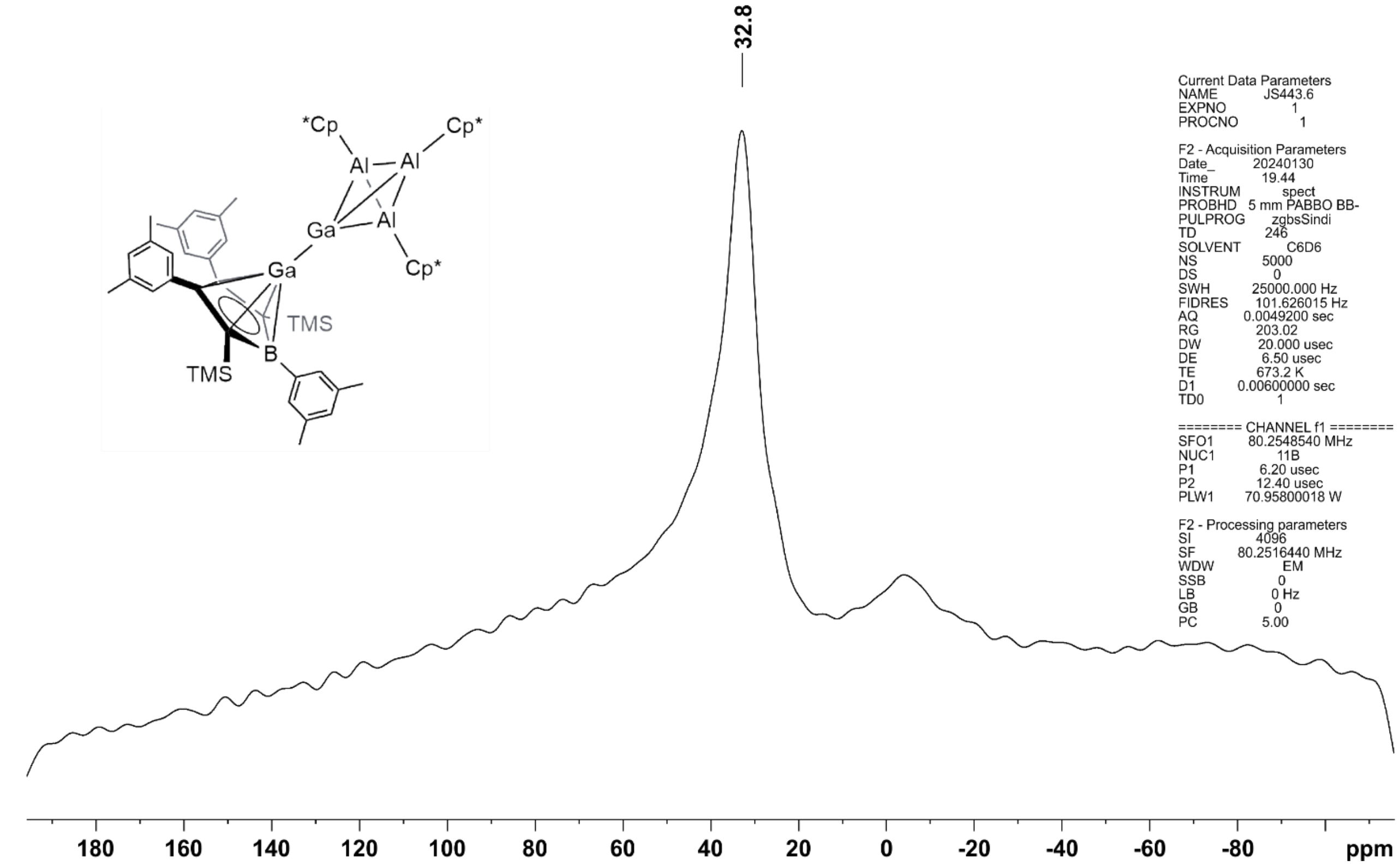

^{27}Al-NMR-spectrum of compound **7** in C_6D_6

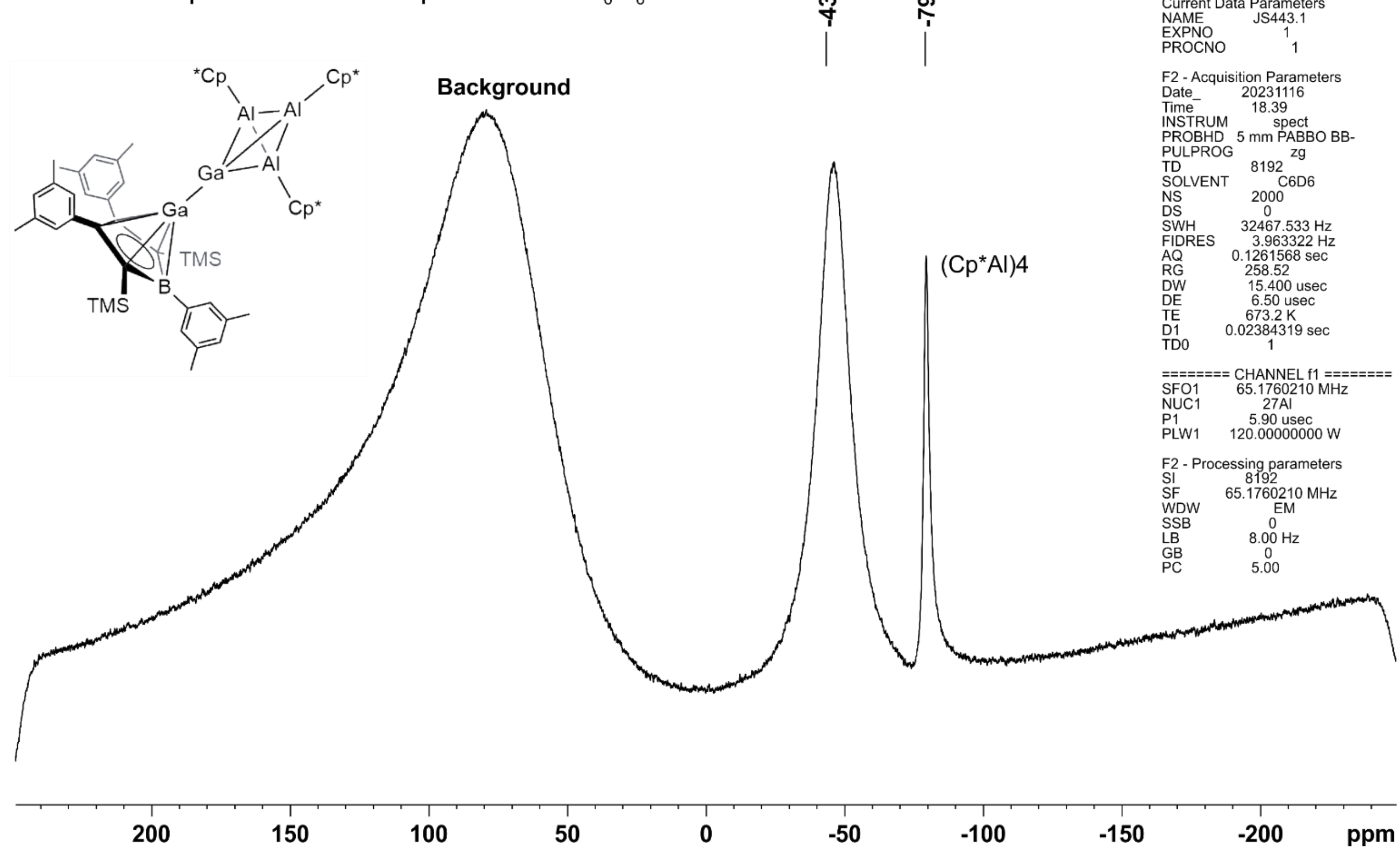

^{29}Si-DEPT-NMR-spectrum of compound **7** in C_6D_6

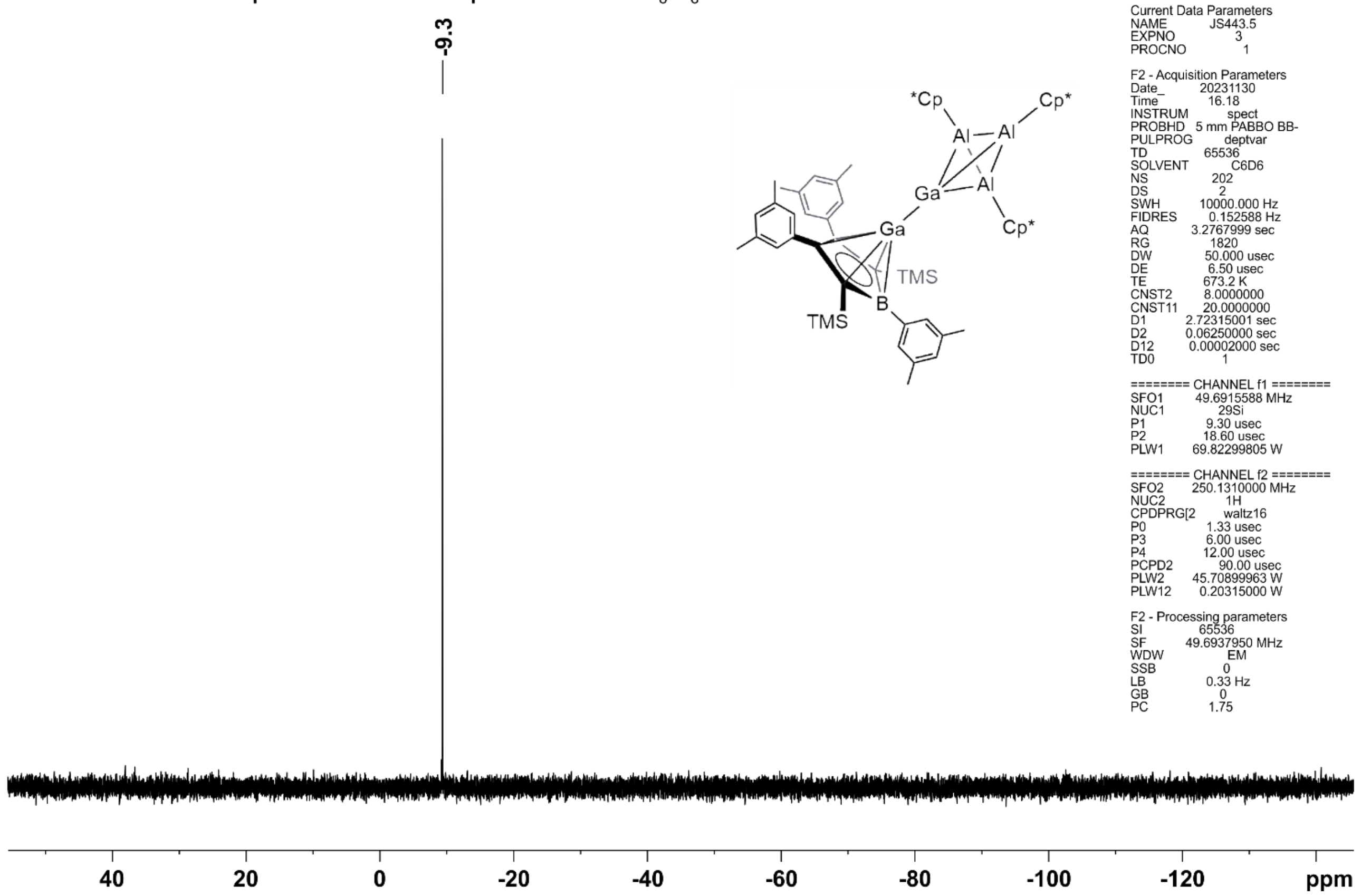

(UV)Vis spectrum of compound 7 in toluene

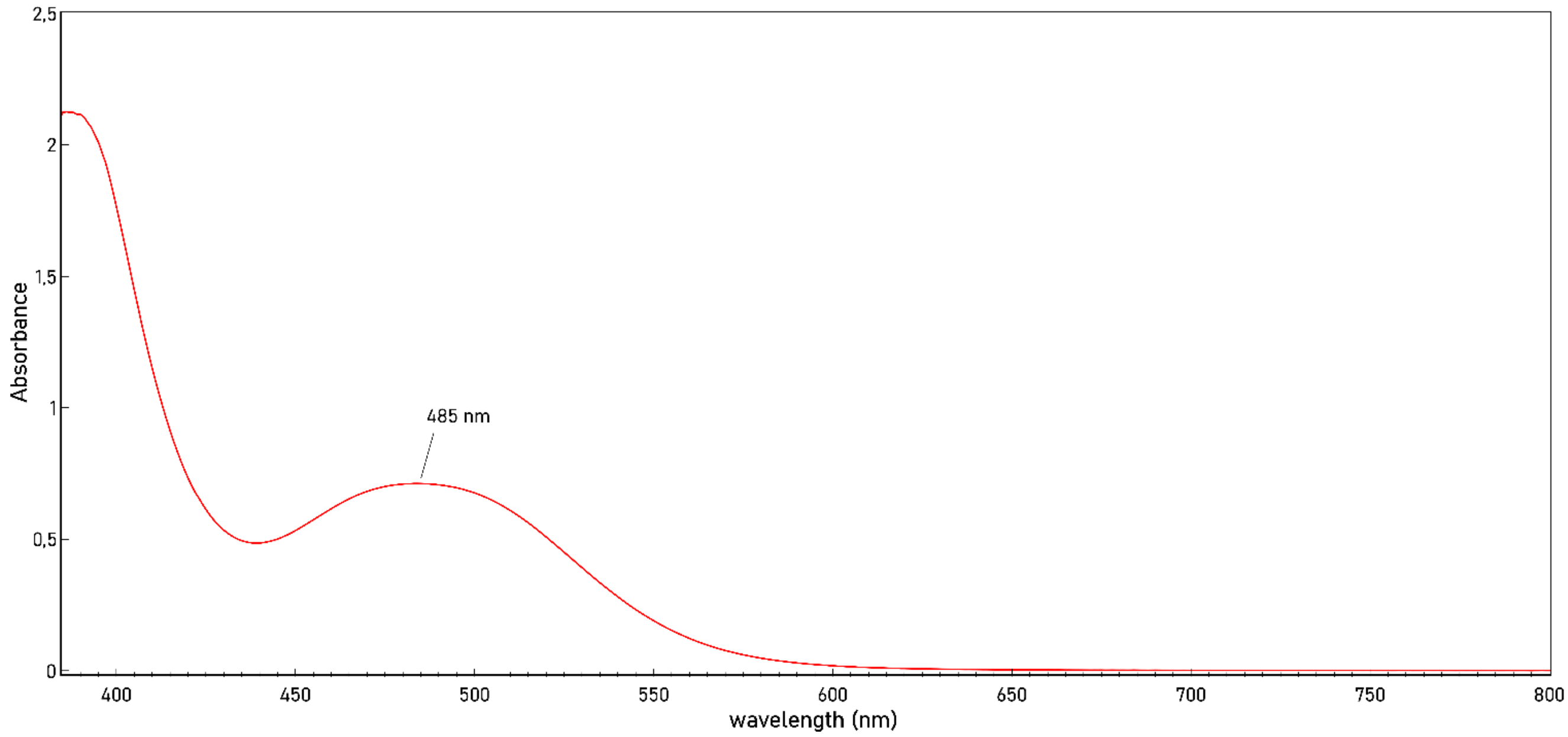

Compound 8

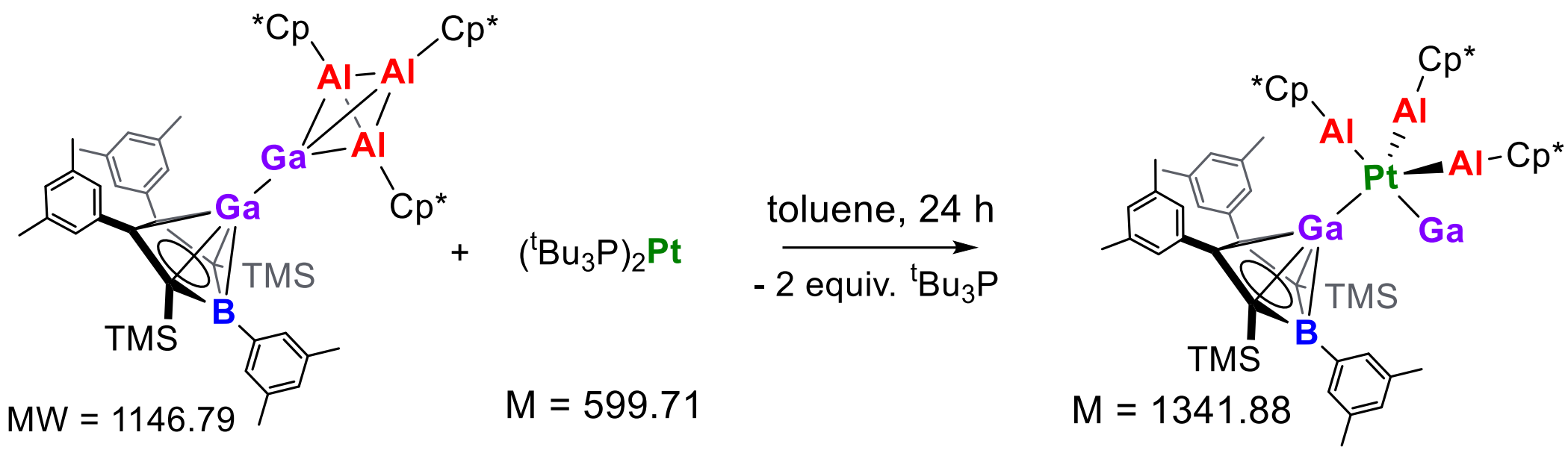

In a glovebox, compound **7** (55.0 mg, 0.0436 mmol, 1 eq.) was added portion-wise in 10 min intervals as a solid to a stirred solution of bis(tri-*tert*-butylphosphine)platinum (34.9 mg, 0.0581 mmol, 1.33 eq) in toluene (1.5 mL). The reddish colored solution was left to stir over night at ambient temperature. The solvent was removed under reduced pressure, hexane (2 mL) was added and again evaporated in *vacuo* to remove left over toluene by co-evaporation. The orange to beige residue was washed with pentane (3 × 2 mL). This was most efficiently achieved by suspending the solid in pentane, centrifugation with a mini centrifuge and decanting the washing phase with a syringe. After drying the solid residue under reduced pressure, the plating complex **8** (39.9 mg, 0.0297 mmol, 68 %) was obtained as a beige to ochre powder.

Note: The gallium compound **7** was added portion-wise in intervals to ensure a clean reaction. In solution, the gallium compound **7** partially decomposes in its starting materials (digallium cluster **1** and (Cp*Al)$_4$) and forms an equilibrium in solution. We observed, that the digallium cluster **1** reacts unselectively with most platinum(0) sources (including bis(tri-*tert*-butylphosphine)platinum) to a series of yet unidentified compounds which are very difficult to separate from **8**. In order to reduce this side reaction, strategies reducing the extent of digallium cluster **1** formation from solutions of **7** are to be preferred.

Analytical Data for Compound 8

NMR:

^{1}H (500.14 MHz, 296 K, C_6D_6, C_6D_5H at 7.15 ppm): 7.80 (s, 2H, B-Xy: *o-H*), 7.45 (s, 4H, *o-H*), 6.99 (s, 1H, B-Xy: *p-H*), 6.69 (s, 2H, *p-H*), 2.52 (s, 6H, B-Xy: Ar-C*H$_3$*), 2.21 (s, 12H, Ar-C*H$_3$*), 1.93 (s, 45H, Cp*), 0.27 (s, 18H, TMS).

^{13}C{^{1}H} (125.77 MHz, 296 K, C_6D_6, solvent signal at 128.0 ppm): 142.4 (*ipso-*C_{ar}), 139.3 (C_β), 135.4 (*m-*C_{ar}), 134.8 (B-Xy: *m-*C_{ar}), 134.6 (B-Xy: *o-*C_{ar}), 132.0 (*o-*C_{ar}), 127.4 (B-Xy: *ipso-*C_{ar}, broad & partly superimposed), 127.1 & 127.2 (*p-*C_{ar} + B-Xy: *p-*C_{ar}, superimposed), 114.7 (Cp*-ring) 108.7 (C_α, only in 2D spectra), 22.1 (B-Xy: *m-*CH_3), 21.6 (*m-*CH_3), 10.9 (Cp*-CH_3) 5.1 (TMS).

^{11}B (80.25 MHz, 298 K, C_6D_6): 29.6 ($\omega_{1/2}$ = 1017 Hz).

^{29}Si-DEPT (49.69 MHz, 298 K, C_6D_6): –10.9.

^{27}Al: no signal detected (screened from +100 to – 100 ppm)

^{71}Ga: no signal detected (screened from +300 to – 700 ppm)

Elemental Analysis: ($C_{64}H_{90}BAl_3Ga_2Si_2Pt$) calcd C 57.29, H 6.76; observed C 56.87, H 6.75.

Molecular structure of Compound 8

For further details on the diffraction measurement please see the respective section. Crystals suitable for X-ray analysis were grown from solutions in toluene at –40°C.

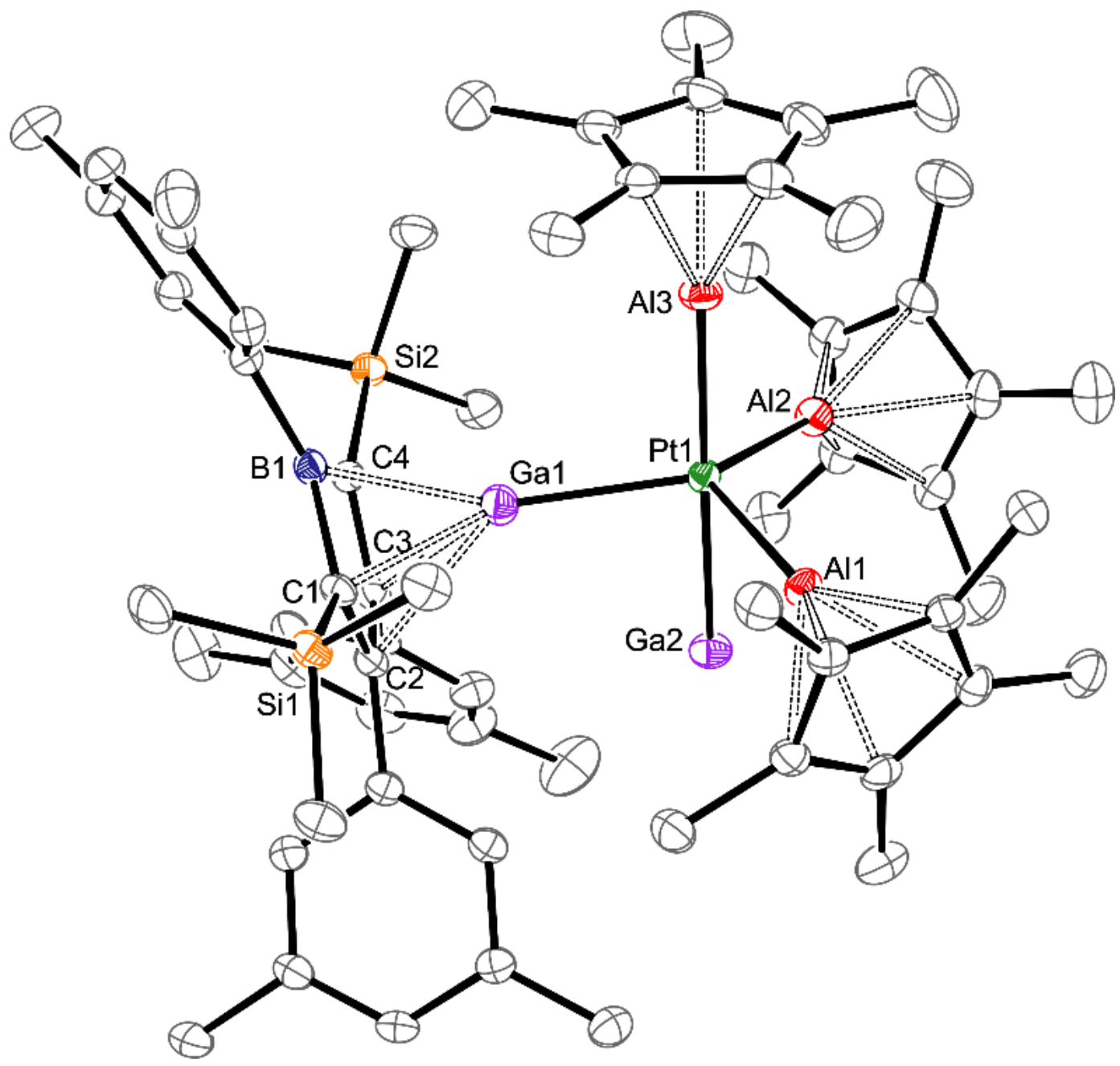

Figure SI13: ORTEP plot of the molecular structure of **8**. A second molecule in the asymmetric unit, hydrogen atoms and co-crystallised toluene molecules are omitted for clarity. Atomic displacement parameters shown at 50% probability. Selected bond lengths [Å] and angles [°] are given. Ga1–Pt1 2.3990(7), Ga2–Pt1 2.4680(6), Al1–Pt1 2.3083(8), Al2–Pt1 2.316(1), Al3–Pt1 2.361(1), B1–C1 1.568(4), C1–C2 1.467(4), C2–C3 1.421(3), C3–C4 1.458(4), C4–B1 1.553(4), B1–Ga1 2.234(3), C1–Ga1 2.231(3), C2–Ga1 2.378(3), C3–Ga1 2.395(3), C4–Ga1 2.225(3), Ctr_{BC4}–Ga1 1.911; Ga1-Pt1-Ga2 83.36(2)°, Ga2-Pt1-Al3 176.13(3)°, Ga1-Pt1-Al1 116.00(3)°, Al1-Pt1-Al2 115.14(4)°, Al2-Pt1-Ga1 124.04°, Ctr_{BC4}-Ga1-Pt1 167.1°. The structure was deposited with the CCSD.

Spectra Plots for Compound 8

^{1}H-NMR-spectrum of compound **8** in C_6D_6

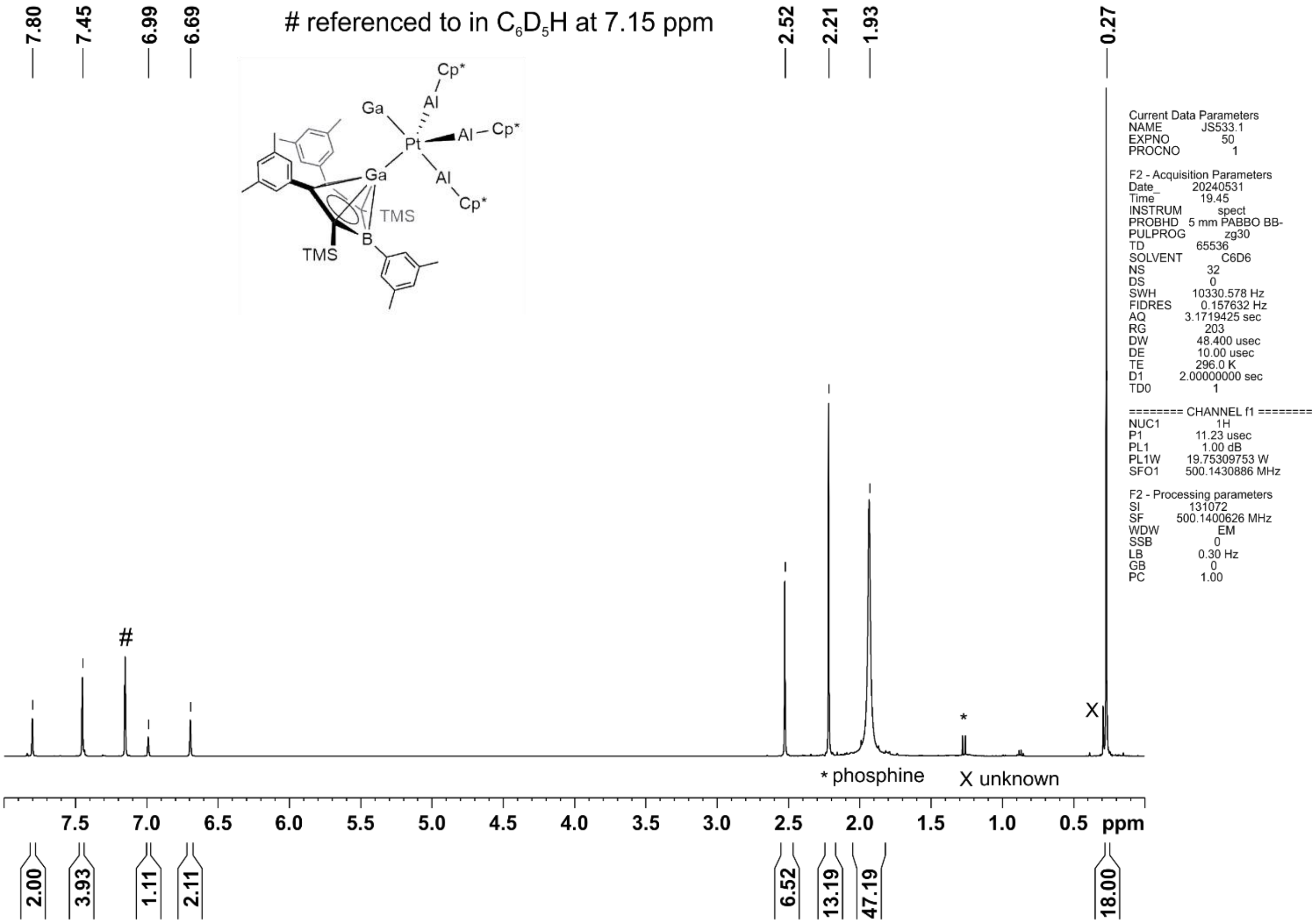

^{13}C{^{1}H}-NMR-spectrum of compound **8** in C_6D_6

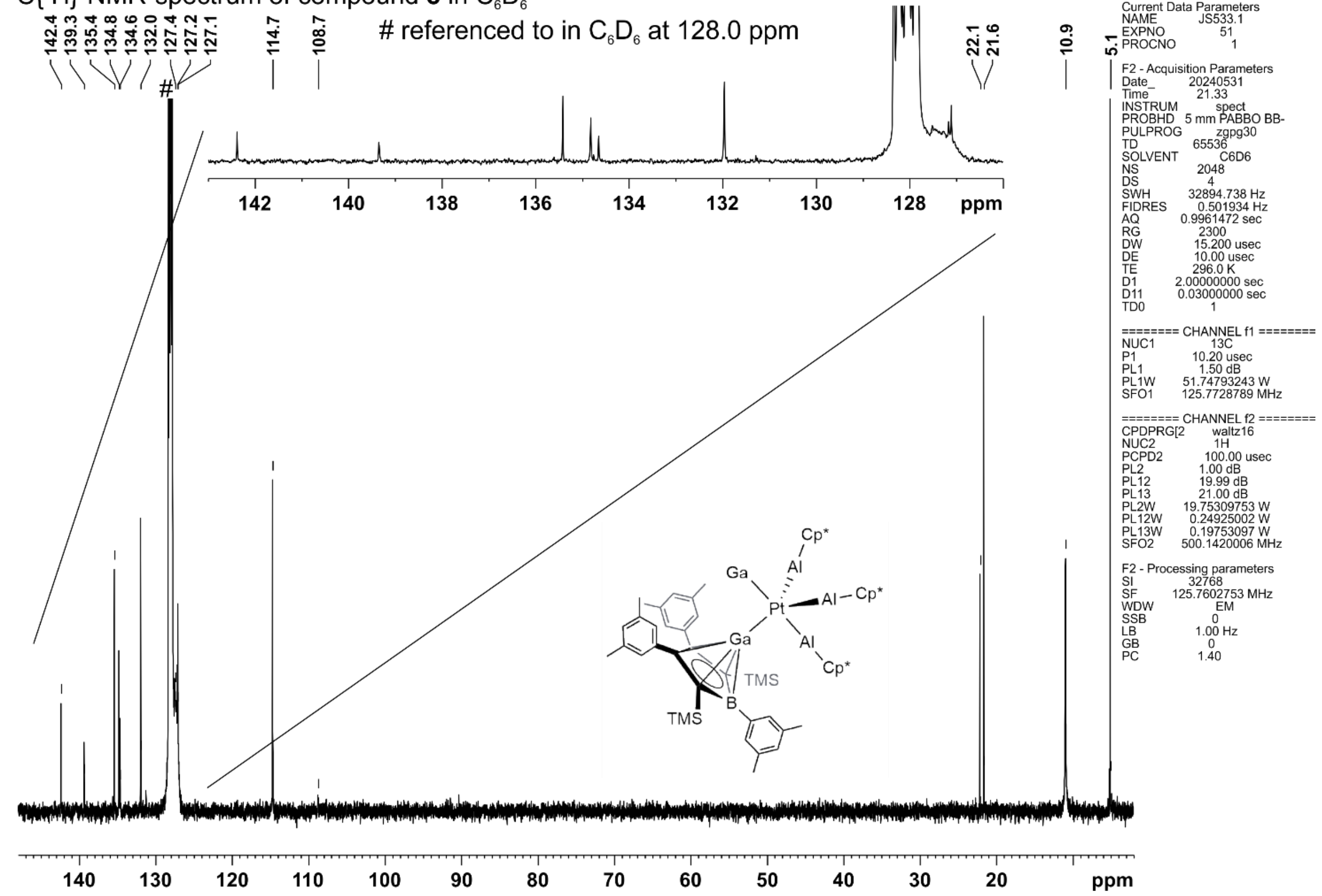

^{11}B-NMR-spectrum (background suppressed) of compound **8** in C_6D_6

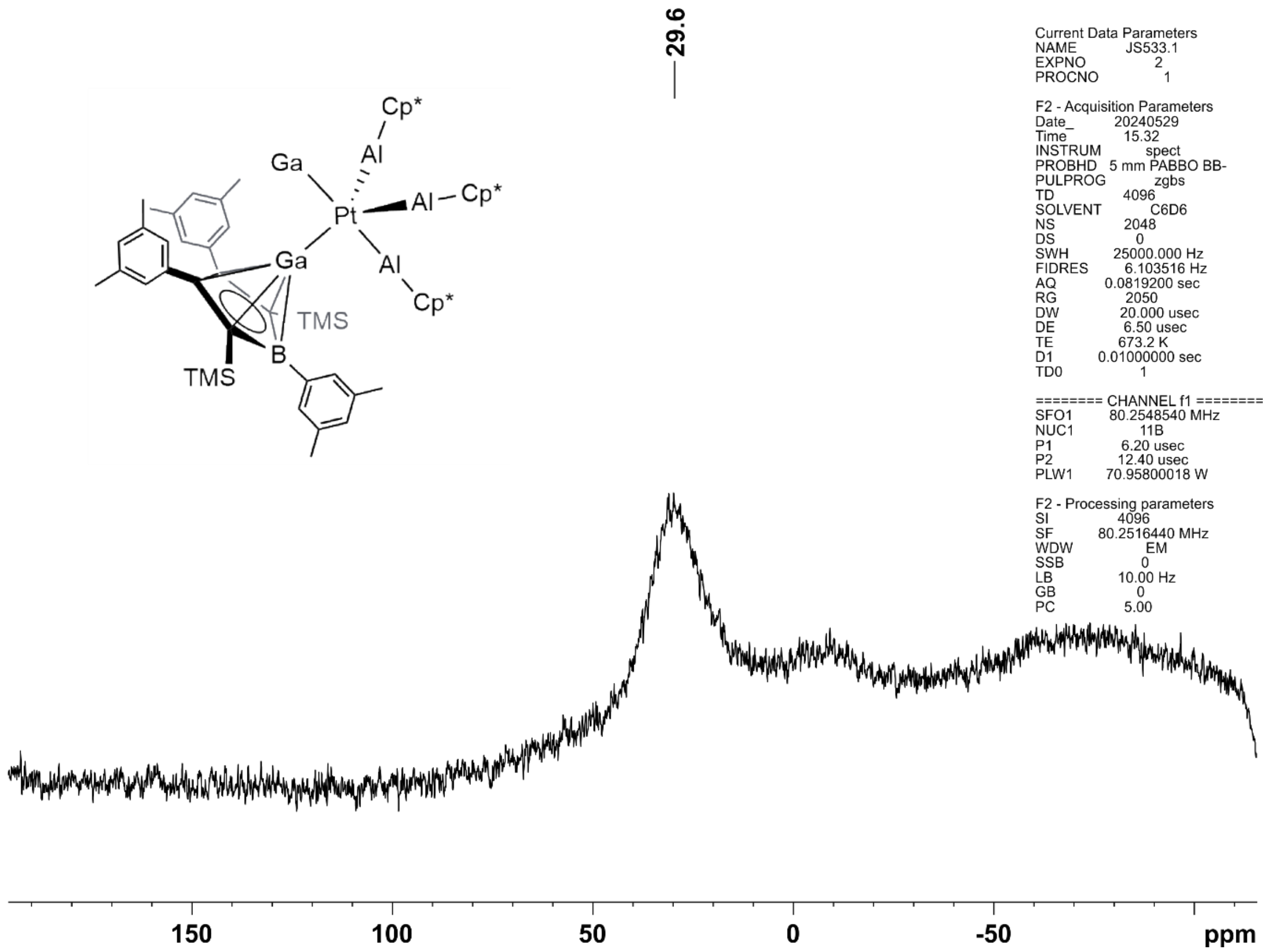

^{27}Al-NMR-spectrum of compound **8** in C_6D_6

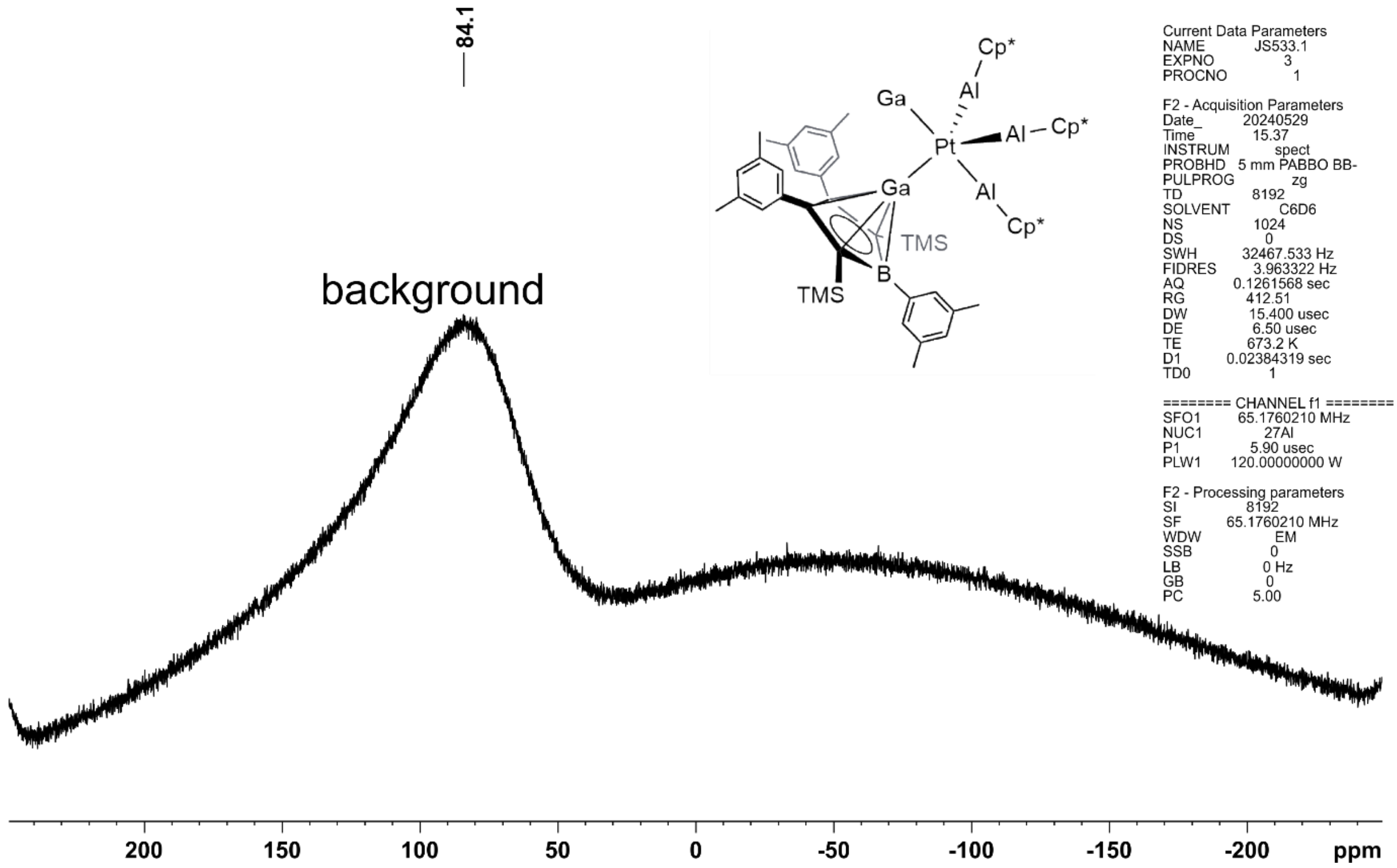

^{29}Si-DEPT-NMR-spectrum of compound **8** in C_6D_6

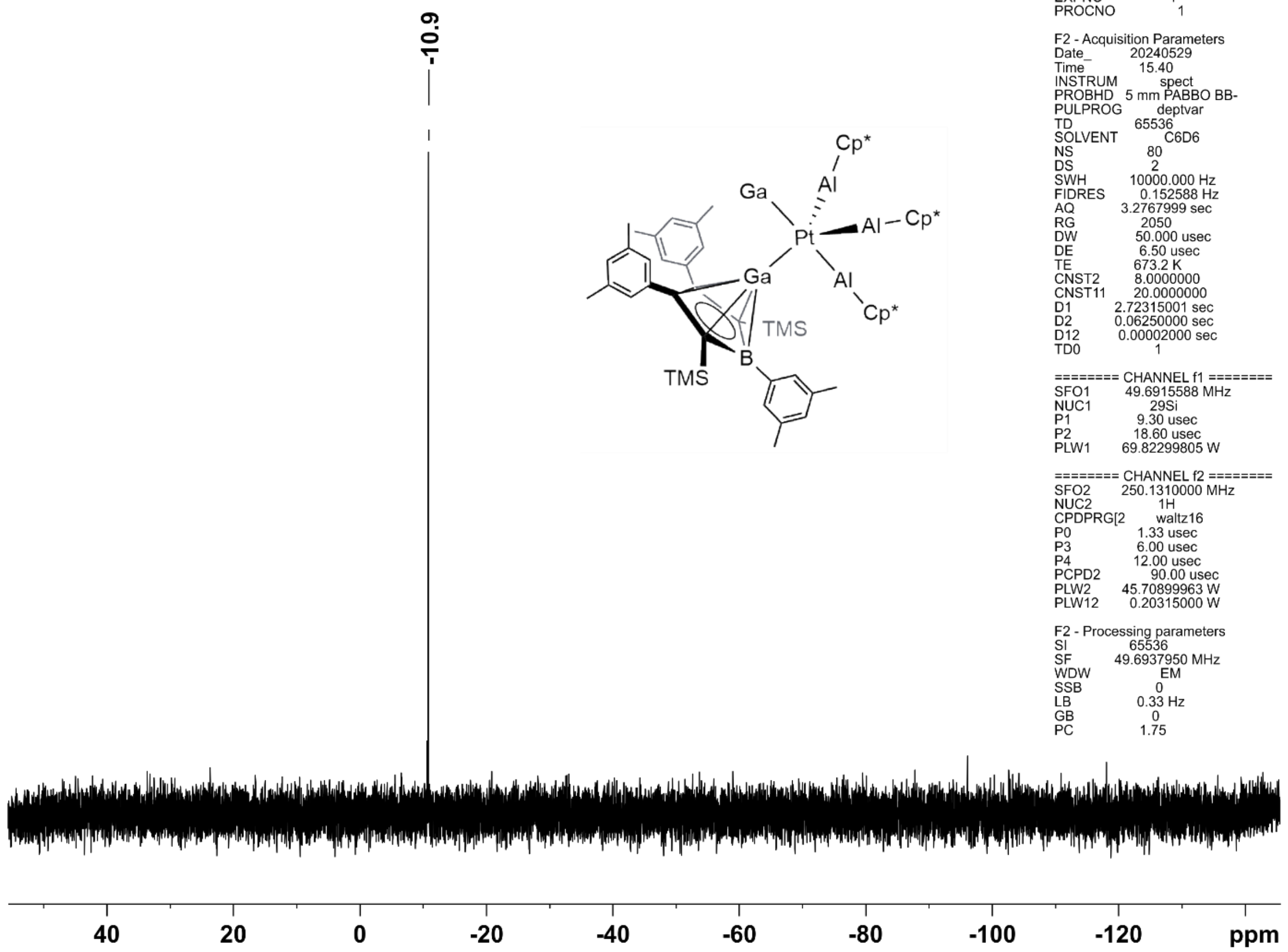

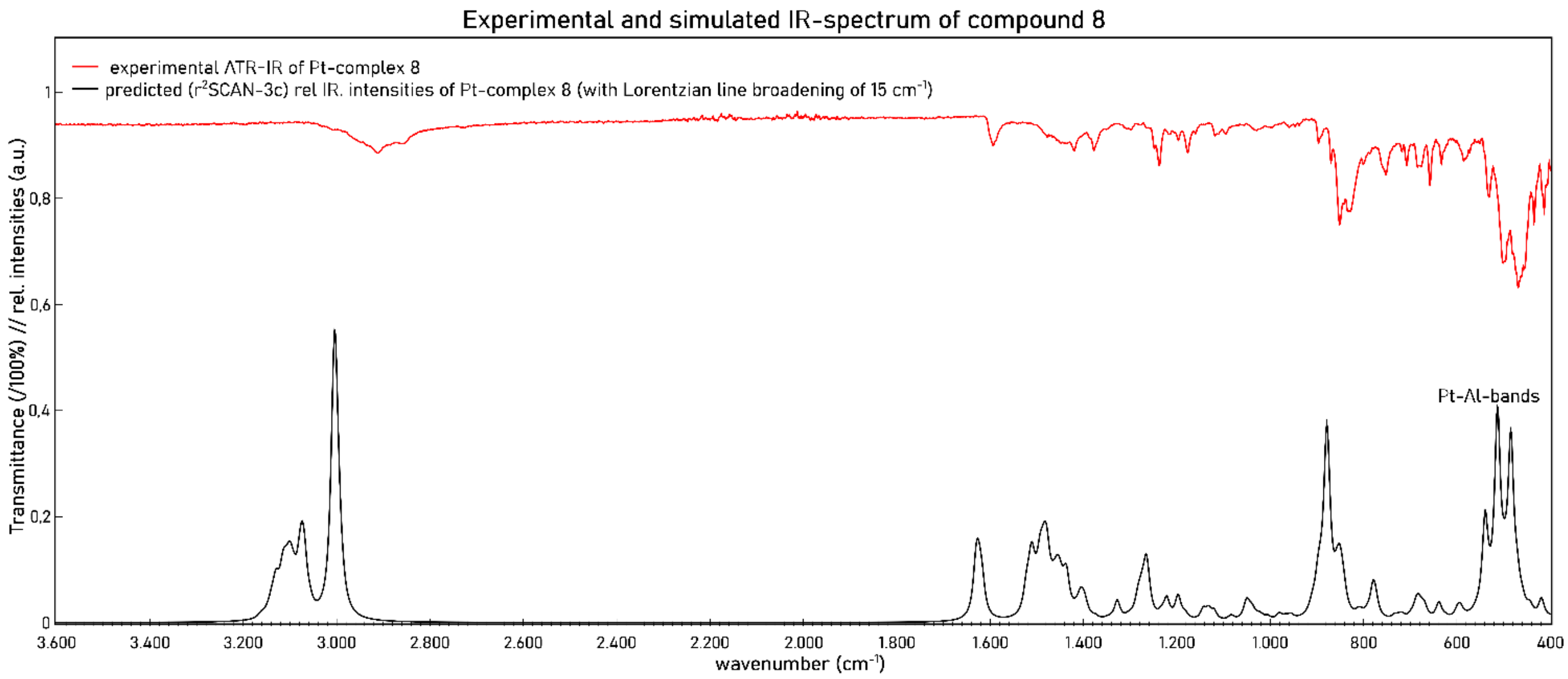

Crystallographic Details

General Data Acquisition and Processing

X-ray data were either collected on Bruker APEX II CCD diffractometers with a microfocus sealed tube of Mo Kα radiation or on a Rigaku XtaLAB Synergy-DW four-circle diffractometer with Mo and/or Cu Kα radiation using a mirror as monochromator and a HyPix-Arc 100 detector or on a STOE STADIVARI Eulerian 4-circle diffractometer with a Mo-Genix source using a Graded multilayer mirror as monochromator and a DECTRIS PILATUS 200K detector. All diffractometers were equipped with an Oxford Cryosystems cryostream.

The Bruker data were integrated using SAINT implemented in Bruker's APEX3 programme suite.[8] SADABS was used for multi-scan absorption correction.[9]

The Rigaku data were processed using the Crysalis Pro software suite including Autochem and Olex2 integration.[10]

The STOE data were processed using the X-Area software including STOE LANA for absorption corrections.[11]

Structure solution was performed with SHELXT[12] and refined using SHELXL[13] along the graphical user interphase of ShelXle.[14] In some cases DSR has been applied to treat disordered solvent molecules.[15] . All hydrogen atoms were refined isotropic on calculated positions using a riding model with their U_{iso} values constrained to 1.5 times the U_{eq} of their pivot atoms for terminal sp^3 carbon atoms and 1.2 times for all other carbon atoms. Disordered moieties were refined using bond lengths restraints and displacement parameter restraints. Further details on the individual data sets are tabulated in the analytical section of each compound.

Crystallographic and Refinement Details A(Li(tol))$_2$

Due to mediocre crystal quality only a dataset to resolutions of 0.84 Å was recorded. A toluene molecule was found disordered over a special position and modelled in PART-1 using Kratzert's DSR tool making use of FLAT, SADI, SIMU and RIGU restraints.

Crystallographic and Refinement Details 2

The asymmetric unit (depicted) contained two full molecules of **2** with several ^{t}Bu groups of each molecule being disordered over two positions. The disorder was modelled accordingly using SAME/SADI, SIMU and RIGU commands. CHECKCIF indicates a low-coordinate metal atom as an A-level alert, in the crystal packing, no relevant intermolecular Ga---Ga contacts could be identified.

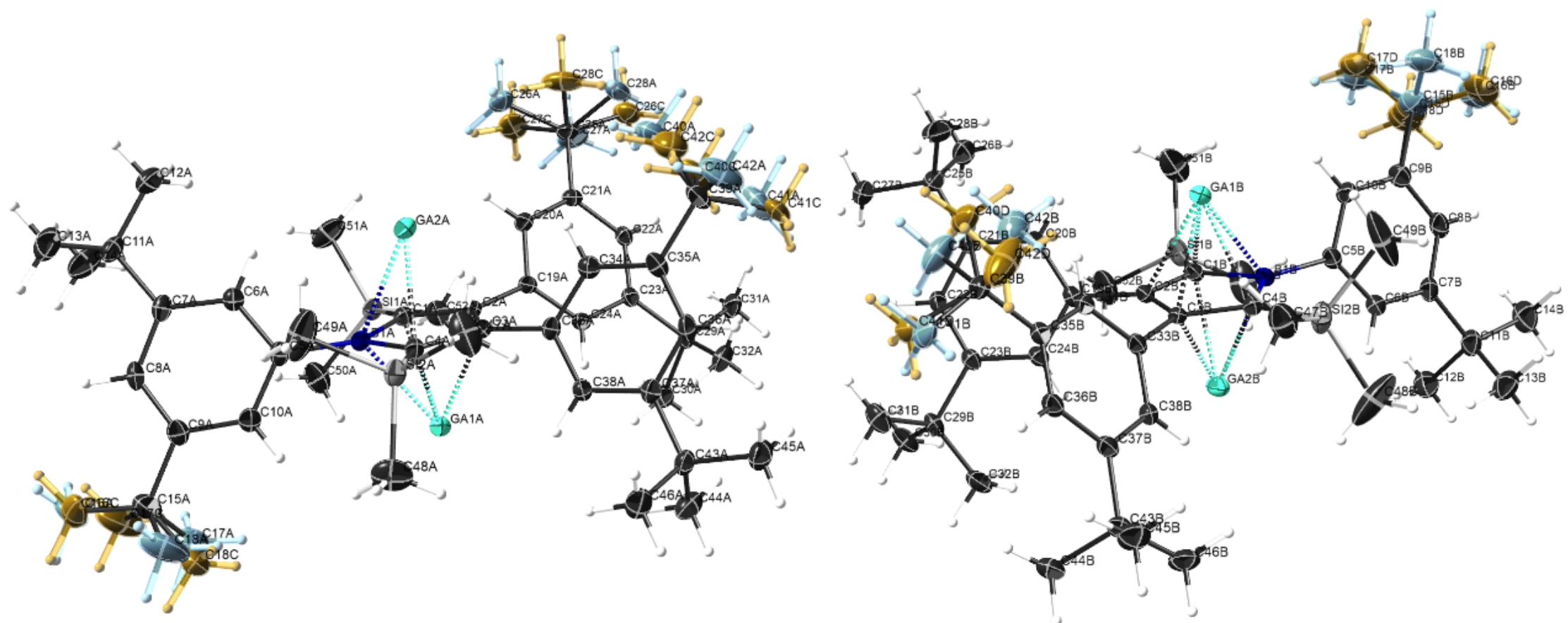

Figure SI14: Depiction of the asymmetric unit of **2** including the modelled disorder (PART1 blue, PART 2 orange)

Crystallographic and Refinement Details 3

Crystals of **3** were obtained at –40°C from hexane solutions. CHECKCIF indicates a low-coordinate metal atom as an A-level alert. Considering packing of the molecules very weak intermolecular Ga----Ga orientations can be identified with a Ga….Ga distance of 3.674 Å. This is slightly smaller than the sum of Van-der-Waals-Radii (2 × 1.87 Å = 3.74 Å) suggesting at best weak interactions.[16]

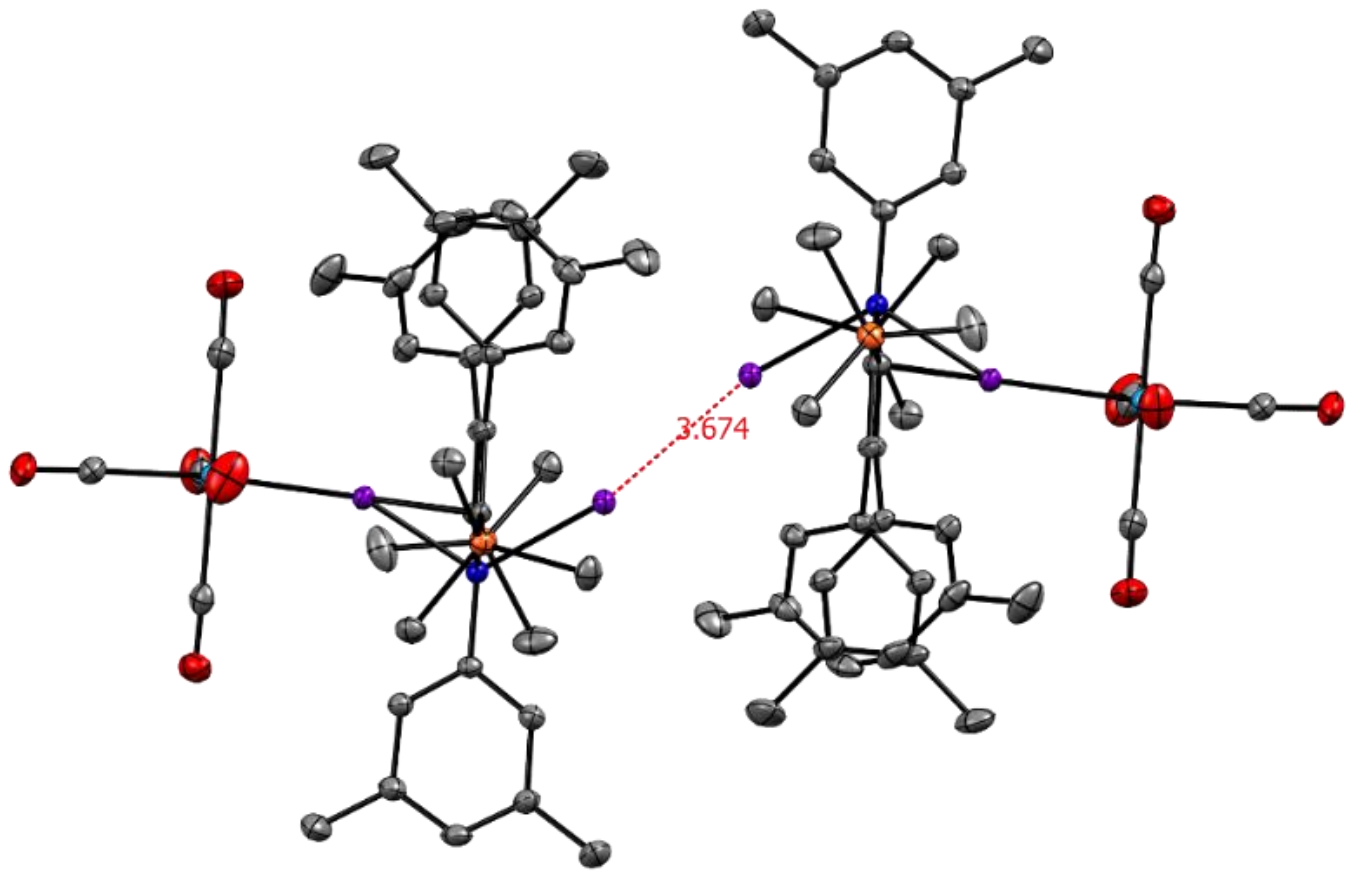

Figure SI15: Depiction of two molecules of **3** as an excerpt of the packing in the crystal with long intermolecular Ga….Ga distances indicated.

Crystallographic and Refinement Details 4

Crystals were grown from cold (–40°C) solutions in hexane/toluene. The dataset was refined as an inversion twin. The equatorial CO groups were restrained using DANG, SADI, SIMU and RIGU commands.

Crystallographic and Refinement Details 5

Crystals were grown from cold (–40°C) solutions in pentanes. The structure contained two sites of significantly disordered lattice pentane/cyclopentane molecules which were only poorly modelled making use of Kratzert's DSR tool and SADI, SIMU and RIGU restraints. EADP was used on the borane boron atom B2. CHECKCIF indicates a low-coordinate metal atom as an A-level alert. Considering packing of the molecules very weak intermolecular Ga----Ga orientations can be identified with a Ga….Ga distance of 4.213 Å This is beyond the sum of Van-der-Waals-Radii (2 × 1.87 Å = 3.74 Å).[16]

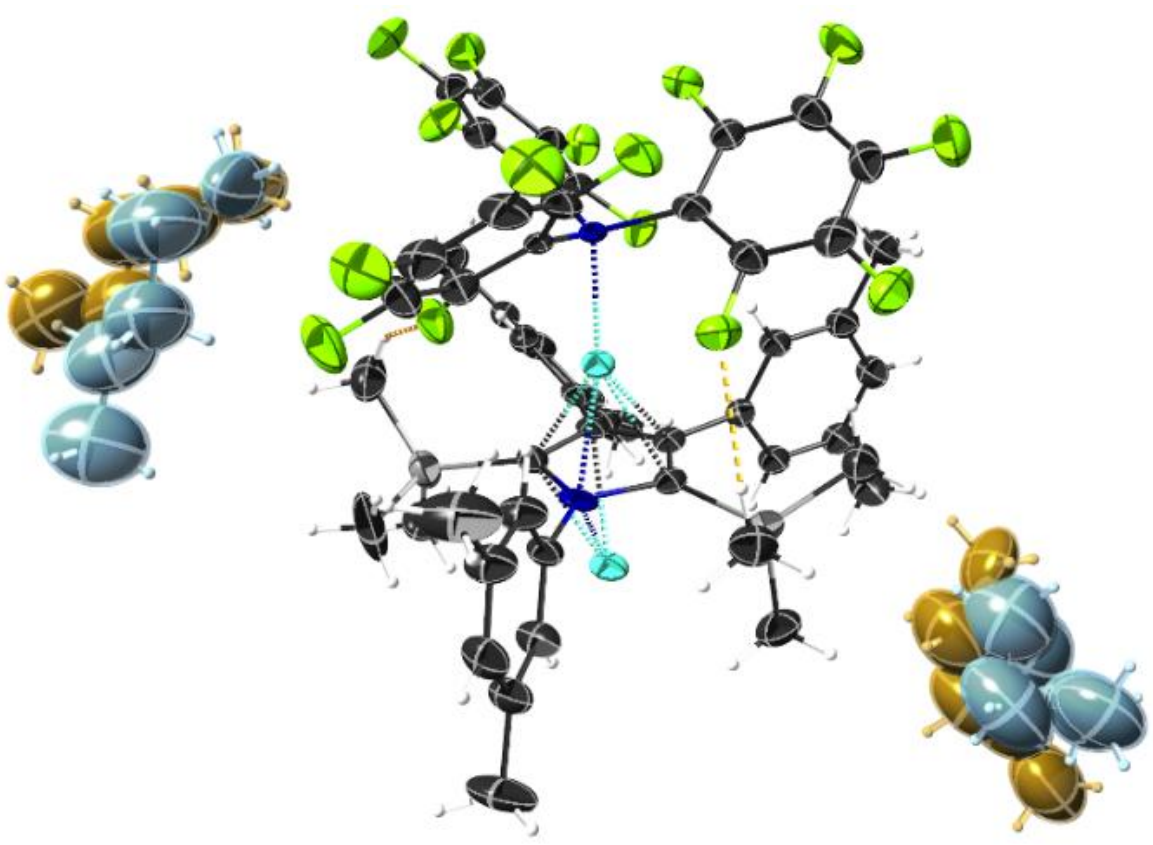

Figure SI16: Depiction of the asymmetric unit of **5** including the modelled disorder of lattice (cyclo)pentane molecules (PART1 blue, PART 2 orange)

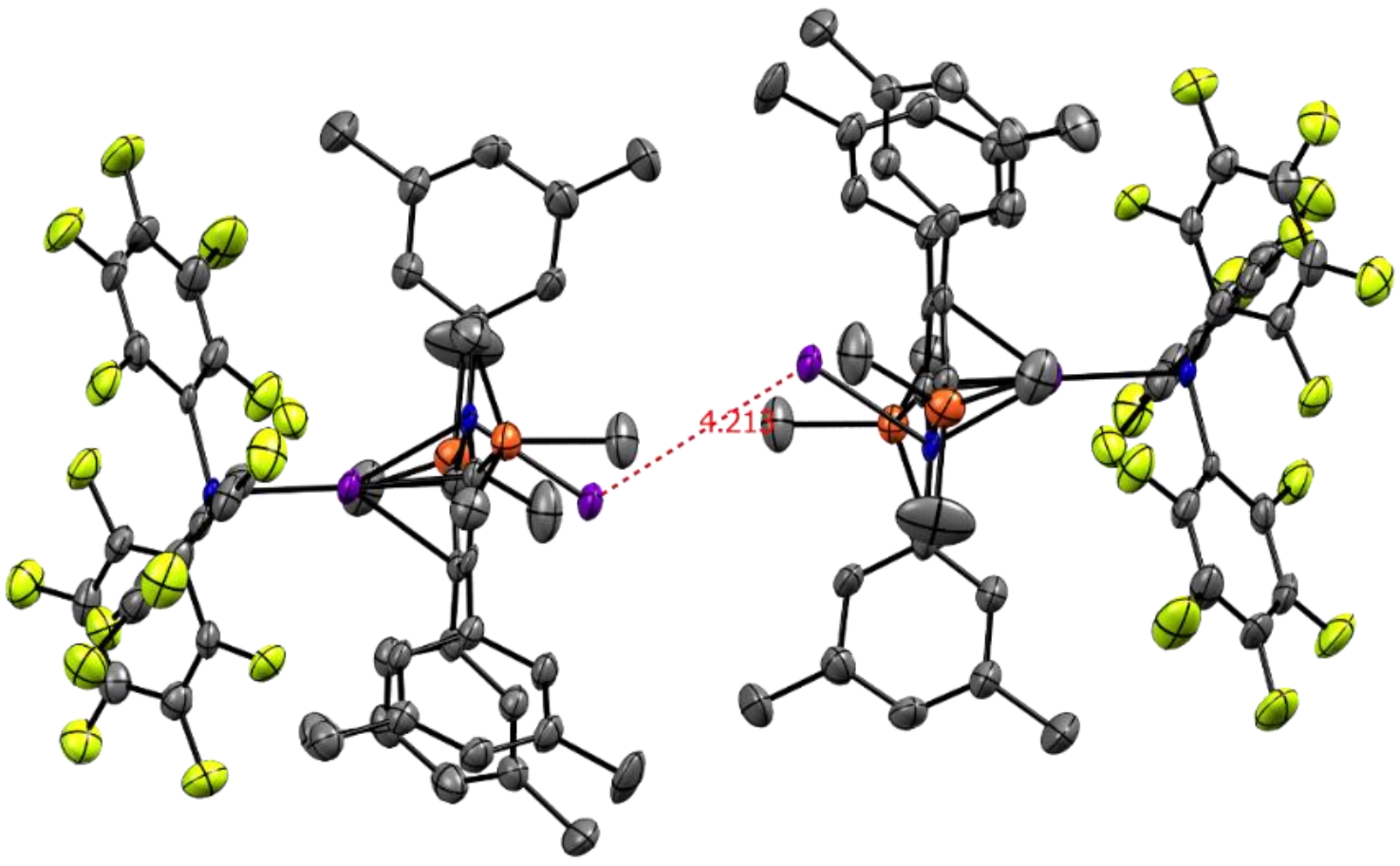

Figure SI17: Depiction of two molecules of **5** as an excerpt of the packing in the crystal with very long intermolecular Ga….Ga distances indicated.

Crystallographic and Refinement Details 6

Crystals of excellent quality were grown from cold (–40°C) solutions in toluene allowing for the acquisition data sets of resolutions down to 0.45 Å. Minor rotational disorder was only observed for a number of methyl groups.

Crystallographic and Refinement Details 7

Crystals were grown from solutions in diethyl ether. The structure contained a severely disordered lattice molecule of diethyl ether only partially occupying a special position. The severely disordered ether molecule was modelled over two positions using Kratzert's DSR tool and DFIX constraints as well as SADI, SIMU and RIGU restraints in PART -1. Attempts for further modelling did not lead to stable refinements.

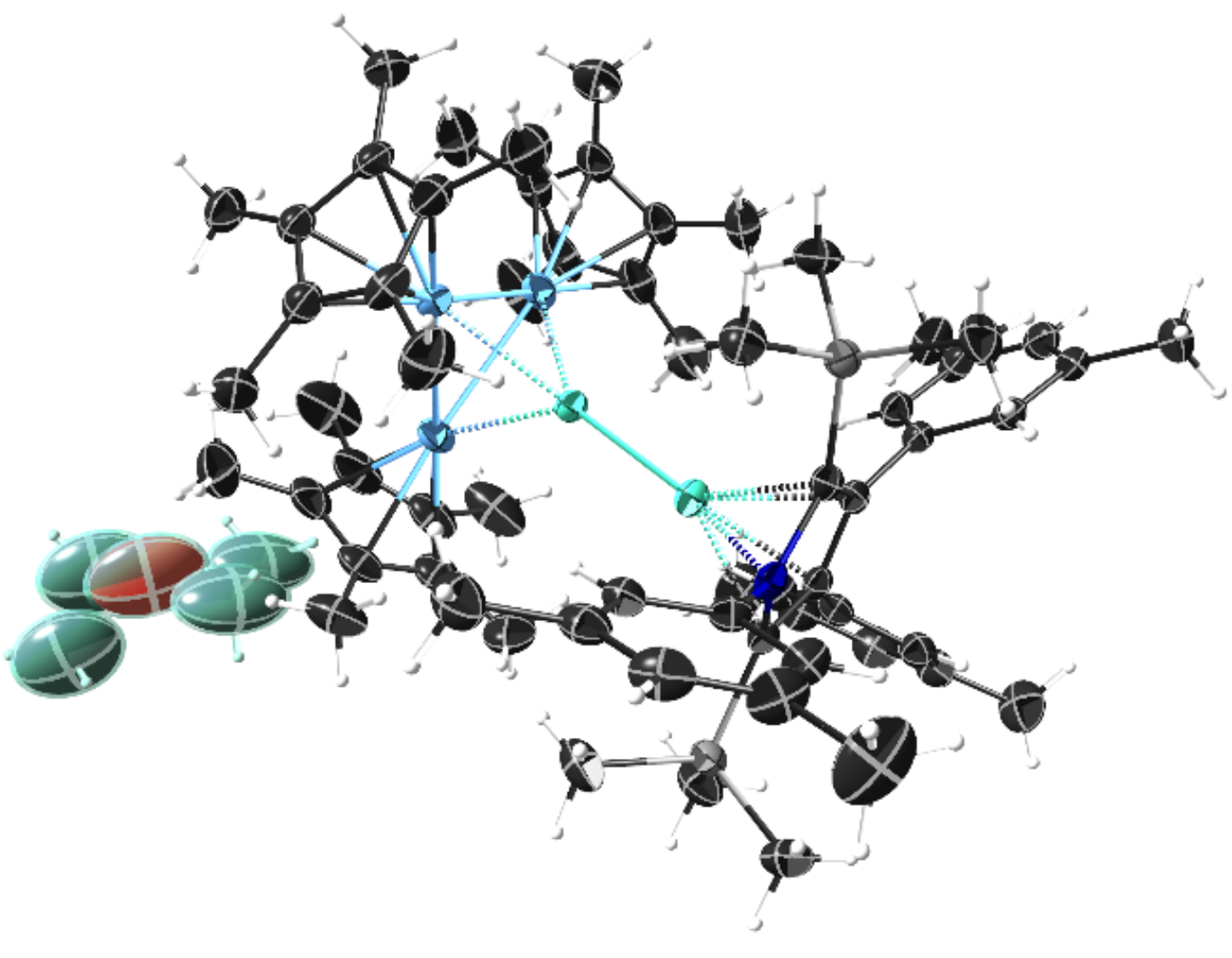

Figure SI18: Depiction of the asymmetric unit of **7** including the modelled disordered Et_2O molecule in PART-1

Crystallographic and Refinement Details 8

Crystals were grown from solutions in toluene at –40°C. The structure contained two molecules of **8** in the asymmetric unit and 4 molecules of toluene of which two were found to be disordered over two positions and modelled accordingly making use of Kratzerts DSR tool and FLAT, SADI, SIMU and RIGU restraints. **Note:** in some data sets obtained, the F_o-F_c residual electron density map suggests partial occupation of the borole based Ga-atom site by a lighter element, while a Cp*-bond Al-atom site seems to be partially occupied by a heavier element. Given the rather similar covalent radii of Ga and Al a reasonable structurally minimally invasive exchange would be between them. The effects are subtle and the assignment could not be corroborated by further analytical data.

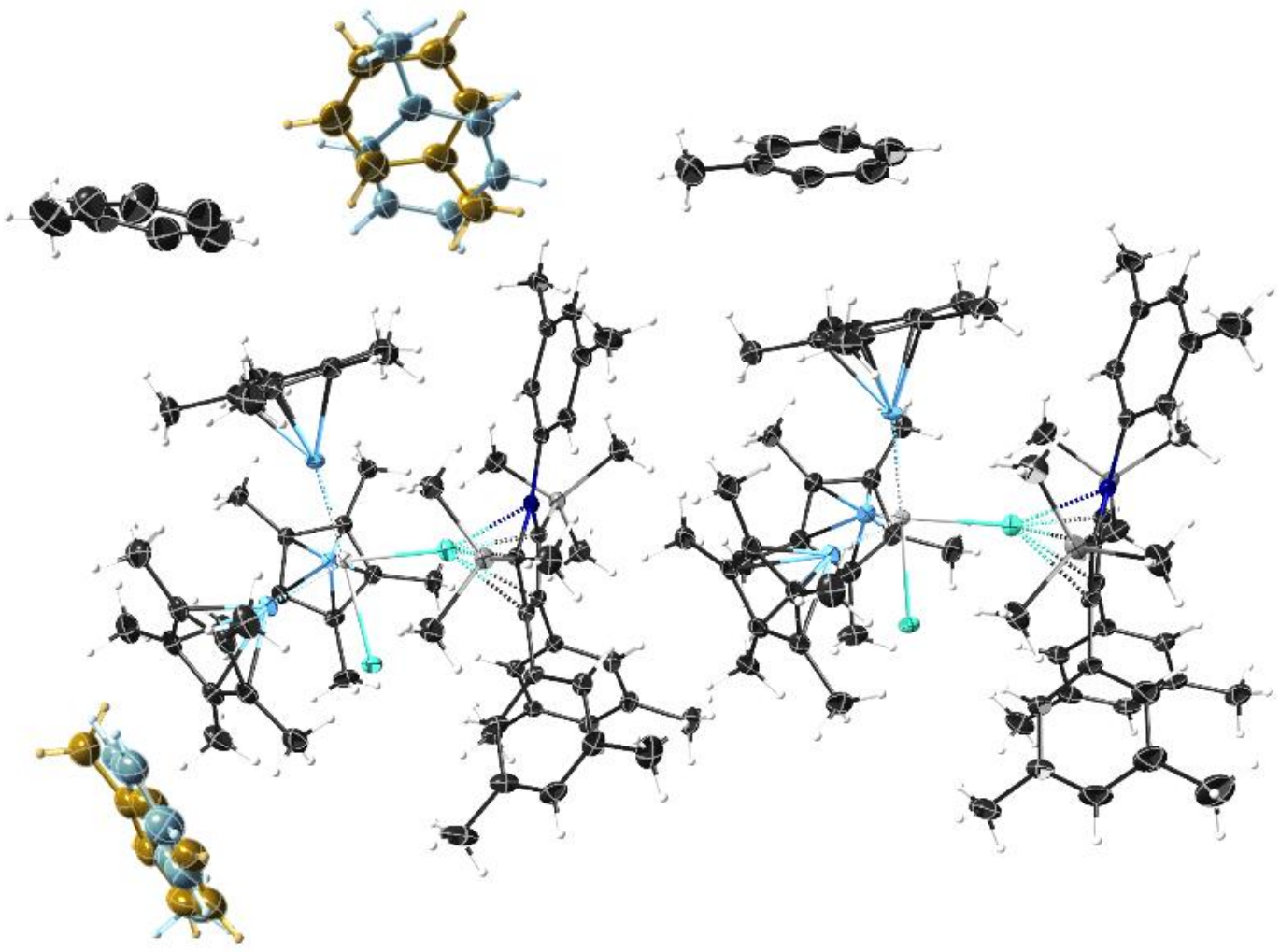

Figure SI19: Depiction of the asymmetric unit of **8** including the modelled disorder of lattice toluene molecules (PART1 blue, PART 2 orange)

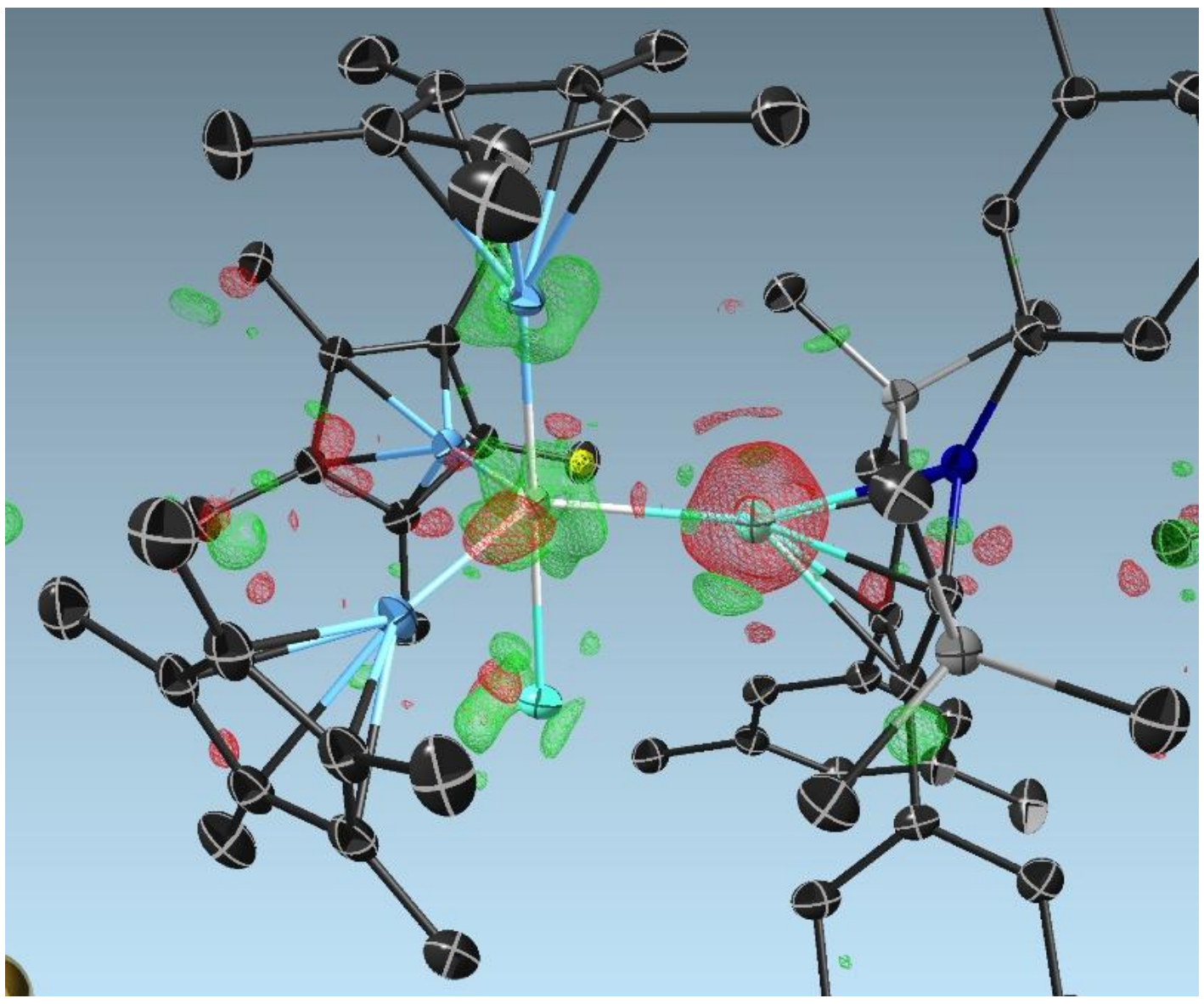

Figure SI20: Depictions of the residual electron density (F_o-F_c) map of an IAM refined data-set obtained of **8.** Revealing a slight excess of modelled electron density at the borole-bound Ga-atom position and some unaccounted minor residual electron density at the apical Cp*-Al-atom site.

Tabulated Crystallographic Details

CCDC number	2519914	2519919	2519920	2519917	2519915	2519916	2519918	2519921
Compound	**ALi$_2$(tol)$_2$×(tol)**	**2**	**3**	**4**	**5**	**6**	**7**	**8**
Empirical formula	$C_{55}H_{69}BLi_2Si_2$	$C_{52}H_{81}BGa_2Si_2$	$C_{39}H_{45}BGa_2O_5Si_2W$	$C_{44}H_{45}BGa_2O_{10}Si_2W_2$	$C_{62}H_{67.72}B_2F_{15}Ga_2Si_2$	$C_{82}H_{106}B_2Ga_2Si_4$	$C_{132}H_{190}Al_6B_2Ga_4OSi_4$	$C_{78}H_{106}Al_3BGa_2PtSi_2$
Formula weight	810.97	912.59	984.03	1307.93	1315.11	1365.08	2367.57	1526.08
Temperature [K]	150(2)	100(2)	100(2)	100(2)	160(2)	100.00(10)	160(2)	100(2)
Crystal system	orthorhombic	triclinic	monoclinic	orthorhombic	triclinic	triclinic	monoclinic	triclinic
Space group (#)	$Pbcn$ (60)	$P\overline{1}$ (2)	$P2_1/n$ (14)	$P2_12_12_1$ (19)	$P\overline{1}$ (2)	$P\overline{1}$ (2)	$C2/c$ (15)	$P\overline{1}$ (2)
a [Å]	11.628(2)	11.574(5)	12.6746(16)	11.2220(16)	11.8096(10)	11.4501(1)	44.1321(10)	16.3478(8)
b [Å]	21.614(4)	18.584(8)	20.002(2)	18.252(2)	15.9390(13)	12.7618(1)	12.5110(3)	20.0136(10)
c [Å]	19.893(4)	25.955(11)	16.728(2)	23.847(3)	17.0529(15)	14.3430(1)	26.8548(6)	25.3012(13)
α [°]	90	105.110(7)	90	90	84.026(5)	68.836(1)	90	68.108(2)
β [°]	90	91.928(7)	92.978(4)	90	80.481(5)	88.281(1)	114.3000(10)	87.645(2)
γ [°]	90	95.317(7)	90	90	81.815(5)	75.773(1)	90	82.624(2)
Volume [Å^3]	4999.7(17)	5357(4)	4235.1(9)	4884.3(11)	3122.8(5)	1890.54(3)	13513.8(5)	7617.3(7)
Z	4	4	4	4	2	1	4	4
ρ_{calc} [gcm^{-3}]	1.077	1.132	1.543	1.779	1.399	1.199	1.164	1.331
μ [mm^{-1}]	0.105	1.081	4.067	5.887	0.984	0.818	0.909	2.640
F(000)	1752	1952	1952	2528	1349	726	5032	3144
Crystal size [mm^3]	0.2×0.15×0.1	0.37×0.35×0.06	0.36×0.16×0.13	0.040×0.094×0.224	0.374×0.219×0.191	0.23×0.26×0.53	0.094×0.210×0.350	0.404×0.387×0.249
Crystal colour	colourless	colourless	colourless	colourless	colourless	yellow	red	red
Crystal shape	block	block	block	block	block	block	plate	block
Radiation	'Mo $K_{\alpha'}$ (λ=0.71073 Å)	MoK_α (λ=0.71073 Å)	MoK_α (λ=0.71073 Å)	MoK_α (λ=0.71073 Å)	MoK_α (λ=0.71073 Å)	Mo K_α (λ=0.71073 Å)	MoK_α (λ=0.71073 Å)	MoK_α (λ=0.71073 Å)
2θ range [°]	3.98 to 50.05 (0.84 Å)	2.28 to 55.09 (0.77 Å)	3.18 to 61.23 (0.70 Å)	2.81 to 52.76 (0.80 Å)	3.40 to 53.05 (0.80 Å)	3.54 to 64.28 (0.67 Å)	3.33 to 52.85 (0.80 Å)	3.04 to 61.11 (0.70 Å)
Index ranges	−13 ≤ h ≤ 11<br>−18 ≤ k ≤ 25<br>−23 ≤ l ≤ 23	−14 ≤ h ≤ 14<br>−24 ≤ k ≤ 24<br>−31 ≤ l ≤ 33	−18 ≤ h ≤ 18<br>−28 ≤ k ≤ 25<br>−23 ≤ l ≤ 23	−8 ≤ h ≤ 13<br>−22 ≤ k ≤ 22<br>−29 ≤ l ≤ 29	−14 ≤ h ≤ 14<br>−19 ≤ k ≤ 19<br>−21 ≤ l ≤ 21	−16 ≤ h ≤ 16<br>−18 ≤ k ≤ 18<br>−20 ≤ l ≤ 20	−55 ≤ h ≤ 55<br>−12 ≤ k ≤ 15<br>−33 ≤ l ≤ 33	−19 ≤ h ≤ 23<br>−27 ≤ k ≤ 28<br>−35 ≤ l ≤ 36
Reflections collected	21628	72082	75074	47855	38663	35470	58325	186400
Indep. reflections	4417<br>R_{int} = 0.1100<br>R_{sigma} = 0.0642	24496<br>R_{int} = 0.0502<br>R_{sigma} = 0.0577	12953<br>R_{int} = 0.0377<br>R_{sigma} = 0.0304	9946<br>R_{int} = 0.0591<br>R_{sigma} = 0.0605	12675<br>R_{int} = 0.0658<br>R_{sigma} = 0.0954	11134<br>R_{int} = 0.0191<br>R_{sigma} = 0.0220	13849<br>R_{int} = 0.0604<br>R_{sigma} = 0.0540	46537<br>R_{int} = 0.0324<br>R_{sigma} = 0.0377
Completeness to θ = 25.025°	99.9 %	100.0 %	100.0 %	100.0 %	99.9 %	100.0 %	100.0 %	100.0 %
Data / Restr. / Paramtrs.	4417/122/312	24496/928/1239	12953/0/463	9946 / 509 / 563	12675/391/854	11134 / 0 / 419	13849 / 185 / 724	46537/1008/1755
Absorption corr. T_{min}/T_{max} (method)	0.0729/1.0000 (multi-scan)	0.6433/0.7455 (multi-scan)	0.3467/0.7037 (numerical)	0.3993 / 0.8102 (numerical)	0.5625 / 0.7454 (multi-scan)	0.8805 / 1.0000 (multi-scan)	0.6601 / 0.7454 (multi-scan)	0.4319/0.6462 (numerical)
GooF on F^2	1.040	1.016	1.011	1.082	1.000	1.043	1.008	1.031
Final R indexes [I≥2σ(I)]	R_1 = 0.0820<br>wR_2 = 0.1804	R_1 = 0.0424<br>wR_2 = 0.0977	R_1 = 0.0236<br>wR_2 = 0.0468	R_1 = 0.0382<br>wR_2 = 0.0693	R_1 = 0.0649<br>wR_2 = 0.1597	R_1 = 0.0283<br>wR_2 = 0.0716	R_1 = 0.0411<br>wR_2 = 0.0930	R_1 = 0.0322<br>wR_2 = 0.0743
Final R indexes [all data]	R_1 = 0.1076<br>wR_2 = 0.2035	R_1 = 0.0674<br>wR_2 = 0.1068	R_1 = 0.0356<br>wR_2 = 0.0503	R_1 = 0.0539<br>wR_2 = 0.0742	R_1 = 0.1352<br>wR_2 = 0.2073	R_1 = 0.0326<br>wR_2 = 0.0729	R_1 = 0.0761<br>wR_2 = 0.1085	R_1 = 0.0544<br>wR_2 = 0.0846
Largest peak/hole [eÅ^{-3}]	0.52/−0.37	0.44/-0.50	1.83/−0.63	2.54/−1.24	1.67/-1.00	0.46/−0.33	0.66/−0.47	1.53/−2.43
Flack X parameter				0.397(12)				

Computational Details

Structure Optimisation, Frequency Calculation and Thermochemical Approximations

Computational examination was performed using ORCA (version 4.2.1.).[17] All neutral structures were initially optimised starting from experimental X-Ray structures were available using a RI-BP86-D3BJ[18] def2SVP/J model chemistry[19] in the gas phase followed by a frequency calculation on the same level of theory and thermochemical corrections were taken from these frequency calculations throughout this study. For numerical accuracy, grid6 and finalgrid7 were applied. No imaginary frequencies were observed confirming true minima, except for **4** (i50 cm^{-1}) and **H(**W(CO)$_5$)$_2$ (i23 cm^{-1}) for which a minor imaginary frequency was repeatedly obtained. All structures were then reoptimized using BP86-D3BJ-def2TZVP/J model chemistry and all property calculations as well as NBO analyses[20] are based on these gas phase structures. Finally, a single point calculation using BP86-D3BJ-def2TZVPP/J model chemistry and a solvent model (CPCM) for benzene as implemented in ORCA was performed to obtain thermochemical data. Graphical depictions were created using ChemCraft or IBOview.[21] For tungsten and platinum ECPs were automatically applied as implemented in ORCA.[22]

Assessment of isomers of compound **8** was performed using ORCA (version 6.1.0).[18, 23] The r^{2}SCAN-3c method incl. def2-mTZVPP basis-set and def2-mTZVPP/J auxiliaries were applied.[23f, 24] Geometry optimization with TIGHTOPT convergence criteria was performed prior to a frequency calculation all applying a numerical accuracy of DEFGRID3. To account for solvent effects a singlepoint calculation using r^{2}SCAN-3c method and CPCM for benzene as implemented in ORCA was performed. Starting points for these optimizations of putative isomers was in all cases the optimised X-ray structure of compound **8** from which Al and Ga atom positions were switched *in silico*.

Table SI2: Thermochemical data for computationally addressed compounds and reactions considered:

		BP86-D3BJ/**def2-TZVP**/J (gasphase structures); thermal corrections from def2-SVP frequency calculations in Hartree								single point BP86-D3BJ/**def2-TZVPP**/J (**CPCM = benzene)**, thermal corr. From def2-SVP calcs; in Hartree			
**#**	**MOLECULE**	**E(SCF)**	**$E(ZPV)_{corr}$**	**H_{corr}**	**G_{corr}**	**E(ZPV)**	**H**	**G**		**E(SCF,solv)**	**H(solv)**	**G(solv)**	
0	$[GaCp^*Ga]^+$	-4240,374147	0,214778	0,232775	0,170484	-4240,159369	-4240,141372	-4240,203663		-4240,420886	-4240,18811	-4240,250401	
1	$B(C_6F_5)_3$	-2209,331003	0,149932	0,180628	0,093277	-2209,181071	-2209,150375	-2209,237726		-2209,334942	-2209,154314	-2209,241665	
2	THF	-232,5514624	0,113156	0,119106	0,085190	-232,4383065	-232,4323568	-232,466272		-232,5572311	-232,4381255	-232,4720407	
3	**A**	-1927,35881	0,667776	0,715438	0,595277	-1926,691034	-1926,643372	-1926,763533		-1927,382589	-1926,667151	-1926,787312	
4	**1**	-5777,735229	0,669436	0,720813	0,592389	-5777,065793	-5777,014416	-5777,14284		-5777,758995	-5777,038182	-5777,166606	
5	**2**	-6485,69263	1,163225	1,237214	1,069798	-6484,529404	-6484,455416	-6484,622832		-6485,730015	-6484,492802	-6484,660218	
6	$(Cp^*Al)_4$	-2531,113749	0,862276	0,924575	0,777622	-2530,251473	-2530,189174	-2530,336127		-2531,142088	-2530,217513	-2530,364466	
7	(THF)W(CO)$_5$	-866,8860462	0,155079	0,174821	0,108755	-866,7309669	-866,711225	-866,7772907		-866,8966573	-866,7218362	-866,7879018	
8	**3** [**1**(W(CO)$_5$]	-6412,105878	0,711818	0,776744	0,621635	-6411,39406	-6411,329134	-6411,484243		-6412,132334	-6411,35559	-6411,510699	
9	**4** [**1**(W(CO)$_5$]$_2$	-7046,469047	0,752649	0,830134	0,651145	-7045,716398	-7045,638913	-7045,817902		-7046,496376	-7045,666242	-7045,845231	
10	**5** [**1**(B(C_6F_5)$_3$)]	-7987,108954	0,823445	0,904224	0,721425	-7986,285509	-7986,20473	-7986,387529		-7987,136122	-7986,231898	-7986,414697	
11	[**1**(B(C_6F_5)$_3$)$_2$]	-10196,46684	0,976733	1,086884	0,850538	-10195,49011	-10195,37996	-10195,61631		-10196,49389	-10195,40701	-10195,64336	
12	**6** Digallane	-7705,153043	1,340431	1,438652	1,220551	-7703,812612	-7703,71439	-7703,932492		-7705,198814	-7703,760161	-7703,978263	
13	**7**	-7676,095945	1,317974	1,416238	1,198886	-7674,777971	-7674,679706	-7674,897059		-7676,139308	-7674,72307	-7674,940422	
14	**8** Pt-Complex	-7795,791714	1,319385	1,419409	1,199275	-7794,472328	-7794,372305	-7794,592439		-7795,838687	-7794,419278	-7794,639412	
15	Model Ga(C_4BH_5)Ga **H**	-4030,65733	0,078136	0,086347	0,045035	-4030,579194	-4030,570983	-4030,612295		-4030,661081	-4030,574734	-4030,616046	
16	Model-**H**(W(CO)$_5$)	-4665,010574	0,119194	0,141689	0,068628	-4664,89138	-4664,868885	-4664,941946		-4665,019446	-4664,877757	-4664,950819	
17	Model **H**(W(CO)$_5$)$_2$	-5299,353053	0,159769	0,195966	0,092876	-5299,193284	-5299,157087	-5299,260178		-5299,365944	-5299,169979	-5299,273069	
18	**H-(Al_3Cp_3) DISPERSION**	-5338,963984	0,324026	0,354091	0,264314	-5338,639958	-5338,609893	-5338,69967					
19	**H-(Al_3Cp_3) NO-DISP**	-5338,856774	0,323850	0,352163	0,266800	-5338,532925	-5338,504611	-5338,589975					
		Optimization and frequency calculation using r^2-SCAN-3c model chemistry energies include dispersion and counterpoise corrections, given in Hartree							rel. free energies kcal mol^{-1}	Single-point r^2-SCAN-3c model chemistry with **CPCM = benzene**, thermal corr. from gas phase calculations; in Hartree			rel. free energies kcal mol^{-1}
20	**8** Pt-Complex	-7792,944283	1,353107	1,450382	1,236312	-7791,591176	-7791,4939	-7791,707971	4,63	-7792,963596	-7791,513213	-7791,727284	4,03
21	Isomer **8-I**	-7792,951862	1,353191	1,450394	1,236509	-7791,59867	-7791,501467	-7791,715353	0	-7792,970213	-7791,519819	-7791,733704	0
22	Isomer **8-II**	-7792,947239	1,353253	1,450281	1,237093	-7791,593986	-7791,496958	-7791,710146	3,27	-7792,965122	-7791,514841	-7791,728028	3,56
23	Isomer **8-III**	-7792,919092	1,352209	1,449880	1,234537	-7791,566883	-7791,469213	-7791,684555	19,33	-7792,937788	-7791,487908	-7791,70325	19,11
24	Isomer **8-IV**	-7792,922182	1,352669	1,449875	1,236037	-7791,569513	-7791,472307	-7791,686145	18,33	-7792,939374	-7791,489499	-7791,703337	19,06

			BP86-D3BJ**def2TZVP** (gasphase structures) in kcal mol^{-1}					BP86-D3BJ**def2TZVPP (CPCM = benzene)** in kcal mol^{-1}		
**ENTRY**	**Reactant Entries #**	**Reaction Description**	**ΔE(SCF)**	**ΔE(ZPV)**	**ΔH**	**ΔG**		**ΔE(SCF,solv)**	**ΔH(solv)**	**ΔG(solv)**
RXN1	4+5 –> 8 + 2	**1** + (THF)W(CO)$_5$ –> **3** + THF	-22,63	-22,34	-22,50	-19,07		-21,28	-21,15	-17,72
RXN2	8 + 5 –> 9 + 2	**3** + (THF)W(CO)$_5$ –> **4** + THF	-17,94	-18,62	-19,40	-14,21		-15,45	-16,91	-11,72
RXN 3	15 + 5 –> 16 + 2	**H** + (THF)W(CO)$_5$ –> **H**(W(CO)$_5$) + THF	-11,71	-12,25	-11,94	-11,69		-11,88	-12,12	-11,87
RXN 4	16 + 5 –> 17 + 2	**H**(W(CO)$_5$) + (THF)W(CO)$_5$ –> **H**(W(CO)$_5$)$_2$ + THF	-4,95	-5,80	-5,86	-4,53		-4,44	-5,34	-4,01
RXN5	4 + 1 –> 10	**1** + B(C_6F_5)$_3$ –> **5**	-26,81	-24,25	-25,06	-4,37		-26,47	-24,73	-4,03
RXN6	10 + 1 – > 11	**5** + B(C_6F_5)$_3$ –> Bis-adduct **1**[B(C_6F_5)$_3$]$_2$	-16,87	-14,77	-15,60	5,62		-14,33	-13,05	8,16
RXN7	4 + 3 –>12	**1** + **A** –> **6**	-37,03	-35,01	-35,52	-16,39		-35,91	-34,41	-15,28
RXN8	4 + 0.75×6 –> 13	**1** + 0.75×$(Cp^*Al)_4$ –> **7**	-15,94	-14,79	-14,69	-1,33		-14,90	-13,65	-0,29

NBO, NLMO and NRT Analyses

NBO, NLMO and NRT analyses were conducted using NBO7.0.[20] Wavefunctions to be analysed by NBO were obtained on the structures optimised on BP86-D3BJ-def2TZVP/J level of theory. BP86 single point calculation with def2-SVP basis sets on the organic substituents and def2-TZVP basis set for the elements of the central heterocyclic [C_4BGa]-fragment were chosen.

ETS-NOCV

ETS-NOCV analysis[25] was performed using ADF2022 programme suite with BP86-D3BJ[18-19]/TZ2P[26] model chemistry on the basis of an experimental molecular structure obtained from X-ray crystallography with only the hydrogen positions of one molecule of **7** optimised in by DFT (BP86-D3BJ/TZ2P).[27] Depictions of deformation densities and SFO were prepared using ADF2022.

Frontier molecular orbitals of Cp*Ga, [GaCp*Ga]$^+$, H and 1

Graphical depictions of molecular orbitals were created with iboView with an *iso*-density-level of 80% and orbital energies are taken from wavefunctions of BP86-D3BJ/def2-TZVP calculations.

Cp*Ga:

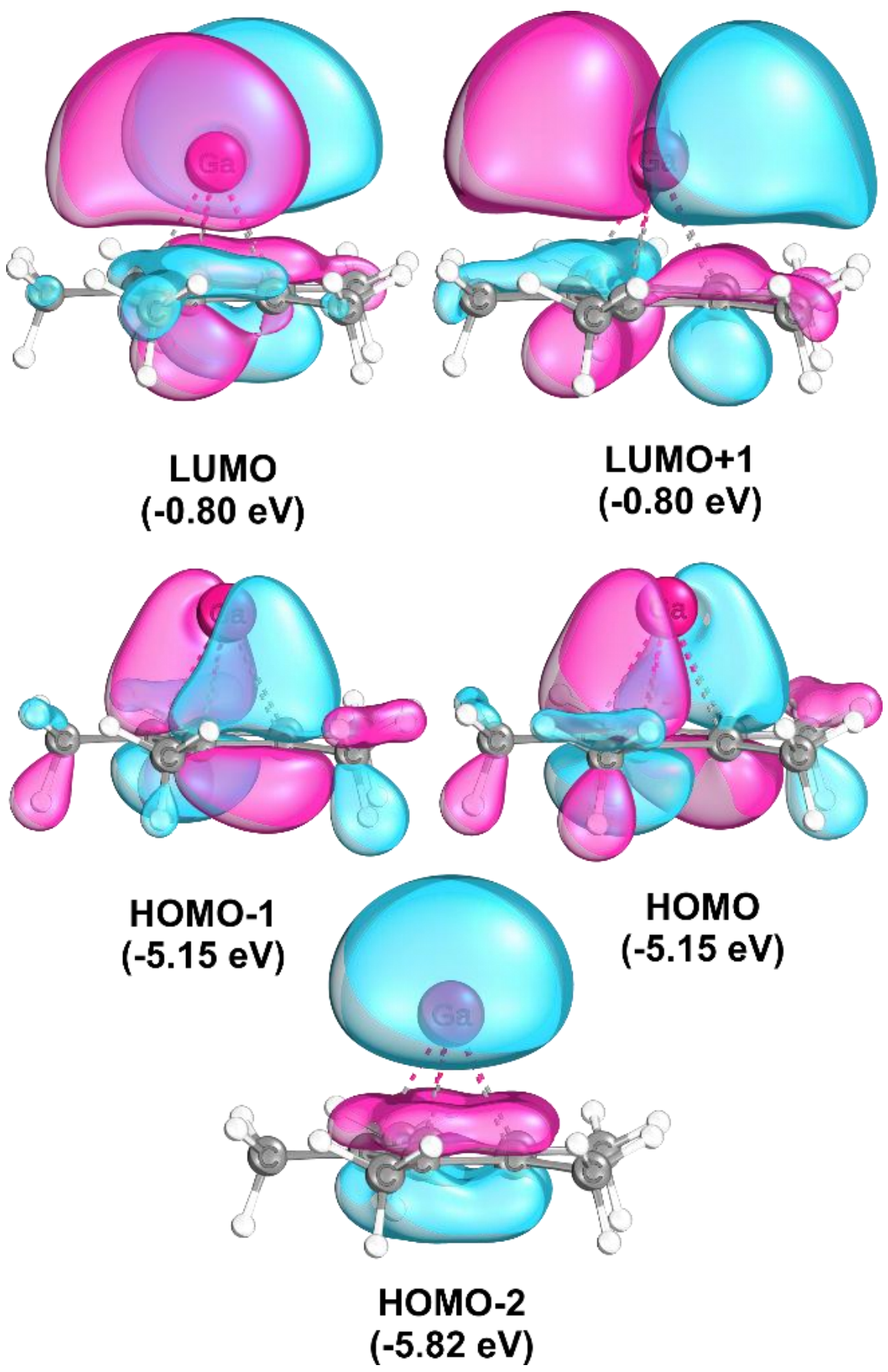

Figure SI21

[GaCp*Ga]⁺:

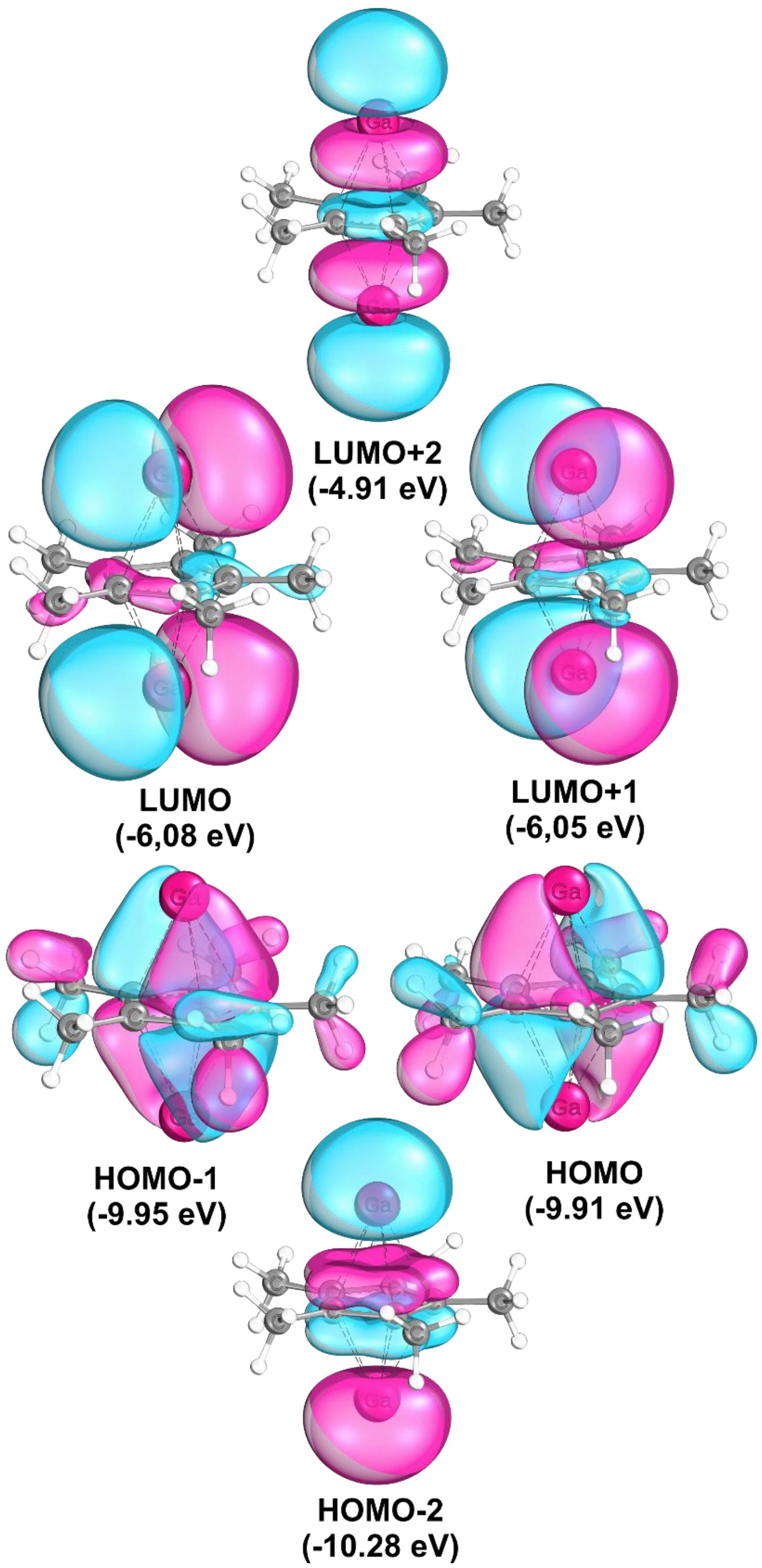

Figure SI22

Model Compound H Ga(C_4BH_5)Ga

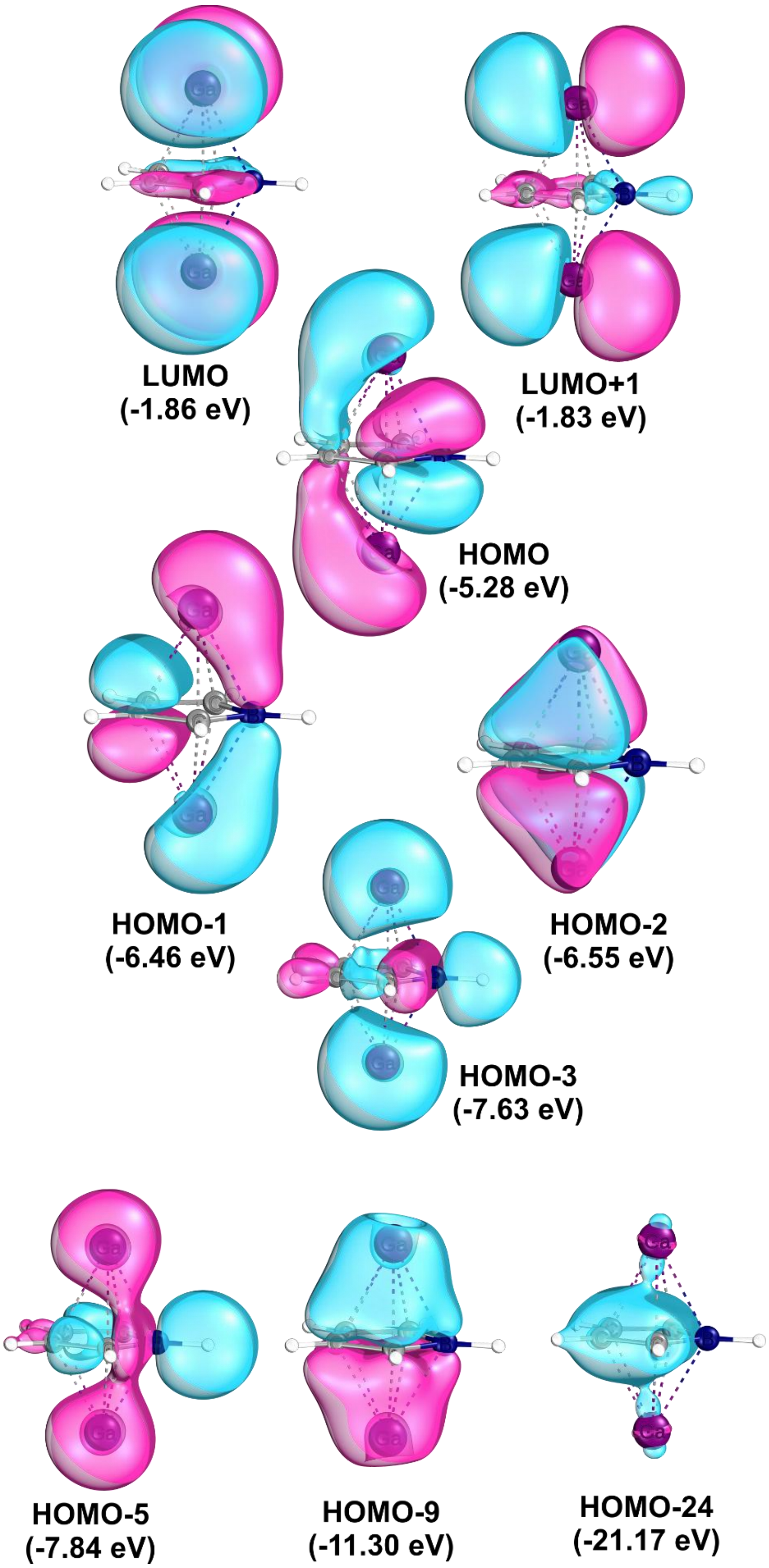

Figure SI23

Compound 1

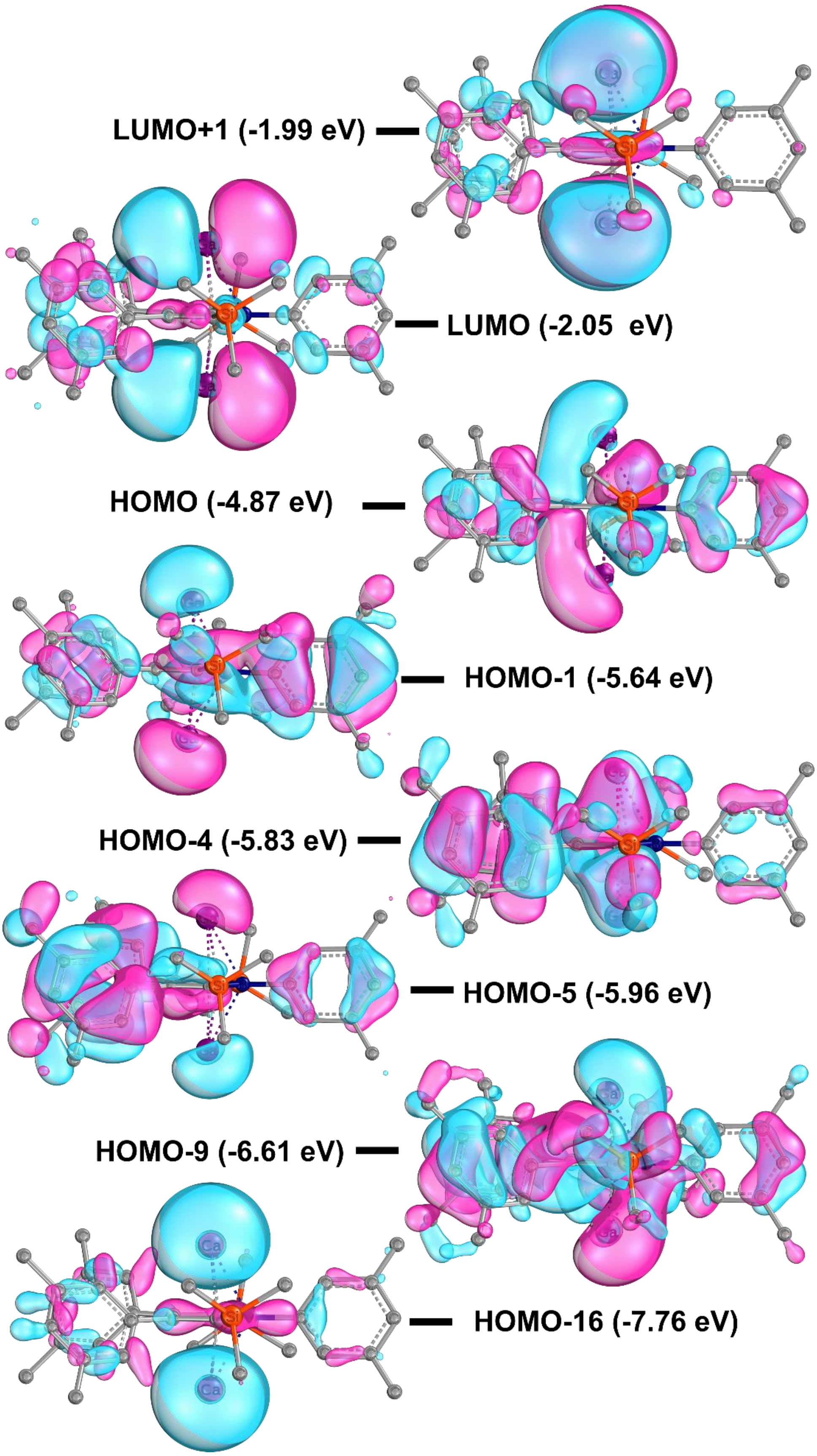

Figure SI24

Summary and comparison of HOMO of 1, 3 and 5

Graphical depictions of molecular orbitals were created with iboView with an *iso*-density-level of 80% and orbital energies are taken from wavefunctions of BP86-D3BJ/def2-TZVP calculations.

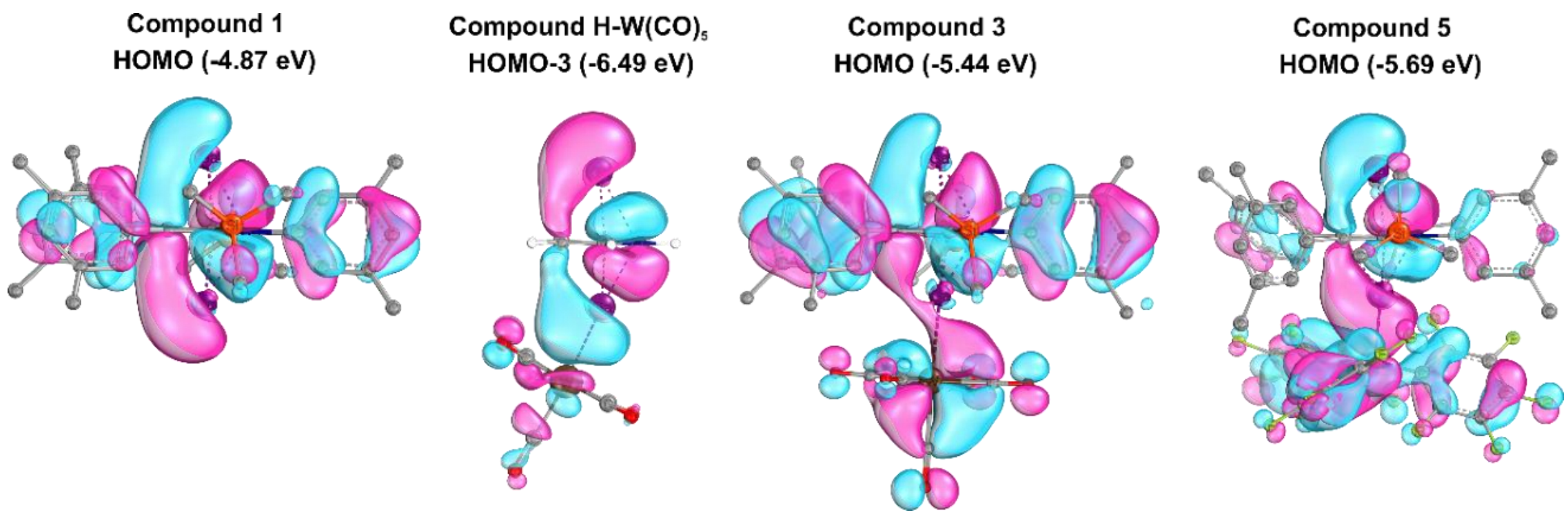

Figure SI25

Frontier molecular orbitals of 6

Graphical depictions of molecular orbitals were created with iboView with an *iso*-density-level of 80% and orbital energies are taken from wavefunctions of BP86-D3BJ/def2-TZVP calculations.

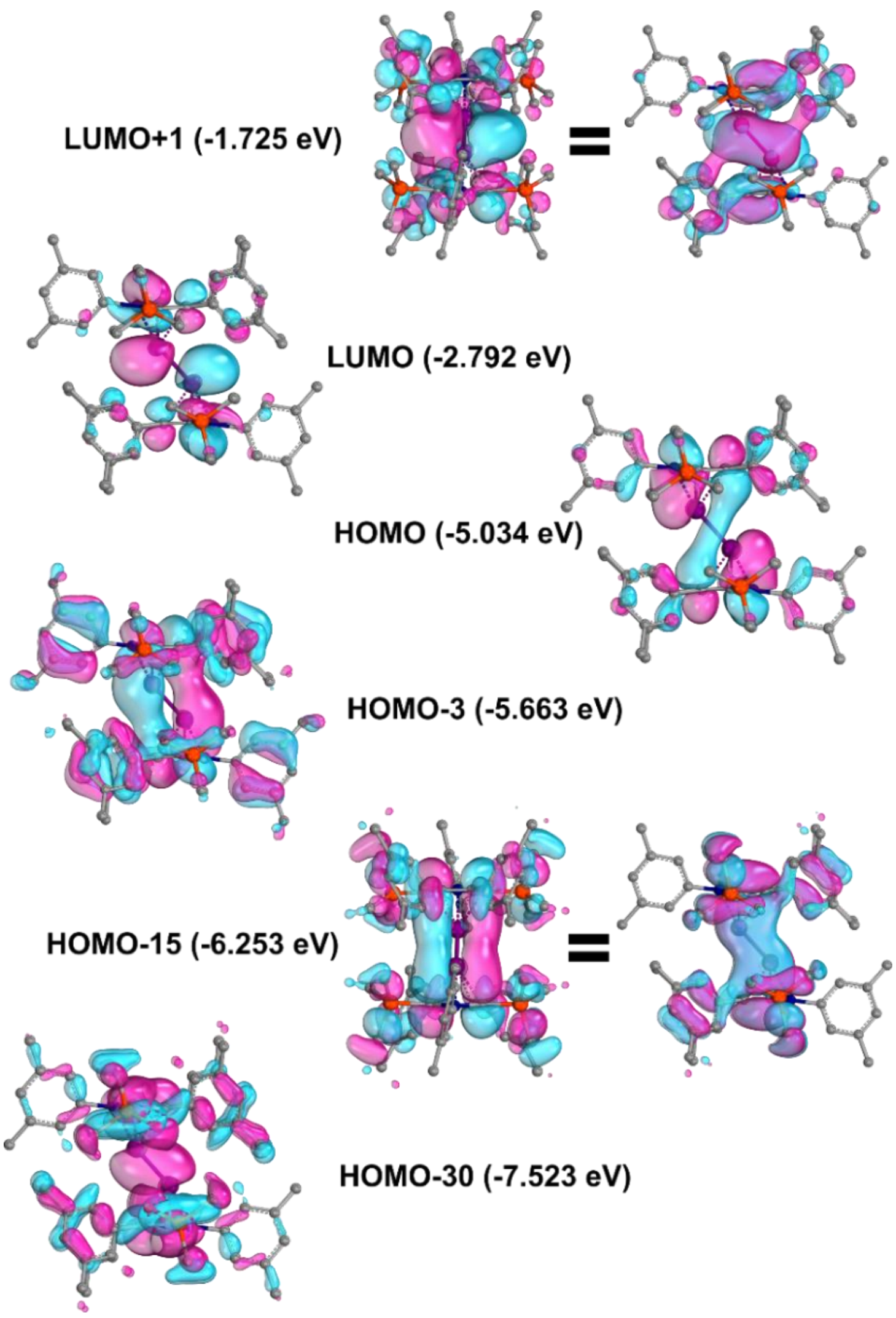

Figure SI26

IBO-Analysis of H

An “intrinsic basis bonding analysis (IBBA)” was performed using the default settings implemented in IBOview.[21] A summary of the relevant Ga-borole-Ga bonding IBOs is given below and depicted (*iso*-density-level 80%). The localized perspective on bonding in **H** is represented by three multicenter-2 electron bonding interactions involving the borole and both Ga-atoms. IBO48 and IBO47 represent two 5c-2e-bonds (2 Ga atoms and $BC_{\alpha}C_{\beta}$ moiety) and IBO48 represents a 4c2e-bond of two Ga-atoms and the $C_{\beta}C_{\beta}$-moiety. Low-lying IBO44 and IBO43 represent s-orbital-type Ga lone-pairs.

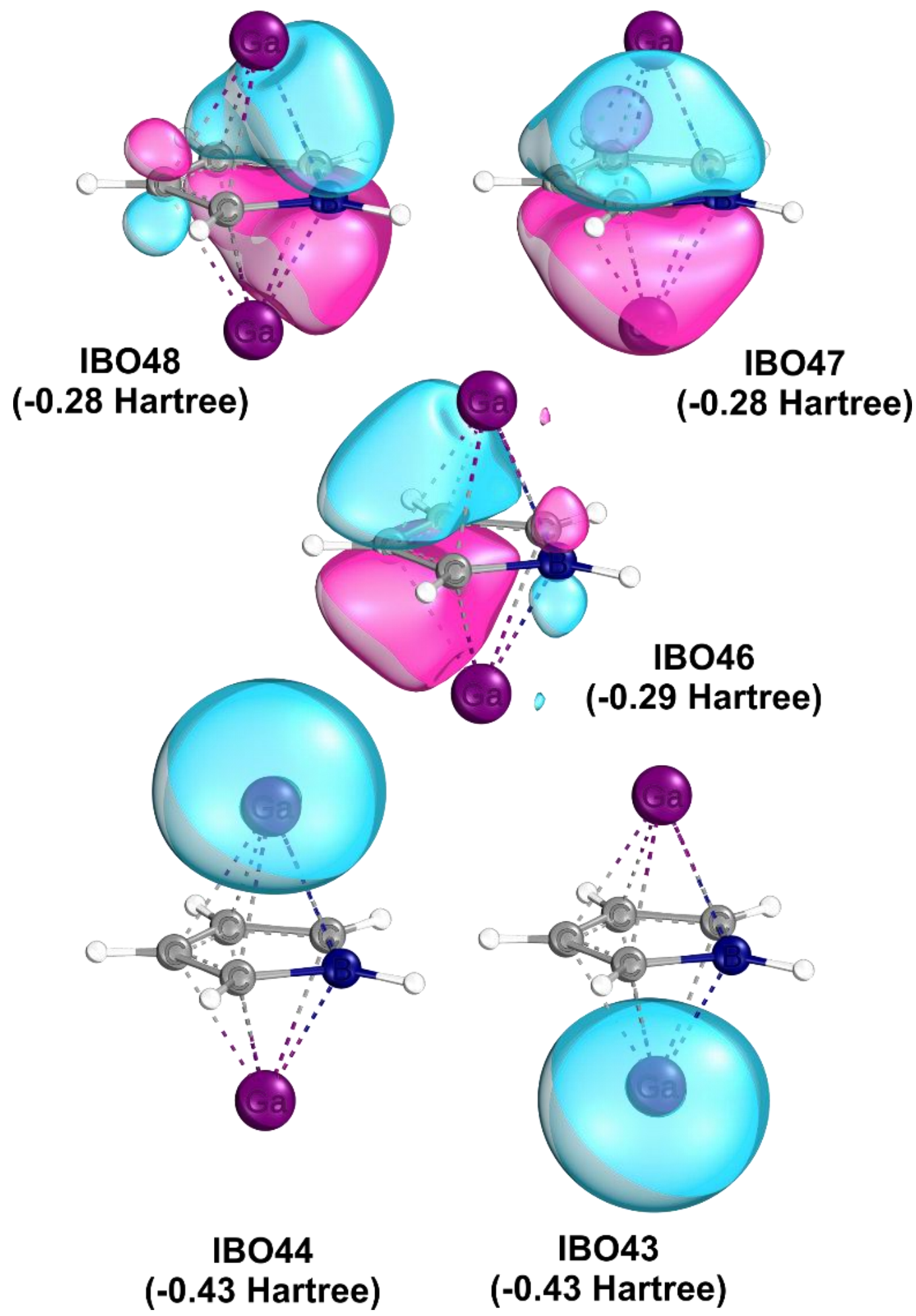

Figure SI27

Summary of localized orbital composition: [THRPRINT= 0.020]

```
 ORB  GRP   ORB.ENERGY   CENTERS/CHARGES
   43   AB    -0.426477   GA  2  1.958  GA  1  0.028
   44   AB    -0.426263   GA  1  1.958  GA  2  0.028
   45   AB    -0.345570    B  3  0.995   H  8  0.969   (other: 0.036)
   46   AB    -0.291044    C  5  0.781   C  6  0.781  GA  1  0.172  GA  2  0.172   B  3  0.078
   47   AB    -0.277084    C  7  1.014   B  3  0.298  GA  1  0.216  GA  2  0.216   C  6  0.177   C  5  0.074
   48   AB    -0.277084    C  4  1.014   B  3  0.298  GA  1  0.216  GA  2  0.216   C  5  0.177   C  6  0.074
```

ETS-NOCV on $Ga(H_4C_4BH)Ga$ H and $[GaCp^*Ga]^+$ for comparison

An ETS-NOCV analysis (BP86-D3BJ/TZ2P) analysis was performed on the fragments $[Ga(H_4C_4BH)]^-$....(Ga^+) and GaCp*....(Ga^+) to elucidate the differences in bonding interactions between the Ga^+ ion and the π-donor- *nido*-cluster fragment. Graphical depictions of the leading NOCV interactions and contribution fragment orbitals are provided. Different density plots are shown at an iso-density surface-value of 0.003 a.u. (green: positive charge difference; magenta: negative charge difference) and SFO are shown at an iso-density surface value of 0.04 a.u.. Results of the energy decomposition analysis are tabulated below.

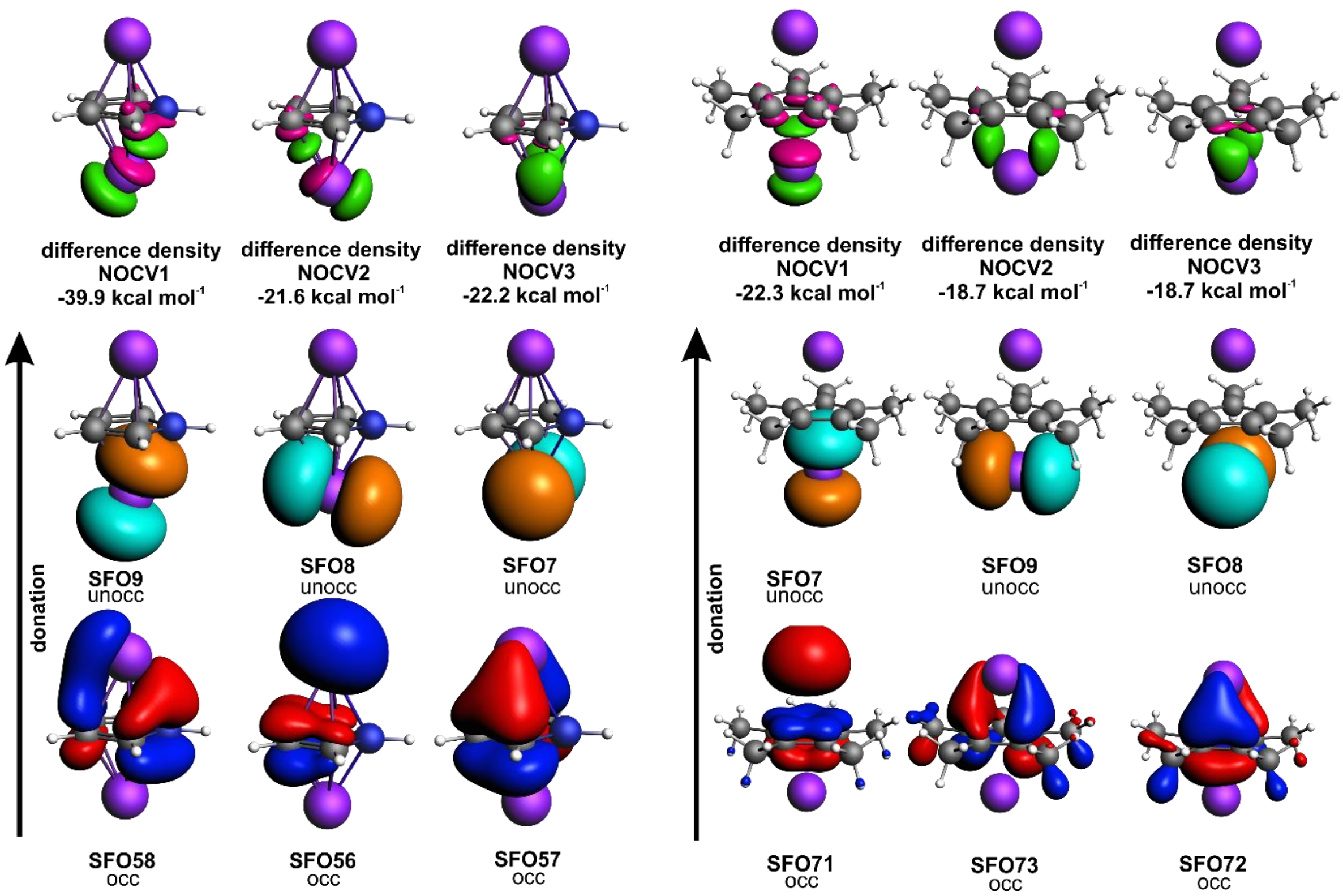

Figure SI28: Left: Relevant NOCV-interactions of $[Ga(H_4C_4BH)]^-$ towards Ga^+; right: relevant NOCV-interactions of GaCp* towards Ga^+.

Table SI3: EDA-NOCV results for the bonding interactions of $[Ga(H_4C_4BH)]^-$ and GaCp* respectively towards (Ga^+). Energies given in kcal mol^{-1}.

Energy	$[Ga(H_4C_4BH)]^-$--(Ga^+)	GaCp*--(Ga^+)
ΔE_{int}	–170.7	–62.9
ΔE_{Pauli}	91.6	72.3
ΔE_{disp}	–5.0 (2 %)	–8.7 (6 %)
ΔE_{elstat}	–159.8 (61 %)	–51.0 (38 %)
ΔE_{orb}	–97.5 (37 %)	–75.5 (56 %)

The ΔE_{int} between $[Ga(H_4C_4BH)]^-$ and (Ga^+) (–170.7 kcal mol^{-1}) is much larger than for GaCp*--(Ga^+) (–62.9 kcal mol^{-1}) which is to be expected due to much higher ΔE_{elstat} values between oppositely charged species. However, the absolute ΔE_{orb} value describing the covalent interaction contribution between the Ga^+ ion and the π-donor fragment is ca 29% higher for $[Ga(H_4C_4BH)]^-$--(Ga^+) (–97.5 kcal mol^{-1}) than for GaCp*--(Ga^+) (–75.5 kcal mol^{-1}) corroborating the significantly stronger covalent bonding in **H** compared the the known $[GaCp^*Ga]^+$ cation.

Experimental *vs*. DFT-optimised (gas phase) structures with and without dispersion of Compound 7

They key experimental geometrical features of compound **7** (i.e. the relative orientation of the anionic *nido*-[**A**Ga]$^-$ cluster fragment and the $[GaAl_3Cp^*_3]^+$ were found to be hard to reproduce by various DFT functionals. Especially the Ctr(C_4B)-Ga-Ga angle was repeatedly found to be on a very shallow energy surface with little energetic difference made from by significant changes to the angle. We reason, that this behaviour further corroborates our ionic description of an electrostatic rather than covalent Ga-Ga interaction between the two fragments.

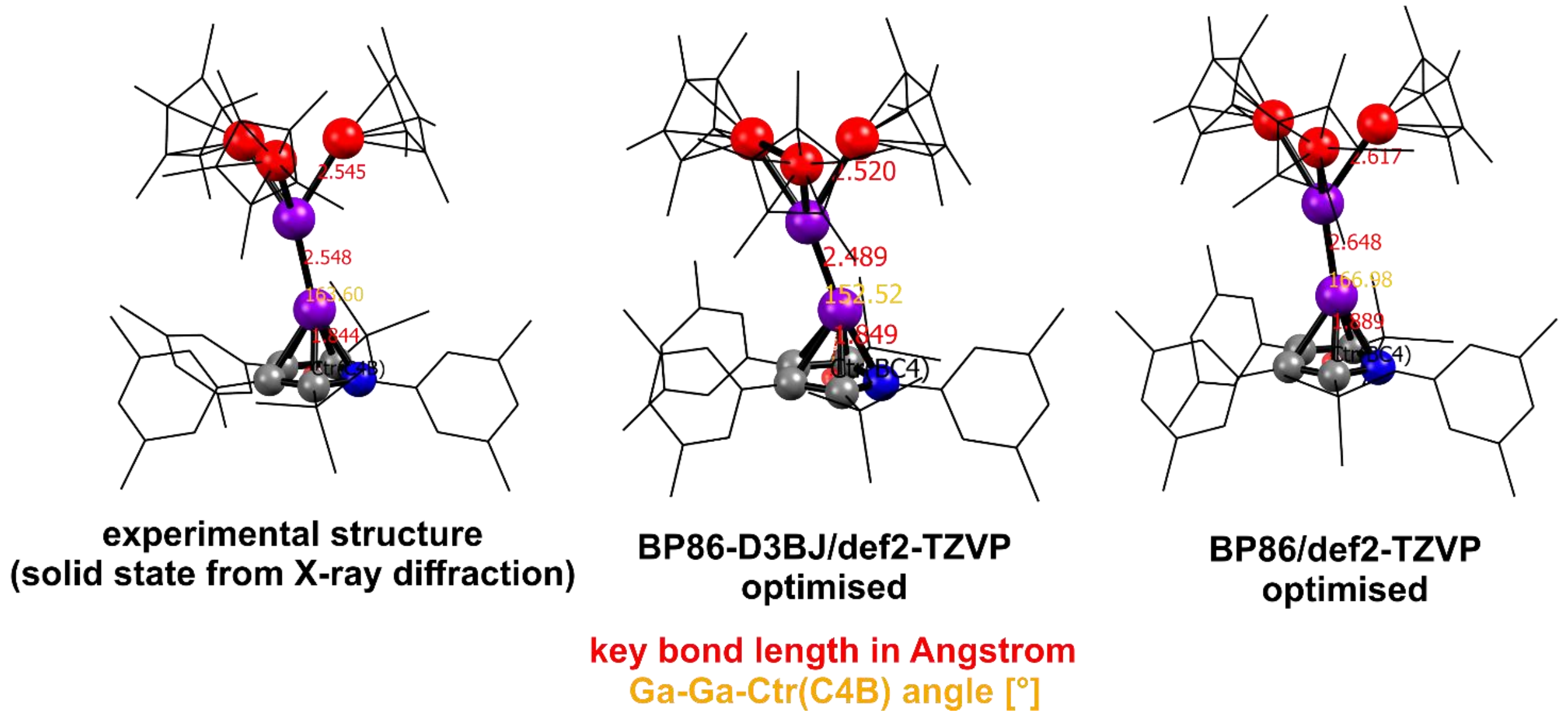

Figure SI29

The vast flexibility of the Ga-Ga-Ctr(C_4B) angle becomes also obvious from comparison with the respective angle in a computationally assessed, sterically vastly trimmed complex as shown below (hydrogen atoms omitted for clarity). Starting from the structure obtained in **7** Cp* was reduced to Cp and borole **A** was trimmed down to parent $(HC)_4BH$. The Ga-Ga-Ctr(C_4B) angle vastly varies, again depending on whether or not dispersion was considered during the optimisation. The overall angle is much smaller than experimentally observed again suggesting a rather shallow energy surface of the Ga-Ga-Ctr(C_4B) mode.

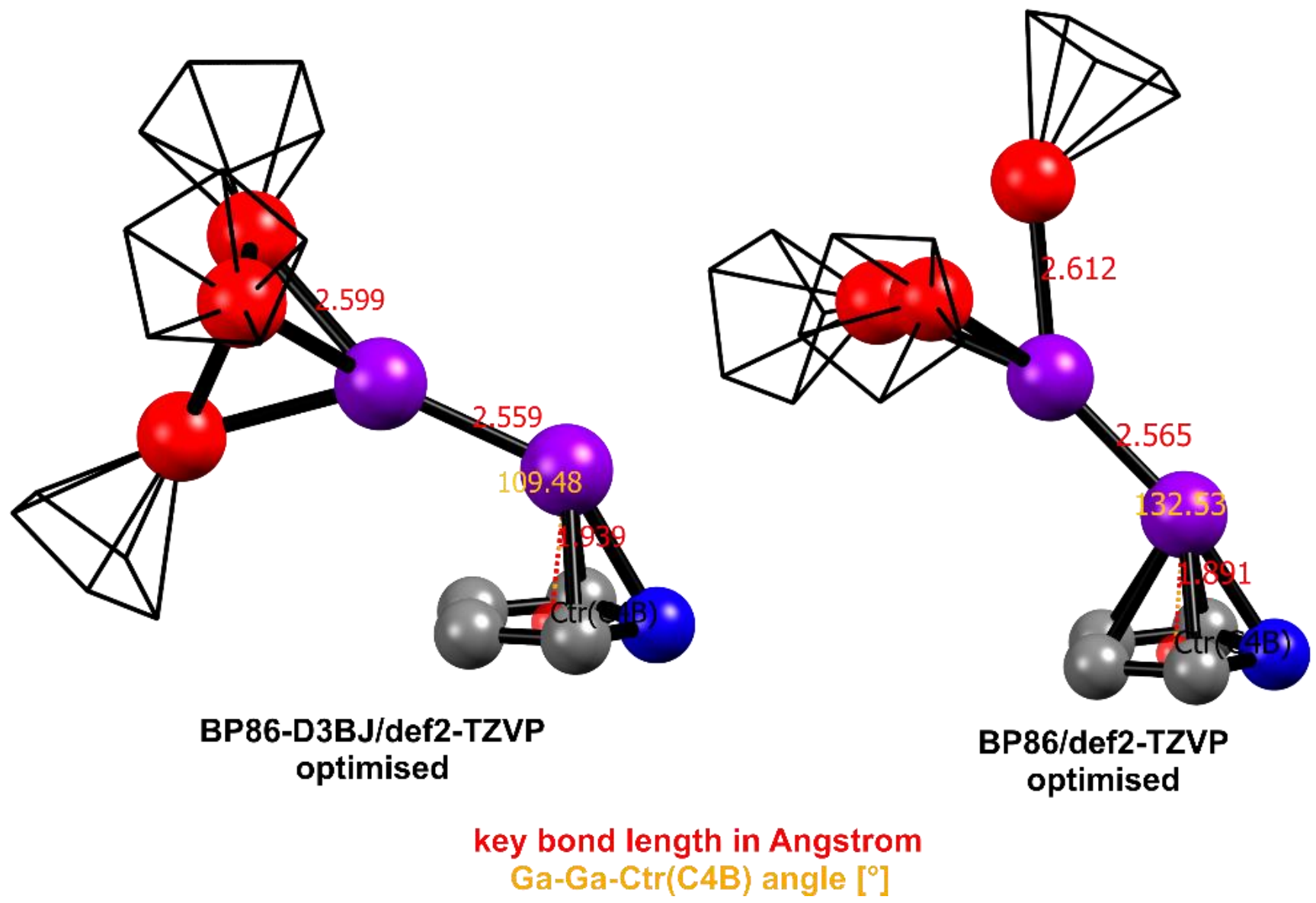

Figure SI30

XYZ-coordinates of optimised structures

Structures optimised at RI-BP86-D3BJ\def2TZVP\J level of theory or on r²SCAN-3c level of theory (see above).

27
Enry 0 - (GaCp*Ga)-Cation // -4240.374147399389
Ga 2.90286036210420 7.01779494853762 4.79721201933367
C 2.72182046497631 4.49158153735806 5.13590058842589
Ga 4.74422836802805 2.93935810615315 4.95140680377193
C 3.26596588220108 4.68746858716676 3.81318546505751
C 4.57400043100031 5.28310730100731 3.95335286406593
C 4.83941543881552 5.45612014337367 5.36284161354833
C 3.69291593231623 4.96749789156352 6.09370982346477
C 1.38620620506055 3.89524926245593 5.47708982073810
H 0.63399135300137 4.66651999383905 5.69879499150369
H 1.45255809217660 3.25301013774021 6.36539110947220
H 0.99528323432232 3.28909064611871 4.65100280140613
C 2.60544241363494 4.33273013307784 2.51185560582226
H 2.13231511203539 5.20508621705770 2.03763833705508
H 1.82375765881927 3.57650182193618 2.65264018647083
H 3.33298272018013 3.93607937825175 1.79128679476383
C 5.49552109938208 5.64994748476053 2.82515010148308
H 5.24002748125773 6.62365468043753 2.38190107854618
H 5.44862415357953 4.91057186516634 2.01465815312145
H 6.53626013577162 5.71314283590884 3.16576582283539
C 6.09263243453415 6.03649752214969 5.95422475068274
H 6.98085207521376 5.73038857284030 5.38613178360097
H 6.23155711997670 5.71178759492068 6.99248425341873
H 6.07869381808759 7.13616915575802 5.95657559707587
C 3.52312385490487 4.94181126191125 7.58612740387346
H 4.48047427407847 5.08582943771364 8.10068967084114
H 3.09980983605125 3.98599968007108 7.92370280586971
H 2.84166704848980 5.73054382272437 7.93689075375090
34
Entry 1 - B(C6F5)3 // -2209.331003144081
B -1.01890795159885 -0.53919035946113 0.00004671227695
C 0.54277520499585 -0.53916635448950 0.00547986216416
C 1.28667109521707 0.38307153769317 0.76082268708020
C 1.29194328721239 -1.46138820177081 -0.74465181472281
C 2.67912320306486 0.39091075883873 0.78797150813600
C 2.68455203129473 -1.46919542688226 -0.76208117581519
C 3.38079180588090 -0.53913528220149 0.01538425353863
C -1.79975389041052 0.81326105219411 -0.00008638753052
C -2.97544757437624 0.99656634059988 0.74730668734567
C -1.37051458050068 1.92306115051882 -0.74745122905668
C -3.67848017469694 2.19863638791150 0.76963393536710
C -2.05979568904869 3.13307077528637 -0.76963498706846
C -3.21855704822578 3.27120705167933 0.00004178665628
C -1.79966514889533 -1.89168700116261 -0.00528792780100
C -2.97011107480377 -2.07503111369039 -0.76085752716127
C -1.37557971500201 -3.00148028008848 0.74501788618315
C -3.67290903259610 -3.27713671939217 -0.78811171560438
C -2.06493351560289 -4.21152833350615 0.76236813813391
C -3.21829906645623 -4.34970373171843 -0.01536410277452
F 0.65731989461976 1.29013628760857 1.53486575457842
F 3.35175473899340 1.27359198903006 1.54049322784758
F 4.71656953186769 -0.53911914995300 0.02004232440996
F 3.36243805539831 -2.35186255944164 -1.50989097579309
F 0.66803164644253 -2.36846063797852 -1.52307678059920
F -0.27827495524597 -2.91446616850232 1.52370321349083
F -1.63947714711353 -5.23980302494021 1.51035229376187
F -3.88600675386543 -5.50662751064477 -0.02011700115913
F -4.77342228641099 -3.41855369215234 -1.54091763262113
F -3.44101591387426 -1.07658138149417 -1.53504125127115
F -3.45167593711007 -0.00187711335436 1.51823484976179
F -4.78421302511806 2.34002006739104 1.51475788676522
F -3.88633872658482 4.42809654556781 0.00011876867287
F -1.62919117666728 4.16134860887998 -1.51466349863559
F -0.26781150078303 1.83609301962538 -1.51847377855646
13
Entry 2 - THF // -232.551462357512
O 3.29321471751808 -1.77377823008622 0.23550792999422
C 2.15304687520256 -2.50474438324996 0.69862012953595
C 3.76065594785517 -2.50479351314735 -0.90285932470614
C 2.53317328592736 -3.99370044968616 0.57333761247213
H 1.94333861832638 -2.17966052598951 1.72577999451298
H 1.27286923229336 -2.27182996043409 0.06632506421835
C 3.63391447296370 -3.99372732699139 -0.52314394247910
H 3.13171317574645 -2.27191560375204 -1.78544510206889
H 4.78860888803123 -2.17972630287397 -1.10867114636130
H 1.66728154654232 -4.61212007572682 0.30177929214379
H 2.93074576842555 -4.37500312519268 1.52284639189586
H 3.36572411914419 -4.61219954124394 -1.39004716475607
H 4.58187861402360 -4.37499396162583 -0.12186754240179
82
Entry 3 - Borole A // -1927.35881006512
Si 5.60874279620215 4.49539496674244 3.76096938661527
C 7.43113532872527 4.93679395831814 3.78238604230596
B 8.30132565912897 5.43024509349392 5.01763763580970
Si 11.32147726232342 6.06149551582257 5.44371855379859
C 8.35176437959098 4.60527141181766 2.82843529806179
C 10.93758570456809 4.46417629642020 2.47309525093941
C 7.76264513498414 5.93564187309457 6.37662494577493
C 9.82876997517176 5.30681482977390 4.59596775356218
H 9.15611963518326 4.78715759347875 7.56519294367732
C 8.32280284045301 5.49354864570450 7.58983013414701
C 7.82992660539355 5.91653712448286 8.82813304857685
C 6.78634938451017 6.85308437844348 8.84125852347492
H 6.40562686990364 7.21086929078014 9.80239783625353
C 6.21681896037732 7.34413957951143 7.65779426376062
C 6.69693593010756 6.85276745977440 6.43981148217335
H 6.24135008725346 7.20417863155774 5.51086624290733
C 5.10485667421488 3.93655175087791 5.49402017707709
H 4.06820006463848 3.56647427408243 5.48089195066338
H 5.17418735013070 4.74566873616776 6.23210277301742
H 5.75011967677309 3.11584068711615 5.84131200055945
C 4.53644558972069 5.97123722275259 3.25782563133326
H 4.74251035855602 6.27556325136805 2.22137048218143
H 4.71018518345695 6.84366869248469 3.90372031594110
H 3.46869180634972 5.71203633529916 3.32559618004740
C 5.22217931566381 3.04922893999758 2.60741731199129
H 5.29316750933690 3.31274499139508 1.54490440211784
H 4.19944828327828 2.69220105588598 2.80500387805010
H 5.91146408051666 2.21130615968293 2.78753729799552
C 12.11149790978481 4.86107363276401 6.67499059951314
H 12.52454703943875 3.98062403096405 6.16168268484721
H 11.38773211083450 4.50440285074270 7.42162644430549
H 12.93551353710695 5.35164956056498 7.21556869508431
C 12.64521996954109 6.62776638672830 4.21953862997874
H 13.18313958613140 5.79597307065563 3.74820435453032
H 13.37955341456641 7.25699611679696 4.74614180547991
H 12.19981114957995 7.23206362357633 3.41570971187178
C 10.76617475985451 7.61019065379099 6.37270653375265
H 10.24650930859776 8.30488703934120 5.69603999072990
H 11.64278462662903 8.13401417830675 6.78374926921217
H 10.08152722758917 7.38032932753000 7.19893828784302
C 8.08442464164011 4.12283515968087 1.46762483329158
C 7.22322163890776 4.85604428083397 0.63203047549027
H 6.77237355105745 5.77096779768798 1.02054876026706
C 6.96433639082343 4.45017925198708 -0.67846463434744
C 7.58906962979053 3.28585907930342 -1.15165253383809
H 7.40468922068263 2.96251652279993 -2.17984026200451
C 8.45379248727975 2.53427370280771 -0.34688001956263
C 8.70111033070936 2.96961997143709 0.95949889192299
H 9.38349722012490 2.40300140252647 1.59453436276722
H 9.79159544081547 4.80715520200542 3.32454345215514
C 11.04460007262036 4.93402671258980 1.15543949884929
H 10.26739886488911 5.58596880281444 0.75434685495244
C 12.13224504821241 4.58163945212485 0.34906242569955
C 13.10980402985236 3.72947902854521 0.87671673724485
H 13.95377215038157 3.43479343956043 0.24695718400977
C 13.02777744860014 3.23262812890337 2.18681593012925
C 11.93554966032244 3.60771673298263 2.97125355871513
H 11.83063723916980 3.21782857498671 3.98527992930581
C 6.01904987462728 5.22926789058918 -1.55637815671487
H 6.37817284967002 5.27531726197255 -2.59422815944712
H 5.02374830589738 4.75727728778245 -1.57680337353494
H 5.88930865661252 6.25640595855638 -1.19035228022261
C 9.12344167531578 1.28944335092466 -0.86947522664152
H 10.20764306721367 1.32060372539275 -0.68689335711686
H 8.73536097265701 0.38978992102598 -0.36730645464851
H 8.95933707103409 1.16844788404244 -1.94835050718009
C 12.22869220272750 5.10265753780067 -1.06175253489881
H 13.04518396052681 4.61825929710750 -1.61332710017920
H 11.29115031931564 4.92805257440477 -1.60980841114238
H 12.41333924314434 6.18797971757482 -1.07193225665992
C 14.10495823603707 2.33545244106884 2.74002875477236
H 13.74703131044668 1.77351294227991 3.61290396936410
H 14.45674439824070 1.61621324737823 1.98690252186580
H 14.97900003274795 2.92419977179883 3.06131469062162
C 5.12075325640796 8.37754078031530 7.69870145810307
H 4.48847923843284 8.32250561642993 6.80207660013059
H 4.47916071936013 8.24936500716386 8.58164657222830
H 5.54051662304684 9.39524159391833 7.74336768677897
C 8.40006878486104 5.37750975560621 10.11473452604282
H 7.81749307363157 4.51335473455516 10.47214583652702
H 9.43713962628223 5.04123285842425 9.98025029197159
H 8.38350913171467 6.13477457021976 10.91097708996960
84
Entry 4 - Compound 1 closo-Cluster // -5777.735229232076
Si 2.70562366323973 7.17466443641242 4.80394135365481
Ga -0.36905106204308 5.37271870234483 6.46780903896650
C 0.87491043894070 7.20211701533332 5.22202001038009
B -0.30027947399924 6.67919403187355 4.33355993363126
Si -3.36566822437552 6.64719047725188 4.65778671665995
Ga -0.38598774576275 9.17921762854469 4.23874318312584
C 0.27022568108728 7.86370300606305 6.37382973899047
C -1.16981364404440 7.83100823873723 6.28520677601633
C -1.59854176063836 7.11691268823957 5.08667036549212
C -0.19888298273057 5.88738854777341 2.98197228650366
C -0.75705088168539 6.38921563356569 1.79430083526989
H -1.27927478830807 7.35122186284146 1.81490149012593

C -0.67147516882459 5.70229816494066 0.57705130090385
C -0.01817190224102 4.46345003446349 0.55620106239326
H 0.05141799360104 3.91078528738953 -0.38517068481567
C 0.54784145677949 3.91721525073214 1.71600793600653
C 0.45380099881149 4.64469406545851 2.90805107732914
H 0.90649222335753 4.22518428159142 3.81228648706738
C 0.99285632030672 8.49057748898304 7.50208167276032
C 1.87730845334215 7.73009805588055 8.28148600002272
H 2.02452520518226 6.67496489241984 8.03942569681193
C 2.56327058672380 8.29114354882524 9.36125470749867
C 2.33780645299892 9.64217036730495 9.66518086724857
H 2.86132128027138 10.09356080778006 10.51222851450742
C 1.45410240871177 10.42481086602397 8.91381924205558
C 0.79054641407832 9.83465962000936 7.83088217566535
H 0.09324168617600 10.42826651175581 7.23577998026242
C -2.05220586132801 8.45657870740407 7.29425416177894
C -1.97436643303447 8.09702765816200 8.64340250736323
H -1.25523473143606 7.33426082975897 8.94961466573634
C -2.78975746102801 8.70653073948464 9.60518777084814
C -3.69833927833415 9.68619002348546 9.18961294918146
H -4.33938559863778 10.16812950475056 9.93265103719256
C -3.80166629451225 10.06905249769713 7.84377003636442
C -2.96648056607910 9.44933129497037 6.91126698697874
H -3.01752791557166 9.74640873479520 5.86115892622256
C -4.42406284248410 6.42203185892563 6.20576026357550
H -4.61429412503063 7.36468217698459 6.73386778248479
H -3.93474363730978 5.73867772665216 6.91646283534610
H -5.39304188865872 5.97759423207045 5.93076048041855
C -4.14287355671818 7.95509793642927 3.53681076816798
H -3.54806039097101 8.08296168583230 2.62015884594301
H -4.20856783958862 8.93180899602167 4.03766378186090
H -5.16111961994515 7.66434794931093 3.23685260136297
C -3.35962270664864 4.99450552252049 3.75209541295331
H -4.39271466734498 4.66947956853728 3.55504143634412
H -2.87471553701287 4.21594764899204 4.36052935227855
H -2.82253464420111 5.04584844737788 2.79669263954779
C 2.90913724341278 7.20012861555765 2.93047360283662
H 2.47802437432354 6.31158120497773 2.45241964514718
H 3.97704792485200 7.25389434389094 2.66884378825386
H 2.41377399059514 8.08117478870541 2.49473218139747
C 3.51470413657851 5.60887275788729 5.48566412750291
H 3.02038397316488 4.71128368029650 5.08524640900478
H 3.45672838528816 5.56719057937053 6.58295083427520
H 4.57774069023086 5.55849700850007 5.20506385002186
C 3.59809410850828 8.69853070527581 5.47368043498903
H 4.61811126157591 8.74153798379351 5.06163568147258
H 3.66764820787811 8.70615228284824 6.56855823316094
H 3.07773621165180 9.61962208521238 5.16983757329911
C 1.21758945518960 2.56707577317371 1.68424021935508
H 1.91740310785344 2.44980183048485 2.52294329726729
H 0.47540716826757 1.75595200288290 1.75774679910000
H 1.77371425884679 2.41520980140704 0.74827039791964
C -1.24402238383120 6.29444217816793 -0.68551400574162
H -0.48257278471858 6.87688036108318 -1.22890530713466
H -1.60632644902941 5.51336971391661 -1.36847297723798
H -2.07937525198934 6.97318468441340 -0.46449923684726
C -4.80553884036333 11.10611885796180 7.41051646262254
H -4.52608213530285 11.55503635636320 6.44791581280621
H -5.80468153374518 10.65820643755153 7.28822500459201
H -4.89864085133875 11.91130289574874 8.15277354100435
C -2.67438240939046 8.31372162696966 11.05553424931379
H -2.87246788513441 7.24011695111176 11.19360649765540
H -1.65961873157739 8.50649816132864 11.43532787377683
H -3.38369642042139 8.87258287749824 11.67989456474334
C 1.20408131289288 11.87014581023671 9.25960751649654
H 0.14835341695287 12.03356375559652 9.52438069293500
H 1.82141650253023 12.19193635249955 10.10850614393238
H 1.42864349871169 12.52784832633503 8.40655665047620
C 3.54011515720862 7.47127658061309 10.16409401102614
H 3.50052098106508 7.72922997272818 11.23171502700787
H 3.33893657395826 6.39648598019041 10.06123858343484
H 4.57311355222511 7.64775151771560 9.82402143664787
138
Entry 5 - Compound 2 // -6485.692629538363
Si 2.76848534497659 7.04380441733489 4.83205552527006
Ga -0.36899225826844 5.34613741300494 6.42026353221081
C 0.92865456714820 7.14169785057209 5.18791401770840
B -0.24845307102294 6.64415243426038 4.28421305250590
Si -3.33107855891899 6.75062987972926 4.56095680105102
Ga -0.27312279613810 9.14484250473101 4.17489873525941
C 0.32262571531783 7.82423884548214 6.32381301683227
C -1.11761900486164 7.81953508080219 6.21626620087980
C -1.54806839332628 7.11687195976695 5.01579553886020
C -0.14986661739521 5.84437932150576 2.93926936349450
C -0.74385342862250 6.32061372973745 1.76071257116851
H -1.28527345171909 7.26975846825776 1.79925906316855
C -0.67477809530325 5.62431375605737 0.54811557249315
C 0.02027717980448 4.40642983547060 0.52860773014311
H 0.08552328253784 3.85028866966835 -0.40315150599783
C 0.63319585145993 3.88571510406304 1.67748024641339
C 1.40959175022758 2.56257205582520 1.67174334193717
C 1.39133547954235 1.87625083608036 0.29770767646408
H 0.36711570689257 1.63693024831694 -0.02334524758416
H 1.95528103035201 0.93357133662572 0.34830819874936
H 1.85772812260994 2.50262969970155 -0.47643451368208
C 2.87824558692974 2.84310701847392 2.05660593331310
H 3.45827528973754 1.90768309993477 2.07015797026081
H 2.95314226301630 3.30283443904126 3.05149236846955
H 3.34544381276868 3.52909902856413 1.33542478288315
C 0.78432694030924 1.60008071801086 2.70385114294976
H -0.26567199643568 1.39045577683556 2.45383796491137
H 0.81028202611148 2.02340869406538 3.71723444886224
H 1.33347249253103 0.64631903649697 2.72080261662196
C 0.53302816758305 4.62096240183330 2.86463062083352
H 1.00755905004899 4.23526972352002 3.77105003840159
C -1.36454241857865 6.20158750433419 -0.69468009381718
C -0.79291628895786 7.60466284373359 -0.98988427947126
H 0.28828691810613 7.55148223278371 -1.18255104934138
H -0.95047056264036 8.29004431534025 -0.14594951705498
H -1.28158095553536 8.03934649953027 -1.87519276684680
C -2.87964761221330 6.31308143962395 -0.41915008398684
H -3.39976868665897 6.73547909381890 -1.29247655441889
H -3.08561511358367 6.96061554270542 0.44399742203194
H -3.31026240942658 5.32420776404341 -0.20545134974227
C -1.16160658126963 5.32526910010531 -1.93975199493932
H -1.66931008839001 5.78372062099297 -2.80073949423284
H -1.58216749795092 4.31862112577335 -1.80202387654969
H -0.09683758039509 5.22216032548017 -2.19477377031055
C 0.99895998175246 8.47034599791737 7.46894151782887
C 1.83330797326615 7.74753549938745 8.32066283570322
H 2.03219987635304 6.69952885828387 8.08914035863908
C 2.39310654327074 8.33456334644371 9.46610802114048
C 2.07444808062207 9.66762542730376 9.74254196849172
H 2.47720420124987 10.13289382244915 10.64082548443097
C 1.24108762710556 10.42828630918262 8.90193202504400
C 0.72736651027203 9.81460378457316 7.76274796594949
H 0.07134545354603 10.36248768864626 7.08948868842777
C 3.32387773433341 7.50086292864387 10.35508089857809
C 4.56375741457423 7.09798276174664 9.52779075811291
H 4.28315948026917 6.50950044974887 8.64340067227838
H 5.25103867665108 6.49049476978453 10.13585965062437
H 5.10593418088734 7.98867753858569 9.17914451484578
C 3.79366847117654 8.27431765990463 11.59576084947596
H 4.36442304527633 9.17377270792674 11.32332378619662
H 4.45025429624004 7.63452201009631 12.20265981457258
H 2.94662950478962 8.57961027534044 12.22731470909700
C 2.58846410767602 6.22892870495246 10.82734544925417
H 2.25197625956547 5.61485333224021 9.98085379750547
H 1.70560440666246 6.48971169436497 11.42782354606638
H 3.25587587001884 5.61094498393722 11.44656644619170
C 0.86998144293741 11.86829507298623 9.27492696092296
C 2.14237656308553 12.68595504180901 9.57313706826879
H 2.80349423813793 12.71295234456287 8.69501230988761
H 2.71247219749221 12.26433175037294 10.41206820270334
H 1.87605084525840 13.72050434865547 9.83661348794865
C -0.03085225932983 11.82962157946465 10.52852533041085
H -0.31428052483134 12.84966397077869 10.82983430717556
H 0.48241928742726 11.35405068139876 11.37627538060953
H -0.94972749080533 11.26219376669385 10.32071245473729
C 0.09219302474566 12.56926290550503 8.14999954579489
H 0.67521500669799 12.61213564504932 7.21848099958545
H -0.14129221565585 13.60079942339741 8.45064984145907
H -0.85832290604258 12.05848473557785 7.93944299270864
C -1.96577385983592 8.46121685419800 7.24469603912830
C -1.89855851722744 8.03313013576142 8.56945527532021
H -1.26108747822767 7.17994468464219 8.80499108358764
C -2.59301787918439 8.69465007661043 9.59033398973133
C -3.38403596446023 9.79151860382596 9.23706703842529
H -3.93513953477195 10.31925411090760 10.01403989176860
C -2.46210752040780 8.18624369521635 11.03021368830032
C -0.96821875226921 8.08652808566174 11.40563058406867
H -0.42545225616025 7.40150872322124 10.74183242409403
H -0.86109050762586 7.71659970824281 12.43654071179339
H -0.47656746857979 9.06547187099155 11.33340308854246
C -3.15562855815229 9.11173523362232 12.04033394659828
H -2.73755253123298 10.12854443765559 12.00940122445455
H -3.01215371665948 8.72099795263885 13.05790649296015
H -4.23848080004751 9.17681559544984 11.86021709602060
C -3.10418661712164 6.78475520776734 11.11639357722225
H -4.17051390306055 6.82658293694821 10.85157208145417
H -3.01558616915617 6.38397594028981 12.13761454187900
H -2.61397606519468 6.08054021147116 10.42984632600281
C -3.49534575069761 10.24017735532471 7.90775257127848
C -2.77212639311041 9.56380106682913 6.92412698778852
H -2.80319988350145 9.89566417424347 5.88670171798203
C -4.40873890133677 11.42816130628545 7.58252115052465
C -4.02133370744292 12.64356419731151 8.45030507126821
H -2.98751965707110 12.95613202549902 8.24736103070931
H -4.10100455842188 12.42175956096532 9.52298061905725
H -4.68622604738132 13.49267878798547 8.23224195893492
C -5.86734063756904 11.02346205273232 7.88826386611779
H -6.55160739634303 11.85620927777370 7.66547668180789
H -5.99369805556639 10.75127400636997 8.94524606508178
H -6.16629849181509 10.15809110115870 7.27942546165420
C -4.31652577656381 11.84093705993611 6.10575724065282
H -3.29263346077249 12.13181188615963 5.82871380334133
H -4.97228371936411 12.70377194821268 5.92133593915083
H -4.63849737078398 11.03109242755046 5.43517905069627
C -4.42604759785665 6.60938545651689 6.09292242649996
H -4.55752241281599 7.56927471927916 6.60835429054832
H -3.99659289565539 5.90265586711561 6.81893193145482
H -5.41950717313948 6.23133568587184 5.80633655488740

C -3.99912703288709 8.12054056434648 3.44273222721531
H -3.38868767996617 8.21945748738518 2.53283677050414
H -3.99293054116918 9.09271823085702 3.95768659414229
H -5.03378268672110 7.91388172962053 3.13007912057001
C -3.41762883105104 5.10143449910875 3.65509057914001
H -4.46331387299172 4.85138656021388 3.41969292360942
H -3.01267796009646 4.29294499023749 4.28261474422665
H -2.84289486983647 5.11109258868481 2.72059470234481
C 3.04421178237700 7.05348070865731 2.96841129288247
H 2.57772769956275 6.19236242826625 2.47404196796403
H 4.12198999924237 7.04278346225352 2.74531754726637
H 2.61807167039188 7.96279460008545 2.51784397213204
C 3.48007117719667 5.44952619957326 5.55774060236633
H 2.97299238223627 4.56820931102586 5.13756830997483
H 3.35952726162469 5.41469336392162 6.65061091070064
H 4.55389476742403 5.35395566572052 5.33721334009263
C 3.69624611118459 8.52872040560557 5.53844896417613
H 4.73083433390246 8.53373187657201 5.16231637398147
H 3.72782744202856 8.52853672842372 6.63497221152916
H 3.22180381905791 9.47046796144635 5.22314828754954
104
Entry 6 - (Cp*Al)-Tetramer // -2531.113748677080
Al 4.49829371443774 3.66093081067934 5.71715868057764
C 5.15308105270179 5.50018588659149 7.03291231526314
C 3.96058064664412 5.87630356138354 6.33833325124789
C 2.90460010595710 5.01900675025691 6.76976540008504
C 3.44810823697877 4.10632555166066 7.72733336996508
C 4.83690961096376 4.40752432885120 7.89815854081268
C 6.45655597056127 6.22207131443691 6.89328848141188
H 6.85050418876854 6.16900675121246 5.86554739513873
H 7.22211129570238 5.81329186250699 7.56260642374821
H 6.33745356131980 7.28973769763490 7.13800515639595
C 3.87595953872907 7.03524120277909 5.39626025364240
H 4.21617003396705 7.96092128273275 5.88743112437908
H 2.84852468563203 7.20064561499297 5.05238943122059
H 4.50615441870760 6.89170061201455 4.50596241206259
C 1.47542747578212 5.03738709829834 6.32119167348707
H 0.78938957930139 4.97354191862482 7.17882537000342
H 1.24101174250216 4.19034696129095 5.65318268670614
H 1.23851798455685 5.95993805055786 5.77558779689989
C 2.66265456610680 3.10531779934253 8.51325895158453
H 1.91532384213857 2.59552182684444 7.89129842072436
H 2.12294699830815 3.59200344648898 9.34325139393765
H 3.31350836118691 2.33863373513193 8.95024397653810
C 5.75975933031775 3.69606185509621 8.84000030163407
H 6.77452927049563 4.11130129184739 8.79521078852561
H 5.83555708362286 2.62044882756625 8.60992292184672
H 5.41421841877411 3.78393506250913 9.88172575154281
Al 6.44062981756977 2.28984193608293 4.40894216483772
C 8.54917608115273 2.70701158401820 5.25865269868813
C 8.27822699017672 1.32213953879473 5.49000754163092
C 8.16084229768158 0.68348898193797 4.21911877710814
C 8.36542682517902 1.67015702567693 3.20366549391751
C 8.61046808460579 2.92379614299089 3.84532091629964
C 8.84526854268578 3.71904428690142 6.31883450095083
H 9.88100696367434 3.61605090639205 6.68425454992998
H 8.17967383515938 3.60623995587496 7.18491919363331
H 8.73134074874868 4.74177930892938 5.94012371632050
C 8.13989371234821 0.69340414735422 6.84263837129967
H 7.18617648937628 0.96452598703463 7.32822056445920
H 8.94934536085746 1.01543058298815 7.51443208578393
H 8.17679081085215 -0.40195187433332 6.78109047271375
C 7.94129904236725 -0.76898860116521 3.93825126373317
H 8.74820545625054 -1.16924689655472 3.30403992512182
H 6.99381411799644 -0.94912357858220 3.40827230668908
H 7.92159497023782 -1.35848038688362 4.86207779396415
C 8.39600064981223 1.36007555829528 1.73987474270633
H 7.44571209614769 0.92722352199071 1.38903363168972
H 9.18681762193269 0.62623992879140 1.51503945338715
H 8.59544345114834 2.25394920971874 1.13794824522557
C 8.90348283751181 4.23865105788630 3.18972769763905
H 8.83028946861794 4.16660184464809 2.09714712342231
H 9.91988634733365 4.58890390112755 3.42885726366942
H 8.20203748891976 5.02466875464505 3.51374566360008
Al 4.01609342702079 1.15792984318544 4.78172400034762
C 3.13668523167142 -0.40680711576780 6.30135127458780
C 4.08004681443671 -1.09979493091790 5.47865763672080
C 3.61116179472230 -1.05763575548764 4.13133201293194
C 2.37918130035097 -0.33190831679152 4.12142955328154
C 2.07815516553386 0.06255975746094 5.46359758979545
C 3.25961715675957 -0.29032319816006 7.78843248260174
H 4.14569732261243 0.29420958265492 8.08545330488497
H 3.35998087029860 -1.28541698133224 8.25019727330340
H 2.38173332053857 0.19357290206811 8.23185629142990
C 5.28469248058705 -1.81048201707889 6.00967171548728
H 4.99130636890493 -2.56891912055587 6.75329894256733
H 5.98472564871081 -1.12380247272304 6.50788085936126
H 5.83359237673051 -2.32461166495390 5.21244011911985
C 4.25689820036376 -1.65780465318837 2.92032121105363
H 4.60392715765374 -0.88568550117844 2.21281645166398
H 3.55360703813020 -2.30504623407832 2.37435527493236
H 5.12679284064209 -2.26978192605785 3.19084540231880
C 1.49905434745752 -0.13569778508138 2.92871036344942
H 0.89531319177851 -1.03729083618854 2.72960045849526
H 2.08547911630925 0.07143265522934 2.02365914720192
H 0.80443945539186 0.69998990023115 3.07546801715713
C 0.85470642175789 0.82093895840097 5.87924019320770
H 0.82770711032892 1.83381846475126 5.44349390800665
H 0.80568664477836 0.93311130282652 6.96972667101981
H -0.06271073522603 0.30323030999270 5.56016872834522
Al 4.28565027687523 3.23779323730438 3.05713620715475
C 3.71555170023826 2.96820877844326 0.81453477756673
C 2.60327649109932 3.60199406759376 1.45030140931957
C 3.03033957946525 4.88510555774928 1.91716799501022
C 4.40616631581084 5.04804469886863 1.57428301685939
C 4.83063692024135 3.86085725633309 0.89995367233249
C 3.74167346559778 1.62434765426571 0.15290361593945
H 3.98806217772279 1.70941880017980 -0.91689304892814
H 2.76826076849214 1.12319730649409 0.22645008360706
H 4.49033443806734 0.95554720354171 0.60882870462588
C 1.19646239126917 3.10349727835195 1.56213825345458
H 0.86881745782818 3.02086692910389 2.61085001924013
H 1.07624778301602 2.11742253314727 1.09905096645797
H 0.49861017462079 3.79290700412067 1.06018262972047
C 2.12124893107019 5.88036133924547 2.56635894518436
H 1.71967627313334 5.51314356020692 3.52252478409786
H 1.25910540067254 6.10017071603875 1.91648674790715
H 2.63645390600802 6.82726789542551 2.76447065817670
C 5.28401132254076 6.22943043966441 1.85293966488952
H 4.70152632335000 7.08476189758968 2.21856631525555
H 5.81396376506996 6.55340068932955 0.94451693529140
H 6.05082084126728 6.00242602789956 2.61357727958315
C 6.17600102453655 3.65134081681628 0.28234801907131
H 6.98026428990690 4.03567646698653 0.92388081496484
H 6.25153718726670 4.17246914913422 -0.68700881696368
H 6.37321690407894 2.58816060301217 0.10003403910484
24
Entry 7 - (THF)W(CO)5 // -866.886046163400
W -0.02469088084884 -0.42478813980554 -1.93022504523507
C 1.41501929390225 -1.44456971313155 -2.98414663047584
C -1.49035529150601 -1.51550130454395 -2.87219955621857
C -0.10564114601990 0.88156095574897 -3.41946129105559
C 1.43770476197991 0.72962542510153 -1.06166147459032
C -1.47022712459229 0.67467593933109 -0.96839219619975
O 2.22283897062754 -1.97457882524444 -3.62460921736585
O -2.31808071768949 -2.08869274881627 -3.44618003558515
O -0.15311988701861 1.64882383409095 -4.29399306126499
O 2.25921197162267 1.41701582118767 -0.61928088599997
O -2.28712233451600 1.33425129939466 -0.47711680737356
O 0.06884814708325 -1.92281198944025 -0.22177911900438
C 0.16390832893562 -1.56591438346809 1.18834386191677
C 0.03368193641520 -3.37059200167365 -0.38748410047881
C -0.11987001628147 -2.86053159659298 1.93899644060047
C 0.42261914401634 -3.92916296720329 0.97506059274187
H -0.98841549185741 -3.65208392065385 -0.68599686610767
H -0.56387438815516 -0.76637941892857 1.37650170836146
H -1.20187861942430 -2.98862010403759 2.08678524155839
H 1.17966513560614 -1.18201642956131 1.37223027579450
H 0.36687171938267 -2.88346191204528 2.92187296507080
H -0.00664398763656 -4.92310853112882 1.15196294031623
H 1.51620590767266 -4.00544734570719 1.06014479172724
H 0.72958663730179 -3.62745429387213 -1.19622824913217
95
Entry 8 - Compound 3 [1W(CO)5] // -6412.105878194633
Si 2.67951805188312 7.25929633390742 4.74672321191455
Ga -0.41100656396453 5.36642899326275 6.46608998241747
C 0.84708776627578 7.19409358176642 5.20279311666024
B -0.31920691470866 6.61182833534974 4.32115536354366
Si -3.40475388194787 6.71543764620505 4.53871183290777
Ga -0.39176111456705 8.84431515237505 4.26793691928587
C 0.22973039186882 7.85198756098903 6.35733107274204
C -1.21028924409123 7.81478695870417 6.25593248540827
C -1.62806007962373 7.10563128210507 5.04400463712242
C -0.18398239302264 5.82014480403067 2.97809180066835
C -0.69195580378923 6.32851251939728 1.77025004968930
H -1.21344561228456 7.28945602135396 1.76669311683644
C -0.53914805829822 5.65174190548536 0.55511752511887
C 0.13453612241336 4.42210405091548 0.55969543369270
H 0.26896928384627 3.88382020907205 -0.38290561964758
C 0.64598073725082 3.87005106366610 1.74037488489415
C 0.48333142341372 4.58593340904265 2.93326026826490
H 0.90609798967735 4.17047820086681 3.85395075783196
C 0.94607328026871 8.51110598206929 7.46739737868350
C 1.89641828237521 7.79476057047999 8.20902392426026
H 2.09563166156324 6.75166793825231 7.95414480130006
C 2.58184188123802 8.38848336239905 9.27127356279573
C 2.28702895150034 9.72259805446853 9.59041548389135
H 2.80908376892893 10.19828472041765 10.42491873184165
C 1.33502003790218 10.45895023402852 8.87584900783125
C 0.67050832412323 9.83732946863388 7.81121359243156
H -0.07970537198452 10.39192685768743 7.24708654641140
C -2.09773943827400 8.45479474393788 7.24943193834517
C -2.03052810123613 8.09037468137359 8.59780481268519
H -1.32524248154846 7.31590078427643 8.90762096775010
C -2.83483462973796 8.71860130376666 9.55551225652615
C -3.71800432249576 9.71882338713645 9.13269609850112
H -4.35084662007876 10.21649743751442 9.87221279773982
C -3.81011368781887 10.10387465356390 7.78723154221876
C -2.98552549040406 9.46471953137063 6.85591451325610
H -3.02563536011331 9.76300758082022 5.80776399725635
C -4.51955093983021 6.53827865866814 6.04875268765268
H -4.70047086603246 7.49113568652833 6.56073450242815

H -4.07963614251971 5.84661135653375 6.78313564131878
H -5.49034760038799 6.12124629679050 5.74000556152441
C -4.04014245272700 8.06712900234514 3.39059823151063
H -3.38937206245455 8.17526843867951 2.51071378554055
H -4.09194132842334 9.04333202190598 3.89205648241198
H -5.05060928295061 7.82543083474322 3.02801266406806
C -3.41247499717462 5.06154243901062 3.64037996275070
H -4.44672097202795 4.77326501025461 3.39842664825950
H -2.98405297332989 4.26786022826969 4.27122861999073
H -2.83332462647081 5.09086002110561 2.70916235698797
C 2.83021542577068 7.16428536014035 2.87471240600384
H 2.58944077357346 6.16511902220752 2.49062060954957
H 3.85849732697581 7.41637697598846 2.57524684295548
H 2.15804892152675 7.87613810326616 2.37629414983793
C 3.59463231637337 5.79211955765368 5.50336052890416
H 3.12442276921449 4.84503134862845 5.19893116051147
H 3.60898315764686 5.83122418920156 6.60134782835915
H 4.63910256767900 5.76998183225403 5.15737108208006
C 3.42828689214693 8.88990408145596 5.32186905123448
H 4.41449278185934 9.03138403186804 4.85507993122040
H 3.54847506024237 8.93982311782888 6.41117332235741
H 2.79657890404399 9.73798132081026 5.01940742510564
C 1.34072584812794 2.53254188516294 1.73071230628160
H 2.06353939229549 2.45146589449112 2.55430686636079
H 0.61590775117592 1.71088785735097 1.84746624355193
H 1.87531265666993 2.36408494026944 0.78564082878102
C -1.09524963291084 6.22162458356148 -0.72405636680705
H -0.36791392866726 6.14536078578575 -1.54521320439418
H -1.99757244809525 5.67367575760936 -1.03890897309720
H -1.36979053259145 7.27700284055184 -0.60381992289498
C -4.79166214277625 11.15954918900146 7.35081511195554
H -4.44974475256689 11.67536152014196 6.44514457588634
H -5.77042564051950 10.70859928570433 7.12084874396168
H -4.95374971687851 11.90578974174089 8.14054636634805
C -2.72606188514020 8.33613111963768 11.00880126059970
H -2.81910753104423 7.24852418667784 11.14372956326777
H -1.74754416955678 8.63071487594386 11.41845904226555
H -3.50438575751817 8.82290705799447 11.61063615428183
C 1.02133673862781 11.88735649450837 9.23393457665629
H -0.06080167828822 12.02969513341086 9.36934116333847
H 1.52756002199812 12.18837590047069 10.16033360104610
H 1.33664738124624 12.57004305761602 8.43159390654378
C 3.62691680080972 7.62282932875676 10.04067861357858
H 3.57571706013470 7.84210083652033 11.11642319322743
H 3.51068088165763 6.53931082543056 9.90425583540350
H 4.63968721169560 7.89257412450872 9.70155576644576
W -0.34518800380352 11.15481277818722 3.14630419150188
C -2.24924055492572 11.54691098257730 3.78788015240723
C 0.42489342614778 12.02467869087272 4.84068476169125
C -1.12496853004740 10.24030289853735 1.48321081782174
C 1.56775290767687 10.77259247496757 2.48825472452065
C -0.30346428106084 12.92403171129444 2.18646897645038
O -0.28267657233273 13.94228769737001 1.63077937112186
O -3.33254612331328 11.78914087391306 4.12688750366076
O 0.86000937084597 12.54273443516786 5.78125761605766
O -1.57914495006856 9.73262771208211 0.54476690725536
O 2.64464752273256 10.59328457534913 2.10377743328022
106
Entry 9 - Compound 4 {1[W(CO)5]2} // -7046.469047050506
Si 2.72110076196643 7.30619106770393 4.60302701728088
Ga -0.28809205094442 5.59816706185072 6.14391643138671
C 0.88949993870015 7.24380224578792 5.09150720919084
B -0.28088590172298 6.66301092518311 4.19468134607048
Si -3.35317840613326 6.57781217019394 4.50958157362157
Ga -0.42173399715595 8.88637176591159 4.20858211882260
C 0.27843997869793 7.88558458170207 6.26286188742701
C -1.16368963838532 7.81808450393844 6.18387600268012
C -1.58826173971742 7.08560327216504 4.98549086412953
C -0.15401454873612 5.82357381938272 2.88429847133854
C -0.61961522369065 6.30084975809901 1.64697159729120
H -1.06874573908116 7.29510829970596 1.58537079656801
C -0.50354981039656 5.54797250525629 0.47470064004876
C 0.08850464771499 4.27770498569654 0.55533374557879
H 0.18389067503928 3.67562751197603 -0.35246583431970
C 0.56312828750749 3.76171335719352 1.76593274756949
C 0.43996528617431 4.55278698137442 2.91658226358105
H 0.81471520044464 4.15417587273112 3.86513405433391
C 1.00431354930582 8.44027785928755 7.41839144189145
C 1.93397099698734 7.63069891271315 8.08456254159570
H 2.11422867246221 6.61463541834963 7.72747939428413
C 2.61483226989287 8.09486362422130 9.21166340304266
C 2.35744510907588 9.40192323028665 9.64982684863251
H 2.89176523559836 9.78364780087587 10.52379194979910
C 1.43179392730416 10.23117408290326 9.00354074644214
C 0.75296272064795 9.73292666074046 7.88509127662257
H 0.02397216581081 10.36032495786143 7.37324640796790
C -2.05311112146487 8.41461832698313 7.20019932005116
C -2.09649516994454 7.89230621371304 8.49420509853606
H -1.47857145487507 7.02998159022295 8.74520751070258
C -2.92502480378392 8.46256928944348 9.46734434943100
C -3.68929386480315 9.58337831226446 9.11864729618257
H -4.33337673135181 10.04184555715853 9.87364293035684
C -3.65111764003597 10.13620689401429 7.83028940589181
C -2.83095590808002 9.53093647372156 6.87347203625627
H -2.78588094835788 9.94043511252777 5.86277828947479
C -4.57038922007235 6.78719576909468 5.92260775175442
H -4.77520869415963 7.84173228827539 6.14530090659358
H -4.21438173786537 6.31274014671073 6.84654739826709
H -5.51945660702394 6.30380752649813 5.64374583964774
C -3.88337089965022 7.61922667553206 3.03732062440348
H -3.20807826546139 7.47819171356656 2.18251464482737
H -3.90413201844501 8.68836822614699 3.28951814715648
H -4.89660087705515 7.33618209611888 2.71485425401043
C -3.25377223305963 4.75792961612702 4.04711051093369
H -4.23960692506770 4.39146838855412 3.72454080617774
H -2.94450208341247 4.14988748097831 4.91065255219853
H -2.53689371593654 4.58077239722355 3.23455795285323
C 2.77489990676754 7.45134043616965 2.72927223254095
H 2.38246297645008 6.55314704636471 2.23582730968830
H 3.81291616415983 7.60549135685794 2.39884950182614
H 2.18705425059889 8.31093370584803 2.37500484676709
C 3.57014227124622 5.72022236596467 5.14663804218954
H 3.06661225655282 4.84068767953098 4.72048989062987
H 3.58154344076845 5.60758681317814 6.23876962532374
H 4.61439140723441 5.70654096595006 4.80029279858291
C 3.53745545456226 8.82486110839159 5.35323787595808
H 4.49481382063945 9.01373660531388 4.84505610901706
H 3.72959271783234 8.71273075058300 6.42727821813827
H 2.91241754342093 9.71915392207571 5.21921212678228
C 1.18953161143645 2.39396302900670 1.84561082549638
H 2.22172330151470 2.45136411061028 2.22313961691081
H 0.62938981290941 1.74493735190931 2.53510654473654
H 1.21487609083481 1.90652282661693 0.86242975566052
C -1.01595660761656 6.07798485893449 -0.83901215417895
H -1.22157820779773 7.15436080618631 -0.78086360702817
H -0.29148504647911 5.90887902102792 -1.64853251227047
H -1.95014867882380 5.57180021368761 -1.12934795882861
C -4.44623951360860 11.36819816923653 7.48624702585099
H -4.66294249309348 11.41650364814948 6.41111916316722
H -5.39717781422591 11.39811357787487 8.03538879436789
H -3.88583751434232 12.27924665890228 7.74957505982727
C -2.99980085122010 7.85943151771172 10.84503275638282
H -3.67692144414396 6.99078532653438 10.85278490495956
H -2.01418928551862 7.50594253199847 11.17718600171627
H -3.37799308766970 8.58197490672123 11.58014198100377
C 1.14084700408080 11.61916478886902 9.51049058322885
H 0.21856410050982 11.62512122183140 10.11268250468872
H 1.95501569775049 11.99380957512415 10.14466077992649
H 0.98994847598486 12.32278286981937 8.68103043445278
C 3.56876372182644 7.19660464224979 9.95299732271169
H 3.05642284917953 6.69742829333170 10.79028006099842
H 3.96918927925229 6.41065610638138 9.29878718658667
H 4.41191835777969 7.76260769582249 10.37200073744225
W -0.55455568131496 11.23459059866628 3.20223615742257
W -0.32334014575548 3.67309383965132 7.81835610995620
C -2.58541166206890 11.26344835688464 3.47170055644107
C -0.34656744153454 12.08126988079508 5.06079666164435
C -0.81434049999794 10.32240024258204 1.37742797173142
C 1.49050107241545 11.21332104074377 2.95359084478250
C -0.63822755111796 13.05495373591068 2.34473023889821
C 0.05363131360734 4.92857943242414 9.39764629150015
C -2.33615635218271 4.01689109810866 8.05081543110178
C -0.66035707067881 2.44302184177159 6.20075002558720
C -0.42743649033193 2.10042798448955 9.07203653235270
C 1.70469968612967 3.41422923311713 7.61412871517843
O -0.68562359449592 14.10153929024262 1.84893631947960
O -3.73339662028208 11.30152646092324 3.63205581309193
O -0.25669590114863 12.57269430630184 6.10619164445285
O -0.97652685777981 9.80821723105113 0.35256956446189
O 2.63760855688355 11.22274705781076 2.80420946026701
O -0.83486525391665 1.75046071486152 5.29006582865669
O 2.85104210144313 3.27310353910199 7.52977964090019
O -0.49385817015185 1.19242858038729 9.78966656437635
O -3.46614246421590 4.21802449499485 8.20444657673961
O 0.26817822828258 5.61716733944118 10.30445068013455
118
Entry 10 - Compound 5 [1(B(C5F6)3)] // -7987.108954100875
Ga 7.04354907143560 4.32339283923654 4.57639420756236
Si 3.93949230702799 5.00768426807627 2.63078204796276
F 5.33555228870381 2.19219539965929 3.98002378845456
C 5.70161067096812 5.44236434815169 3.18492062758579
Si 9.98932200721872 5.93270411725510 2.76317457357534
F 5.30342556215582 -0.08167563157030 2.55498807486460
C 6.06381884629804 6.44090074709420 4.20872038811364
B 7.12503551569519 2.84377565382273 6.20922157998101
F 7.05736871252583 -2.11230595554546 3.14871291171601
C 7.49692579445102 6.64486132840191 4.22544559864949
F 8.82500911901002 -1.80572267383947 5.21929228324876
C 8.15897310203092 5.80518199789677 3.23014944726351
F 8.85346277795366 0.43119244690536 6.67400613480102
C 7.30350194725782 4.01616614321573 1.27783676165125
F 4.80881778407119 0.97527433768195 6.80230808830560
C 7.67447164883785 2.68540003955044 1.52286741190675
H 7.75209690984735 2.32618614681852 2.55291291017628
F 2.71043634403818 1.56826417986150 8.32144189077444
C 8.00639875095511 1.79910279413725 0.49087842533453
F 2.51060074397358 4.00292709413639 9.56032655990702
C 7.96282989111052 2.26861361604210 -0.82708902924625
H 8.23209619248941 1.59314747809584 -1.64388081328980
F 4.47510122109509 5.88367596184403 9.16943116598674
C 7.59833005734073 3.58899799218016 -1.12262707008915
F 6.54074382420168 5.35989967829704 7.54757876212090

C	7.27835891156316	4.44246690325561	-0.06076692315656
H	7.00935785965556	5.47912753756170	-0.29244481166385
F	9.62991293884598	3.03147342741513	4.78418478317805
C	7.53673501402194	4.07040799321873	-2.54933867964352
H	8.36177124991243	3.66009016068038	-3.14856767790437
H	7.58423441469457	5.16657525062289	-2.60402502585803
H	6.59705738624177	3.75540055226903	-3.03053733770544
F	12.06586450456092	3.49378772711892	5.83082546025514
C	8.40134333234925	0.37757474942015	0.79419144028993
H	7.52139619002498	-0.28448282628472	0.79531429962686
H	8.87506407141162	0.29886978869107	1.78217948977369
H	9.10275059017987	-0.01289958487370	0.04439091676190
F	12.34686604454386	3.96830554522624	8.52083860704801
C	4.04866948699016	3.94872352073398	1.08442631124580
H	3.03021887481070	3.71356315180889	0.73896601317904
H	4.56964538334756	3.00140070443199	1.27927270710115
H	4.58147754687495	4.45119472141090	0.26693170841332
F	10.14809696147953	3.85156455169006	10.14859631865876
C	3.08966655748168	6.64202025021880	2.21086091869651
H	3.59522516615437	7.16081308774000	1.38110353773761
H	3.06950427132664	7.31898331574161	3.07676748241290
H	2.05020837683710	6.46240067456218	1.89670148417795
F	7.74264794754627	3.26985916901872	9.15873595681567
C	2.86739529319326	4.07346366757677	3.88093691359885
H	2.22117997094628	3.37137113445873	3.33295221259925
H	2.21892089034482	4.75870811052464	4.44171003655699
H	3.44894790033592	3.48261718142672	4.60002293800353
C	10.09511168761748	7.36949829925465	1.53586190703515
H	11.13515674653769	7.52123480376828	1.20858329642669
H	9.75099528511135	8.31308657346494	1.98556585160432
H	9.49330649791213	7.16855705871841	0.63601330617869
C	10.58521381099511	4.38098964081163	1.89281352365620
H	10.02657232573385	4.19263764675915	0.96696797829821
H	10.49705561868359	3.49110616909125	2.52812514693987
H	11.64834567274465	4.50699801189054	1.63568450597239
C	11.11023556562425	6.32363260366332	4.22187360257536
H	12.15938831118984	6.18622615286992	3.91979381176293
H	10.92138754171897	5.66387545711464	5.07714141313260
H	10.98326311040741	7.35796867335714	4.56748258602725
C	5.10387496239893	7.15525451436506	5.07545342507013
C	5.15766455637932	8.54881323614830	5.20314893078478
H	5.94602057165130	9.10607540074606	4.69727577916153
C	4.20274125663184	9.24507935340442	5.95369942337182
C	3.16839409173629	8.52217066711060	6.55774881311183
H	2.40263005947502	9.05870544028083	7.12422534261466
C	3.08820670062472	7.12673483231485	6.45080357435179
C	4.07505820752885	6.45777607795736	5.72247053045306
H	4.04103122505830	5.37271121060592	5.65137565639188
C	4.30214094918107	10.73998409059447	6.11316428705677
H	4.71765746926001	11.21286539746652	5.21257741960978
H	4.96669857784523	10.99687223649039	6.95331370300174
H	3.32125835359484	11.18894939924583	6.31878016170502
C	1.95586387211715	6.37216369085983	7.09586029381097
H	0.98293126377534	6.75332977878082	6.75147905566246
H	1.97683256608850	6.47807495885046	8.18998013671672
H	2.00191914609188	5.30180658081638	6.85841930417212
C	8.23788802590422	7.53557618684738	5.15265086707872
C	8.54391143759034	8.85186130222599	4.77964969210674
H	8.18357105901954	9.23590880368204	3.82117546227966
C	9.30940228522329	9.67994122946167	5.60485260587356
C	9.77494369224571	9.16049715493243	6.82174945143101
H	10.37776906214509	9.79580489038338	7.47607876959052
C	9.49407021396357	7.84840235438441	7.21640904267439
C	8.72224552130310	7.04552228645839	6.36724643278263
H	8.51509312294649	6.01419561418225	6.65234523349230
C	9.61623095569385	11.09997593784023	5.20527183387638
H	9.34776815425556	11.28712078716486	4.15714087844069
H	10.68432523601519	11.32859920263842	5.33092358399000
H	9.05622314850820	11.81515680003774	5.82766995179788
C	10.03797201911155	7.28558528460091	8.50245819339734
H	10.95469926323825	6.70439855993253	8.31562749339967
H	9.31463797383508	6.61065917121719	8.97902691304903
H	10.29202184113632	8.08131403565565	9.21486943391687
C	7.12232238064104	1.45474485718506	5.37043383367185
C	6.23119516152568	1.22229236522714	4.31737126348277
C	6.18144746093498	0.05518068389317	3.56390206226642
C	7.07156988573839	-0.97672749022881	3.86241629231103
C	7.97506603777541	-0.81094623593622	4.91142811732550
C	7.97978241543465	0.38028214615556	5.64300923890326
C	5.79719738134865	3.14833217005755	7.07740075681824
C	4.76862191081579	2.22454740271768	7.31845198127327
C	3.66222764308356	2.49828228251995	8.12858676367664
C	3.55656338512857	3.73423893011618	8.76329154734649
C	4.55366264600366	4.68838652491446	8.55951968530421
C	5.61906614632651	4.38221196665009	7.72042389977733
C	8.54822655100750	3.18086813515845	6.90262734493779
C	8.75893251497026	3.37576207919956	8.27370072956956
C	10.01277067321527	3.65257192764774	8.82568968950064
C	11.13613725840338	3.70976377919213	8.00325561630329
C	10.98734110478028	3.48494349708939	6.63373578021821
C	9.71465629045189	3.23955569287790	6.12797833350015
B	7.04151193757923	5.02092029026935	2.45425014754832
Ga	6.63783497261158	7.43731062377157	1.91891250291148

152

Entry 11 - Bis(Borane)adduct 1[B(C6F5)3]2 // -10196.466843601504

Ga	7.07862712371191	4.40615173445825	4.42960433960286
Si	4.06838602085665	4.95559342027961	2.29811987947912
F	5.61171041359606	2.22810002815014	3.61477593600002
C	5.78946988848589	5.52195950139547	2.91889328773694
Si	10.09237438638986	5.94771684227261	2.49029691516847
F	5.81425100170908	-0.09864806240630	2.29028317272432
C	6.12184871382004	6.52112601368969	3.97541114685975
B	6.94463565331616	2.92035068871034	6.14516592410828
F	7.38491259581149	-2.12010646050359	3.28365479980770
C	7.56685084701335	6.72180823693771	4.01465753585409
F	8.73172080913228	-1.75241909348596	5.63886479005661
C	8.25459781549073	5.89015151869457	3.02538430249478
F	8.52434952112828	0.54061778027442	6.98897217821856
C	7.52650613180725	3.91610672408624	1.23376336886401
F	4.44917892444259	1.22024463860304	6.25050815551506
C	8.14061964008577	2.74444843521112	1.71595459177335
H	8.31395200513578	2.62301009655557	2.79029771065939
F	2.18824994138012	1.90700418245831	7.48100742255361
C	8.58486433765065	1.72359591593917	0.87027395476648
F	1.97407210176183	4.30394016818886	8.78316904365577
C	8.40313039001128	1.88311246916459	-0.51002499243084
H	8.75045944287267	1.09999496928954	-1.18964029405270
F	4.07685305103860	6.06029212669223	8.74840198279310
C	7.80135165103652	3.03064377457801	-1.03751735732714
F	6.31016516908337	5.47045072975756	7.37569785523477
C	7.37923715542613	4.03344794979524	-0.15460897810465
H	6.93296183154946	4.93302918188939	-0.57480486148879
F	9.60588895573966	3.25342035626856	5.10746681533459
C	7.58951390844189	3.18671559008272	-2.52108995739645
H	7.70022341650290	4.23460378041269	-2.83219452588015
H	6.57348467550829	2.86994824508013	-2.80549140564564
H	8.29904754041411	2.57689374811588	-3.09629110929260
F	11.82700935730296	3.88786764061487	6.49747338116191
C	9.26134602081877	0.49723367194390	1.42496197455401
H	8.64715054833174	-0.40287053881383	1.27052466637601
H	9.44877928665521	0.59944641172013	2.50203140654305
H	10.22617909451528	0.31687005409098	0.92870609156883
F	11.69591367550278	4.21344925926109	9.22177670216836
C	4.26520427229534	4.02573340605320	0.68195494937407
H	3.25857038569257	3.72600721225755	0.35018980260776
H	4.87220479488196	3.11865183412889	0.79218821238847
H	4.71020609011066	4.63269036963557	-0.11549959883087
F	9.30316706684230	3.89176578500078	10.50910584199892
C	2.99404288020714	6.45872705836680	1.94199695102546
H	3.47210151891972	7.12224782969082	1.20908962431763
H	2.76831573056321	7.05321871624170	2.83499483651003
H	2.04358528258747	6.11395218203219	1.50646185053667
F	7.09261769711343	3.25446100892651	9.14962126660123
C	3.15929997982271	3.77529239056334	3.47908003159533
H	3.02453349921862	2.80379079749468	2.98404672691629
H	2.16218309152266	4.17208401582234	3.71904710219479
H	3.68067133624299	3.58159397929712	4.42439988543965
C	10.04723854514699	6.19880395629255	0.61996389646900
H	11.03502393310180	6.52481719640218	0.26162190141432
H	9.31096764858058	6.94474386752508	0.28936387301102
H	9.79419207313614	5.25317420370253	0.12192201746527
C	11.03762774269804	4.34930660506372	2.77192376871946
H	10.49394083943547	3.46638417353782	2.41628627091418
H	11.31615155108292	4.19890296055969	3.82048323892039
H	11.96765600444327	4.42699667836142	2.18640582638246
C	11.07521478445155	7.30548545715797	3.33743392005114
H	12.11187527601844	7.24442598410141	2.97102335675736
H	11.09461653850365	7.16819057080750	4.42723363259773
H	10.70214437125295	8.31316923931929	3.12952558157985
C	5.15914647447609	7.16461430265895	4.89979883121625
C	5.43659302614122	8.37327533835864	5.55999871343768
H	6.37293095436873	8.89142380431592	5.38691319445361
C	4.53216975063247	8.94048003806197	6.46029361748990
C	3.29404966528061	8.32005615317632	6.65881817446697
H	2.57071660361209	8.76746176409700	7.34509586122399
C	2.97478406762485	7.12491872087649	6.00734111325677
C	3.92825783426914	6.54861696599987	5.16386680177979
H	3.70380202224569	5.58229558498794	4.72581110776995
C	4.90285191545406	10.18955975370622	7.21513916954842
H	4.08063512885043	10.91764276506343	7.22362817135426
H	5.78779489099403	10.67042097398088	6.77767793162671
H	5.14282790886192	9.94955985387763	8.26265874702512
C	1.63302881571049	6.46910587683580	6.20044490590663
H	0.86846569179512	6.95832660005813	5.57640911284956
H	1.29550388203352	6.53739894094897	7.24312291941020
H	1.66054387834780	5.40933774471429	5.91477399578752
C	8.29302986981438	7.61295445081362	4.95226843119629
C	8.50282390565625	8.95961413780288	4.63076854240011
H	8.10322869342647	9.35706139265220	3.69698704020870
C	9.19763685151658	9.80732290222414	5.49609611548132
C	9.68138551989246	9.27675828225443	6.70050194464820
H	10.22579223458191	9.92881666394538	7.38823673797128
C	9.48547771283539	7.93520929015110	7.04793008560428
C	8.78870631258631	7.10816035811531	6.15596844794095
H	8.61907359217733	6.06101586108281	6.41570607405660
C	9.39010137337736	11.25997291343279	5.14947100716635
H	9.44275632319098	11.40711497057635	4.06278279948021
H	10.30933920270939	11.66083424975090	5.59679267222483
H	8.55240833540369	11.86947572888656	5.52384167352384
C	10.02305012925710	7.38042463004075	8.33985240149479
H	10.96270672927233	6.83166558420490	8.17192641800351
H	9.31107148435303	6.67966895198611	8.79660605965539

H 10.23328285985921 8.17952448900306 9.06234536909102
C 7.07875250831200 1.52347759474190 5.34860990344238
C 6.40477141508333 1.26193922470404 4.15063004419348
C 6.48295084404125 0.06895252080362 3.44226920617351
C 7.28080612734029 -0.95953065823335 3.94543213429017
C 7.97226005386233 -0.76307082174792 5.14043650380831
C 7.85901944396300 0.45535121485099 5.81585050460793
C 5.51194732329438 3.31343121704966 6.75308711712844
C 4.40552134343885 2.45062044381762 6.80659846803509
C 3.21444246271797 2.77371692112159 7.46120991885851
C 3.09901941671465 3.99551189078733 8.12249937575431
C 4.16758675750976 4.89293387407080 8.09376993322955
C 5.31849597279048 4.54873538783257 7.39411265760209
C 8.23000206061719 3.29007334014732 7.04278208081630
C 8.22384646291220 3.44064924956473 8.43598822414589
C 9.36807427242675 3.74941845057563 9.17490079627937
C 10.59277175423143 3.90571095191397 8.52530555046868
C 10.65572050417760 3.74154701467292 7.14136717141070
C 9.48936443308534 3.42663912170483 6.45119320785060
B 7.16287518661323 5.05050965928091 2.26056398952687
Ga 6.80818046834325 7.29326495005873 1.80656006870042
F 6.25708316884790 6.96124909342321 -1.05171338139200
F 4.10683399835022 6.03794579249926 -2.37259794991670
B 6.39818075383853 9.36592770554955 0.56402271240530
F 1.61728516505319 9.81755927650832 -0.96749741486240
F 3.74827430265036 10.77143365598607 0.32603315561867
F 6.60954308015977 9.76515050997566 -2.38938841108413
F 8.88836656253450 9.93458022876812 -3.76249127258630
F 11.30700742896823 9.80513882710008 -2.48705531700147
F 11.38823447588441 9.48987497540187 0.23942936026210
F 9.10666742095498 9.28088127645622 1.63995961470160
F 4.32905897245557 9.11061188444390 2.58385074874077
F 3.68856267588376 10.94006184843770 4.43966130032875
F 5.30968264653265 13.11243111035774 4.84959136758189
F 7.45024880001351 13.53021053796985 3.19655966801279
F 7.97056977477508 11.86477765356135 1.19323512086380
C 5.09515549091542 8.87134870451339 -0.23076854294454
C 5.11063830619932 7.69057005679697 -0.98551284205672
C 4.01845115765122 7.18732047500839 -1.68308966119373
C 2.81980490786046 7.90309801856396 -1.66529140870863
C 2.75644576995544 9.10830822001427 -0.96547001168828
C 3.88092886077461 9.57204758358272 -0.27900915829757
C 7.74247429412327 9.47541670493592 -0.29506714265598
C 7.75823661786262 9.65310225826400 -1.68797317146094
C 8.93530243633242 9.76200536721965 -2.43193458373464
C 10.17088661490624 9.70530737298129 -1.78650746152941
C 10.20888915328966 9.54870809165823 -0.40000631729737
C 9.01097054269173 9.44348258416386 0.29640104420002
C 6.18817794707048 10.36766060241501 1.79319125405651
C 6.94668447838231 11.53106872658901 2.00832135091958
C 6.68204778120160 12.43979653154635 3.03564844601258
C 5.59335735138751 12.23195161581971 3.88073502083803
C 4.78771486041725 11.10750129526846 3.69118323722845
C 5.12176603080211 10.19867398896161 2.69340356506122
F 1.74942464844499 7.44510830009015 -2.32544062147484
166
Entry 12 - Compound 6 Digallane // -7705.153042811474
C 8.92592726530136 3.45038665569386 5.23781978835476
H 8.64387559446499 3.50259018226653 4.18234572703255
C 9.29548442051147 2.20923276517117 5.77740136216877
C 9.64131918307791 2.14779669769476 7.13070031760838
H 9.92061124495600 1.18485976601883 7.56782309902557
C 9.63176253335028 3.29192051233997 7.94240845950027
C 9.25716813905638 4.51096465686490 7.36815693749592
H 9.22902509640564 5.40266383572136 8.00117961718196
C 9.33104355491007 0.97676369190239 4.91000802775002
H 10.23467386961165 0.96363096121084 4.27990482534112
H 8.46487127053855 0.93842722381724 4.23393689755177
H 9.33567968493680 0.06005996130338 5.51499179693891
C 10.02863334761705 3.20087694012736 9.39354055832664
H 11.11363407529832 3.04188434687071 9.49695062865599
H 9.52791310452959 2.35907222792449 9.89383138490643
H 9.77518708313159 4.11953162182402 9.93676770415912
C 3.84585311437741 6.56794043784867 5.38500411706798
H 3.53561260154954 6.80520836353666 4.35927814298818
H 4.03216578035417 7.52106926259980 5.89922943116706
H 3.00534349818350 6.07475938256920 5.89677529825792
C 5.15170984344838 4.01138022236520 4.23769980767752
H 5.06302119002188 4.36010557207757 3.19893259227985
H 4.24740692506274 3.43198436584004 4.47802627140455
H 6.01599914968862 3.33412493622066 4.29895056962043
C 5.54760281835323 4.77349822217260 7.17930955733636
H 4.68209091146139 4.14712225060273 7.44285928032388
H 5.60164851855356 5.58870115787805 7.91571404333074
H 6.45681972134651 4.16514486622189 7.27738070093541
C 11.94732809208583 6.28027695558159 3.55817620294724
H 11.64730950829386 6.56110133866277 2.53860610246614
H 11.64636541190502 5.23556321931266 3.72575357376014
H 13.04513303640452 6.33267738620657 3.61380236414382
C 11.75315708090860 6.89970426651339 6.55406325925791
H 11.44716991643274 5.87532103927947 6.80503368643818
H 11.35413289221538 7.57703900064509 7.32338367770460
H 12.85111379844334 6.95413945557696 6.60458845017994
C 11.65627590625961 9.19780772664938 4.56975377168692
H 11.55634540548049 9.51132994026040 3.52297140278338
H 12.70509814950378 9.33526546915638 4.87466659554499
H 11.04397221608273 9.88024006086570 5.17658641746943
C 5.88328513444235 8.15210197881249 3.43043318301167
C 5.35305795300345 9.41409393243661 3.73491081242480
H 5.77954866830576 9.99148750775482 4.55809273992340
C 4.27249293665890 9.93806904637060 3.02058500013620
C 3.72317622321421 9.17120776313513 1.98143942978732
H 2.87398055553353 9.56867884095805 1.41885051349377
C 4.23503547649498 7.91496515052972 1.64471553872324
C 5.32047204839985 7.41922019676172 2.37921869598840
H 5.74208609403747 6.44242517180165 2.13350312694638
C 3.70140400396021 11.29094581202670 3.35313709542586
H 3.71260940950240 11.95041472886440 2.47241456016398
H 4.26540959131872 11.77948428924689 4.15624951325530
H 2.65598560993967 11.20995305656043 3.68662551283898
C 3.64149971918879 7.10332309954961 0.52198589958527
H 2.77492083058603 7.60991711494503 0.07742343751321
H 3.31259878448105 6.11500939442335 0.87704023642027
H 4.37955528822747 6.93074700208345 -0.27614304986445
C 8.77893807105715 9.09755134370417 3.11899834782438
C 8.49496091204123 10.44936820493883 3.35906716570270
H 7.96570926558655 10.73103476045693 4.27156221873761
C 8.89410256532263 11.44259006895582 2.46048852280938
C 9.59266854767334 11.05945322450253 1.30519906891337
H 9.91362812961885 11.82936952770505 0.59821797440127
C 9.88261062395073 9.72006804461962 1.03137454749948
C 9.46315466987999 8.74797201431849 1.94885553887098
H 9.66836286146959 7.69329827266600 1.75477314019197
C 8.58664253253541 12.89483816968476 2.71291757860396
H 8.01864063985429 13.32820287328567 1.87604786308576
H 9.50925630520517 13.48456180975069 2.82155347430700
H 8.00082316657976 13.02885281999151 3.62906471469578
C 10.62956938745854 9.31917152111562 -0.21470118662799
H 10.03084641351947 8.63396459258266 -0.83366270733979
H 11.56482070714382 8.79529588527548 0.03479725048078
H 10.88455234234700 10.19408671521196 -0.82683874675175
Ga 7.93073086809047 7.75220337750718 6.35323398777254
Si 11.14724751867962 7.40267025994043 4.84649415042591
C 9.28846080880154 7.15125257642940 4.75530597401836
B 8.42942306288627 5.94897033074023 5.32224015187252
Si 5.36683050152696 5.45384597574812 5.43564440588426
C 8.37582723028466 7.99691645302868 4.02135655407709
C 7.02177543345541 7.54553600865601 4.15460398899192
C 6.94383159215947 6.36527040762752 4.98035331365655
C 8.89697037394326 4.62143962533372 6.01183874554519
C 5.44204804159226 13.92264061228400 8.20761488515191
H 5.44714490805505 13.76631158586669 9.29008586552491
C 4.83046059807830 15.07606774373519 7.69514653745413
C 4.81385541272936 15.26118995809337 6.30903458627798
H 4.32911053378131 16.14953335950823 5.89403023914883
C 5.39811917395055 14.32988347324249 5.43823946226363
C 5.99838208045449 13.19177274333329 5.98742705280872
H 6.43684933580037 12.45175240096969 5.31165476365001
C 4.22153122653666 16.09777123414249 8.62134253487753
H 3.73022886060576 15.61745024879692 9.47932027528305
H 4.99290278859267 16.77320101662338 9.02486458079807
H 3.47865918192489 16.71850354422558 8.10219595891083
C 5.38634504739438 14.56550116759440 3.94959349544768
H 4.39661074726170 14.89791341493095 3.60427630942085
H 6.10899202693337 15.34786723293685 3.66836246888407
H 5.65246565280189 13.65590587479003 3.39710732585065
C 11.26876620655298 11.24745969845388 7.96292090585040
H 11.58852124555939 11.00760925549132 8.98481183748327
H 11.11812359155014 10.29521011274301 7.43418000127361
H 12.08973717277351 11.77942957580543 7.45806821933276
C 9.84752685613994 13.75640698133683 9.10937238367344
H 9.98999519549502 13.41438747631457 10.14424939147674
H 10.69810776684532 14.40357119725885 8.84724767958957
H 8.93202647457674 14.36534901921863 9.07545172530504
C 9.52962180190912 12.96904039826552 6.16491937526557
H 10.38484823302862 13.61330936061580 5.91082434136180
H 9.50973370344400 12.14557551102266 5.43610032363101
H 8.60901172670090 13.55576777412878 6.04631553007435
C 3.15149484159654 11.17233240989148 9.84484115759006
H 3.49349594691015 10.90988770589190 10.85610253216143
H 3.36043759396055 12.23906519622449 9.67842733901492
H 2.06078047963492 11.02967772479930 9.80957281092074
C 3.39632959153644 10.65458169382261 6.83411770256332
H 3.67551803946645 11.69421596811269 6.61599655897938
H 3.81984880009626 10.01506670898145 6.04578408255337
H 2.30073927937163 10.57069483157854 6.77453399837477
C 3.57905963157900 8.30469122295163 8.77745568869985
H 3.68739150681020 7.98718121918072 9.82250864676080
H 2.53908200185824 8.12316359014031 8.46574988522340
H 4.22378514812519 7.65507306627565 8.16928752811850
C 9.30726948077118 9.61614397147437 9.93267776063001
C 9.89930661397418 8.37419339015677 9.66241889124768
H 9.50563177984395 7.75878583763832 8.85095323162166
C 10.99555450642425 7.91998886155643 10.40084960674954
C 11.49909127399877 8.73743294147701 11.42446341493508
H 12.36016104514471 8.39369253345670 12.00413278720624
C 10.92511232744667 9.97524448960005 11.72656483091263
C 9.82308556355067 10.39882321445173 10.97239662067481
H 9.34978837650735 11.35753610759262 11.19349150598628
C 11.62946131242170 6.58366847131481 10.11853243558710
H 11.59335278823522 5.93580695136018 11.00739758338825
H 11.12456032730233 6.06399104314928 9.29668578859259

H 12.68776303015239 6.69544223907447 9.83917368060679
C 11.47274240833279 10.84448319870307 12.82924076003147
H 12.31524286876376 10.35962800070489 13.33944842764859
H 11.82556400517533 11.80964706870385 12.43476702200647
H 10.70080358457569 11.06591933676209 13.58151047547803
C 6.46827764917166 8.50207214649580 10.22664128670401
C 6.80487343914720 7.17051705774004 9.94367606437133
H 7.33882736625813 6.93641719323667 9.02018401346417
C 6.44771414248569 6.13300037788763 10.80886990248699
C 5.74113607520231 6.45152580944061 11.97889112775001
H 5.45155987269038 5.64725861069882 12.66074905313246
C 5.40099457913836 7.76936689202876 12.29689154436246
C 5.77512769913051 8.78658513412525 11.40885938131525
H 5.52485199747423 9.82495799888600 11.63550538823876
C 6.80394544061457 4.70305099423511 10.49997960572846
H 7.38978941446197 4.25767796578580 11.31797813698300
H 5.90093818275339 4.08737930843673 10.37269758371467
H 7.38813757120373 4.62429674206509 9.57578601407289
C 4.65046851232687 8.10065136996145 13.56120509910976
H 5.25666941986076 8.73268926538969 14.22810067455494
H 3.72660532108406 8.65637336193756 13.34105117804234
H 4.37711315752373 7.19203672934740 14.11342005859931
Ga 7.22088866265262 9.90847121213441 7.00695862512684
Si 4.00990656075282 10.12430307442194 8.53065012839907
C 5.85675177211615 10.44824126653834 8.62871769712323
B 6.65094520313378 11.68481993669956 8.04746814716872
Si 9.70611830994586 12.30307772258288 7.91460191130495
C 6.81221195514942 9.63955712950505 9.34558893267852
C 8.14214445133171 10.15289169896378 9.19603435070857
C 8.16143788897177 11.33393326875096 8.36407284290499
C 6.03539279829105 12.95976287110174 7.37501270995601
162
Entry 13 - Compound 7 // -7676.095944635204
Si 6.41078320397557 -2.50417836679148 3.48686796871550
Al 3.08182740893214 3.49970389928071 5.82948574387365
C 6.77272060074861 -1.45713933594222 6.24882497903953
C 7.24214243642424 -1.55531245701766 4.86857574607089
B 8.66371861341857 -0.91103010186755 4.83611311037282
Ga 7.10026164822637 0.59779790731919 5.30668726809913
Ga 5.41031213884832 2.41699814706822 5.47850008001382
Si 10.32308984520152 0.70527777870622 6.82640374719403
Al 4.58139295725992 3.86523156011374 3.59072099278126
Al 5.37054374879377 4.90874054885701 5.99005925693845
C 7.72051448197517 -0.74171198789426 7.05779302059785
C 8.86812534842308 -0.32865637333289 6.27112844989033
C 9.67872328569073 -0.76497820634197 3.64604837510051
C 9.51943200277772 0.17678395219132 2.61741616185972
H 8.63659900108879 0.82216409260173 2.62111994044097
C 10.46508109037528 0.34118366520423 1.59859627663880
C 11.60179283781046 -0.47556840309199 1.60542821504571
H 12.35037209163108 -0.35917895332950 0.81621899510517
C 11.80127857626395 -1.43673470867830 2.60632722685315
C 10.83748529327031 -1.56125248121526 3.61435261229816
H 10.99318587027496 -2.30311224861557 4.40290554077267
C 10.27592121816724 1.40034083270602 0.54193162910587
H 10.90568495449999 1.20993745255948 -0.33789418690434
H 10.54004671380551 2.39764793252242 0.92940537196375
H 9.22882227427417 1.44811220846712 0.20826643842265
C 13.01491440028936 -2.33121339858873 2.58181600729235
H 12.85908560592096 -3.19137153886456 1.91066191268534
H 13.23548271245436 -2.73046260908563 3.58126710280954
H 13.90430125491881 -1.79457215817888 2.22142023777484
C 7.56188492908890 -2.62010649076809 2.00047702767846
H 7.10401250075165 -3.26681807569508 1.23552521113855
H 8.54001039096612 -3.04067913210304 2.27053697650694
H 7.74976206461769 -1.63589070261830 1.55028561783943
C 6.02842697241724 -4.25649634722373 4.07834254636893
H 5.57621868289041 -4.85168490714197 3.27007279649641
H 5.33511454300502 -4.25155250137253 4.93066398586024
H 6.95145343930573 -4.76323065014299 4.39653740187668
C 4.79626915929761 -1.70831166236474 2.85039006951610
H 4.73264352174218 -0.64536853792330 3.12669705471739
H 3.90686380563507 -2.21530745584186 3.24961639682185
H 4.75016373413644 -1.77179219893509 1.75248444779314
C 11.76436671309275 -0.43514346538573 7.26723869258754
H 12.04931977305906 -1.03942749586958 6.39393127581647
H 11.50073729107616 -1.12259906519621 8.08424662707955
H 12.64600571119183 0.14319251681974 7.58377820709313
C 10.88544115056773 1.82261500287281 5.41192489132834
H 11.29302610452230 1.23627574163226 4.57809383081272
H 11.66440949966419 2.51711250345773 5.76271159339593
H 10.05089734955630 2.41887583856688 5.01560335302663
C 9.94510274364067 1.80256420575823 8.32297839484521
H 10.67187899672468 2.62781520232015 8.37602911095109
H 9.99514199959025 1.24128002973778 9.26470941468503
H 8.93881142803232 2.23822222571117 8.26474284867863
C 5.54544914753660 -2.06828881968670 6.79503913745535
C 5.62379285334104 -2.85280038517204 7.95432277086059
H 6.58693221312701 -2.96410635386561 8.45398701410867
C 4.49847406285569 -3.50332264519996 8.47380032191540
C 3.16712259724297 -2.60587860579358 6.63311006024043
C 3.28008958256157 -3.37948772980824 7.79742072001825
H 2.39915808484586 -3.90158133882860 8.18098796790021
C 4.30103321253665 -1.94207795410198 6.15788257804178
H 4.22569475325912 -1.31640153425246 5.26889403970182
C 4.61513619598013 -4.33263474112544 9.72729735042254
H 5.30880233605808 -5.17515068741550 9.58368757861870
H 3.64218024022177 -4.74344837518733 10.02814205350591
H 5.00648053651610 -3.73156405972677 10.56178412609357
C 1.84277088403786 -2.48073856778728 5.92577911254833
H 1.20064871735916 -1.72734406732221 6.40910863744535
H 1.29409671978717 -3.43306867212476 5.93911016460856
H 1.97567903028864 -2.17598129705000 4.87936905588762
C 7.51168439786382 -0.44137882432750 8.48960519221584
C 8.43929662506758 -0.90462408915905 9.43233123085546
H 9.30045776582787 -1.47913706150773 9.08436216229173
C 8.28211757828802 -0.64516718968469 10.79815115986174
C 7.16127204896080 0.08432238430292 11.21485382424623
H 7.02760541093206 0.29734774709438 12.27902625844348
C 6.20855295426820 0.54919622004343 10.29828028321573
C 6.39761893833536 0.28123920376800 8.93924896392777
H 5.66935786108046 0.63860304336160 8.20743657629339
C 9.31916289224680 -1.10773094585653 11.78922305210081
H 9.74114225430817 -2.08093740915914 11.50212530456591
H 8.89849865678656 -1.19809526707322 12.79994624308843
H 10.15694476738610 -0.39366553258730 11.84266298818230
C 4.99067249940785 1.30286486824381 10.76541715965974
H 4.70476238565184 2.07679486576895 10.03926954155421
H 5.16257900034991 1.78898744707824 11.73547179688265
H 4.12734764412255 0.62812504239446 10.87982679610719
C 0.84459244692070 3.38214964650835 5.43275964855781
C 1.03252247389397 4.14515472498563 6.63134597208348
C 1.64158047397183 3.28918071625446 7.60544052009960
C 1.83631199843447 2.00256871771308 7.00766815096392
C 1.34082398414841 2.05775951739112 5.66666526493874
C 0.20875373243277 3.88309275564407 4.17415906554049
H 0.45190154236823 4.93852798620095 3.98867071695419
H -0.88914438845530 3.80142195467008 4.22138896572156
H 0.54152707158633 3.30599137086726 3.30100211137265
C 0.64579935434729 5.58016433661243 6.81551461795364
H 0.86034686975564 6.17482029587459 5.91533117683385
H 1.19085466380961 6.03978915281347 7.65091113188461
H -0.43040129659482 5.68387388706833 7.02810677206424
C 1.98127634923048 3.63740159446549 9.02120743552280
H 3.00244662511627 3.32539458147088 9.28555056407759
H 1.29827500107237 3.13518009409623 9.72423976523466
H 1.90615921862631 4.71631994489782 9.20136672059349
C 2.46186847693757 0.81569555295686 7.66915665901018
H 3.08202256145783 0.23265410194464 6.97449454732552
H 1.70020792417863 0.12986138266954 8.07353870323922
H 3.10525902225435 1.11559482856309 8.50565015138857
C 1.38995209527240 0.93622914902809 4.67804144131103
H 1.31303684010326 1.30444458932162 3.64723851117046
H 0.56909498835858 0.22019495961512 4.83755646713759
H 2.33326857042853 0.37634286016941 4.75912451757215
C 5.52144651172658 6.21062091012024 7.83314654207133
C 5.72906004789975 7.02827192139840 6.67131126023117
C 6.93016713331089 6.57631089182381 6.02696386353139
C 7.44634123535293 5.47436258754100 6.77730632303469
C 6.58283163514585 5.25028920899002 7.89519395869039
C 4.40322263166248 6.37926550454410 8.81309399396698
H 3.44651054741955 6.56943358372145 8.30464202306693
H 4.59048545795368 7.23030686009020 9.48715945014559
H 4.27500960774373 5.48520876070623 9.43495875900365
C 4.86384070017624 8.17610677403062 6.25086482630465
H 4.95839660291049 8.38026359704336 5.17553176677459
H 5.13309382348823 9.10089785389177 6.78645993855148
H 3.80245890744376 7.97534393294649 6.45667722077731
C 7.57793808122641 7.18982606519195 4.82565098252681
H 7.97134402509905 6.42672633023069 4.13979680214375
H 8.42387419715973 7.82999015112923 5.12255975264556
H 6.87430777803928 7.81611141975698 4.26420062371660
C 8.64849178297696 4.65169968851762 6.43597309974732
H 8.41642836885602 3.57646368167769 6.44662713565594
H 9.46631087356890 4.80807788785025 7.15586607768408
H 9.03320353992608 4.89506530563204 5.43760471213861
C 6.75397103379683 4.15166846198300 8.89724640109990
H 6.72968669302773 3.15623599522592 8.42285073045901
H 5.96262384272828 4.17060577168857 9.65675027122274
H 7.71928954702368 4.23133575954025 9.41875239785251
C 5.07132086593407 5.20834345819976 1.85268376170464
C 3.76669713223740 4.63370560885398 1.65586888496511
C 3.92974819393126 3.21630312530762 1.50069446481193
C 5.32401618420942 2.91991825735231 1.61267028577379
C 6.02901028511011 4.14492428622735 1.82697962272903
C 5.33764302202017 6.67524092582012 1.99311118824107
H 6.40265139093264 6.87254847713053 2.15973101086089
H 4.78230321505482 7.11718573489105 2.83555077146422
H 5.03575974512798 7.21749561954342 1.08347643534589
C 2.49658979212362 5.41862146721537 1.54176477065386
H 2.42737641596669 5.91689421935610 0.56114309467770
H 2.43160321573716 6.20172814762830 2.31172482845303
H 1.61377433275135 4.77619079261005 1.64664539607226
C 2.84506552284044 2.21654584021890 1.24293742041878
H 2.77891379118217 1.96921934440421 0.17148764672996
H 1.86306461473149 2.60183484438622 1.54827704583866
H 3.01982906247995 1.27784960619901 1.78702322546060
C 5.96189345669980 1.56885599394092 1.51454989845163
H 5.22097810239167 0.78524629846031 1.32085232214712
H 6.49590968679835 1.29166573606369 2.43716450045130
H 6.69860597915808 1.53946444857583 0.69692991229404
C 7.50690963607915 4.23478127798866 2.05151089967383

H 8.06076973861732 3.73742561581525 1.24166108184733
H 7.80359560855048 3.74243218917680 2.99312822483168
H 7.84793583495481 5.27669418225072 2.09814857786937
163
Entry 14 - Compound 8 Pt-complex // -7795.791713783530
Al 8.35115263849579 5.31308271574321 4.50478234325633
C 2.92402559919208 5.03640470635971 4.01832012911195
B 2.55273014294910 3.51544811227079 4.05182686981079
Pt 6.90965691002686 3.67243152700775 5.33898486410551
Ga 4.53403425072457 3.77409890313120 4.94865329860437
Al 7.04699088639388 2.28599085706223 3.39742804823540
C 7.75997791271042 2.42577486000503 1.24910467786723
C 8.76420589458758 1.76050745231809 2.02841830771712
C 8.20289420126007 0.54374740981860 2.52584688751040
C 6.84545313529448 0.46626142325192 2.07746849969123
C 6.57784132092393 1.61632284647913 1.26295938103224
C 5.30830107426052 1.83601613171611 0.50111643603850
H 5.34170891433207 2.76686792590988 -0.07452698414213
H 4.42650402733580 1.88234206547646 1.15465484467392
H 5.14303530144705 1.00708142037266 -0.20428859103971
C 5.88551399680426 -0.65116133449763 2.34067891882665
H 4.85187432793357 -0.28504468046437 2.33710840141439
H 6.07167971417019 -1.12684431054201 3.31375012909048
H 5.96244737852810 -1.43600148450364 1.57046481923636
C 8.90144573507728 -0.49560957091961 3.34012074277425
H 9.22786952785251 -1.33914469604706 2.71076751843046
H 8.24233213783181 -0.90570570170962 4.11817547233155
H 9.78984803858924 -0.08750540325039 3.83528821415151
C 10.13751450550492 2.29455909709006 2.29310188645088
H 10.69348822701769 2.45451050522331 1.35621505183748
H 10.72307005913071 1.60657829062931 2.91609629193713
H 10.09387735292673 3.25979538404862 2.82245219142763
C 7.94945450620809 3.74503319071383 0.56612360480140
H 7.06454879650634 4.02423481639856 -0.01734223837567
H 8.81625241499387 3.72042339867459 -0.11202684612929
H 8.12424649206378 4.55397564488931 1.29598615318534
C 4.54821542223840 5.02407487941624 1.40519419527748
H 3.89492445182372 4.30099052711116 0.90435944244959
H 5.33873541883859 4.46233808863763 1.92667811268553
H 5.02681860846894 5.65536146185883 0.64040540509647
C 2.31158611403464 7.07749649765754 1.66923479361602
H 2.77149262638304 7.53317366691540 0.77833950439423
H 1.87673878338230 7.88757593562558 2.27029016661494
H 1.48789514903452 6.43480850033103 1.32641084692142
C 4.92236496233070 7.31274752983474 3.26963688771043
H 5.40560320970303 7.79164288964590 2.40487843467865
H 5.69058431341214 6.75046629703176 3.82526794630784
H 4.52632165255241 8.09608618685901 3.92677416167486
C 2.98623950413516 6.91656684138642 5.79934395004274
C 2.18397070115145 7.87336902045866 5.16152327813512
H 1.46186984524088 7.53590140559071 4.41657371748514
C 2.29331341456457 9.23860936800975 5.45609355204712
C 3.20992148678120 9.64317764213113 6.43310383894040
H 3.30751247763632 10.70665729206768 6.66928354294093
C 3.99308210909507 8.70667719308831 7.12706252722356
C 3.86446800495220 7.35715399022186 6.80093558526344
H 4.46514255796470 6.62688903970228 7.34816102678403
C 1.45905532439468 10.24652252663600 4.70684196630650
H 1.41514122457375 11.20715797289184 5.23774881115456
H 0.43083436614817 9.88543533814162 4.56190591400587
H 1.87819724433477 10.43865436282255 3.70595247474607
C 4.94358421408517 9.13847163904279 8.21436781535776
H 5.59064417764420 9.96386853553997 7.88143182662717
H 5.58189790019904 8.30142333671083 8.52995278323755
H 4.39534376338150 9.49239784209317 9.10147765676012
C 8.82414975679384 7.44165797953771 5.12405757325175
C 9.02108338424082 7.30444911516807 3.71039535322795
C 10.07315745615628 6.34562179434946 3.49704949720134
C 10.51590345309386 5.88845705754416 4.78175207188847
C 9.74847504612780 6.56740426837068 5.78109265571789
C 7.81049603420375 8.32699679066650 5.78409432407543
H 8.18683556569050 9.35758768076805 5.88173222606309
H 7.56013864560544 7.96823986354759 6.79204295195291
H 6.87119536143824 8.36558703967189 5.21621835218880
C 8.35953069534034 8.10979688237217 2.63545150804922
H 9.03362426250340 8.90623575700797 2.28067670796865
H 7.44213457651705 8.58419958415041 3.00103887132046
H 8.09035914757213 7.49529552023339 1.76470087242242
C 10.68610725362537 6.03102607572476 2.16773673629402
H 9.93238556903638 5.97494487898385 1.37123762154003
H 11.23027095333260 5.07881658619809 2.17928165028653
H 11.40360212989727 6.81764832259820 1.88287898713094
C 11.57761094517696 4.87016699687901 5.06332855339809
H 11.90359535855912 4.36214307807884 4.14703989160715
H 11.21480611141880 4.09983822408068 5.76059069464957
H 12.46489288683500 5.33558866834823 5.52048913482395
C 9.89060762085328 6.34122673413346 7.25393978989796
H 10.84438840371589 6.74569751570684 7.62835694741779
H 9.86957347533387 5.26630107722740 7.49186266874968
H 9.07858522885102 6.81745891083214 7.81842009367322
Ga 6.50425743464305 5.14103894576577 7.39608034594820
Si 3.61324336395087 6.09955913612102 2.63988173392676
C 2.87098191868452 5.50409207991288 5.39307867112675
C 2.67299654575219 4.42160424786522 6.29801160449501
C 2.52946966390865 3.15774051446363 5.58901560980546
Si 2.21693797127537 1.49680517363215 6.38921984019740
C 1.82516177279834 1.55006233832414 8.23890519250351
H 1.06684951382614 2.30921866096097 8.47622761872884
H 2.70401228539750 1.76366088897875 8.85977295874378
H 1.42738649977226 0.56655171264564 8.53641766692983
C 0.71667505855706 0.66969811093759 5.59493838760007
H 0.76604989867686 0.65315549530359 4.49946502676161
H -0.19056902417907 1.22492785840799 5.87865203544064
H 0.60270421784066 -0.36438090224208 5.95565466284275
C 3.76750893663048 0.40935106961177 6.22382013416953
H 3.64881103491625 -0.53819852267720 6.77277739144932
H 4.62693950538984 0.95478388030518 6.64314860175909
H 4.02674302923489 0.17021842363835 5.18325810630263
C 2.60612705907442 4.66617728737436 7.75468770217040
C 1.57841185299202 5.47739685999995 8.25303790781489
H 0.82066199382932 5.84583839521874 7.55888929409763
C 1.52767241996153 5.84770252354367 9.60111131897437
C 2.53499032400036 5.38729166973550 10.45702253527346
H 2.51732579273890 5.67969826103643 11.51037928645734
C 3.57104488587729 4.56545554869117 9.99543217854786
C 3.58735072935245 4.20415017030327 8.64319375769549
H 4.37867746970441 3.55541426295512 8.25443659925001
C 0.42720054929314 6.74620780133685 10.10371219511383
H 0.46985534021225 7.73039314581521 9.61241069607835
H 0.50234225974808 6.90441794867581 11.18786164422100
H -0.56475943741016 6.32109220018412 9.88889662691451
C 4.67372479990978 4.13396970014473 10.92398826866961
H 4.86963317335356 3.05463181739708 10.85162371770124
H 4.43087825937627 4.36580518562410 11.96955158818870
H 5.61078335145483 4.65807955777967 10.67205919517035
Al 7.96404478262317 2.10762730128385 6.70409927494652
C 9.63947829669534 1.66760399576825 8.16262376907830
C 9.38617618804064 0.49976972865112 7.36878139729430
C 8.03768387442947 0.07151003391572 7.63087845202753
C 7.47187291169770 0.97429276443503 8.59109161681792
C 8.46140218421874 1.95499753156976 8.91822501420642
C 10.94113439988646 2.40292386373983 8.23773148163947
H 11.48681719858811 2.36228007208874 7.28524085172052
H 11.59666464871614 1.96555005391792 9.00827466852595
H 10.79546465521608 3.46001548914007 8.49735529795668
C 10.41834507198049 -0.20732826276473 6.54602988806453
H 10.96008322612158 0.47938400537032 5.87903366869929
H 9.97405361779458 -0.99449276631833 5.92747085867771
H 11.16715908542178 -0.68331238041402 7.19894018765047
C 7.34748917313103 -1.12692309176278 7.05178398698356
H 7.89002421197114 -1.51903151750788 6.18247549504998
H 6.32396184393011 -0.89250474948530 6.72704557575550
H 7.28401418803513 -1.93929812389766 7.79258956612723
C 6.08695634063798 0.91078585550480 9.15903655462640
H 5.49838012619100 0.11333864074259 8.69169713499109
H 5.53812496010569 1.85138721570236 9.00784026614795
H 6.11550200366894 0.71774802883509 10.24237633103954
C 8.29558104312544 3.07454731878894 9.89682948064764
H 8.86909789345052 3.96432715646598 9.60301104727855
H 8.64116798711434 2.77529289538830 10.89986141981470
H 7.24526476613771 3.37624517054413 9.98746379041777
C 2.27891677384824 2.59950991605236 2.80605353776013
C 2.50693516526883 1.20707086570602 2.76656674083439
H 2.95492297794257 0.71754777600566 3.63152738868338
C 2.16004239643840 0.41574143329091 1.66796046528486
C 1.61145372252035 1.04046600003342 0.53712073135720
H 1.34477203563502 0.43763611498428 -0.33589472487290
C 1.38419343786180 2.41982373500289 0.51545836867367
C 1.70209317032801 3.16884447172727 1.65704735736146
H 1.47818336363322 4.23780874935833 1.66031017078430
C 2.35229284077477 -1.08005605548289 1.69133930396318
H 3.05934820716084 -1.41291906021885 0.91535623010026
H 1.40117906213103 -1.60176030772825 1.50493237271903
H 2.73292400611747 -1.41776440618973 2.66459311715667
C 0.79960320076861 3.09555718790219 -0.69933917757090
H 0.55251350721409 2.36809508587134 -1.48437234601515
H 1.50563436923526 3.82581160353325 -1.12464154447735
H -0.11787413783632 3.64774650037777 -0.44547139215106
12
Entry 15 - Model Compound H Ga(Borole)Ga // -4030.657330025324
Ga 2.79234558818085 15.42617914564093 18.38584742377781
Ga 3.14188133405881 11.57457299792377 20.41935755050820
B 2.96995024744129 14.06847321158426 20.47856008662677
C 4.16965982284687 13.69798159752733 19.57037487643897
C 3.67833694428106 13.03064929549929 18.39016109155404
C 2.25060472569051 12.93089320892364 18.44662513492113
C 1.76567270050984 13.53001278034081 19.66544764484964
H 2.97281450183713 14.62777732602442 21.53915941299772
H 5.23779726716986 13.86891494534944 19.71054746699636
H 4.28354874210834 12.65123936278540 17.56740102532958
H 1.64116400910408 12.46661893192028 17.67190035972617
H 0.69850036977135 13.55175190548025 19.89006871727333
23
Entry 16 - Model Compound H(W(CO)5) // -4665.010573813350
Ga -0.64727327441654 5.73268175201085 6.85773403779779
C 0.78076704408665 7.30686926181419 5.50455888007849
B -0.13632118812977 6.48507313295046 4.54554718463474
Ga -0.21302290095799 8.65062910920772 3.90583465336844
C -0.03910005205420 8.16489214648732 6.32394046613363
C -1.41350277696528 8.01207831868897 5.97070704445931
C -1.54664082431225 7.04810927170016 4.90631542089649
W -0.25615365667741 11.06435934626650 3.03518352945534

C -2.03809201737360 11.22673371220599 4.03852757611260
C 0.74417435626074 11.53674443639114 4.76270887060561
C -1.26112216715319 10.47890945336708 1.33453448211112
C 1.53998724572721 10.79111345170371 2.06356622403974
C -0.33080258678928 13.00263265255586 2.49306015961095
O -0.37736248689520 14.12212174577177 2.19364839046697
O -3.03827377279548 11.29554505892266 4.62131690123930
O 1.30072633089351 11.77894398720372 5.75082086882196
O -1.82817803418304 10.14603990561667 0.38214174782534
O 2.55055064664554 10.63409370223390 1.52178267886256
H 1.86509185539844 7.29553089269196 5.61188286194185
H 0.16559982645221 5.67167263593520 3.72289044736855
H -2.52209602266696 6.80771068708780 4.48428720896451
H -2.22488970017086 8.58798070150837 6.41205621533823
H 0.32513837407680 8.87148043767762 7.06748424986634
34
Entry 17 - Model Compound H(W(CO)5)2 // -5299.353053253908
Ga 0.02291903416238 5.33012051685332 5.84786649140167
C 1.07601709022973 7.31471547245443 5.21813432206942
B 0.44614416503348 6.54980671689946 3.99866589052827
Ga -0.07447063806510 8.73144170738988 3.76245694279498
C 0.02108697392685 7.80640399024399 6.06926004186297
C -1.24919962190735 7.44578171268070 5.53787546009912
C -1.08535432697864 6.70055273199419 4.31464607501310
W -0.59197505688710 11.19843335159780 3.31921843672409
W -0.39040248617574 3.79524230032951 7.85376461152421
C -2.47490794420949 10.78833911045049 4.02988113694707
C 0.05253887553366 11.52610839715254 5.24106119599426
C -1.22251000317486 10.79172749230217 1.39819294136309
C 1.31643535103763 11.53673504808636 2.61545226973683
C -1.06469403598856 13.14457509152397 3.11908392949625
C 0.41326492499115 5.29989618131759 8.99795433364186
C -2.23040983267516 4.69830607973557 7.97510913993746
C -1.18435799459878 2.32716352221480 6.64299490111740
C -0.78374973406913 2.71499631126855 9.50584026024517
C 1.47400570811736 2.93325943665216 7.66762910769705
O -1.34168618601095 14.26440338124277 3.01546637975935
O -3.52879137214552 10.54139839263065 4.44139004620328
O 0.41216407058794 11.68948368144864 6.32980256296717
O -1.57784633251414 10.56476736517349 0.32183734765854
O 2.38674630959990 11.72816247383530 2.22267895463056
O -1.63181177129509 1.50431507867548 5.96534278229603
O 2.51935899179624 2.45048744018803 7.56536393649854
O -1.01512020989470 2.10341349061053 10.46182688657304
O -3.26015842438463 5.22539480389015 8.03199217397347
O 0.86034692978615 6.16195597733999 9.62876079310723
H 0.98381147657596 6.00428172047166 3.08361535611315
H -1.94465337500241 6.33356394752163 3.75555550950862
H -2.20116530854446 7.70023817331330 5.99839062722877
H 0.16366409839254 8.37329123112357 6.98651701988944
H 2.12399937875087 7.49021877338691 5.45605745129807
45
Entry 18 - Model Compound Borole-Ga-GaAl3Cp3 WITH D3BJ DISPERSION CORRECTION // -5338.963983980456
Al 3.75882006889339 3.10868902371359 5.52931633218240
C 5.74201419074197 -0.74100898800259 5.65608792770437
C 6.71410769206842 -1.57172883424693 5.02019066981258
B 7.97276619418060 -1.64655102487206 5.93350937904187
Ga 7.74216130360890 0.43993368565382 5.10799651109174
Ga 6.18683975004946 2.46365359148935 4.92528101175254
Al 4.96656942413585 4.11437436567696 3.33128129496375
Al 5.77785953465813 4.88038632156367 5.77107519574203
C 6.26892015940670 -0.20200846536856 6.87320093064662
C 7.60571006996311 -0.65998948636510 7.08015681923006
C 1.45934286227328 2.91508276359540 5.37156871234439
C 1.76225636754618 3.58230058379372 6.59351280821489
C 2.48122206829215 2.67409161002024 7.42234697126406
C 2.62186988787273 1.44771381865767 6.71451289870246
C 1.99091152740705 1.59622686975247 5.44807895721351
C 5.57510803188187 6.17984938620731 7.69158323806469
C 5.53607153280306 7.04929375846465 6.56412589594196
C 6.79425667417262 6.96643031094636 5.90254933188813
C 7.60985005086751 6.04754978856309 6.62200341848721
C 6.85698080227849 5.56270409140858 7.72744244898751
C 4.75347677037492 5.82327264512127 1.76159699168570
C 3.55786334854368 5.05107750365818 1.73161602851961
C 3.89407108394956 3.73701193371187 1.29799055655145
C 5.29652224112948 3.69738297921049 1.06052809555332
C 5.82787968693648 4.98630084728277 1.34733171840513
H 2.56793954719789 5.39703275026382 2.01479575317483
H 4.83490668941543 6.86078823981072 2.07262464558325
H 6.87435073731146 5.27167211368772 1.28869984226998
H 5.86676912424410 2.82701369492654 0.74914647854046
H 3.20601848157620 2.90290775109358 1.19403252384670
H 0.93547004193419 3.34485688898738 4.52265228561985
H 1.51086415868538 4.61019299400839 6.83860126075381
H 2.87762229127971 2.88826080695602 8.41076078405196
H 3.14825677038717 0.56439135579725 7.06455203631561
H 1.94957111598932 0.84479071203766 4.66476208401091
H 4.68671305293807 7.64832741013339 6.24861489522604
H 4.76064023469874 6.00125969886994 8.38775839494173
H 7.18952105935552 4.82198197180072 8.44891291800595
H 8.61792478365687 5.74372922324781 6.35505840667706
H 7.07398376554789 7.49263466169054 4.99436556438622
H 8.18918280244702 -0.33417063662245 7.94178341874199
H 5.72723715842789 0.49372373594347 7.51580047578595
H 4.75201619848941 -0.50445044237663 5.26381230914618
H 6.50987501269047 -2.05161587351269 4.06260556664170
H 8.98134143169041 -2.27621170238019 5.77560381448653
45
Entry 19 - Model Compound Borole-Ga-GaAl3Cp3 OPT WITHOUT D3BJ DISPERSION CORRECTION // -5338.856774243411
Al 3.35302739158302 3.22944557582623 5.46194075485859
C 6.36722340219379 -1.18601623409563 6.50474834086246
C 6.99488288243594 -1.84792360773370 5.39684305108775
B 8.52134146060742 -1.54246499696057 5.43635253180615
Ga 7.34961741845814 0.31815286183059 5.01672957044143
Ga 5.78662645405675 2.35126214245407 4.98460299842316
Al 4.81548648529995 4.10452537589584 3.30903703969267
Al 5.54974876074479 4.81001315796292 5.85656490114283
C 7.32802460209069 -0.41764811995894 7.23092665176645
C 8.62400197126550 -0.54559148125501 6.62801568453038
C 1.03331835728847 3.22876659497521 5.14412090368648
C 1.29993252292988 3.89387388180966 6.37446369061388
C 1.88468736254056 2.95000896622028 7.26618103037340
C 1.97651336877070 1.70160321684215 6.58794929944345
C 1.45105233420182 1.87349802178312 5.27678825703790
C 5.46176206148404 6.12861796092565 7.78447500606066
C 5.51136054841434 7.00025493323090 6.65890900815063
C 6.76317999682122 6.80442086786794 6.00763195927862
C 7.48635836008335 5.81497064630010 6.73195600058211
C 6.68270268646160 5.39730958823422 7.82899964237142
C 4.91807139776115 5.81795029912914 1.73009372034495
C 3.66086515502243 5.16248401614009 1.58644657118209
C 3.90943523420569 3.82392575690160 1.16727329794186
C 5.31752308939976 3.65327931680967 1.05118545680084
C 5.94068354954104 4.88408963468294 1.39840971431816
H 2.68608130621698 5.60366736337961 1.77565212902257
H 5.06841403706593 6.84618198664765 2.04758452050156
H 7.01079639775270 5.07073808783897 1.42818898928561
H 5.82860872859498 2.73537815405593 0.77393114717540
H 3.15706858182275 3.06128939907574 0.98507317743175
H 0.59903807442072 3.67951405965381 4.25590992028519
H 1.10889898971798 4.94205439292708 6.58783600169554
H 2.21640481783768 3.14966131592307 8.28154266885397
H 2.39741630642497 0.78507423241411 6.99179788913519
H 1.39797668164674 1.10948004919734 4.50589709408703
H 4.72606838399030 7.68170948684571 6.34324313969371
H 4.63237527694888 6.03037578196668 8.47958949257002
H 6.94475704351521 4.63497365153200 8.55758229453216
H 8.46830488901254 5.42621700041142 6.47639023426405
H 7.10179965726401 7.31221404541200 5.10860209601970
H 9.48702722511937 -0.00816917957659 7.02325374457391
H 7.08345868364248 0.20362459704903 8.09342638377691
H 5.30497385119953 -1.22044362855632 6.74957361364375
H 6.41771941717543 -2.46182159007850 4.70416364771714
H 9.38904057896820 -1.94137312793756 4.70994433493601
163
Entry 20 - Compound 8 r2SCAN-3c // -7792.944282763960
Al 8.53111316732882 5.32105102227553 4.78546878651211
C 3.01985399386925 5.07528124884617 4.02177203432540
B 2.62525008639098 3.56394966154242 4.08026616478950
Pt 7.03975755124380 3.58624611302234 5.22648059949437
Ga 4.64335357131054 3.83988216535573 4.96395720794307
Al 7.31541316159693 2.35608708836827 3.19360710425669
C 7.83263548708079 2.35452211636215 0.97603260554710
C 8.88987745052577 1.71341220271867 1.69453611906602
C 8.35453066520626 0.55022403573797 2.31934705780759
C 6.96753699962618 0.47318780438814 1.99980958931835
C 6.64603035116114 1.57634741738812 1.15100154493688
C 5.32008103312835 1.78085979680267 0.48783677186584
H 5.32550859115843 2.66413782163942 -0.15263001068787
H 4.49972947846675 1.89575822122762 1.20351856347244
H 5.08196305784238 0.91359864836917 -0.14023713506964
C 6.01853144749324 -0.60110588525448 2.42458654679255
H 4.98952510374065 -0.23448700110343 2.40945641257316
H 6.23384527426538 -0.95486820169539 3.43883898571200
H 6.07495300500020 -1.46723723495402 1.75224372191285
C 9.12398646678453 -0.44795654802053 3.11911976266604
H 9.40951569025507 -1.30793318841489 2.49934707812661
H 8.53040860241582 -0.82993032392960 3.95558745321953
H 10.04021471459008 -0.01232617307808 3.52540483800376
C 10.32073950826770 2.14965147240814 1.75757791214765
H 10.46234677334971 3.10190115040518 1.24175898032601
H 10.97476410085102 1.41291236015551 1.27533489779459
H 10.66791549861776 2.27450568004908 2.79119588371114
C 7.96555645743423 3.59589404641466 0.14931317282676
H 8.40799570931871 3.37278518909305 -0.82965943776860
H 8.60438041258556 4.33746261972946 0.64050653084768
H 6.99421407323960 4.06674097508213 -0.01878780218177
C 4.40876673186076 5.04976460688292 1.26786274401004
H 3.72032101031957 4.33477582571565 0.81041351442626
H 5.23080616312048 4.48339903431151 1.72435097314336
H 4.82838915843468 5.68519751468576 0.47727148969438
C 2.19342221850388 7.10141745637645 1.72264440365402
H 2.47988318544679 7.33144959929674 0.68914585701822
H 2.00217314072887 8.05274000315843 2.23049426388121
H 1.24946253179870 6.54698453235145 1.68971559437734
C 4.92547032197123 7.36428980814887 3.07262242709507
H 5.30941172398568 7.86096448461068 2.17257486448943
H 5.75542309872154 6.82297320548324 3.54578977293695
H 4.57298611009216 8.13012166550993 3.76946798232758

C	3.11018657134033	6.97723805773021	5.78402787644779
C	2.24895515410111	7.89537296440411	5.17573478732071
H	1.52398666246254	7.53034630215066	4.45238916224566
C	2.29250946287697	9.25430867371482	5.48888807276880
C	3.20351730486700	9.69215891789759	6.44925523101550
H	3.24451906023546	10.75002978580442	6.70664913796850
C	4.05152012530934	8.79214218472770	7.10260117114566
C	3.99375030016824	7.44469779969665	6.76226219245033
H	4.64422715275339	6.73934396836945	7.27746426499610
C	1.38882268871165	10.22774373137016	4.78004611051054
H	1.85797485675740	10.59543296431115	3.85856939979044
H	1.16807479213069	11.09803075089989	5.40548155280806
H	0.44234274899400	9.75684885793257	4.49754775405631
C	5.00343880022265	9.27250920199027	8.16576282382330
H	5.69290834699627	10.02858992773306	7.77101931267835
H	5.59417015674448	8.44344243396958	8.56680922135399
H	4.46030745377732	9.73276133363327	8.99920999426328
C	8.85439677882789	7.52764067413663	5.13301149596932
C	9.14205206794464	7.21885649559766	3.76761647935660
C	10.23761230381840	6.29554905975738	3.74505298146264
C	10.63170004599141	6.04389241758415	5.09536484387807
C	9.77801967080085	6.80200534836046	5.95145143967583
C	7.78202430435516	8.44764152839224	5.62947158333061
H	8.18510812468443	9.45119385509937	5.81424850735882
H	7.34508965399441	8.08592879765853	6.56573839434041
H	6.96192795857134	8.53831678020397	4.91290296086860
C	8.49626415761755	7.80184501118724	2.54937919363173
H	9.21120440299381	8.43283400027194	2.00705605348469
H	7.63489052386105	8.41729592877134	2.81225316571574
H	8.14490659704952	7.02799609348367	1.85724133624236
C	10.94325470136220	5.81545416373818	2.51792919696897
H	10.25647152832506	5.70346573146724	1.67390107836569
H	11.43776295724360	4.85502505146446	2.68448613486656
H	11.71461395809485	6.53655087851186	2.21715001612343
C	11.76738835125860	5.16858255417861	5.52285855274440
H	11.79919469280979	4.23510141801192	4.95028690772401
H	11.68874672308148	4.90736959520916	6.58097718978881
H	12.72835867040205	5.67788516705493	5.37770639304750
C	9.86526053710735	6.84937488912635	7.44528130521254
H	10.69487254502317	7.49312006601558	7.76403859462767
H	10.03344977372543	5.85526650145829	7.87340813342489
H	8.94565289447879	7.23963117159261	7.88796351225711
Ga	6.61837004128193	4.95992153417526	7.27392633822885
Si	3.58253685722923	6.12888812184120	2.57569602322424
C	3.01110511732762	5.55770623723827	5.39254343743825
C	2.80425293708881	4.49027627732637	6.31149168692818
C	2.62265464158604	3.22196367161072	5.61566957218236
Si	2.15755741349697	1.59089635942916	6.42218422935011
C	1.66971687405203	1.68489153604756	8.24721609828884
H	0.98968197119256	2.51751166896550	8.45552834704699
H	2.52532810738249	1.78895960679405	8.92020408613358
H	1.15092848773485	0.75142976228045	8.50101779560443
C	0.63625561382625	0.86044099961064	5.57829046966056
H	0.73774928593385	0.77046157360889	4.49428162212982
H	-0.22976026045966	1.50295622673592	5.77824890533557
H	0.41549173061111	-0.13175631765825	5.99102319354423
C	3.60900470012650	0.36643503587376	6.35787640781140
H	3.40976269482127	-0.49874014174533	7.00321598731363
H	4.51707729843527	0.86672999284904	6.71696995670798
H	3.82777008049126	-0.00790723007818	5.35264919694157
C	2.68165535067044	4.73584262499089	7.76536343970342
C	1.65738982273543	5.57325659382205	8.21737392240105
H	0.97097012506460	6.00039416557785	7.48963494367185
C	1.51462703207871	5.88046955757010	9.56812110713304
C	2.42496345144946	5.33938412248838	10.47745497121580
H	2.33250046080414	5.58181037278780	11.53483142053724
C	3.45544062951734	4.49861035905165	10.05750591668136
C	3.56770878969277	4.19971004873725	8.69984106036300
H	4.36044030277211	3.53733081288732	8.35197086405367
C	0.39109992278328	6.76794174160138	10.03154883115955
H	0.23425789275597	7.59845469739833	9.33585920435625
H	0.59321782750753	7.18284401643094	11.02332189618662
H	-0.55079826262371	6.20876559913248	10.09109008668116
C	4.45664604527615	3.96292715430901	11.04325881708387
H	4.62065174700421	2.88817807355965	10.90492199621184
H	4.12810696293371	4.12393579623788	12.07397354854605
H	5.42422193849179	4.46536666540289	10.91616833932562
Al	7.89618200120569	2.02625846586656	6.73056430153733
C	9.57853106833330	1.59384255017368	8.16735596691369
C	9.37111621741026	0.45232895955825	7.33245134093346
C	8.04621724111785	-0.03965997869359	7.58031197226245
C	7.45128796610768	0.79707946546026	8.57546748781761
C	8.39724922791589	1.80247384756062	8.93574679054609
C	10.84334835332389	2.38615491944587	8.25834657214622
H	11.37758895009459	2.40226633195744	7.30375558759264
H	11.51946363219981	1.95728442388758	9.00912637328886
H	10.64528473313844	3.42191004088080	8.55034465840877
C	10.42665939640571	-0.16479204595631	6.46956346376749
H	10.89722458573457	0.56895808622503	5.80587366726291
H	10.02444338364372	-0.96590247581589	5.84760799901950
H	11.21831044531436	-0.59669247195505	7.09426410370157
C	7.40260819220699	-1.24561870593481	6.96731067054418
H	7.93458349113781	-1.57099564634071	6.07017452234787
H	6.36337935147623	-1.04941035844685	6.68330630162706
H	7.40332090150736	-2.08557771937872	7.67282698764963
C	6.09293848563637	0.63069397347903	9.18331715613724
H	5.55217350138710	-0.20149324721269	8.72845818707123
H	5.47353040696647	1.52657647760483	9.06368414417656
H	6.17741748486707	0.43075916822370	10.25816604138001
C	8.19882897661808	2.84618501917762	9.98784158384174
H	8.69150035011375	3.78888022322085	9.73129043479328
H	8.60839292302338	2.50549638730956	10.94794301373410
H	7.13851763725938	3.06294514719863	10.13547645621349
C	2.27014573802857	2.63704607867340	2.85978114479392
C	2.54990348636120	1.26040354366331	2.80118034760756
H	3.10134017680332	0.80153413388944	3.61786470632204
C	2.13294570852549	0.45479184083334	1.74388958421360
C	1.44211199584560	1.04478970176975	0.68149922883085
H	1.11614613819861	0.42783943473253	-0.15549175172348
C	1.14996253807537	2.40748709086506	0.68712224450247
C	1.55747117521041	3.17594374813647	1.77932286302060
H	1.30071004622181	4.23344588520743	1.79675795916999
C	2.41818100470324	-1.02400753029532	1.72813043425070
H	3.16904137406996	-1.28080874037876	0.96985572547573
H	1.51338596592234	-1.59453225359694	1.49145983769273
H	2.78863949539811	-1.36872737595185	2.69839200079598
C	0.37066568542177	3.03646441192840	-0.43723573878042
H	0.45539668407112	2.45320918561488	-1.35914329988293
H	0.72083110141924	4.05397059431074	-0.63953905775192
H	-0.69489629246778	3.10352002407738	-0.18477607588897

163

Entry 21 - Isomer 8-I // -7792.951861817245

Ga	7.16459032095809	2.36470255448663	3.18380892248485
Al	8.51723068114701	5.34367811102154	4.83970837682800
Al	7.82529925313919	2.05767732190646	6.79454851319980
Al	4.60932431712070	3.99129499056071	5.11705531145001
Ga	6.69044166582223	4.95002283278173	7.31928606379086
Pt	6.97762031653464	3.62774090997426	5.27448258740947
C	3.07308995441558	5.06042486531999	4.03054599195040
B	2.66938089226485	3.55768025061380	4.10062679036992
C	7.82650646845455	2.36088004859623	0.89822494228420
C	8.87050584085350	1.71947353268983	1.63171874226443
C	8.33676539028133	0.53891295192823	2.22024646394159
C	6.96003700528033	0.44672073937855	1.86178124583797
C	6.64382487731669	1.56493079061633	1.02675187284991
C	5.34148589232839	1.75311535301347	0.31378869976705
H	5.32372934393479	2.68801774012213	-0.24917566927965
H	4.48393686542917	1.76275472645134	0.99478303406787
H	5.18197484991276	0.93291168779211	-0.39819192433592
C	6.02187784040610	-0.66138303698387	2.21430506737582
H	4.98939734535217	-0.30507848285974	2.21723238375217
H	6.23637335604131	-1.07751627587002	3.20483715045696
H	6.08661658762276	-1.48504871069328	1.49031345029957
C	9.10914516708357	-0.46615216314599	3.00892348382122
H	9.46754237954033	-1.28119665921649	2.36561235619788
H	8.49463741873899	-0.92018967239244	3.79304101857257
H	9.98355005853013	-0.01302082388649	3.48381416379743
C	10.29192825604609	2.17341236932630	1.75021825552704
H	10.45757787296340	3.09727251860181	1.19108569070816
H	10.98056422493987	1.41962694267517	1.34872898443168
H	10.58058609776610	2.35850792490533	2.79381886038820
C	7.96711227025811	3.61258325877480	0.08911052484658
H	8.42222104950446	3.40655363360122	-0.88845266173590
H	8.59826917088026	4.35035584322155	0.59641000175940
H	6.99703318263688	4.08378479334997	-0.08688952089614
C	4.42032039002020	5.00059424147744	1.26042250624796
H	3.71648851087851	4.30095755411150	0.80275663069867
H	5.22903985850290	4.41291764879739	1.71327181414255
H	4.85336047324316	5.62614754786425	0.46926127464783
C	2.21163359570624	7.05937479444757	1.72695756449917
H	2.47517626298512	7.26621647604819	0.68254038328025
H	2.03697845258149	8.02250254423983	2.21839991635629
H	1.26453440077394	6.50947557936261	1.72704604717693
C	4.95697110927526	7.34366526090031	3.03787549576211
H	5.32925504503326	7.83562093341494	2.13045875821212
H	5.79532233297525	6.81393673384428	3.50896632761627
H	4.60407273140075	8.11299492625555	3.73080551987588
C	3.16568321747202	6.98111819722102	5.78974980159824
C	2.30715879526874	7.89924981058564	5.17939599145017
H	1.58804461021697	7.53622613453057	4.44938068814708
C	2.34820600455667	9.25696673444923	5.49808645882306
C	3.25316563351220	9.69158255488676	6.46572862329235
H	3.29105611495917	10.74834297368149	6.72786156022812
C	4.09911269990840	8.79062964368850	7.11994238116183
C	4.04538077604550	7.44464306601726	6.77273203332007
H	4.69291096344997	6.73778366631675	7.28903778630031
C	1.44837724039464	10.23247153749200	4.78744632951054
H	1.92154161760046	10.60034919313340	3.86815817965170
H	1.22682384426324	11.10228276885227	5.41314692789720
H	0.50228630020646	9.76333716370191	4.50091878702391
C	5.04207576682424	9.26769982496724	8.19233549775299
H	5.72331996431248	10.03749338695946	7.81041894305447
H	5.64174565669625	8.44085307535580	8.58465956446488
H	4.49052023560371	9.70950538474215	9.03021367092427
C	8.84094533895332	7.55515293690246	5.12129828743938
C	9.13726006616434	7.20778372953461	3.76778786052139
C	10.23427338953543	6.28516311311339	3.77896767856702
C	10.62289014107058	6.07568289642731	5.13737351158326
C	9.76161866639421	6.85484275069032	5.96646862331139
C	7.76749028603991	8.48766619694947	5.59051383801408
H	8.17883652421665	9.48441774277417	5.79286461296453
H	7.29915192735712	8.12746330126221	6.51236709969461

H	6.96996142364043	8.59249114938323	4.85115783301362
C	8.50513537701149	7.75720398422113	2.52703308647534
H	9.23394063914940	8.35572960596079	1.96676175117425
H	7.65409055738654	8.39698687366093	2.76370965941856
H	8.14373552089729	6.96590604156056	1.86043697848022
C	10.94329671825169	5.77056305801072	2.56807965643693
H	10.25618301566193	5.62395543277438	1.72990287336544
H	11.44518849310743	4.81991341507166	2.76423270155707
H	11.70790435915684	6.48830452197531	2.24308546652980
C	11.75696472635175	5.21408138580203	5.59510426656460
H	11.80645959117210	4.27624283251930	5.03138043832607
H	11.66130143565994	4.96148674887980	6.65394083503629
H	12.71622535905679	5.73003353619227	5.46285036262098
C	9.85026582463111	6.96988170794915	7.45629126291122
H	10.63147595429690	7.68679808454356	7.73987838001445
H	10.09373779006077	6.01093036912190	7.92478279968145
H	8.90629573917268	7.31006181526468	7.88941649653113
Si	3.61635093949073	6.09707178352636	2.56527829408706
C	3.06112718011105	5.55989796305675	5.40059294766137
C	2.84503302280446	4.49608986107077	6.32813437734191
C	2.66910189302552	3.22211282270163	5.62969273551305
Si	2.19129776587081	1.59590030101398	6.43864256775665
C	1.69433322962258	1.70537449614348	8.26035712022101
H	1.01439024307576	2.54022579856360	8.45967658025758
H	2.54632404607704	1.81166535759733	8.93765877545400
H	1.17257943414546	0.77458924584090	8.51781927095619
C	0.66949878815749	0.87632936046714	5.58806859349408
H	0.78394295586376	0.76860163132600	4.50698873202537
H	-0.18985027556743	1.53356261387994	5.76746822605399
H	0.43056763809417	-0.10669795091311	6.01240749667625
C	3.63040237926427	0.35786672549975	6.38851563245286
H	3.42473922244654	-0.49434201331963	7.04887474799490
H	4.54557381695520	0.85432415245879	6.73458370785893
H	3.83922595654570	-0.03580300870063	5.38878443036882
C	2.71002370227423	4.75106547872004	7.78035770679230
C	1.67660033910695	5.58667462573060	8.21379876675177
H	0.99767336683699	6.00660560390455	7.47498770799217
C	1.51619868241790	5.90053117125695	9.56073230938851
C	2.41866707909806	5.36868180597044	10.48388186132492
H	2.31167297758052	5.61604980449284	11.53872508173293
C	3.45790127968615	4.53066557475128	10.08175633064719
C	3.58819665856926	4.22462862361156	8.72683709851892
H	4.38768000735609	3.56269161015299	8.39397070422023
C	0.38216863166745	6.78298778820369	10.00749279269836
H	0.20158946093068	7.58631393494514	9.28641665382697
H	0.58807354376089	7.23459636861778	10.98232870751643
H	-0.54809825562353	6.20867201208267	10.09814300329524
C	4.44606996603771	3.99887222824705	11.08266745991006
H	4.59720939902028	2.91999563612377	10.96130664976221
H	4.11208545778284	4.17876785237803	12.10838238129024
H	5.42080146027268	4.48672624287253	10.95524491111935
C	9.52320588556460	1.60601287129834	8.20542154848171
C	9.32326618357139	0.48916501158316	7.33865053388816
C	8.00431108728348	-0.02508539378828	7.57801222595158
C	7.40628135990574	0.77349876219539	8.60288136395125
C	8.34315950161712	1.78029022777316	8.98546819915726
C	10.77205009492458	2.42212113842830	8.30465250065783
H	11.32073329994526	2.43078851327152	7.35840018819208
H	11.44279677716232	2.01910093443105	9.07415689987789
H	10.54940388211194	3.45947790236134	8.57380666680956
C	10.36746396353192	-0.07916253111904	6.42978480364738
H	10.80355406895490	0.68402779447209	5.77568202290291
H	9.96107536396385	-0.86734137184581	5.79422035220108
H	11.18429997271599	-0.51494078147402	7.01802160821819
C	7.37846436946691	-1.22202511331118	6.93049341173775
H	7.85647472538743	-1.45589802357723	5.97620674235185
H	6.31349494884717	-1.06427883037748	6.73407008399676
H	7.47448443168008	-2.10532916908654	7.57398988634622
C	6.05915572236302	0.56953970691380	9.22465423949823
H	5.52226997855824	-0.25476468381486	8.75170361408751
H	5.42583256617496	1.46024330403271	9.14455310468489
H	6.16377796273577	0.33576080839491	10.29088688866499
C	8.14689425287224	2.78068064740842	10.07910191960048
H	8.63037328040598	3.73612851995233	9.85511410010336
H	8.56815688598426	2.40378715935914	11.02049435489860
H	7.08662190529220	2.98287177554837	10.24570332894162
C	2.32350256668581	2.61947272023739	2.88643891907630
C	2.61549593420592	1.24564899587963	2.82967630381201
H	3.19763911299877	0.79796030702227	3.63110833680547
C	2.16151024491285	0.42691169241729	1.79718507145195
C	1.43406547924431	1.00404945329022	0.75234679726521
H	1.08059404248814	0.37641674949206	-0.06530538301036
C	1.13922230405473	2.36578411035095	0.75121833967617
C	1.57523774960094	3.14544635636311	1.82413217744958
H	1.30982389736827	4.20085675710343	1.84223646764330
C	2.42670963474932	-1.05580180867926	1.79622674350574
H	3.10037681552226	-1.34541458624250	0.98006939403339
H	1.49487237720659	-1.61584749927951	1.65934315065255
H	2.87782891265318	-1.38153152356913	2.73822159394076
C	0.32463338079189	2.98108027034889	-0.35555507537971
H	0.38092758017791	2.38670099405954	-1.27243983432174
H	0.66810832176560	3.99592620942462	-0.58127682495743
H	-0.73252750077740	3.05130367592271	-0.07073318299059

163

Entry 22 - Isomer 8-II // -7792.947238941231

Al	7.12235348036007	2.52691577639581	3.14389081784586
Al	8.53223655595444	5.34341434462155	4.76113752304067
Ga	7.88940321299924	1.94372936517470	6.59216708387652
Al	4.59429834443503	3.97198977685982	5.04899220090544
Ga	6.68031024501082	4.97162206545865	7.36808876073097
Pt	6.95578135566032	3.68608691924363	5.23633189167675
C	3.05455495695189	5.04117894136026	3.97175662507491
B	2.64795834529164	3.53978634464470	4.04576373599181
C	7.76350039025314	2.45253708963636	0.96267860335801
C	8.80601721550387	1.88841473540878	1.76003609239652
C	8.28569293213052	0.73888742901644	2.41820262842692
C	6.92309994760637	0.58179538364011	2.02929173544633
C	6.59966620370284	1.63179646590454	1.11496415193918
C	5.30686487305928	1.74714659093289	0.36886634958844
H	5.29406037750300	2.62814645369067	-0.27494258192724
H	4.43960393150235	1.81018850439643	1.03369513059333
H	5.16620206441686	0.86530656110257	-0.26842434997356
C	6.00259140349839	-0.51681813778747	2.45355173869227
H	4.96112192870007	-0.21452543176369	2.32816103839466
H	6.14818407624623	-0.78668700583540	3.50556720796128
H	6.16556669095496	-1.42147529943988	1.85345970868574
C	9.04734177056502	-0.14848572198640	3.34496598523715
H	9.52357070279042	-0.97588226022597	2.80246487181932
H	8.38931939278157	-0.58455494931930	4.10280531056691
H	9.83251271759608	0.40663651750173	3.86568689489401
C	10.20582614600581	2.39916135777772	1.89994641156390
H	10.38223048978712	3.24484391666103	1.23156406004207
H	10.93228911290889	1.61832201726870	1.64644077831814
H	10.41874263749121	2.73110288888665	2.92566062081352
C	7.89533190993869	3.65134736233763	0.07493190621649
H	8.39165356292193	3.38927900346093	-0.86793866080420
H	8.48528166347045	4.44082862146610	0.55217333836767
H	6.91888205914244	4.07750339138511	-0.16583761576545
C	4.38949150578481	5.01911554037039	1.18902666615145
H	3.69043383401989	4.31599060328075	0.72952082040277
H	5.20924127504202	4.43742403536610	1.63026357628168
H	4.80985973998259	5.65852006788287	0.40217906091761
C	2.18084340894541	7.06280806908486	1.69259357076596
H	2.44840499473696	7.29883956449107	0.65540480257876
H	1.99240022689641	8.01092268837431	2.20729529758669
H	1.24028842761382	6.50190479442525	1.67355415846663
C	4.93613032394925	7.33074104800523	2.99644675242289
H	5.31823703008661	7.82221363354834	2.09297653404226
H	5.76813444830008	6.79355653877357	3.47107053532937
H	4.58642649436184	8.10201798371721	3.68896424403859
C	3.14968578404397	6.96218542589070	5.73372622394369
C	2.26918054548060	7.87418067527163	5.14552099214348
H	1.53841641911155	7.50606298072652	4.42976507816840
C	2.29963286846123	9.23001298392401	5.47242544456854
C	3.21547192687884	9.66858169983635	6.42803385783287
H	3.24218809851742	10.72308189809671	6.70045705617779
C	4.08574142249257	8.77437180188784	7.05904897262634
C	4.04475605815018	7.43069731430907	6.70020019543321
H	4.70984370948413	6.72867296263623	7.20209130221593
C	1.37345161368290	10.19929589282493	4.78764658336077
H	1.83338886549061	10.59919942765139	3.87504502533366
H	1.13424124346506	11.04940948935142	5.43370087618784
H	0.43742880308767	9.71421578046620	4.49490266462316
C	5.04122505444168	9.25683706296155	8.11760682235060
H	5.75817411781162	9.97875454799512	7.70787985806361
H	5.60248335860960	8.42295976440808	8.54949184071871
H	4.50376354138577	9.75953200998907	8.92952423014375
C	8.88991347138497	7.51860736112468	5.23337536139070
C	9.11435794613866	7.29360971589179	3.83953923749420
C	10.21000583829692	6.37964148151401	3.70884413955221
C	10.65764347803169	6.03830317060899	5.02202827680071
C	9.84512199637178	6.74318426317747	5.96175155902100
C	7.83387749714226	8.40736620253978	5.81419991146104
H	8.16405661361612	9.45368449405406	5.81878601349586
H	7.60047538644998	8.12681209450399	6.84468602196981
H	6.89839637862897	8.35309566898774	5.24824277339562
C	8.43310237858014	7.98090203479527	2.69790684315293
H	9.11732405266059	8.69906275851406	2.22836977672242
H	7.54924585562818	8.52633701426906	3.03257829147744
H	8.10938589330794	7.27708776495552	1.92345586952122
C	10.85816425582009	6.00103844058304	2.41628038320282
H	10.12211109981260	5.85398307348724	1.62005873819064
H	11.44676627897780	5.08555529239097	2.50827018116752
H	11.53694762918734	6.79905801665083	2.08827656921687
C	11.79897232498279	5.13263042277075	5.36192134404030
H	11.94264438929970	4.36002136360298	4.60061902811829
H	11.63534819887958	4.62842481326552	6.31874250007319
H	12.73639149358083	5.69738217434232	5.44205355890979
C	10.00725051469213	6.68226862153735	7.44848737246175
H	10.85878704087318	7.29587421172873	7.76884811550964
H	10.19038712352418	5.65834369273455	7.79165508907471
H	9.11617041593702	7.04184410825909	7.96853970210332
Si	3.58914732852927	6.09372697520025	2.51597224289533
C	3.04613651112058	5.54142790998589	5.34402685779050
C	2.82951052433485	4.48094097442164	6.27091405945809
C	2.65535661432764	3.20490520854635	5.57520066110724
Si	2.18896187839910	1.58207838381069	6.39889539096926
C	1.70562573081658	1.70291461246371	8.22314945480406
H	1.02376912637766	2.53616943031825	8.42241081962555
H	2.56309399393447	1.81693813116300	8.89219147028076
H	1.19001882704559	0.77151853287687	8.49070746061225
C	0.66187477451155	0.85221597619795	5.56527682835160

H 0.76180695127909 0.74802178362972 4.48239496639662
H -0.19960533569127 1.50288879967657 5.75813902172258
H 0.43532078181766 -0.13349885358394 5.99019019384445
C 3.63007199747226 0.34607315215564 6.35007525094706
H 3.42792694181615 -0.49837260370662 7.02143672349572
H 4.54790559865398 0.84411861679351 6.68669194759865
H 3.83278235614409 -0.06101086071353 5.35441371532927
C 2.70837431485754 4.74038593647062 7.72374940926871
C 1.67547589011217 5.57155377333429 8.16614436048708
H 0.98205702142829 5.98026204116956 7.43452232536580
C 1.53536683595196 5.89710748399599 9.51268581288005
C 2.45848714891058 5.38265077771951 10.42521948645086
H 2.36893567568201 5.64180154121063 11.47883152489893
C 3.49729800428638 4.54820329559912 10.01457764333160
C 3.60543115857527 4.22875786458320 8.66046540498814
H 4.40046224545632 3.56539218274370 8.31987120390043
C 0.40247067070257 6.77557703638294 9.97002446793100
H 0.20573929434214 7.57235521416637 9.24587184159296
H 0.62016015293625 7.23584661647304 10.93821561552521
H -0.52258132139264 6.19600884944014 10.07900656516092
C 4.50976008918084 4.03801647938508 11.00169483315102
H 4.64557949564257 2.95418386168637 10.91291776822468
H 4.21106054094486 4.25807604469510 12.03043030645838
H 5.48518151595737 4.50889830228718 10.82380476249888
C 9.58905108884740 1.45977247461262 8.17167824343186
C 9.33126929361069 0.30471505415658 7.36935688330972
C 7.98010019309274 -0.11768808232532 7.62936510499972
C 7.42734699991547 0.77404495348512 8.60014217444627
C 8.41924048854814 1.74311966221871 8.93043243340460
C 10.88897718162636 2.19425753773131 8.24894365845343
H 11.42181705601707 2.17468412520783 7.29300277687333
H 11.55143854640809 1.74839977700901 9.00270415722121
H 10.73889680270582 3.24187469608931 8.52837727032458
C 10.36670733475121 -0.43486063869303 6.58355591578259
H 11.01773491046341 0.24113895148417 6.01968029058592
H 9.92279717182025 -1.13827649401408 5.87627820286859
H 11.00924101734070 -1.01309497556766 7.26070138322040
C 7.30477143045986 -1.33337362364987 7.07385876613948
H 7.78445149161917 -1.67453444586043 6.15237206117096
H 6.25073710127680 -1.14405895326195 6.84675482396861
H 7.34693038686476 -2.16450229277004 7.78957177095671
C 6.06543635491529 0.68743486304821 9.21518093006534
H 5.48972016507137 -0.14141418138373 8.79817676364941
H 5.48070265103267 1.60118875548581 9.05885118318264
H 6.14148763474757 0.53066175958320 10.29819871843068
C 8.27542728075426 2.81701133502820 9.96090131382919
H 8.79422471574035 3.73757532717386 9.67568556191219
H 8.69132222558705 2.48744898538607 10.92287484467036
H 7.22596183306829 3.07320390530905 10.12456176496892
C 2.30786120406866 2.59579944593928 2.83467362242401
C 2.58982361152522 1.21932636630216 2.79933033055245
H 3.14525724850732 0.77652381177159 3.62182883244990
C 2.16296068935505 0.39319244730088 1.76132002480331
C 1.46508793485462 0.96361529547350 0.69308397496184
H 1.13124229589614 0.33073026674872 -0.12873360591211
C 1.17414055220524 2.32660291901279 0.67396421378780
C 1.58823680459050 3.11500106035576 1.74918037976252
H 1.32805534655921 4.17191385711021 1.75028380580438
C 2.43836695808037 -1.08759803474571 1.77591481618581
H 3.16430978269905 -1.37054648549945 1.00293984977809
H 1.52262528136837 -1.65642041176723 1.58085294769038
H 2.83394933567858 -1.40991942534575 2.74384042910561
C 0.38677189158091 2.93352050427669 -0.45684930806838
H 0.48040157205652 2.34249127137021 -1.37293163750356
H 0.72227019653484 3.95387847754499 -0.66900607639200
H -0.67983579505983 2.98730615837289 -0.20564074664183
163
Entry 23 - Isomer 8-III // -7792.919092182896
Ga 7.38846217530744 2.37750572806093 3.18206053675833
Al 8.44965517492281 5.40302675264673 4.95482612296168
Al 7.85922033323716 2.04740786376696 6.84273018106917
Ga 4.60244513255798 3.84201822565280 5.03690568986873
Al 6.57977926952689 4.82895534191846 7.34045247438782
Pt 7.01016809488306 3.59661193247205 5.31144839583913
C 3.01268985780970 5.09320067411640 4.06751059301699
B 2.58670969171380 3.59212725671487 4.13499628999624
C 7.91618940773775 2.30680088350207 0.85131479118086
C 9.00084671247732 1.67946858176027 1.53728338486553
C 8.49769513959180 0.51308341394455 2.18335127809917
C 7.10279232902019 0.41859918650742 1.90715777402984
C 6.74268824921979 1.51851409533746 1.06922499824496
C 5.40341643684711 1.70533083721974 0.42939971141103
H 5.35673778470469 2.63743509222140 -0.13681007790044
H 4.58377145486624 1.72217304418360 1.15539767662211
H 5.20230069562211 0.88196913910142 -0.26849512863434
C 6.17934372346403 -0.67046777124799 2.34919189096765
H 5.15835473685962 -0.29520609423549 2.45629126519883
H 6.48257302013651 -1.09438591200892 3.31238660189778
H 6.15277979862144 -1.49258159410596 1.62116995683453
C 9.32143609306025 -0.49676645185055 2.91215035198128
H 9.65959191508108 -1.28927817111904 2.23070989687305
H 8.75185351564202 -0.98156415335224 3.71075110378436
H 10.21333187716001 -0.04584694210764 3.35591372552469
C 10.44046292142839 2.08775090659387 1.51540691981051
H 10.57298252621739 3.07016030930427 1.05679169099455
H 11.03829527593856 1.37409073619050 0.93363690111363
H 10.87376423998720 2.12912599196629 2.52193591225190
C 7.99832342797741 3.52965167937914 -0.00837787153884
H 8.18559229556924 3.26538401879206 -1.05744000604819
H 8.80912653752798 4.19250143082397 0.30976839052896
H 7.06957364853413 4.10661207683282 0.02614158292887
C 4.36970513925354 4.92942252025709 1.31499435828684
H 3.65582156121423 4.20783985756593 0.90890249476782
H 5.18985600628193 4.36476752147098 1.77715417613182
H 4.78287608444738 5.51486548390996 0.48344019465475
C 2.21837010880800 7.05721751942961 1.70660630223873
H 2.53197858498347 7.29268508463993 0.68218712638278
H 2.00690976470172 8.00620489374263 2.21067457514193
H 1.28038142489534 6.49574719831546 1.64404661912124
C 4.95935598132956 7.30956720225206 3.02927977746018
H 5.34308318972265 7.76953068641406 2.10973540830266
H 5.78581784914430 6.77559749079951 3.51637360719099
H 4.62532426009860 8.10393468223565 3.70256457445063
C 3.18206227074587 7.00095518175015 5.81521070602825
C 2.38309696368626 7.95656442409989 5.18065344496834
H 1.65754958867755 7.62314220910834 4.44291007679531
C 2.49074872602074 9.31450664830881 5.48277630405437
C 3.40293396981836 9.71480906180021 6.45861720988384
H 3.49587853808302 10.77192593912123 6.70482264448327
C 4.18575880159068 8.77816340763122 7.14103465297894
C 4.06415798170399 7.43250510794930 6.81114156868805
H 4.66411497690809 6.69903412620649 7.34930906857213
C 1.65700098088329 10.32521448959898 4.74142025972569
H 2.14322152848238 10.61702198274540 3.80183038472259
H 1.50968819968399 11.23429005179308 5.33201889946065
H 0.67440016559589 9.91724211697835 4.48511995762253
C 5.12951209479087 9.21359573201930 8.23023832887125
H 5.75042999035317 10.05739357134659 7.90805551619793
H 5.78675945086333 8.39197232358629 8.53184275156646
H 4.57573677680671 9.53759088139815 9.11960336955251
C 8.70686262979972 7.63778668475322 4.95743237670462
C 9.09169905967841 7.11991766827260 3.68300756935038
C 10.19737362533671 6.22873844050566 3.88536615471660
C 10.50442177636241 6.21026115032288 5.28050881057288
C 9.57606667150518 7.06906010077569 5.94442771489599
C 7.60329106819789 8.60574551476088 5.25462545420658
H 8.00490875709161 9.51008287739311 5.72634286948245
H 6.85406294266840 8.18112084363844 5.93422540270540
H 7.07247494381356 8.90098666354223 4.34811098917866
C 8.51725206565483 7.45771513276236 2.34158766727003
H 9.29480158005249 7.87577343250998 1.69152020123789
H 7.71587301837173 8.19225698798260 2.42728821638472
H 8.09819262423370 6.57902689306304 1.83664423786745
C 10.95579684762146 5.53906106430647 2.79781414267823
H 10.30298070146894 5.29762263941989 1.95442180178530
H 11.41116156841379 4.60791918170971 3.14502859124202
H 11.76108507679620 6.18246595788649 2.42005614782881
C 11.62284648759322 5.44755525559643 5.91629808387327
H 11.75442743540005 4.46353071806100 5.45445776999453
H 11.44557052662813 5.29421874425631 6.98370693777700
H 12.57082502232671 5.99012606269486 5.81275054199374
C 9.55012498287426 7.39487222336423 7.40503869239359
H 10.22764353769358 8.23022754832651 7.62473459586464
H 9.85941188506920 6.54408974409949 8.01940181951248
H 8.54559128079219 7.68021535579292 7.72956316913287
Si 3.59031721786338 6.08461817794820 2.58435405088303
C 3.02306931269782 5.58422140869963 5.43386044192263
C 2.78682292445634 4.52382336281042 6.36128347831613
C 2.56510739882852 3.26046326393738 5.66919319772594
Si 1.99869712430256 1.65155041095571 6.45751991725414
C 1.36533548560185 1.80307900985993 8.23359983224455
H 0.72693531115818 2.68269680066591 8.36942987233317
H 2.16357677772728 1.86517317083790 8.97775494046306
H 0.76415787117885 0.91185277157047 8.45396381755639
C 0.53208040853509 0.94694377981786 5.50406324100238
H 0.74572941952578 0.76046657514522 4.44920768799442
H -0.30910913092304 1.64879537696721 5.55246094498705
H 0.20733587180038 0.00593083366963 5.96507055998404
C 3.41400613307879 0.38887228702646 6.49733703571150
H 3.15082326207238 -0.47509852025948 7.12049595967025
H 4.31293903173365 0.85738552165375 6.91597771075259
H 3.67709691996722 0.01885267729738 5.50076256456160
C 2.68709127080059 4.76141756606079 7.81681431662868
C 1.75849506119865 5.69462154023092 8.28786364594799
H 1.12454546823622 6.21150979164644 7.57110872921040
C 1.64082391406251 5.97926459301879 9.64587802734553
C 2.47710120190314 5.31488428334877 10.54473601724058
H 2.40178665883165 5.53521201264016 11.60830118758102
C 3.40870891002281 4.37429825281529 10.10654604753837
C 3.50034885248028 4.10308660891605 8.74150582382947
H 4.21161884145567 3.35872752969750 8.38342810605087
C 0.61709814533412 6.97118226264753 10.12829028469224
H 0.55131701474826 7.82862021525985 9.45095324347977
H 0.85954166479403 7.34018164858072 11.12913639607415
H -0.37912194552060 6.51431104137895 10.17538513020120
C 4.33233855522839 3.70020232876968 11.08248922361846
H 4.43480950362298 2.63124368560089 10.86395354048819
H 3.97260481758090 3.80193001085546 12.11037843578616
H 5.33353345297498 4.14748559210443 11.03104482799391
C 9.56575598948679 1.62352794432342 8.23167421271199
C 9.43990952210765 0.54862054895461 7.29874400366161
C 8.15436988313719 -0.05837699556573 7.49689265005254

C	7.50275076884327	0.64009588597020	8.56210852770996
C	8.37473413104830	1.67582352122697	9.01415269883291
C	10.75922911267106	2.51070537281031	8.38905508772665
H	11.32439537817944	2.58941035743753	7.45609722081709
H	11.43810007018804	2.12001819547277	9.15774964370316
H	10.46700305558577	3.52146441221532	8.69122227705656
C	10.51357375101485	0.11976634618958	6.34777097338980
H	10.82451371032179	0.93378378469998	5.68258147593085
H	10.18433760820314	-0.71068073042183	5.72167239700720
H	11.40020000736062	-0.21225737181541	6.90093923887075
C	7.59255227195843	-1.24105326489775	6.76961330785629
H	8.12175845856336	-1.42149949564454	5.83140297852152
H	6.53405245301159	-1.10016811305694	6.52766755398119
H	7.67824613380359	-2.14876707899567	7.37935215470141
C	6.16669108693331	0.31397630607143	9.15282841878164
H	5.63193197752831	-0.42349393759243	8.55110849162618
H	5.52649442242553	1.19913769110621	9.23279621251320
H	6.28531141233606	-0.09784279817704	10.16251245922587
C	8.11720717799493	2.59353948667280	10.16561403660105
H	8.51439562808274	3.59737142060876	9.98831105773649
H	8.58673708543872	2.20081469034389	11.07707385077040
H	7.04708961721606	2.69582083411900	10.35828694542935
C	2.21442393488237	2.66329732867065	2.92241373534985
C	2.58748131608145	1.31337226416717	2.81123976699019
H	3.22935820644927	0.88004105984720	3.57637165567869
C	2.14911006305259	0.50219497918444	1.76600616903176
C	1.33674054547282	1.06220891997570	0.77617591551706
H	0.99107460559248	0.43951861663607	-0.04840503552219
C	0.95150066802295	2.40018863416749	0.83568257652222
C	1.38939031151420	3.17474613540805	1.91142066007064
H	1.06692723622438	4.21271886348232	1.97391399269527
C	2.54261222125039	-0.94873938225190	1.68291536246320
H	3.32866321154839	-1.10770826182105	0.93352670268020
H	1.68886623953899	-1.57043579118383	1.39307398984757
H	2.91786481283856	-1.31589810044040	2.64305372424954
C	0.04627955373545	2.99251117509464	-0.21142782667039
H	0.08067061612914	2.41839152079847	-1.14217240859983
H	0.32533554310664	4.02745308865775	-0.43485087469338
H	-0.99529009451575	3.00356467411645	0.13293836014662

163

Entry 24 - Isomer 8-IV // -7792.922181933833

Ga	6.94625031164400	2.10493446799994	3.27464467984554
Ga	8.46256961511777	5.22804185726876	4.64748930278504
Al	7.74233173940317	2.01985397385944	6.82839406056828
Al	4.51621177332061	4.03020983343231	5.16106266796022
Al	6.67218918820442	4.91658608413282	7.28791971567367
Pt	6.85264726829073	3.55674296856378	5.31056327373999
C	3.01246292525339	5.09867003485840	4.03230146980861
B	2.61222906961835	3.60035541292599	4.14045426911579
C	7.84481687594971	2.62813169924768	1.13594345617793
C	8.86976350133534	1.93459787966315	1.85338872017577
C	8.42002227433981	0.60919999476061	2.09910210467177
C	7.10498019010014	0.48546322638350	1.55650051890354
C	6.75619348274777	1.72729835462464	0.93662929856844
C	5.51171842411516	1.93876289657191	0.13178632294876
H	5.46770080532942	2.94604183457305	-0.28459529176295
H	4.60054748003207	1.78880663144084	0.72307890050517
H	5.48097543489703	1.22803045466785	-0.70374050490963
C	6.30398286450989	-0.77254437392186	1.47082270660193
H	5.24925946675469	-0.54943190672893	1.29700282849791
H	6.37482516014654	-1.37156011272248	2.38496438966201
H	6.65322567106981	-1.40194797896854	0.64050114542300
C	9.19958329435293	-0.51671803563129	2.69632313914204
H	9.58941325034829	-1.17648443607175	1.90965928884238
H	8.58027752007358	-1.13713949712001	3.35352935434450
H	10.05358480831343	-0.15945193137003	3.27589016343549
C	10.17705762962039	2.53737409404409	2.25320282874005
H	10.78454801030164	2.79411600634718	1.37553368442910
H	10.76286138813329	1.85476453721282	2.87534024063271
H	10.02674202107786	3.46013481832872	2.82865157348256
C	7.95910795521025	4.04503619387223	0.66874790618805
H	7.08504255878689	4.34979452293645	0.08972529363528
H	8.84690380347638	4.17693915082500	0.03751330799323
H	8.04794068144653	4.74344814246982	1.51370488643620
C	4.33761732871741	4.94369863166657	1.27223551296429
H	3.62199379840098	4.23828787017038	0.84245501499113
H	5.13707635443481	4.35849139733203	1.74266546949105
H	4.77797365147979	5.53209601402232	0.45725542118949
C	2.13312746809484	7.00160622413454	1.67711515662243
H	2.42916355430932	7.26978295338822	0.65560721140411
H	1.86077205552065	7.92710102779256	2.19465637614350
H	1.23509330922133	6.37741578102546	1.60992333363427
C	4.90209198828181	7.34640425980682	2.96578508763862
H	5.28823186214342	7.79659623534861	2.04271607854737
H	5.73355547574654	6.82929269888768	3.46292972352690
H	4.55662983178682	8.14830309493533	3.62496566153897
C	3.12660439883688	7.06856230412888	5.73413183342150
C	2.24898776800686	7.97029983047900	5.12718391353441
H	1.50866032609464	7.58794299864861	4.42894552283024
C	2.29281388640185	9.33469202496658	5.41609515364949
C	3.22054680180752	9.79167891092979	6.35099680556482
H	3.25868710988835	10.85313033613433	6.59318513320216
C	4.09093246035869	8.90850132747387	6.99778126054689
C	4.03473121657906	7.55544888950792	6.67888193295142
H	4.70212500716787	6.86148173217406	7.18884606456773
C	1.36777539127926	10.29080375731551	4.71180269568955
H	1.79786470263342	10.61518380069829	3.75580339771304
H	1.18566525250955	11.18740979425049	5.31163589719007
H	0.40419133376743	9.82112440641144	4.49173674781846
C	5.06714015575303	9.41461448824620	8.02578958832499
H	5.81849551367155	10.07099487039552	7.57000036188498
H	5.58984818161948	8.58641677360790	8.51289838630438
H	4.55527185929680	9.99647624791849	8.80031522275776
C	8.88980246565603	7.47140353373343	5.21925538873105
C	9.11377942482098	7.29212942701466	3.82054509036811
C	10.21322909340628	6.38138910617217	3.66071638089824
C	10.65783635972977	5.99760926112428	4.96483380933290
C	9.84579266208868	6.67285448894436	5.92385682703026
C	7.85402602016175	8.36044051811142	5.83223816285159
H	8.23131680602530	9.38567790488460	5.94039056630735
H	7.56201676423462	8.00665183483564	6.82578516261397
H	6.94225515159415	8.40109865051994	5.22790395866015
C	8.45253434800749	8.03342807326899	2.70288814395281
H	9.12036273270697	8.81725195621189	2.32147640546962
H	7.52762459921139	8.51192522230969	3.02994605012083
H	8.20500935513027	7.37710374462513	1.86161533647137
C	10.89485243741644	6.08813365146042	2.36293133130414
H	10.18324978769972	5.89555040147358	1.55457197559372
H	11.56219717909367	5.22637258090472	2.43747556701347
H	11.50468088603992	6.95026164768546	2.06116623504349
C	11.80611599889015	5.08982172449828	5.27234341630059
H	11.91805674832516	4.30767876640135	4.51467117950254
H	11.67354045001426	4.59808520479863	6.24067784018460
H	12.75107408483842	5.64699313345010	5.31446325311459
C	10.02375698224841	6.59604754820281	7.40698988867357
H	10.86151029125016	7.22856438399996	7.72903172352216
H	10.23783410009781	5.57342770046181	7.73632968363157
H	9.12803391558376	6.92479985563431	7.94019528456613
Si	3.55375593283182	6.09204069746530	2.53865676491468
C	3.00102677445211	5.63697655463809	5.38795112295763
C	2.76432355610375	4.60325643436213	6.34572957800085
C	2.57176267990354	3.31538607408429	5.67832057058088
Si	1.91645153808659	1.75708232422474	6.49881172268391
C	1.24858553960962	2.02255872725532	8.24777618300674
H	0.61928822838814	2.91629082640275	8.31674560916217
H	2.03139908286701	2.11837659451553	9.00489852824245
H	0.63058738467782	1.15310125374845	8.50626650369073
C	0.43587036403712	1.11806120743603	5.51998732748604
H	0.67999661732871	0.89002482575930	4.47926233850955
H	-0.36232137813652	1.86961593813007	5.51891734951602
H	0.03994723214279	0.20977120949913	5.99114849822183
C	3.22692744007681	0.38163917060445	6.61953658419271
H	3.08454786750204	-0.19876334630030	7.53911446904542
H	4.23782843872213	0.80822522844598	6.63235794389515
H	3.16657741580627	-0.31425413858964	5.77582292537761
C	2.69498820537517	4.87651975965418	7.79776388081911
C	1.78005536480784	5.81886928611783	8.27232317301103
H	1.12408393197498	6.31846429360048	7.56311771400209
C	1.70651012748195	6.13803739911667	9.62636871652406
C	2.57112092971864	5.49716430774324	10.51437212366197
H	2.52880452329590	5.74426067668958	11.57372670511648
C	3.49205609362565	4.54742996071914	10.07145830792986
C	3.54081010253216	4.24294779492812	8.71159287901628
H	4.23942247723683	3.48801619529819	8.34847756237232
C	0.70434022457588	7.15078860113484	10.11083891446981
H	0.71526418125681	8.04431199436904	9.47763266236558
H	0.91035607810873	7.45611079020629	11.14055562925565
H	-0.31286543811675	6.74210898188332	10.07992792166544
C	4.44596802576201	3.89750323470839	11.03472982395015
H	4.52822375231240	2.82047828518419	10.84989931908763
H	4.12717731190593	4.03937639391469	12.07126137727651
H	5.44918097134261	4.33062855760973	10.92923782487059
C	9.44240005444722	1.43016417672849	8.16902271990102
C	9.20933508913527	0.39447409959589	7.21464391549682
C	7.88047690950017	-0.10741727720937	7.41902877703358
C	7.30717530628913	0.61481100074586	8.51463547604230
C	8.27031950160654	1.56681417111114	8.97079804603278
C	10.71170487787809	2.20562006091076	8.32172123898750
H	11.24027679551308	2.29835750712619	7.36824214179253
H	11.38887343306828	1.70918732520495	9.02829261706035
H	10.52147893292507	3.21353692009404	8.70294606879650
C	10.21819964636863	-0.10425142336674	6.22908767100194
H	10.63656325712032	0.70734877914911	5.62270712540435
H	9.77975139897014	-0.83645537359911	5.54944730802213
H	11.05170817269716	-0.59101097572929	6.74982985259828
C	7.22266161028949	-1.21105486222434	6.64862677448802
H	7.57393546143482	-1.23801790382690	5.61318700536893
H	6.13666849155232	-1.08549136023118	6.61816327347363
H	7.43767232538665	-2.18718070367529	7.10069820424581
C	5.95729591161513	0.39695987751594	9.12635466726761
H	5.41739807199049	-0.40590472450640	8.62192320824614
H	5.32751469775358	1.29245161666973	9.07732197844554
H	6.05905601569508	0.12214345467998	10.18267101716439
C	8.10156906635361	2.48617536488107	10.13807277413343
H	8.56487581431230	3.46185400161332	9.96265237284133
H	8.56014764100978	2.05262385939622	11.03630191925441
H	7.04502904070664	2.66012305275832	10.35344988254485
C	2.40933700930491	2.53684796072855	3.00133494094772
C	2.88591080351028	1.22053475032790	3.12194346488450
H	3.50871319830489	0.96363074638047	3.97674105484907
C	2.54768683208427	0.21892914285076	2.21373548099590
C	1.78291276856222	0.56027479959962	1.09503656333118

H	1.52306744593286	-0.20810326007171	0.36759438785591
C	1.32973862558135	1.86659052653887	0.90594714344777
C	1.63300266000524	2.82849250983244	1.87163718189861
H	1.23878778226830	3.83675386124969	1.74832737113516
C	2.91715687915814	-1.21660872224238	2.47941478961039
H	3.14136160510091	-1.76241027276923	1.55670921491342
H	2.08185414728983	-1.73508999749245	2.96766023228832
H	3.78185830156110	-1.29093368084594	3.14539043453576
C	0.48404681752002	2.22325978841755	-0.28779707898140
H	0.64800040343322	1.52800632347121	-1.11655091721547
H	0.70385204076811	3.23624497999830	-0.63984022745798
H	-0.58304655290831	2.19081898465024	-0.03501791673104

162

Entry 25 - Compound 7 // Input structure for EDA-NOCV analysis // from an X-ray structure with only H optimised

Si	6.216750740	-2.582725720	3.623263850
Al	3.069016740	3.523515970	5.602487360
C	6.718317500	-1.614620650	6.391892330
C	7.104049760	-1.673487340	4.983685340
B	8.518084960	-0.977256030	4.872456640
Ga	6.882194910	0.456251940	5.475535800
Ga	5.431659300	2.553431010	5.335608470
Si	10.217089820	0.638972720	6.826442010
Al	4.691049170	4.071034860	3.421532700
Al	5.372590300	5.049588300	5.930250060
C	7.675357110	-0.862577830	7.146546440
C	8.764886330	-0.391004650	6.297096860
C	9.510422650	-0.811690760	3.680341490
C	9.395536740	0.175592390	2.673179470
H	8.518680610	0.844259150	2.676580170
C	10.361421530	0.327611550	1.678031710
C	11.474009220	-0.467080620	1.685708430
H	12.246782690	-0.328020110	0.909773660
C	11.648636570	-1.459984420	2.652960220
C	10.665446100	-1.589790510	3.622963500
H	10.805354030	-2.365858820	4.396851590
C	10.192811720	1.409196530	0.628898180
H	11.117902280	1.556851020	0.037395890
H	9.923911930	2.381140050	1.091931860
H	9.378931110	1.156885870	-0.085302100
C	12.876620170	-2.333447840	2.622854430
H	12.867166560	-3.012019140	1.742725060
H	12.952209530	-2.965473700	3.529844180
H	13.804098850	-1.727894580	2.550416200
C	7.357397980	-2.742748880	2.161942490
H	6.877942760	-3.349453480	1.365509770
H	8.312705450	-3.230701250	2.439964300
H	7.622604390	-1.757346570	1.731147470
C	5.724072150	-4.307281420	4.156300610
H	5.286089130	-4.864248880	3.301544740
H	4.982192300	-4.299513440	4.978277050
H	6.607504280	-4.876137980	4.512478340
C	4.670624800	-1.715945000	2.967678490
H	4.882242600	-0.652318350	2.730483280
H	3.821604470	-1.738203930	3.678954800
H	4.331235010	-2.208396130	2.032206980
C	11.670600710	-0.485585840	7.137367780
H	11.929402320	-1.054826860	6.222357270
H	11.446372950	-1.218650800	7.939173290
H	12.569359000	0.088263140	7.446995100
C	10.668222860	1.855776720	5.475791280
H	11.059321580	1.343647640	4.575429190
H	11.439703750	2.569392720	5.833090940
H	9.781790000	2.440532750	5.157354300
C	9.942886020	1.644303290	8.374472930
H	10.786854470	2.351699620	8.516112250
H	9.863647260	1.015987000	9.282699220
H	9.009588150	2.241251490	8.321657120
C	5.566311440	-2.309009750	7.030384480
C	5.804892530	-3.122956550	8.164695770
H	6.829809910	-3.203002190	8.552650860
C	4.780005250	-3.821046650	8.788267160
C	3.222279010	-2.957242400	7.147659030
C	3.515798560	-3.736304010	8.279687900
H	2.695036400	-4.290399420	8.767234700
C	4.260580930	-2.250473090	6.561743740
H	4.047334470	-1.613540750	5.689199180
C	5.086587910	-4.642178640	10.031364660
H	5.879413210	-5.392673690	9.831695480
H	4.190763340	-5.180153470	10.398753450
H	5.456259460	-3.991227630	10.851175710
C	1.829945310	-2.899715170	6.572382070
H	1.054462080	-2.911525630	7.365089210
H	1.632956830	-3.773055900	5.913175350
H	1.681976780	-1.988887130	5.959152400
C	7.568242160	-0.608446120	8.606127390
C	8.437163860	-1.283784000	9.460256290
H	9.162540920	-1.992631540	9.030013100
C	8.386820680	-1.085372360	10.840241320
C	7.484205670	-0.179142460	11.345051430
H	7.446729630	-0.007086860	12.434640930
C	6.598575760	0.530113270	10.520211910
C	6.650600580	0.287284900	9.144503880
H	5.963337290	0.818052710	8.465597540
C	9.280045790	-1.883781090	11.753218950
H	8.926042760	-2.933944100	11.837448710
H	9.308453780	-1.460558920	12.776619330
H	10.320493210	-1.927762110	11.370926190
C	5.627542380	1.521798320	11.098298140
H	4.895688670	1.860737440	10.338511590
H	6.150071190	2.423133650	11.484676800
H	5.060388190	1.090874160	11.949377870
C	0.902566040	3.270941800	5.066130420
C	0.964410600	4.078524230	6.233078430
C	1.552053380	3.306686900	7.268125460
C	1.815915700	2.024725690	6.754960770
C	1.415531830	1.984028490	5.404920760
C	0.300914040	3.685137690	3.755372550
H	0.495338350	4.753149540	3.535414180
H	-0.801105790	3.542297650	3.753515550
H	0.710626660	3.093735200	2.914061660
C	0.452904810	5.490427470	6.335970390
H	0.612289050	6.052548870	5.393834460
H	0.959647990	6.052753000	7.145163660
H	-0.637059910	5.510649710	6.551737370
C	1.718289910	3.730259770	8.697460320
H	2.674740030	3.369087620	9.128401870
H	0.902574570	3.319891150	9.330239990
H	1.700301030	4.831042910	8.805195530
C	2.475466640	0.897727220	7.537151280
H	3.036005250	0.208510480	6.877315590
H	1.725969360	0.289970250	8.086349390
H	3.195352620	1.290391020	8.281619090
C	1.552979980	0.826121300	4.479145510
H	1.464319080	1.134619350	3.420048150
H	0.772147710	0.057508440	4.663908300
H	2.537011600	0.323539080	4.588221010
C	5.426627150	6.331133970	7.781564880
C	5.670867530	7.178783450	6.637791750
C	6.874891750	6.775774910	6.049624570
C	7.404466870	5.673162260	6.790147980
C	6.513390930	5.410526860	7.858673660
C	4.312510670	6.491769200	8.736582980
H	3.362094230	6.753148080	8.226955270
H	4.516818210	7.300810190	9.471814060
H	4.125299510	5.566335960	9.315432870
C	4.809521890	8.348097340	6.224573020
H	4.904393590	8.565811840	5.142415510
H	5.091503030	9.271276200	6.775115570
H	3.737411070	8.155665310	6.429823930
C	7.539181000	7.440759030	4.892776410
H	7.980830970	6.706987880	4.187743130
H	8.370707160	8.094521770	5.234273780
H	6.837357800	8.075305880	4.321087430
C	8.666417880	4.921693580	6.460203080
H	8.829548270	4.858214680	5.366063510
H	8.637187700	3.886032340	6.848420300
H	9.562196850	5.409869340	6.900481470
C	6.693449460	4.328688190	8.876365490
H	6.901300470	3.344411730	8.405246520
H	5.790244830	4.201166240	9.502298660
H	7.543878120	4.544527970	9.556458730
C	5.132354480	5.502539220	1.749579860
C	3.840111660	4.944984060	1.523049230
C	4.012750060	3.548251470	1.316112180
C	5.388126220	3.239654830	1.422770130
C	6.083735430	4.462270520	1.684792290
C	5.391069620	6.968773040	1.974317660
H	6.462556940	7.169109790	2.155749860
H	4.825136530	7.358972140	2.847055560
H	5.082812370	7.567712480	1.091463920
C	2.575771990	5.742676650	1.459134200
H	2.568683950	6.416631430	0.575581250
H	2.444785030	6.383208320	2.356994040
H	1.683540060	5.092930670	1.384764780
C	2.948812020	2.556912730	0.964323680
H	3.045163290	2.225709400	-0.091508330
H	1.934520820	2.985005080	1.079569030
H	2.999494480	1.646477660	1.596561620
C	5.996994870	1.880872140	1.311185930
H	5.298348960	1.154049090	0.856106890
H	6.293066320	1.466691390	2.301133130
H	6.915917530	1.894270030	0.690660850
C	7.571942040	4.554287640	1.855317790
H	8.099714190	4.285956310	0.915924170
H	7.938724190	3.861971560	2.643254120
H	7.893125240	5.575673340	2.135917660

Literature

[1] R. K. Harris, E. D. Becker, S. M. Cabral de Menezes, R. Goodfellow, P. Granger, *Pure Appl. Chem.* **2001**, *73*, 1795-1818.

[2] a) M. Schormann, K. S. Klimek, H. Hatop, S. P. Varkey, H. W. Roesky, C. Lehmann, C. Röpken, R. Herbst-Irmer, M. Noltemeyer, *J. Solid State Chem.* **2001**, *162*, 225-236; b) S. Schulz, H. W. Roesky, H. J. Koch, G. M. Sheldrick, D. Stalke, A. Kuhn, *Angew. Chem. Int. Ed. Engl.* **1993**, *32*, 1729-1731; c) C. Ganesamoorthy, S. Loerke, C. Gemel, P. Jerabek, M. Winter, G. Frenking, R. A. Fischer, *Chem. Commun.* **2013**, *49*, 2858-2860.

[3] a) P. Jutzi, B. Neumann, G. Reumann, H.-G. Stammler, *Organometallics* **1998**, *17*, 1305-1314; b) P. Jutzi, L. O. Schebaum, *J. Organomet. Chem.* **2002**, *654*, 176-179.

[4] T. Heitkemper, L. Naß, C. P. Sindlinger, *Dalton Trans.* **2020**, *49*, 2706 - 2714.

[5] T. Heitkemper, J. Sarcevic, C. P. Sindlinger, *J. Am. Chem. Soc.* **2020**, *142*, 21304-21309.

[6] J. Sarcevic, T. Heitkemper, P. N. Ruth, L. Naß, M. Kubis, D. Stalke, C. P. Sindlinger, *Chem. Sci.* **2023**, *14*, 5148-5159.

[7] J. Sarcevic, T. Heitkemper, C. P. Sindlinger, *Chem. Commun.* **2022**, *58*, 246-249.

[8] SAINTv8.30C, Bruker AXS, WI, USA, Madison, **2013**.

[9] G. M. Sheldrick, **SADABS, 2008 University of Göttingen, Göttingen, Germany**.

[10] a) R. O. Diffraction, Rigaku Corporation, Wroclaw, Poland, **2025**; b) R. O. Diffraction, Rigaku Corporation, Wroclaw, Polan, **2025**.

[11] a) S. Cie, Stoe & Cie, Darmstadt, Germany, **2016**; b) J. Koziskova, F. Hahn, J. Richter, J. Kožíšek, *Acta Chimica Slovaca* **2016**, *9*, 136-140.

[12] G. M. Sheldrick, *Acta Cryst.* **2015**, *A71*, 3.

[13] G. M. Sheldrick, *Acta Cryst.* **2015**, *C71*, 3.

[14] C. B. Hübschle, G. M. Sheldrick, B. Dittrich, *J. Appl. Crystallogr.* **2011**, *44*, 1281-1284.

[15] D. Kratzert, I. Krossing, *J. Appl. Crystallogr.* **2018**, *51*, 928-934.

[16] a) A. Bondi, *J. Phys. Chem.* **1964**, *68*, 441-451; b) M. Mantina, A. C. Chamberlin, R. Valero, C. J. Cramer, D. G. Truhlar, *J. Phys. Chem. A* **2009**, *113*, 5806-5812.

[17] a) F. Neese, *Wiley Interdiscip. Rev. Comput. Mol. Sci.* **2012**, *2*, 73-78; b) F. Neese, *Wiley Interdiscip. Rev. Comput. Mol. Sci.* **2018**, *8*, e1327.

[18] S. Grimme, S. Ehrlich, L. Goerigk, *J. Comput. Chem.* **2011**, *32*, 1456-1465.

[19] a) A. D. Becke, *Phys. Rev. A* **1988**, *38*, 3098-3100; b) J. P. Perdew, W. Yue, *Phys. Rev. B* **1986**, *33*, 8800-8802; c) A. Schäfer, C. Huber, R. Ahlrichs, *J. Chem. Phys.* **1994**, *100*, 5829-5835; d) F. Weigend, R. Ahlrichs, *Phys. Chem. Chem. Phys.* **2005**, *7*, 3297-3305; e) K. Eichkorn, F. Weigend, O. Treutler, R. Ahlrichs, *Theor. Chem. Acc.* **1997**, *97*, 119-124.

[20] a) E. D. Glendening, C. R. Landis, F. Weinhold, *Wiley Interdiscip. Rev. Comput. Mol. Sci.* **2012**, *2*, 1-42; b) E. D. Glendening, J. K. Badenhoop, A. E. Reed, J. E. Carpenter, J. A. Bohmann, C. M. Morales, P. Karafiloglou, C. R. Landis, F. Weinhold, Theoretical Chemical Institute, University of Wisconsin Madison, **2018**.

[21] a) G. A. Zhurko, Version 1.7 (build132) ed., **2014**; b) G. Knizia, J. E. M. N. Klein, *Angew. Chem. Int. Ed.* **2015**, *54*, 5518-5522; c) G. Knizia, v20150427 ed., **2015**.

[22] D. Andrae, U. Häußermann, M. Dolg, H. Stoll, H. Preuß, *Theor. Chim. Acta* **1990**, *77*, 123-141.

[23] a) F. Neese, *WIREs Computational Molecular Science* **2025**, *15*, e70019; b) F. Neese, *J. Comput. Chem.* **2003**, *24*, 1740-1747; c) F. Neese, F. Wennmohs, A. Hansen, U. Becker, *Chem. Phys.* **2009**, *356*, 98-109; d) D. Bykov, T. Petrenko, R. Izsák, S. Kossmann, U. Becker, E. Valeev, F. Neese, *Mol. Phys.* **2015**, *113*, 1961-1977; e) F. Neese, *J. Comput. Chem.* **2023**, *44*, 381-396; f) H. Kruse, S. Grimme, *J. Chem. Phys.* **2012**, *136*; g) E. Caldeweyher, C. Bannwarth, S. Grimme, *J. Chem. Phys.* **2017**, *147*; h) E. Caldeweyher, S. Ehlert, A. Hansen, H. Neugebauer, S. Spicher, C. Bannwarth, S. Grimme, *J. Chem. Phys.* **2019**, *150*; i) E. Caldeweyher, J.-M. Mewes, S. Ehlert, S. Grimme, *Phys. Chem. Chem. Phys.* **2020**, *22*, 8499-8512.

[24] S. Grimme, A. Hansen, S. Ehlert, J.-M. Mewes, *J. Chem. Phys.* **2021**, *154*.

[25] M. P. Mitoraj, A. Michalak, T. Ziegler, *J. Chem. Theory Comput.* **2009**, *5*, 962-975.

[26] E. Van Lenthe, E. J. Baerends, *J. Comput. Chem.* **2003**, *24*, 1142-1156.
[27] a) G. te Velde, F. M. Bickelhaupt, E. J. Baerends, C. Fonseca Guerra, S. J. A. van Gisbergen, J. G. Snijders, T. Ziegler, *J. Comput. Chem.* **2001**, *22*, 931-967; b) SCM, SCM, Theoretical Chemistry, Vrije Universiteit, Amsterdam, The Netherlands, **2022**.
